# oggmap: a Python package to extract gene ages per orthogroup and link them with single-cell RNA data

**DOI:** 10.1093/bioinformatics/btad657

**Published:** 2023-11-11

**Authors:** Kristian K Ullrich, Nikoleta E Glytnasi

**Affiliations:** Department for Evolutionary Genetics, Max Planck Institute for Evolutionary Biology, 24306 Plön, Germany; Max Planck Research Group: Dynamics of Social Behavior, Max Planck Institute for Evolutionary Biology, 24306 Plön, Germany

## Abstract

**Summary:**

For model species, single-cell RNA-based cell atlases are available. A good cell atlas includes all major stages in a species’ ontogeny, and soon, they will be standard even for nonmodel species. Here, we propose a Python package called *oggmap*, which allows for the easy extraction of an orthomap (gene ages per orthogroup) for any given query species from OrthoFinder and other gene family data resources, like homologous groups from eggNOG or PLAZA. *oggmap* provides extracted gene ages for more than thousand eukaryotic species which can be further used to calculate gene age-weighted expression data from scRNA sequencing objects using the Python Scanpy toolkit. Not limited to one transcriptome evolutionary index, *oggmap* can visualize the individual gene category (e.g. age class, nucleotide diversity bin) and their corresponding expression profiles to investigate scRNA-based cell type assignments in an evolutionary context.

**Availability and implementation:**

*oggmap* source code is available at https://github.com/kullrich/oggmap, documentation is available at https://oggmap.readthedocs.io/en/latest/. *oggmap* can be installed via PyPi or directly used via a docker container.

## 1 Introduction

In recent years, the availability of single-cell RNA (scRNA) sequencing data and its analysis tools has constantly been on the rise. However, using phylogenetic information with scRNA data to, e.g. better predict cell types in a cross-species manner is so far sparse (Tarashansky *et al.* 2021). Linking gene age with RNA sequencing data to better explain the developmental stages of an organism in the context of evolution has been a topic for over a decade (Domazet-Lošo *et al.* 2007, Domazet-Lošo and Tautz 2010, Quint *et al.* 2012, Liu and Robinson-Rechavi 2018, Liu *et al.* 2020, Ma and Zheng 2023). Methods to infer phyletic pattern of genes for a given query species are still a time-consuming step and as such a bottleneck to weight expression of a gene by its gene age. Once gene ages are inferred, the transcriptome age index (short TAI) can distinguish between a “young” and an “old” transcriptome to, e.g. investigate the hourglass model of embryonic development (Domazet-Lošo and Tautz 2010, Ma and Zheng 2023) or to highlight cell type-specific enrichment patterns (Cazet *et al.* 2022). TAI calculation is already implemented in the R *myTAI* package, primarily working with bulk-RNA data (Drost *et al.* 2018), so far lacking a Python supplement.

Since introducing TAI, a variety of analysis types have been created to link and weight transcriptome data with an evolutionary age or a different evolutionary index, like gene substitution rates or promoter conservation score (Quint *et al.* 2012, Drost *et al.* 2015, Gossmann *et al.* 2016, Liu *et al.* 2020, Ma *et al.* 2021). As a consequence, not be limited to age, we use the parent term transcriptome evolutionary index (short TEI) as introduced by Liu and Robinson-Rechavi (2018). The transition of the TEI analysis from bulk-RNA data to scRNA data, which both rely on an evolutionary age category, just recently begun (Cazet *et al.* 2022, Ma and Zheng 2023). If based on blast sequence searches, the resulting gene ages are collected for each individual gene (so-called *phylostratigraphic maps*). The age assignment is based on the “oldest” found homolog along the tree of life.

In contrast to the original implementation (Domazet-Lošo and Tautz 2010) using blast hits to extract gene ages, orthologous groups can be used (Ruprecht *et al.* 2017). This will assign gene ages not per gene, but will assign the same evolutionary age to all genes of a given *orthogroup*. It is important to note that to extract the gene age for a given *orthogroup*, one needs to define a query species to start from (sometimes called focal species). Given a query species, all other species members of the same *orthogroup* are compared using a species tree to extract the lowest common ancestor (LCA). In other words, the deepest node from the query species tip to the root node or the last universal common ancestor of the species tree (Julca *et al.* 2021). We call the resulting gene age classification ***o**rtholo**g**ous**g**roups****map*** (short *orthomap*).

## 2 Oggmap implementation

**Figure 1.**
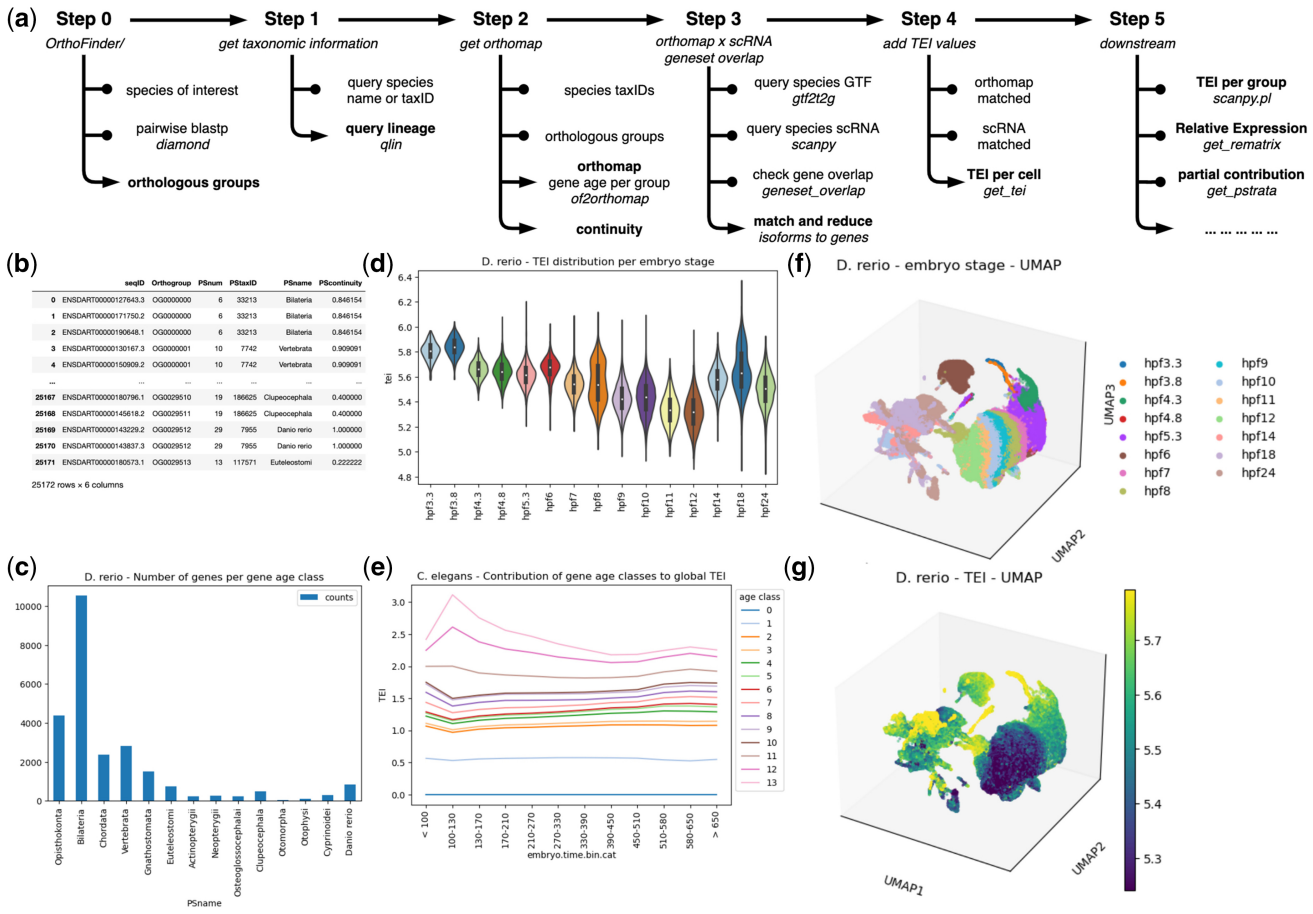
(a) Overview of *oggmap* steps. (b) *Orthomap* for the query species zebrafish (*Danio rerio*). Each gene (seqID) from an Orthogroup is assigned to a gene age class (PSnum, PSname) given a continuity score (PScontinuity). (c) Number of genes per gene age class. (d) Boxplot of zebrafish (*D.rerio*) TEI values grouped per embryo stage. (e) Contribution of gene age classes to the global TEI per embryo time (nematode). Zebrafish scRNA data UMAP, each cell is colored by embryo stage (f) or its corresponding TEI value (g).

*oggmap* (implemented in Python) uses different bioinformatic methods for importing, analyzing, and visualizing. All main steps of *oggmap* are illustrated in Fig. 1a. The documentation of *oggmap* will guide the user through all necessary steps. *oggmap* relies on orthogroups and can parse so far results from either OrthoFinder (Emms and Kelly 2019), or precalculated gene family databases, like eggNOG (Hernández-Plaza *et al.* 2023) or PLAZA (Van Bel *et al.* 2022). *oggmap* provides gene age class assignments for a high number of species (Ensembl release-110: 317; eggNOG v6: 1322, PLAZA v5: 98 dicots and 52 monocots), so that a researcher might start directly to analyze scRNA data for a given query species of interest. If the query species is not among them, the guide will help how to perform the mandatory step 0. All further steps to create an *orthomap* (steps 1 and 2) can be run on command line as well as using the Python API. Given a query species, the taxonomic lineage information will be used (*qlin module*) to assign an age class per *orthogroup* based on the LCA (*of2orthomap module*). Internally, these steps use the Python toolkit *ete3* (Huerta-Cepas *et al.* 2016) to fetch NCBI taxonomic tree information. In addition, for each *orthogroup* a continuity score can be optionally calculated, which represents the species completeness along the tree nodes from the LCA to the focal species. A low continuity score would thereby highlight, e.g. a possible horizontal gene transfer event, where intermediate tree nodes lack any detectable *orthologs*. All further steps (3, 4, and downstream analysis 5) to match gene names from the *orthomap* and scRNA data (in case of different annotation source; *gtf2t2g module*), calculate TEI, and visualizing the results (*orthomap2tei module*) are run with the Python API in, e.g. a Jupyter notebook (Fig. 1b–g). Internally, NumPy (Harris *et al.* 2020), pandas *DataFrame* objects (McKinney *et al.* 2011), and the Scanpy toolkit (Wolf *et al.* 2018) with *AnnData* objects are the working horses.

Dealing with scRNA data introduces a computational burden to TEI calculation, since unlike for bulk-RNA data with a rather low number of stages, now thousand of cells need to be processed. TEI is implemented as given in Domazet-Lošo and Tautz (2010) and due to sparse-matrix calculation scales for millions of cells. TEI represents the weighted arithmetic mean (expression levels as weights for the age category) over all evolutionary age categories denoted as *phylostra*.

(1) $${\text{TEI}}_{c}=\sum \left(e_{ic}*ps_{i}\right)/\sum e_{ic}.$$

${\text{TEI}}_{c}$ denotes the TEI value in a cell or a cell type *c*, $e_{ic}$ denotes the gene expression level of gene *i* in cell *c*, and $ps_{i}$ denotes the corresponding *phylostratum* of gene *i*, i=1,…,N, where *N* is the total number of genes.

Next to adding TEI values to scRNA data (*get_tei*), other useful function from the *myTAI* R package (Drost *et al.* 2018) has been ported to Python and extended to deal with cell-type groups. For example, one can calculate partial TEI values (*get_pstrata*) to visualize the contribution of each gene age class to the global TEI pattern. Or extract the relative expression per gene age class grouped by any annotated observation like cell-type or sampling timepoint starting either from raw counts or using the implemented expression transformation options (*get_rematrix*). Other gene based metric, like Tajima’s D (Tajima 1989) or F-statistics (Wright 1965) can be binned and used as gene groups to weigh expression (*get_bins*).

## 3 Case study: re-analysis of zebrafish (*Danio rerio*) and nematode (*Caenorhabditis elegans*) single-cell data

To demonstrate *oggmap*, we re-analyzed a single-cell dataset of combined and integrated ∼70 000 zebrafish cells (Farrell *et al.* 2018, Wagner *et al.* 2018, Qiu *et al.* 2022) and ∼90 000 nematode cells (Packer *et al.* 2019). A detailed Jupyter notebook to reproduce the case study for zebrafish (*D.rerio*) and nematode (*C.elegans*) is shown in the Supplementary Material.

In brief, to obtain an *orthomap* for zebrafish, we first run OrthoFinder (-S last) (Kiełbasa *et al.* 2011, Emms and Kelly 2019) to get orthologous groups for the complete species set of Ensembl release-110. Starting from coding sequences only the longest-isoform per gene was retained and converted into amino acid sequences (Ullrich 2020). For nematode, a pre-existing gene age map was imported (Sun *et al.* 2021).

Further, setting zebrafish as the focal species, we extracted the corresponding *orthomap* (Fig. 1b) and highlight the number of genes per gene age class (Fig. 1c). TEI was calculated for each cell and the distribution of TEI values visualized per developmental stage (Fig. 1d). As compared to the original publication, showing the hourglass model of embryonic development in zebrafish and the vertebrate phylotypic phase falling between 11- and 42-h postfertilization (hpf) (Domazet-Lošo and Tautz 2010), the re-analysis using scRNA data shows the lowest global TAI values at the 12-h stage (12 hpf). Here, like recently shown for *C.elegans* by Ma and Zheng (2023), this results confirms the “hourglass” pattern using embryonic zebrafish scRNA data. Researcher should now be able to investigate in more detail individual cell types that contribute to the development stages in the context of evolution (see Supplementary Material). The stacked partial TEI values (Fig. 1e) can highlight the contribution of each gene age class to the total TEI pattern. Here, the results confirm the findings of Ma and Zheng (2023) that the “youngest” genes specific to *C.elegans* and sister species (age class 11–13) peak in early embryos (Fig. 1e). Following the basic tutorial of Scanpy (Wolf *et al.* 2018) to get a dimensional reduction of the given scRNA data and the Uniform Manifold Approximation and Projection (UMAP) algorithm (McInnes *et al.* 2018), cells were visualized and colored by stage (Fig. 1f) or its corresponding TEI values (Fig. 1g).

Additional downstream analysis and other species, like *Xenopus tropicalis*, *Mus musculus*, and *Hydra vulgaris*, are available via the documentation of *oggmap* at https://oggmap.readthedocs.io/en/latest/.

## 4 Conclusion

*oggmap* is a versatile Python package to extract gene ages per orthologous group from OrthoFinder (Emms and Kelly 2019), eggNOG (Hernández-Plaza *et al.* 2023), and PLAZA (Van Bel *et al.* 2022) results and seamless integrate the resulting evolutionary age index with transcriptome data from scRNA datasets and the Scanpy toolkit (Wolf *et al.* 2018). *oggmap* can help the investigator to map gene and transcript names to be able to integrate nonstandard gene annotations (e.g. for species with only transcriptome assemblies). Next to evolutionary age indices, other indices like gene adaptation scores become relevant (Moutinho *et al.* 2022) and can be used to calculate TEI to look into cell-type specific pattern. With *oggmap*, evolutionary biologist, medical research data analysts, and the up-rising community of single-cell data researchers will be able to enrich their scRNA data by another layer, namely evolution.

## Supplementary Material

btad657_Supplementary_Data

## Data Availability

OrthoFinder (Emms and Kelly 2019) results for Ensembl release-110, including species taxonomic IDs, are available here: https://doi.org/10.5281/zenodo.7242263. For each species of the eggNOG database v6.0 (Hernández-Plaza *et al.* 2023) and each species of the PLAZA database v5.0 (Van Bel *et al.* 2022), an *orthomap* is available here: https://doi.org/10.5281/zenodo.7242263. scRNA data for zebrafish (*Danio rerio*), frog (*Xenopus tropicalis*), and mouse (*Mus musculus*) were obtained from https://tome.gs.washington.edu(Qiu *et al.* 2022), converted into Scanpy AnnData objects (Wolf *et al.* 2018) and are available here: https://doi.org/10.5281/zenodo.7243602, https://doi.org/10.5281/zenodo.7244440, and https://doi.org/10.5281/zenodo.7244567. scRNA data for *Caenorhabditis elegans* (Packer *et al.* 2019) were obtained from https://www.ncbi.nlm.nih.gov/geo/ using the accession number GSE126954, converted into Scanpy AnnData objects (Wolf *et al.* 2018), and are available here: https://doi.org/10.5281/zenodo.7245548. Precalculated gene age assignments were obtained from Sun *et al.* (2021). Precalculated gene adaptation indices were obtained from Ma *et al.* (2021).
