# Supplementary material for: oggmap: a Python package to extract gene ages per orthogroup and link them with single-cell RNA data
Source: Bioinformatics. 2023 Nov 11;39(11):btad657. doi: 10.1093/bioinformatics/btad657 (PMC10663984; doi:10.1093/bioinformatics/btad657)
Supplement: btad657_Supplementary_Data [file btad657_supplementary_data.pdf]

Supplementary data
oggmap: a Python package to extract gene ages per orthogroup and link them with single-cell RNA data

Please visit https://oggmap.readthedocs.io/en/latest/index.html to see the documentation of `oggmap`.

`oggmap` tutorials (jupyter notebooks are better displayed) can be found here:

https://oggmap.readthedocs.io/en/latest/tutorials/index.html

1 Case study: re-analysis of zebrafish (*Danio rerio*) embryogenesis single-cell data

This notebook will demonstrate scRNA-seq processing with `oggmap` using zebrafish scRNA data from (Farrell et al., 2018; Wagner et al., 2018; Qiu et al., 2022).

scRNA data were obtained from http://tome.gs.washington.edu/, converted into Scanpy `AnnData` objects (Wolf et al., 2018) and are availabe here:

https://doi.org/10.5281/zenodo.7243602

or can be accessed with the `dataset` submodule of `oggmap`

`datasets.qiu22_zebrafish(datapath='data')` (download folder set to `'data'`).

1.1 Notebook file

Notebook file can be obtained here:

`https://raw.githubusercontent.com/kullrich/oggmap/main/docs/notebooks/zebrafish_example.ipynb`

1.2 Steps

To process the scRNA data, we will do the following:

0. Run OrthoFinder to obtain orthogroups
1. Get query species taxonomic lineage information
2. Get query species orthomap
3. Map OrthoFinder gene names and scRNA gene/transcript names
4. Get TEI values and add them to scRNA dataset
5. Get partial TEI values to visualize gene age class contributions
6. Process scRNA data and visualize TEI

1.3 Import libraries

[1]:
```
import numpy as np
import pandas as pd
import scanpy as sc
import seaborn as sns
import matplotlib.pyplot as plt
from statannot import add_stat_annotation
# increase dpi
%matplotlib inline
#plt.rcParams['figure.dpi'] = 300
#plt.rcParams['savefig.dpi'] = 300
#plt.rcParams['figure.figsize'] = [6, 4.5]
plt.rcParams['figure.figsize'] = [4.4, 3.3]
```

1.4 Import oggmap python package submodules

[2]:
```
# import submodules
from oggmap import qlin, gtf2t2g, of2orthomap, orthomap2tei, datasets
```

1.5 Step 0 - run OrthoFinder to obtain orthogroups

`oggmap` can extract gene age classification from existing OrthoFinder results and link them with scRNA data.

A detailed how-to is available here:

https://oggmap.readthedocs.io/en/latest/tutorials/orthofinder.html

However, any pre-calculated gene age classification can be imported as a table using the function `orthomap2tei.read_orthomap(orthomapfile=filename)`.

The pre-calculated gene age classification file should be delimited with two columns `GeneID<tab>Phylostratum`, like e.g.:

```
GeneID<tab>Phylostratum
WBGene00000001<tab>1
WBGene00000002<tab>1
WBGene00000003<tab>1
WBGene00000004<tab>1
WBGene00000005<tab>2
```

OrthoFinder (Emms and Kelly, 2019) results (-S last) using translated, longest-isoform coding sequences (CDS) from Ensembl release-110, including species taxonomic IDs, are available here:

https://doi.org/10.5281/zenodo.7242264

or can be accessed with the `dataset` submodule of `oggmap`

`datasets.ensembl110_last(datapath='data')` (download folder set to `'data'`).

[3]:
```
datasets.ensembl110_last(datapath='data')
```

```
 99% [... ] 1318912 /
1325809100% [...] 11317 /
11317
```

```
[3]: ['data/ensembl_110_orthofinder_last_Orthogroups.GeneCount.tsv.zip',
 'data/ensembl_110_orthofinder_last_Orthogroups.tsv.zip',
 'data/ensembl_110_orthofinder_last_species_list.tsv']
```

1.6 Step 1 - get query species taxonomic lineage information

Given a species name or taxonomic ID, the query species lineage information is extracted with the help of the `ete3` python toolkit and the `NCBI taxonomy` (Huerta-Cepas et al., 2016). This information is needed alongside with the taxonomic classifications for all species used in the OrthoFinder comparison.

The `oggmap` submodule `qlin` helps to get this information for you with the `qlin.get_qlin()` function as follows:

```
[4]: # get query species taxonomic lineage information
query_lineage = qlin.get_qlin(q='Danio rerio')
```

```
query name: Danio rerio
query taxID: 7955
query kingdom: Eukaryota
query lineage names:
['root(1)', 'cellular organisms(131567)', 'Eukaryota(2759)',
'Opisthokonta(33154)', 'Metazoa(33208)', 'Eumetazoa(6072)', 'Bilateria(33213)',
'Deuterostomia(33511)', 'Chordata(7711)', 'Craniata(89593)', 'Vertebrata(7742)',
'Gnathostomata(7776)', 'Teleostomi(117570)', 'Euteleostomi(117571)',
'Actinopterygii(7898)', 'Actinopteri(186623)', 'Neopterygii(41665)',
'Teleostei(32443)', 'Osteoglossocephalai(1489341)', 'Clupeocephala(186625)',
'Otomorpha(186634)', 'Ostariophysi(32519)', 'Otophysi(186626)',
'Cypriniphysae(186627)', 'Cypriniformes(7952)', 'Cyprinoidei(30727)',
'Danionidae(2743709)', 'Danioninae(2743711)', 'Danio(7954)', 'Danio
rerio(7955)']
query lineage:
[1, 131567, 2759, 33154, 33208, 6072, 33213, 33511, 7711, 89593, 7742, 7776,
117570, 117571, 7898, 186623, 41665, 32443, 1489341, 186625, 186634, 32519,
186626, 186627, 7952, 30727, 2743709, 2743711, 7954, 7955]
```

1.7 Step 2 - gene age class assignment (query species orthomap)

Here, `oggmap` use the query species information and OrthoFinder results to extract the oldest common tree node per orthogroup along a species tree and to assign this node as the gene age to the corresponding genes.

In a pairwise manner, the query species and any other species in the OrthoFinder result might share multiple tree nodes down to the root of the species tree, but have only one youngest tree node in common. Among all possible comparison between the query species and the other species, the oldest as defined by the species tree root is seected and used for the gene age assignment.

Given the `query species sequence name` (`seqname=`) used in the OrthoFinder comparison, the `query species taxonomic ID`(`qt=`), the `taxonomic IDs of all species` (`sl=`) used in the OrthoFinder comparison, the `orthogroup gene count` (`oc=`) results and the `orthogroups` (`og=`), an orthomap is constructed.

Note: This step can take up to five minutes, depending on your hardware.

For this step to get the query species orthomap, one uses the `of2orthomap.get_orthomap()` function, like:

corresponds to main figure Figure 1B

```
[5]: # get query species orthomap
```

```python
# download orthofinder results here: https://doi.org/10.5281/zenodo.7242264
# or download with datasets.ensembl110_last('data')
query_orthomap, orthofinder_species_list, of_species_abundance =\
    of2orthomap.get_orthomap(
    seqname='7955.danio_rerio.pep',
    qt='7955',
    sl='data/ensembl_110_orthofinder_last_species_list.tsv',
    oc='data/ensembl_110_orthofinder_last_Orthogroups.GeneCount.tsv.zip',
    og='data/ensembl_110_orthofinder_last_Orthogroups.tsv.zip',
    continuity=True)
query_orthomap
```

```
7955.danio_rerio.pep
Danio rerio
7955
                                     species  taxID  \
0                   10020.dipodomys_ordii.pep  10020
1    10029.cricetulus_griseus_chok1gshd.pep  10029
2       10029.cricetulus_griseus_crigri.pep  10029
3         10029.cricetulus_griseus_picr.pep  10029
4            10036.mesocricetus_auratus.pep  10036
..                                       ...    ...
313          9986.oryctolagus_cuniculus.pep   9986
314        99883.tetraodon_nigroviridis.pep  99883
315        9994.marmota_marmota_marmota.pep   9994
316            9999.urocitellus_parryii.pep   9999
317   Xtropicalisv9.0.Named.primaryTrs.pep   8364

                                               lineage  youngest_common  \
0    [1, 131567, 2759, 33154, 33208, 6072, 33213, 3...           117571
1    [1, 131567, 2759, 33154, 33208, 6072, 33213, 3...           117571
2    [1, 131567, 2759, 33154, 33208, 6072, 33213, 3...           117571
3    [1, 131567, 2759, 33154, 33208, 6072, 33213, 3...           117571
4    [1, 131567, 2759, 33154, 33208, 6072, 33213, 3...           117571
..                                                 ...              ...
313  [1, 131567, 2759, 33154, 33208, 6072, 33213, 3...           117571
314  [1, 131567, 2759, 33154, 33208, 6072, 33213, 3...           186625
315  [1, 131567, 2759, 33154, 33208, 6072, 33213, 3...           117571
316  [1, 131567, 2759, 33154, 33208, 6072, 33213, 3...           117571
317  [1, 131567, 2759, 33154, 33208, 6072, 33213, 3...           117571

     youngest_name
0     Euteleostomi
1     Euteleostomi
2     Euteleostomi
3     Euteleostomi
4     Euteleostomi
..             ...
313   Euteleostomi
314  Clupeocephala
315   Euteleostomi
316   Euteleostomi
317   Euteleostomi
```

```
[318 rows x 5 columns]
```

[5]:

```
                        seqID Orthogroup  PSnum PStaxID         PSname  \
0      ENSDART00000013359.10  OG0000000      6   33213      Bilateria
1       ENSDART00000136092.3  OG0000001      6   33213      Bilateria
2       ENSDART00000148349.3  OG0000001      6   33213      Bilateria
3       ENSDART00000160249.2  OG0000001      6   33213      Bilateria
4       ENSDART00000174091.2  OG0000001      6   33213      Bilateria
...                      ...        ...    ...     ...            ...
24952   ENSDART00000125904.3  OG0035492     19  186625  Clupeocephala
24953   ENSDART00000191935.1  OG0035493     25   30727    Cyprinoidei
24954   ENSDART00000143229.2  OG0035494     29    7955    Danio rerio
24955   ENSDART00000143837.3  OG0035494     29    7955    Danio rerio
24956   ENSDART00000143384.2  OG0035495     22  186626       Otophysi

       PScontinuity
0          0.846154
1          1.000000
2          1.000000
3          1.000000
4          1.000000
...             ...
24952      0.400000
24953      1.000000
24954      1.000000
24955      1.000000
24956      0.666667

[24957 rows x 6 columns]
```

1.7.1 Gene age assignments per query species lineage node

Given an orthomap, one can get an overview of the gene age assignments per query species lineage node.

The `oggmap` submodule `of2orhomap` and the `of2orthomap.get_counts_per_ps()` function will show the distribution of the gene age classes and can be further visualized as follows:

[6]:

```python
# show count per taxonomic group (PStaxID)
of2orthomap.get_counts_per_ps(query_orthomap)
```

[6]:

```
       PSnum  counts  PStaxID               PSname
PSnum
3          3    4376    33154         Opisthokonta
6          6   10530    33213            Bilateria
8          8    2390     7711             Chordata
10        10    2838     7742           Vertebrata
11        11    1515     7776         Gnathostomata
13        13     760   117571         Euteleostomi
14        14     235     7898       Actinopterygii
16        16     275    41665          Neopterygii
18        18     231  1489341  Osteoglossocephalai
19        19     506   186625        Clupeocephala
20        20      33   186634            Otomorpha
22        22     120   186626             Otophysi
```

25	25	317	30727	Cyprinoidei
29	29	831	7955	Danio rerio

1.7.2 Visualize number of species along query lineage and counts per gene age class

[7]:
```python
# show number of species along query lineage
of_species_abundance

# bar plot number of species along query lineage
of_species_abundance.plot.bar(y='counts', use_index=True)
```

[7]: <AxesSubplot: >

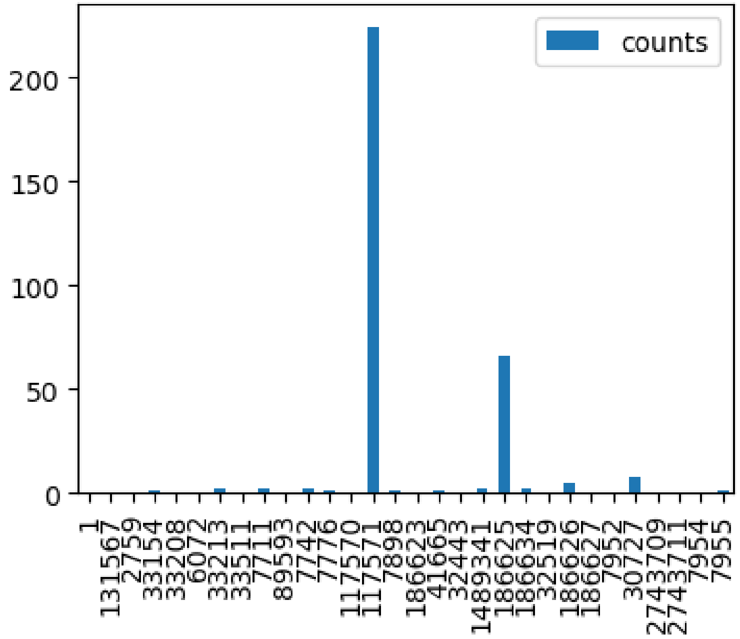

corresponds to main figure Figure 1C

[8]:
```python
plt.rcParams['figure.figsize'] = [6.5, 4.5]
# show count per taxonomic group (PStaxID)
of2orthomap.get_counts_per_ps(query_orthomap)

# bar plot count per taxonomic group (PSname)
ax = of2orthomap.get_counts_per_ps(query_orthomap).plot.bar(y='counts', x='PSname')
ax.set_title('D. rerio - Number of genes per gene age class')
plt.show()
plt.rcParams['figure.figsize'] = [4.4, 3.3]
```

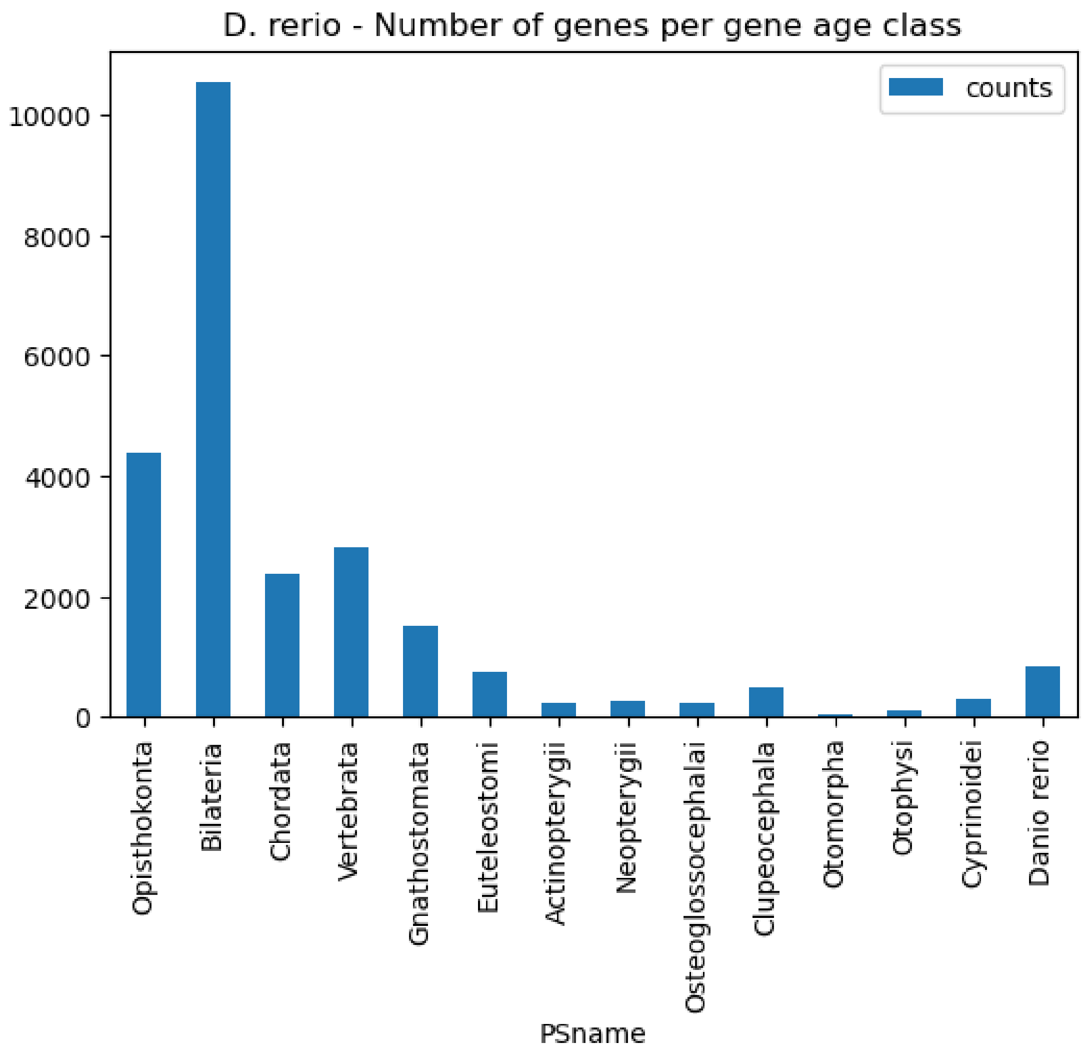

1.8 Step 3 - map OrthoFinder gene names and scRNA gene/transcript names

To be able to link gene ages assignments from an orthomap and gene or transcript of scRNA dataset, one needs to check the overlap of the annotated gene names. With the `gtf2t2g` submodule of `oggmap` and the `gtf2t2g.parse_gtf()` function, one can extract gene and transcript names from a given gene feature file (GTF).

[9]:
```
datasets.zebrafish_ensembl110_gtf('data')
```

```
100% [...] 18055420 / 18055420
```

[9]:
```
'data/Danio_rerio.GRCz11.110.gtf.gz'
```

[10]:
```
# get gene to transcript table for Danio rerio

# download zebrafish GTF file here:
# https://ftp.ensembl.org/pub/release-110/gtf/danio_rerio/Danio_rerio.GRCz11.110.gtf.gz
```

```python
# or download with datasets.zebrafish_ensembl110_gtf('data')
query_species_t2g = gtf2t2g.parse_gtf(
    gtf='data/Danio_rerio.GRCz11.110.gtf.gz',
    g=True, b=True, p=True, v=True, s=True, q=True)
```

```
32520 gene_id found
59876 transcript_id found
59876 protein_id found
0 duplicated
```

[11]:
```python
query_species_t2g
```

[11]:
```
                  gene_id       gene_id_version       transcript_id  \
0      ENSDARG00000000001  ENSDARG00000000001.6  ENSDART00000000004
1      ENSDARG00000000002  ENSDARG00000000002.8  ENSDART00000000005
2      ENSDARG00000000018  ENSDARG00000000018.9  ENSDART00000181044
3      ENSDARG00000000018  ENSDARG00000000018.9  ENSDART00000138183
4      ENSDARG00000000019  ENSDARG00000000019.9  ENSDART00000124452
...                   ...                   ...                 ...
59871  ENSDARG00000117825  ENSDARG00000117825.1  ENSDART00000194739
59872  ENSDARG00000117826  ENSDARG00000117826.1  ENSDART00000194042
59873  ENSDARG00000117826  ENSDARG00000117826.1  ENSDART00000194514
59874  ENSDARG00000117827  ENSDARG00000117827.1  ENSDART00000194378
59875  ENSDARG00000117827  ENSDARG00000117827.1  ENSDART00000194710

      transcript_id_version gene_name       gene_type          protein_id  \
0      ENSDART00000000004.5   slc35a5  protein_coding  ENSDARP00000000004
1      ENSDART00000000005.7    ccdc80  protein_coding  ENSDARP00000000005
2      ENSDART00000181044.1      nrf1  protein_coding  ENSDARP00000149440
3      ENSDART00000138183.2      nrf1  protein_coding  ENSDARP00000116798
4      ENSDART00000124452.3     ube2h  protein_coding  ENSDARP00000107407
...                     ...       ...             ...                 ...
59871  ENSDART00000194739.1      None         lincRNA                None
59872  ENSDART00000194042.1      None         lincRNA                None
59873  ENSDART00000194514.1      None         lincRNA                None
59874  ENSDART00000194378.1      None         lincRNA                None
59875  ENSDART00000194710.1      None         lincRNA                None

         protein_id_version
0      ENSDARP00000000004.2
1      ENSDARP00000000005.6
2      ENSDARP00000149440.1
3      ENSDARP00000116798.1
4      ENSDARP00000107407.2
...                     ...
59871                  None
59872                  None
59873                  None
59874                  None
59875                  None

[59876 rows x 8 columns]
```

1.8.1 Import now, the scRNA dataset of the query species

Here, data is used, like in the publication (Farrell et al., 2018; Wagner et al., 2018; Qiu et al., 2022).

scRNA data was downloaded from http://tome.gs.washington.edu/ as R rds files, combined into a single Seurat object and converted into loom and AnnData (h5ad) files to be able to analyse with e.g. python scanpy or `oggmap` package and is available here:

https://doi.org/10.5281/zenodo.7243602

or can be accessed with the `dataset` submodule of `oggmap`:

`datasets.qiu22_zebrafish(datapath='data')` (download folder set to `'data'`).

[12]:
```
# load scRNA data

# download zebrafish scRNA data here: https://doi.org/10.5281/zenodo.7243602
# or download with datasets.qui22_zebrafish(datapath='data')

#zebrafish_data = datasets.qiu22_zebrafish(datapath='data')
zebrafish_data = sc.read('data/zebrafish_data.h5ad')
```

1.8.2 Get an overview of observations

[13]:
```
zebrafish_data
```

[13]:
```
AnnData object with n_obs × n_vars = 71203 × 20418
    obs: 'orig.ident', 'nCount_RNA', 'nFeature_RNA', 'sample', 'stage', 'group',
'cell_state', 'cell_type'
    var: 'features', 'genes'
```

[14]:
```
zebrafish_data.obs
```

[14]:
```
                                  orig.ident  nCount_RNA  nFeature_RNA  \
hpf3.3_ZFHIGH_WT_DS5_AAAAGTTGCCTC     ZFHIGH      5773.0          2570
hpf3.3_ZFHIGH_WT_DS5_AAACAAGTGTAT     ZFHIGH      2312.0          1451
hpf3.3_ZFHIGH_WT_DS5_AAACACCTCGTC     ZFHIGH      4180.0          2166
hpf3.3_ZFHIGH_WT_DS5_AAATGAGGTTTN     ZFHIGH      6686.0          2845
hpf3.3_ZFHIGH_WT_DS5_AACCCTCTCGAT     ZFHIGH     20095.0          4993
...                                      ...         ...           ...
hpf24_DEW057_TGACACAACAG_GCCACATC     DEW057      3916.0          1328
hpf24_DEW057_CTTACGGG_AACCTGAC        DEW057      5611.0          1700
hpf24_DEW057_TGAACATCTAT_GACGATGG     DEW057      3676.0          1345
hpf24_DEW057_TGAGGTTTCTC_CTCAGAAT     DEW057      7021.0          1778
hpf24_DEW057_ACGTGCTAG_CAAGTCAT       DEW057      3378.0          1170

                                                             sample   stage  \
hpf3.3_ZFHIGH_WT_DS5_AAAAGTTGCCTC   ZFHIGH_WT_DS5_AAAAGTTGCCTC  hpf3.3
hpf3.3_ZFHIGH_WT_DS5_AAACAAGTGTAT   ZFHIGH_WT_DS5_AAACAAGTGTAT  hpf3.3
hpf3.3_ZFHIGH_WT_DS5_AAACACCTCGTC   ZFHIGH_WT_DS5_AAACACCTCGTC  hpf3.3
hpf3.3_ZFHIGH_WT_DS5_AAATGAGGTTTN   ZFHIGH_WT_DS5_AAATGAGGTTTN  hpf3.3
hpf3.3_ZFHIGH_WT_DS5_AACCCTCTCGAT   ZFHIGH_WT_DS5_AACCCTCTCGAT  hpf3.3
...                                                         ...     ...
hpf24_DEW057_TGACACAACAG_GCCACATC  DEW057_TGACACAACAG_GCCACATC   hpf24
hpf24_DEW057_CTTACGGG_AACCTGAC        DEW057_CTTACGGG_AACCTGAC   hpf24
```

```
hpf24_DEW057_TGAACATCTAT_GACGATGG  DEW057_TGAACATCTAT_GACGATGG   hpf24
hpf24_DEW057_TGAGGTTTCTC_CTCAGAAT  DEW057_TGAGGTTTCTC_CTCAGAAT   hpf24
hpf24_DEW057_ACGTGCTAG_CAAGTCAT      DEW057_ACGTGCTAG_CAAGTCAT   hpf24

                                    group            cell_state  \
hpf3.3_ZFHIGH_WT_DS5_AAAAGTTGCCTC   F_3.3      hpf3.3:blastomere
hpf3.3_ZFHIGH_WT_DS5_AAACAAGTGTAT   F_3.3      hpf3.3:blastomere
hpf3.3_ZFHIGH_WT_DS5_AAACACCTCGTC   F_3.3      hpf3.3:blastomere
hpf3.3_ZFHIGH_WT_DS5_AAATGAGGTTTN   F_3.3      hpf3.3:blastomere
hpf3.3_ZFHIGH_WT_DS5_AACCCTCTCGAT   F_3.3      hpf3.3:blastomere
...                                   ...                    ...
hpf24_DEW057_TGACACAACAG_GCCACATC  batch2         hpf24:midbrain
hpf24_DEW057_CTTACGGG_AACCTGAC     batch2   hpf24:pharyngeal arch
hpf24_DEW057_TGAACATCTAT_GACGATGG  batch2         hpf24:midbrain
hpf24_DEW057_TGAGGTTTCTC_CTCAGAAT  batch2        hpf24:optic cup
hpf24_DEW057_ACGTGCTAG_CAAGTCAT    batch2  hpf24:hindbrain dorsal

                                          cell_type
hpf3.3_ZFHIGH_WT_DS5_AAAAGTTGCCTC        blastomere
hpf3.3_ZFHIGH_WT_DS5_AAACAAGTGTAT        blastomere
hpf3.3_ZFHIGH_WT_DS5_AAACACCTCGTC        blastomere
hpf3.3_ZFHIGH_WT_DS5_AAATGAGGTTTN        blastomere
hpf3.3_ZFHIGH_WT_DS5_AACCCTCTCGAT        blastomere
...                                             ...
hpf24_DEW057_TGACACAACAG_GCCACATC          midbrain
hpf24_DEW057_CTTACGGG_AACCTGAC      pharyngeal arch
hpf24_DEW057_TGAACATCTAT_GACGATGG          midbrain
hpf24_DEW057_TGAGGTTTCTC_CTCAGAAT         optic cup
hpf24_DEW057_ACGTGCTAG_CAAGTCAT     hindbrain dorsal

[71203 rows x 8 columns]
```

1.8.3 Helper functions to match gene names

The `orthomap2tei` submodule contains the `orthomap2tei.geneset_overlap()` helper function to check for gene name overlap between the constructed orthomap from `OrthoFinder` results and a given scRNA dataset.

[15]:
```
# check overlap of orthomap <seqID> and scRNA data <var_names>
orthomap2tei.geneset_overlap(zebrafish_data.var_names, query_orthomap['seqID'])
```

[15]:
```
   g1_g2_overlap  g1_ratio  g2_ratio
0              0       0.0       0.0
```

[16]:
```
# check overlap of transcript table <gene_id> and scRNA data <var_names>
orthomap2tei.geneset_overlap(zebrafish_data.var_names, query_species_t2g['gene_id'])
```

[16]:
```
   g1_g2_overlap  g1_ratio  g2_ratio
0          20418       1.0   0.62786
```

1.8.4 Reduce isoforms to genes

The `of2orthomap.replace_by()` helper function can be used to add a new column to the orthomap dataframe by matching e.g. gene isoform names and their corresponding gene names.

[17]:
```python
query_orthomap
```

[17]:
```
                       seqID Orthogroup  PSnum PStaxID         PSname  \
0      ENSDART00000013359.10  OG0000000      6   33213      Bilateria
1       ENSDART00000136092.3  OG0000001      6   33213      Bilateria
2       ENSDART00000148349.3  OG0000001      6   33213      Bilateria
3       ENSDART00000160249.2  OG0000001      6   33213      Bilateria
4       ENSDART00000174091.2  OG0000001      6   33213      Bilateria
...                      ...        ...    ...     ...            ...
24952   ENSDART00000125904.3  OG0035492     19  186625  Clupeocephala
24953   ENSDART00000191935.1  OG0035493     25   30727    Cyprinoidei
24954   ENSDART00000143229.2  OG0035494     29    7955    Danio rerio
24955   ENSDART00000143837.3  OG0035494     29    7955    Danio rerio
24956   ENSDART00000143384.2  OG0035495     22  186626      Otophysi

       PScontinuity
0          0.846154
1          1.000000
2          1.000000
3          1.000000
4          1.000000
...             ...
24952      0.400000
24953      1.000000
24954      1.000000
24955      1.000000
24956      0.666667

[24957 rows x 6 columns]
```

[18]:
```python
zebrafish_data.var_names
```

[18]:
```
Index(['ENSDARG00000002968', 'ENSDARG00000056314', 'ENSDARG00000102274',
       'ENSDARG00000012468', 'ENSDARG00000063621', 'ENSDARG00000044802',
       'ENSDARG00000011410', 'ENSDARG00000041170', 'ENSDARG00000011855',
       'ENSDARG00000103957',
       ...
       'ENSDARG00000078476', 'ENSDARG00000058562', 'ENSDARG00000110745',
       'ENSDARG00000114172', 'ENSDARG00000110433', 'ENSDARG00000098193',
       'ENSDARG00000101137', 'ENSDARG00000095817', 'ENSDARG00000079034',
       'ENSDARG00000063372'],
      dtype='object', name='index', length=20418)
```

[19]:
```python
# convert orthomap transcript IDs into GeneIDs and add them to orthomap
query_orthomap['geneID'] = orthomap2tei.replace_by(
    x_orig = query_orthomap['seqID'],
    xmatch = query_species_t2g['transcript_id_version'],
    xreplace = query_species_t2g['gene_id'],
)
```

[20]:
```python
query_orthomap
```

[20]:
```
                          seqID Orthogroup  PSnum PStaxID         PSname  \
0      ENSDART00000013359.10  OG0000000      6   33213      Bilateria
1       ENSDART00000136092.3  OG0000001      6   33213      Bilateria
2       ENSDART00000148349.3  OG0000001      6   33213      Bilateria
3       ENSDART00000160249.2  OG0000001      6   33213      Bilateria
4       ENSDART00000174091.2  OG0000001      6   33213      Bilateria
...                      ...        ...    ...     ...            ...
24952   ENSDART00000125904.3  OG0035492     19  186625  Clupeocephala
24953   ENSDART00000191935.1  OG0035493     25   30727    Cyprinoidei
24954   ENSDART00000143229.2  OG0035494     29    7955    Danio rerio
24955   ENSDART00000143837.3  OG0035494     29    7955    Danio rerio
24956   ENSDART00000143384.2  OG0035495     22  186626       Otophysi

       PScontinuity              geneID
0          0.846154  ENSDARG00000013014
1          1.000000  ENSDARG00000100568
2          1.000000  ENSDARG00000094216
3          1.000000  ENSDARG00000099070
4          1.000000  ENSDARG00000105690
...             ...                 ...
24952      0.400000  ENSDARG00000110323
24953      1.000000  ENSDARG00000114540
24954      1.000000  ENSDARG00000069978
24955      1.000000  ENSDARG00000078193
24956      0.666667  ENSDARG00000092452

[24957 rows x 7 columns]
```

[21]:
```
# check overlap of orthomap <geneID> and scRNA data
orthomap2tei.geneset_overlap(zebrafish_data.var_names, query_orthomap['geneID'])
```

[21]:
```
   g1_g2_overlap  g1_ratio  g2_ratio
0          19952  0.977177  0.799455
```

The created orthomap can be stored as a separated file like:

[22]:
```
query_orthomap.to_csv('./data/zebrafish_ensembl_110_orthomap.tsv',\
    sep='\t', index=False)
```

To re-use the saved orthomap, so that one does not need to repeat step1 and step2, one could load it with `orthomap2tei.read_orthomap()` function.

1.9 Step 4 - get TEI values and add them to scRNA dataset

Since now the gene names correspond to each other in the orthomap and the scRNA `adata` object, one can calculate the transcriptome evolutionary index (`TEI`) and add them to the scRNA dataset (`adata` object).

The `TEI` measure represents the weighted arithmetic mean (expression levels as weights for the phylostratum value) over all evolutionary age categories denoted as *phylostra*.

$TEI_s = \sum(e_{is} * ps_i)/\sum e_{is}$

, where TEI_s denotes the `TEI` value in developmental stage s, e_{is} denotes the gene expression level of gene i in stage s, and ps_i denotes the corresponding phylostratum of gene $i, i = 1, ..., N$ and $N = total\ number\ of\ genes$.

Note: If e.g. two different isoforms would fall into two different gene age classes, their gene ages might differ based on the oldest ortholog found in their corresponding orthologous groups. However, both isoforms share the same gene name and their gene ages would clash. In this case one can decide either to use the `keep='min'` or `keep='max'` gene age to be kept by the `get_tei` function, which defaults to keep in this cases the `keep='min'` or in other words the 'older' gene age.

To be able to re-use the original `count` data, they are added as a new `layer` to the `adata` object. This is useful because later on the `count` data can be used to extract either the relative expression per gene age class or re-calculate other metrics.

This can be done either on un-normalized `counts`, on `normalized` and `log-transformed` data.

[23]:
```
zebrafish_data.layers['counts'] = zebrafish_data.X
```

1.9.1 add TEI to adata object

Using the submodule `orthomap2tei` from `oggmap` and the `orthomap2tei.get_tei()` function, transcriptome evolutionary index (`TEI`) values are calculated and directyl added to the existing `adata` object (`add_obs=True`).

There are other options to e.g. not start from the `adata.X` `counts` but from another `layer` from the `adata` object, the default is to use the `adata.X` (`layer=None`). The values can be pre-processed by the `normalize_total` option and the `log1p` option.

If `add_obs=True` the resulting `TEI` values are added to the existing `adata` object as a new observation with the name set with the `obs_name` option.

If `add_var=True` the gene age values are added to the existing `adata` object as a new variable with the name set with the `var_name` option.

Note: Genes not assigned to any gene class will get a missing assignment.

If one wants to calculate bootstrap `TEI` values per cell, the `boot` option can be set to `boot=True` and gene age classes will be randomly chosen prior calculating `TEI` values `bt=10` times.

[24]:
```
# add TEI values to existing adata object
orthomap2tei.get_tei(adata=zebrafish_data,
    gene_id=query_orthomap['geneID'],
    gene_age=query_orthomap['PSnum'],
    keep='min',
    layer=None,
    add_var=True,
    var_name='Phylostrata',
    add_obs=True,
    obs_name='tei',
    boot=False,
    bt=10,
    normalize_total=True,
    log1p=True,
    target_sum=1e6)
```

[24]:

	tei
hpf3.3_ZFHIGH_WT_DS5_AAAAGTTGCCTC	5.847391
hpf3.3_ZFHIGH_WT_DS5_AAACAAGTGTAT	5.794241
hpf3.3_ZFHIGH_WT_DS5_AAACACCTCGTC	5.783182
hpf3.3_ZFHIGH_WT_DS5_AAATGAGGTTTN	5.780480
hpf3.3_ZFHIGH_WT_DS5_AACCCTCTCGAT	5.915213

```
...                                        ...
hpf24_DEW057_TGACACAACAG_GCCACATC  5.429996
hpf24_DEW057_CTTACGGG_AACCTGAC     5.612010
hpf24_DEW057_TGAACATCTAT_GACGATGG  5.374328
hpf24_DEW057_TGAGGTTTCTC_CTCAGAAT  5.260610
hpf24_DEW057_ACGTGCTAG_CAAGTCAT    5.644344

[71203 rows x 1 columns]
```

1.10 Step 5 - downstream analysis

Once the gene age data has been added to the scRNA dataset, one can e.g. plot the corresponding transcriptome evolutionary index (`TEI`) values by any given observation pre-defined in the scRNA dataset.

Here, we plot them against the assigned embryo stage and against assigned cell types of the zebrafish using the `scanpy` `sc.pl.violin()` function as follows:

1.10.1 Boxplot gene age class per embryo stage

corresponds to main figure Figure 1D

[25]:
```
plt.rcParams['figure.figsize'] = [6.5, 4.5]
ax = sc.pl.violin(adata=zebrafish_data,
                  keys=['tei'],
                  groupby='stage',
                  rotation=90,
                  palette='Paired',
                  stripplot=False,
                  inner='box',
                  show=False)
ax.set_title('D. rerio - TEI distribution per embryo stage')
plt.show()
plt.rcParams['figure.figsize'] = [4.4, 3.3]
```

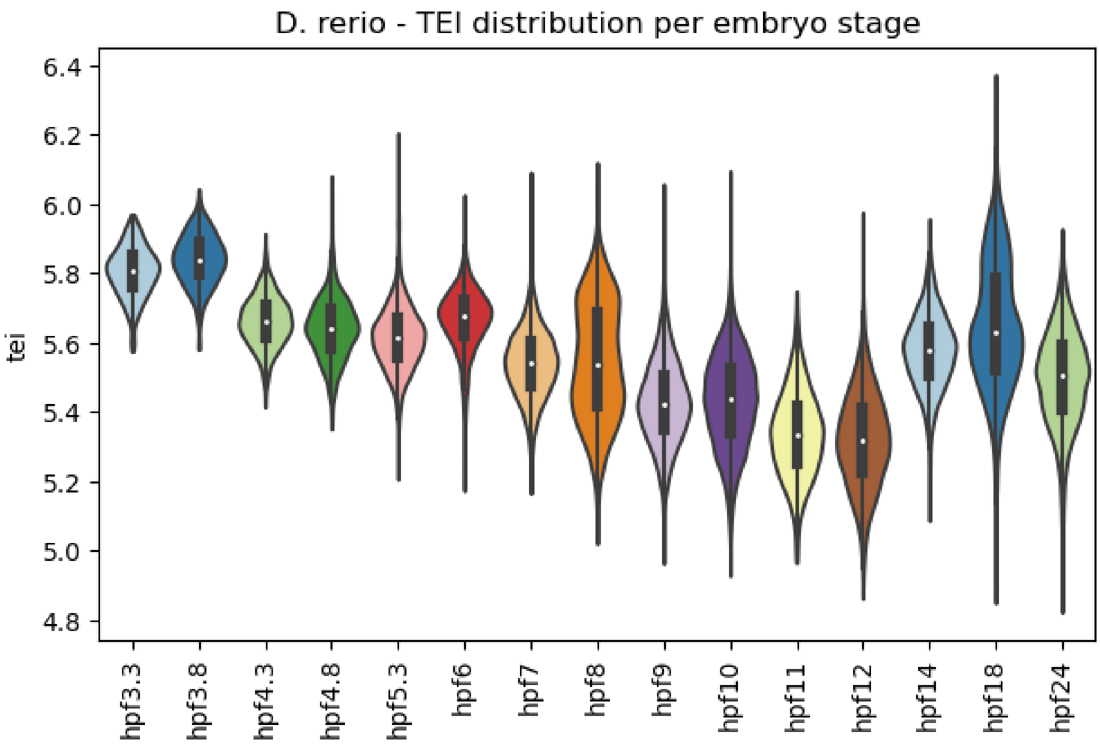

1.10.2 Boxplot gene age class per embryo stage and add significance

Note: Please change notebook cell from `raw` to `code` to see the plots.

```
ax = sns.boxplot(
    x='stage',
    y='tei',
    data=zebrafish_data.obs)
ax.set_title('D. rerio - TEI distribution per embryo stage')
test_results = add_stat_annotation(
    ax,
    x='stage',
    y='tei',
    data=zebrafish_data.obs,
    box_pairs=orthomap2tei._get_pairwise_comb_self(
        list1=zebrafish_data.obs['stage'].value_counts().index),
        test='Mann-Whitney',
        text_format='star',
        loc='outside',
    verbose=0)
plt.xticks(rotation=90)
plt.show()
```

1.10.3 Boxplot gene age class per embryo stage and per cell type

E.g. to just show the same plot for a selected cell-type, one could do the following.

1. List all annotated cell types:

[26]:
```
list(set(zebrafish_data.obs['cell_type']))
```

[26]:
```
['DEL',
 'heart',
 'epidermal',
 'diencephalon',
 'EVL',
 'neural anterior',
 'telencephalon',
 'hindbrain',
 'endothelial',
 'mesoderm lateral plate',
 'dorsal margin involuted',
 'differentiating neurons',
 'midbrain ventral',
 'neural plate anterior',
 'epidermal (gbx2+)',
 'germline',
 'hatching gland',
 'tailbud spinal cord',
 'lens placode',
 'roofplate',
 'tailbud mesoderm',
 'neural crest',
 'blood island',
 'differentiating neurons (eomesa+)',
 'differentiating neurons (rohon beard)',
 'optic primordium',
 'floorplate',
 'epidermal (foxi3a+)',
 'neural plate posterior',
 'pronephric duct',
 'retina pigmented epithelium',
 'macrophage',
 'hindbrain dorsal',
 'epiblast',
 'optic cup',
 'forerunner cells',
 'mesoderm lateral plate (fli1a+)',
 'erythroid',
 'apoptotic like',
 'lateral line primordium',
 'ionocyte',
 'midbrain',
 'mesoderm adaxial cells',
 'mesoderm lateral plate (tbx1+)',
 'xanthoblast',
 'margin',
 'notochord',
 'pectoral fin field',
 'otic placode',
 'differentiating neurons (dlx1a+)',
 'diencephalon (aplnr2+)',
```

```
'melanoblast',
'non dorsal margin involuted',
'gut',
'myotome',
'pharyngeal arch',
'periderm',
'prechordal plate',
'hindbrain ventral',
'olfactory placode',
'endoderm',
'blastomere',
'anterior neural ridge']
```

2. Loop over all cell types:

Note: Please change notebook cell from `raw` to `code` to see the plots.

[27]:
```
for ct in list(set(zebrafish_data.obs['cell_type'])):
    ax = sc.pl.violin(adata=zebrafish_data[zebrafish_data.obs['cell_type']==ct],
                      keys=['tei'],
                      groupby='stage',
                      rotation=90,
                      palette='Paired',
                      stripplot=False,
                      inner='box',
                      order=list(zebrafish_data.obs['stage'].cat.categories),
                      show=False)
    ax.set_title(ct)
    ax.set_xlabel('stage')
    plt.show()
```

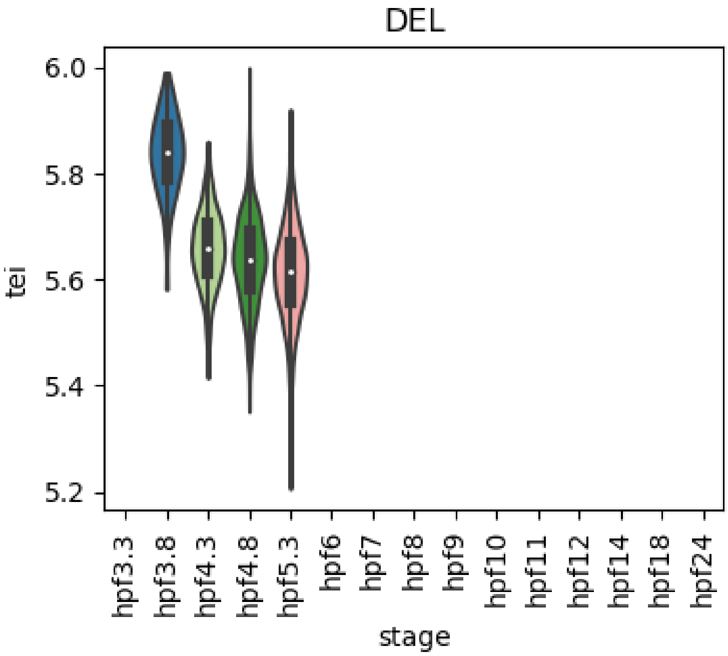

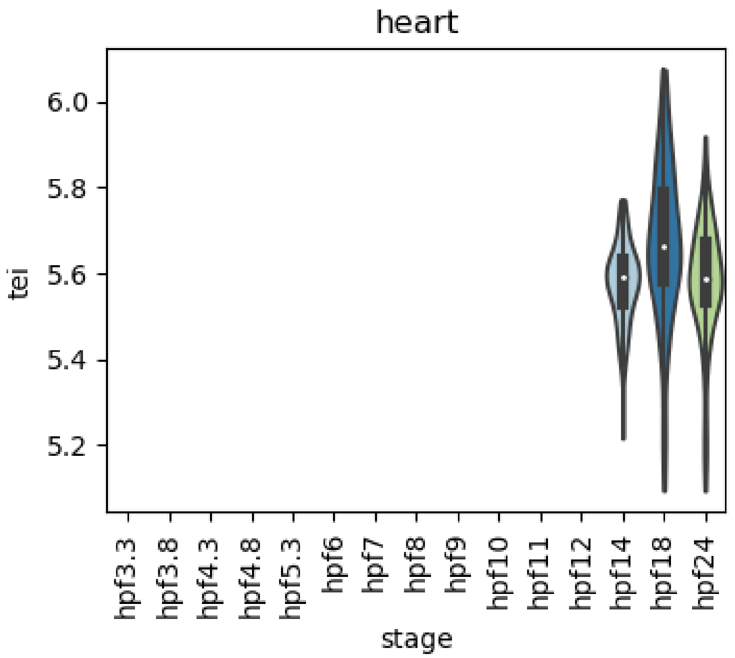
heart
tei
6.0
5.8
5.6
5.4
5.2
hpf3.3
hpf3.8
hpf4.3
hpf4.8
hpf5.3
hpf6
hpf7
hpf8
hpf9
hpf10
hpf11
hpf12
hpf14
hpf18
hpf24
stage

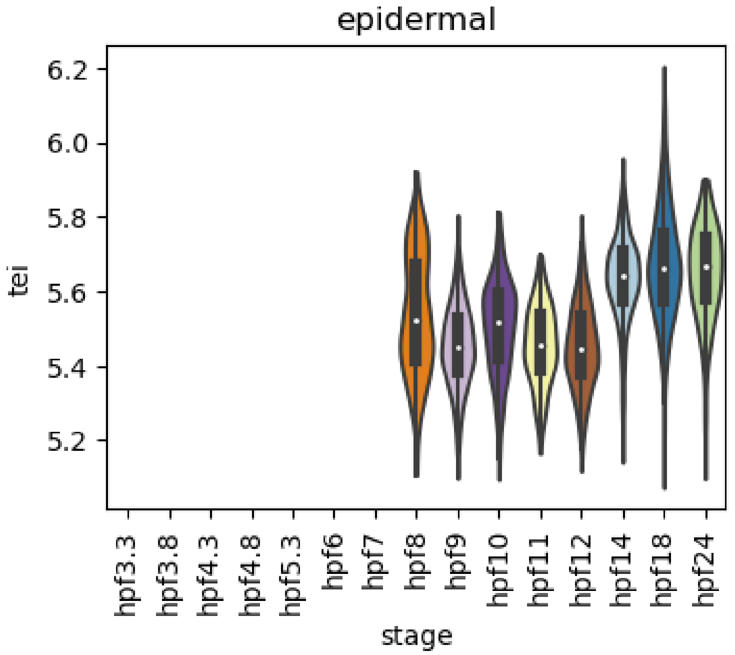
epidermal
tei
6.2
6.0
5.8
5.6
5.4
5.2
hpf3.3
hpf3.8
hpf4.3
hpf4.8
hpf5.3
hpf6
hpf7
hpf8
hpf9
hpf10
hpf11
hpf12
hpf14
hpf18
hpf24
stage

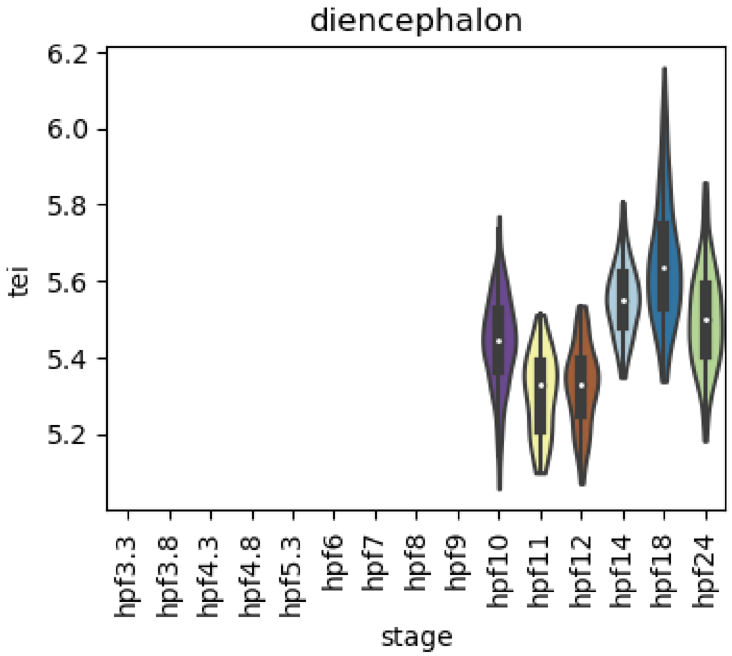
diencephalon
tei
6.2
6.0
5.8
5.6
5.4
5.2
hpf3.3
hpf3.8
hpf4.3
hpf4.8
hpf5.3
hpf6
hpf7
hpf8
hpf9
hpf10
hpf11
hpf12
hpf14
hpf18
hpf24
stage

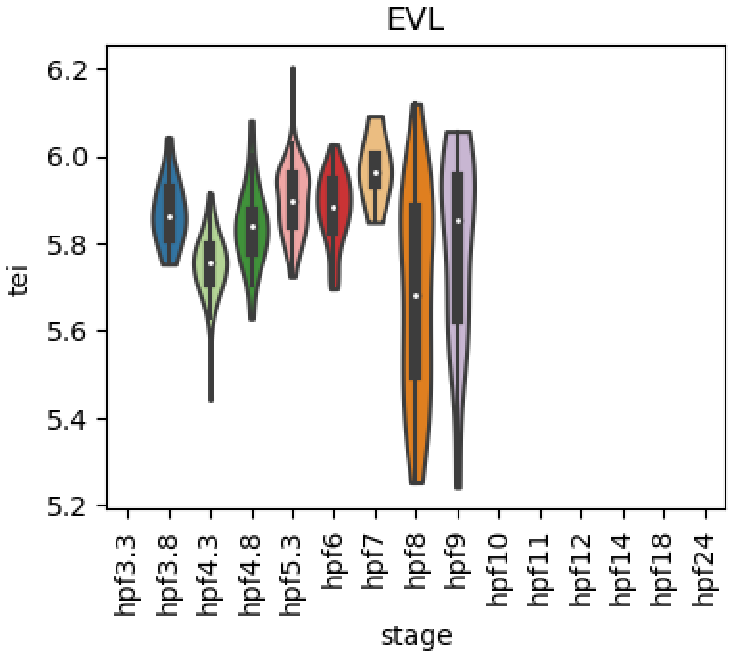
EVL
tei
6.2
6.0
5.8
5.6
5.4
5.2
hpf3.3
hpf3.8
hpf4.3
hpf4.8
hpf5.3
hpf6
hpf7
hpf8
hpf9
hpf10
hpf11
hpf12
hpf14
hpf18
hpf24
stage

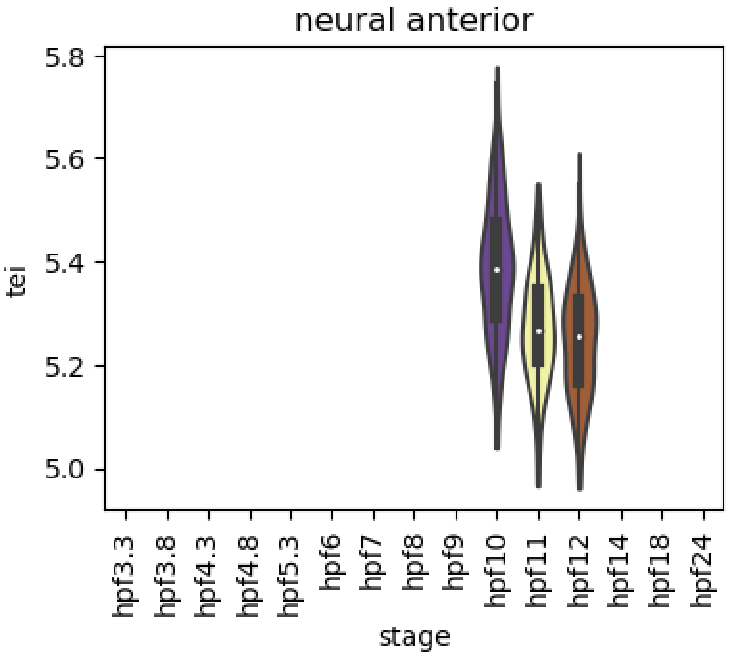

neural anterior
tei
5.8
5.6
5.4
5.2
5.0
hpf3.3
hpf3.8
hpf4.3
hpf4.8
hpf5.3
hpf6
hpf7
hpf8
hpf9
hpf10
hpf11
hpf12
hpf14
hpf18
hpf24
stage

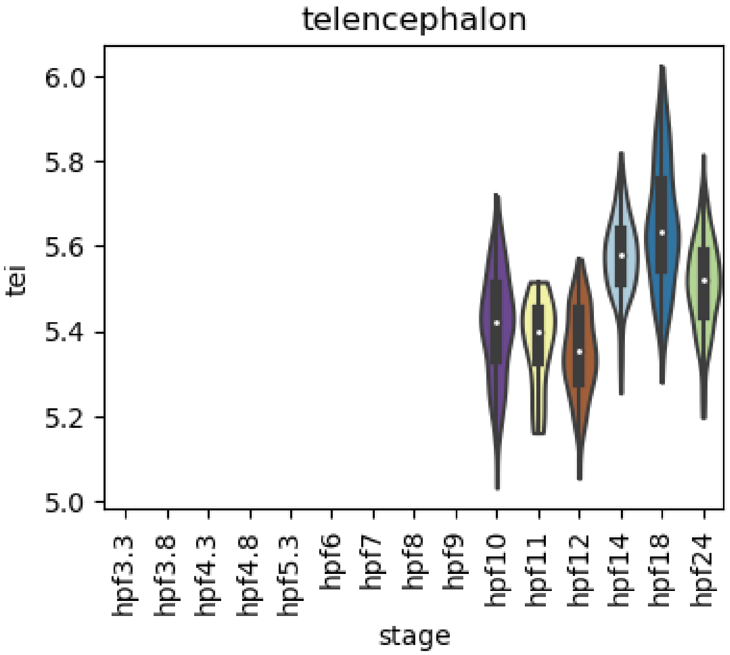

telencephalon
tei
6.0
5.8
5.6
5.4
5.2
5.0
hpf3.3
hpf3.8
hpf4.3
hpf4.8
hpf5.3
hpf6
hpf7
hpf8
hpf9
hpf10
hpf11
hpf12
hpf14
hpf18
hpf24
stage

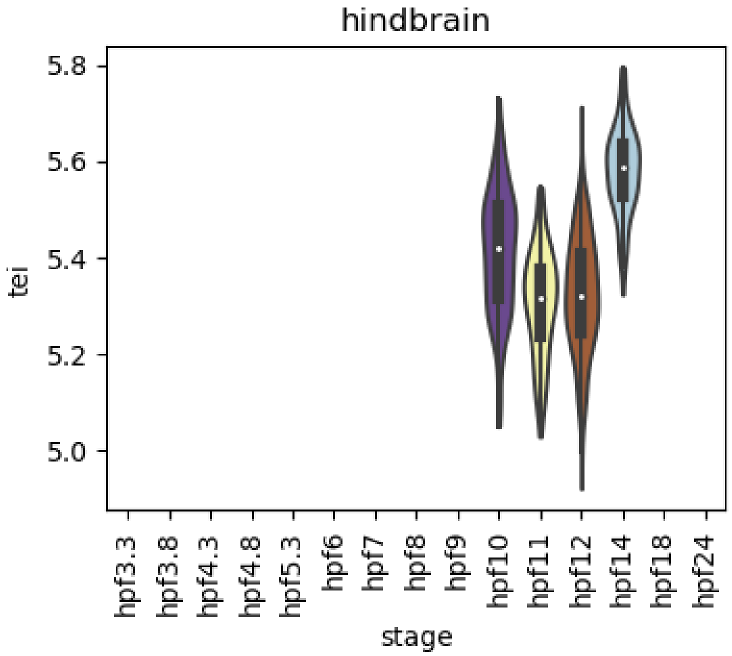
hindbrain
5.8
5.6
5.4
5.2
5.0
tei
hpf3.3
hpf3.8
hpf4.3
hpf4.8
hpf5.3
hpf6
hpf7
hpf8
hpf9
hpf10
hpf11
hpf12
hpf14
hpf18
hpf24
stage

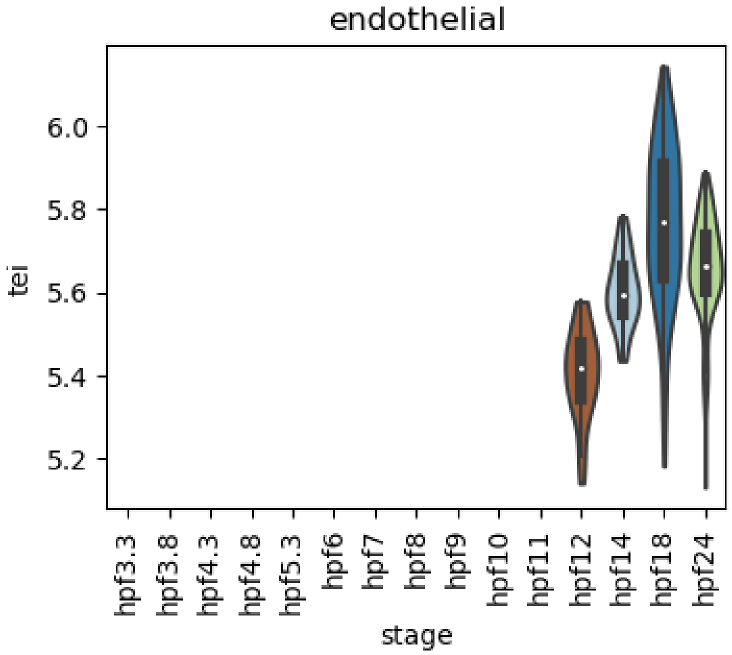
endothelial
6.0
5.8
5.6
5.4
5.2
tei
hpf3.3
hpf3.8
hpf4.3
hpf4.8
hpf5.3
hpf6
hpf7
hpf8
hpf9
hpf10
hpf11
hpf12
hpf14
hpf18
hpf24
stage

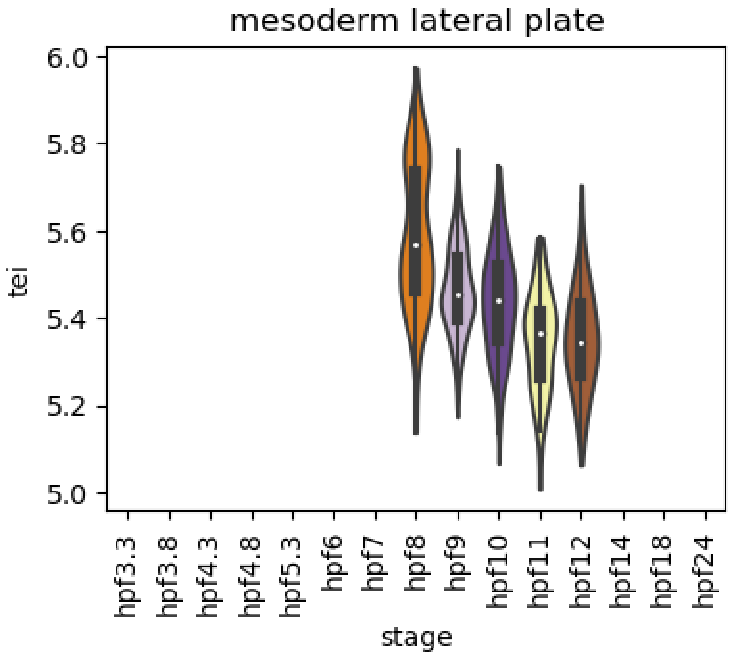
mesoderm lateral plate
6.0
5.8
5.6
5.4
5.2
5.0
tei
hpf3.3
hpf3.8
hpf4.3
hpf4.8
hpf5.3
hpf6
hpf7
hpf8
hpf9
hpf10
hpf11
hpf12
hpf14
hpf18
hpf24
stage

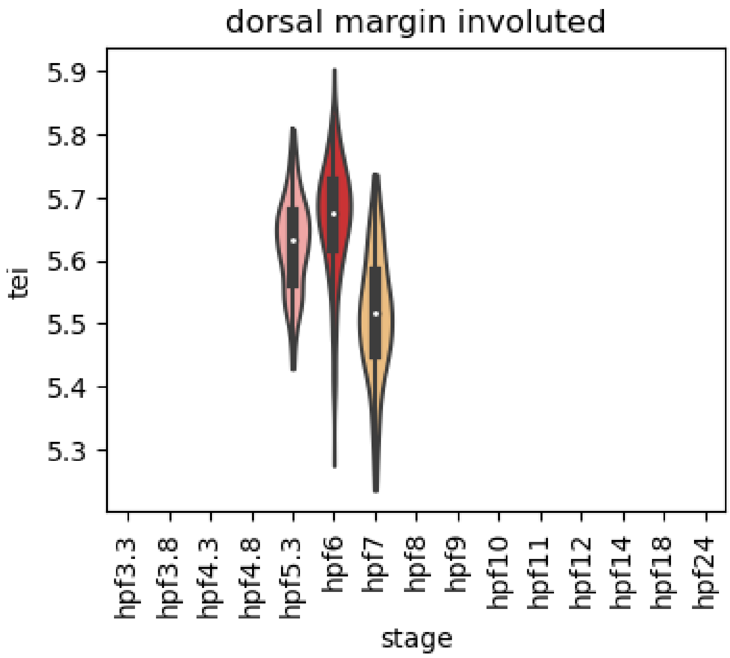
dorsal margin involuted
5.9
5.8
5.7
5.6
5.5
5.4
5.3
tei
hpf3.3
hpf3.8
hpf4.3
hpf4.8
hpf5.3
hpf6
hpf7
hpf8
hpf9
hpf10
hpf11
hpf12
hpf14
hpf18
hpf24
stage

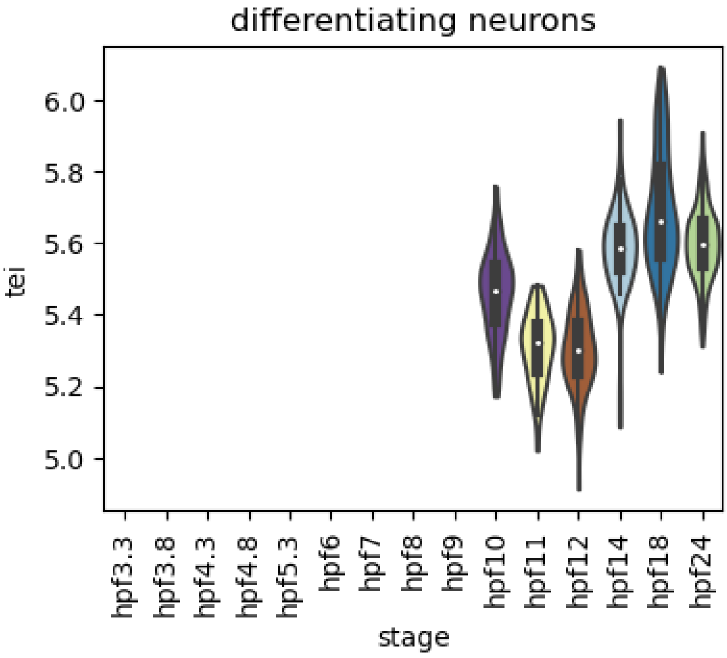
differentiating neurons
6.0
5.8
5.6
5.4
5.2
5.0
tei
hpf3.3
hpf3.8
hpf4.3
hpf4.8
hpf5.3
hpf6
hpf7
hpf8
hpf9
hpf10
hpf11
hpf12
hpf14
hpf18
hpf24
stage

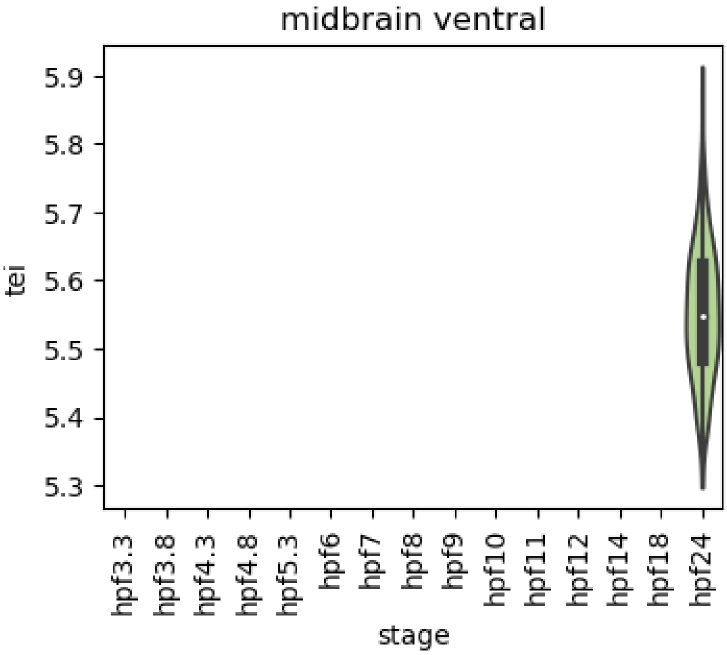
midbrain ventral
5.9
5.8
5.7
5.6
5.5
5.4
5.3
tei
hpf3.3
hpf3.8
hpf4.3
hpf4.8
hpf5.3
hpf6
hpf7
hpf8
hpf9
hpf10
hpf11
hpf12
hpf14
hpf18
hpf24
stage

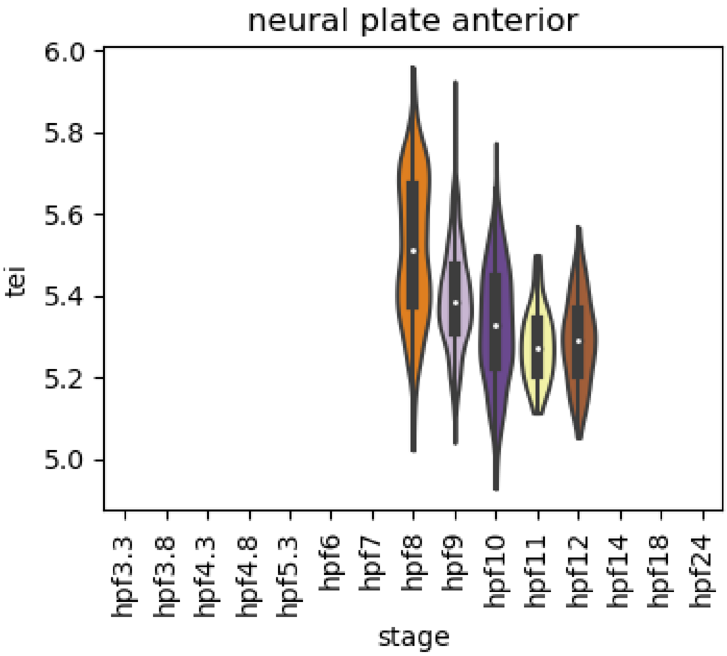
neural plate anterior
6.0
5.8
5.6
5.4
5.2
5.0
tei
hpf3.3
hpf3.8
hpf4.3
hpf4.8
hpf5.3
hpf6
hpf7
hpf8
hpf9
hpf10
hpf11
hpf12
hpf14
hpf18
hpf24
stage

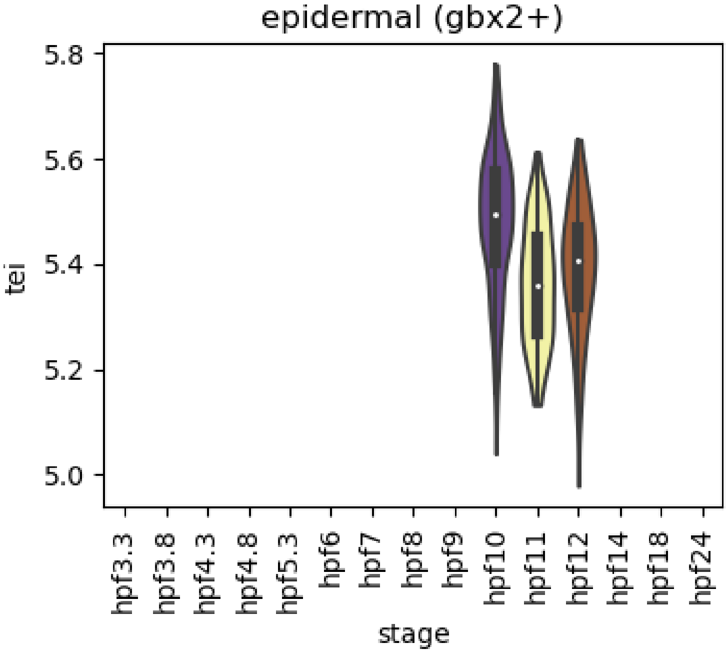
epidermal (gbx2+)
5.8
5.6
5.4
5.2
5.0
tei
hpf3.3
hpf3.8
hpf4.3
hpf4.8
hpf5.3
hpf6
hpf7
hpf8
hpf9
hpf10
hpf11
hpf12
hpf14
hpf18
hpf24
stage

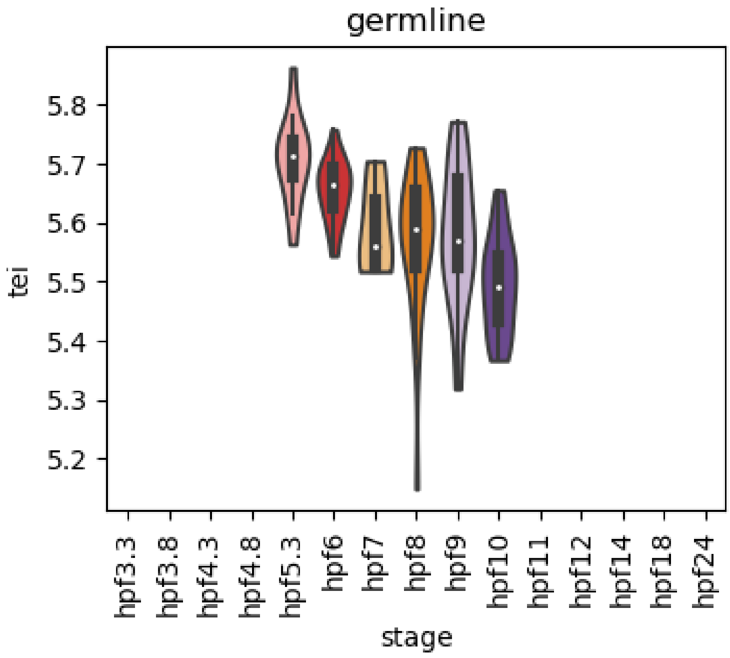
germline
5.8
5.7
5.6
5.5
5.4
5.3
5.2
tei
hpf3.3
hpf3.8
hpf4.3
hpf4.8
hpf5.3
hpf6
hpf7
hpf8
hpf9
hpf10
hpf11
hpf12
hpf14
hpf18
hpf24
stage

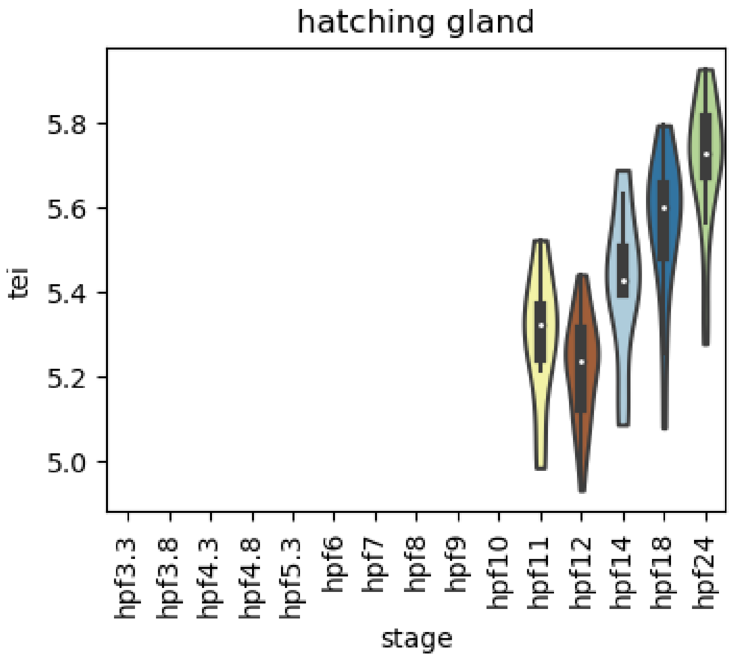
hatching gland
5.8
5.6
5.4
5.2
5.0
tei
hpf3.3
hpf3.8
hpf4.3
hpf4.8
hpf5.3
hpf6
hpf7
hpf8
hpf9
hpf10
hpf11
hpf12
hpf14
hpf18
hpf24
stage

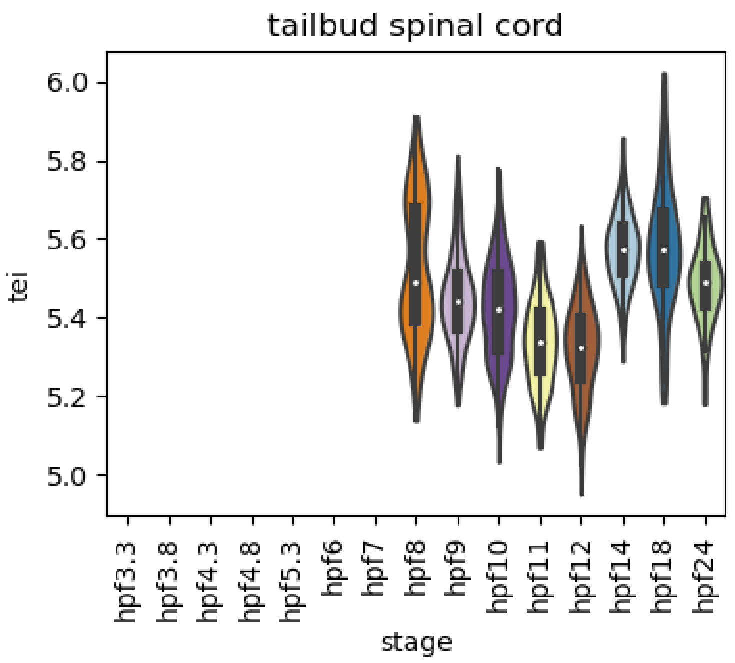
tailbud spinal cord
tei
6.0
5.8
5.6
5.4
5.2
5.0
hpf3.3
hpf3.8
hpf4.3
hpf4.8
hpf5.3
hpf6
hpf7
hpf8
hpf9
hpf10
hpf11
hpf12
hpf14
hpf18
hpf24
stage

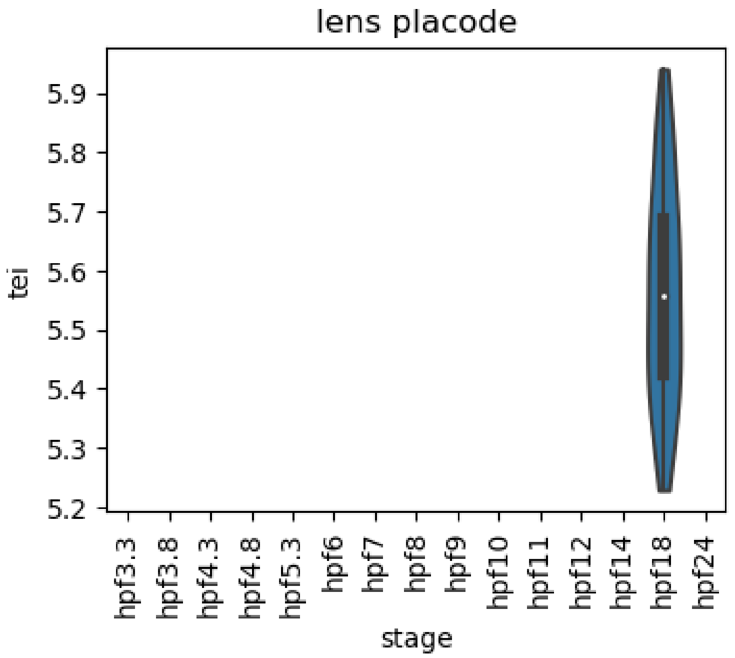
lens placode
tei
5.9
5.8
5.7
5.6
5.5
5.4
5.3
5.2
hpf3.3
hpf3.8
hpf4.3
hpf4.8
hpf5.3
hpf6
hpf7
hpf8
hpf9
hpf10
hpf11
hpf12
hpf14
hpf18
hpf24
stage

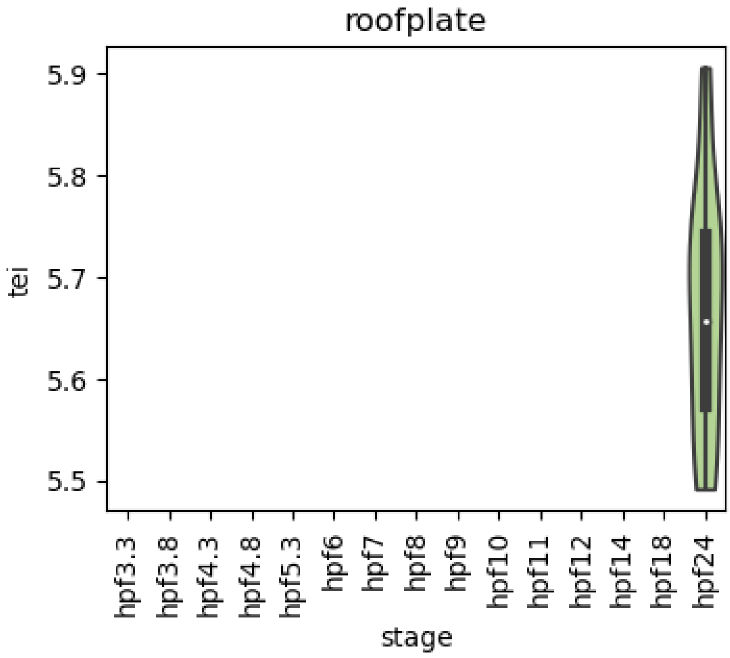
roofplate
tei
5.9
5.8
5.7
5.6
5.5
stage
hpf3.3
hpf3.8
hpf4.3
hpf4.8
hpf5.3
hpf6
hpf7
hpf8
hpf9
hpf10
hpf11
hpf12
hpf14
hpf18
hpf24

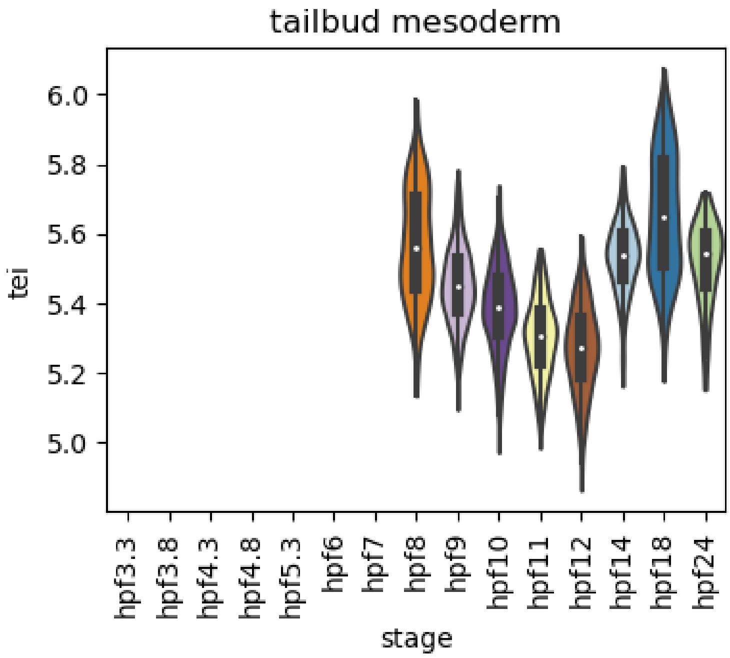
tailbud mesoderm
tei
6.0
5.8
5.6
5.4
5.2
5.0
stage
hpf3.3
hpf3.8
hpf4.3
hpf4.8
hpf5.3
hpf6
hpf7
hpf8
hpf9
hpf10
hpf11
hpf12
hpf14
hpf18
hpf24

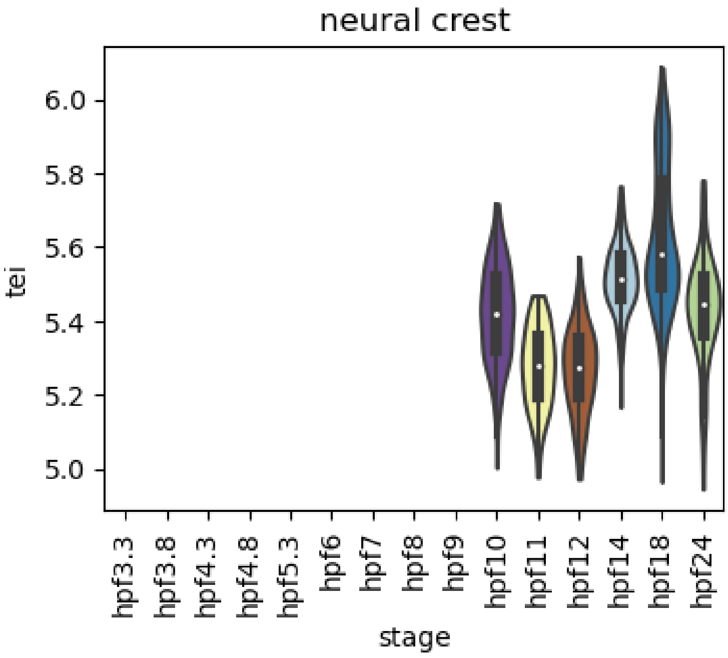
neural crest
tei
6.0
5.8
5.6
5.4
5.2
5.0
hpf3.3
hpf3.8
hpf4.3
hpf4.8
hpf5.3
hpf6
hpf7
hpf8
hpf9
hpf10
hpf11
hpf12
hpf14
hpf18
hpf24
stage

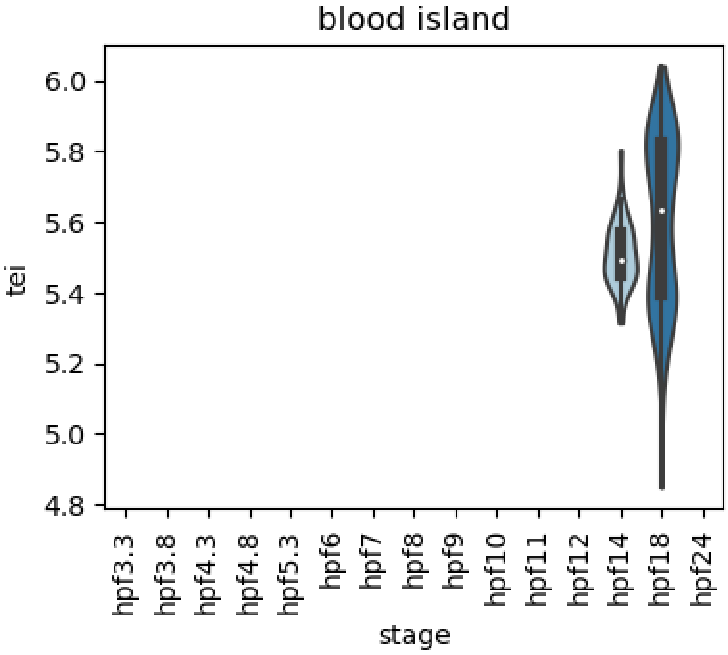
blood island
tei
6.0
5.8
5.6
5.4
5.2
5.0
4.8
hpf3.3
hpf3.8
hpf4.3
hpf4.8
hpf5.3
hpf6
hpf7
hpf8
hpf9
hpf10
hpf11
hpf12
hpf14
hpf18
hpf24
stage

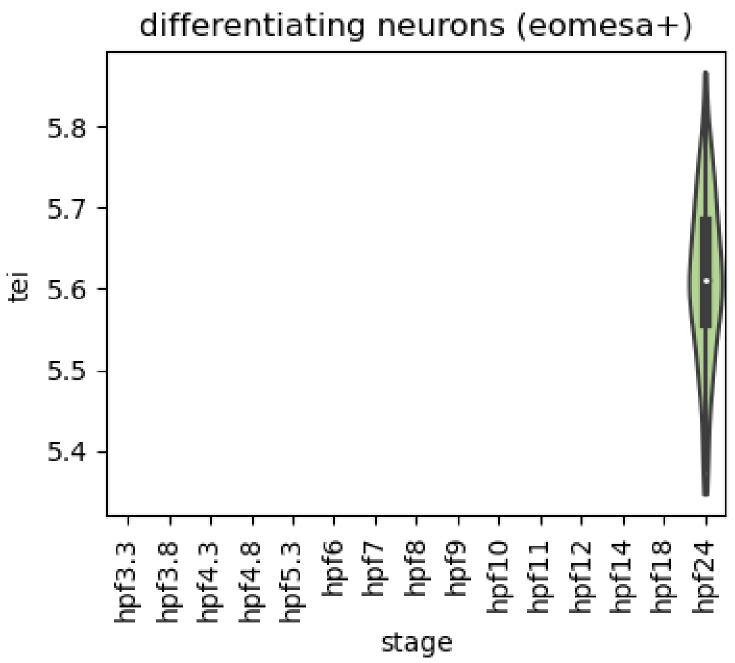
differentiating neurons (eomesa+)
5.8
5.7
5.6
5.5
5.4
tei
hpf3.3
hpf3.8
hpf4.3
hpf4.8
hpf5.3
hpf6
hpf7
hpf8
hpf9
hpf10
hpf11
hpf12
hpf14
hpf18
hpf24
stage

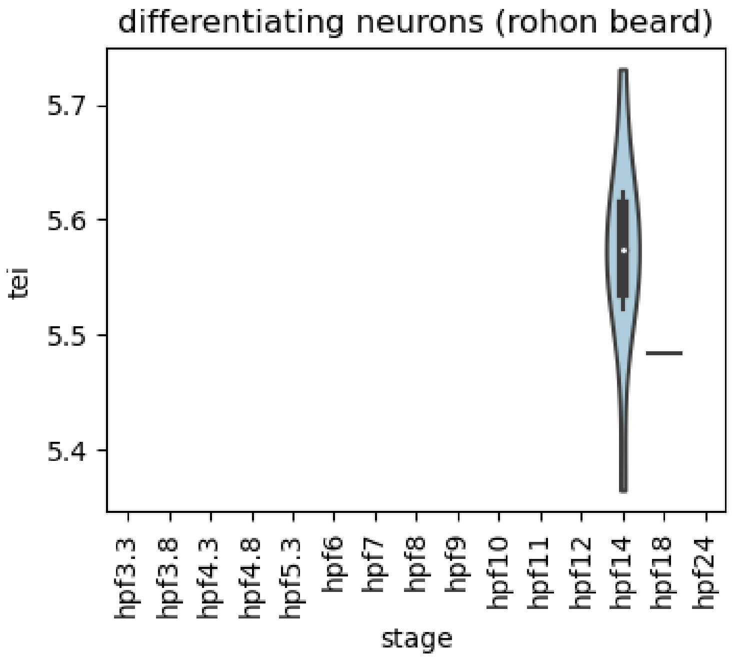
differentiating neurons (rohon beard)
5.7
5.6
5.5
5.4
tei
hpf3.3
hpf3.8
hpf4.3
hpf4.8
hpf5.3
hpf6
hpf7
hpf8
hpf9
hpf10
hpf11
hpf12
hpf14
hpf18
hpf24
stage

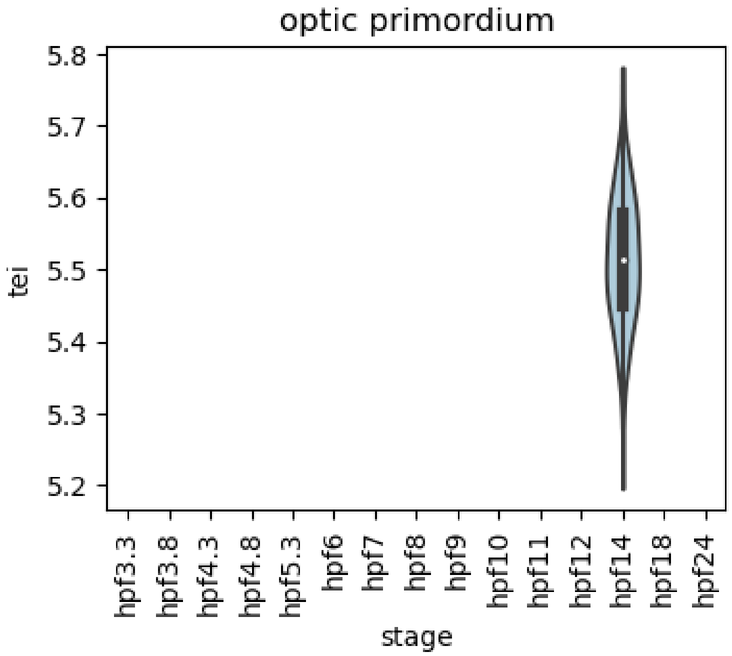
optic primordium
5.8
5.7
5.6
5.5
5.4
5.3
5.2
tei
hpf3.3
hpf3.8
hpf4.3
hpf4.8
hpf5.3
hpf6
hpf7
hpf8
hpf9
hpf10
hpf11
hpf12
hpf14
hpf18
hpf24
stage

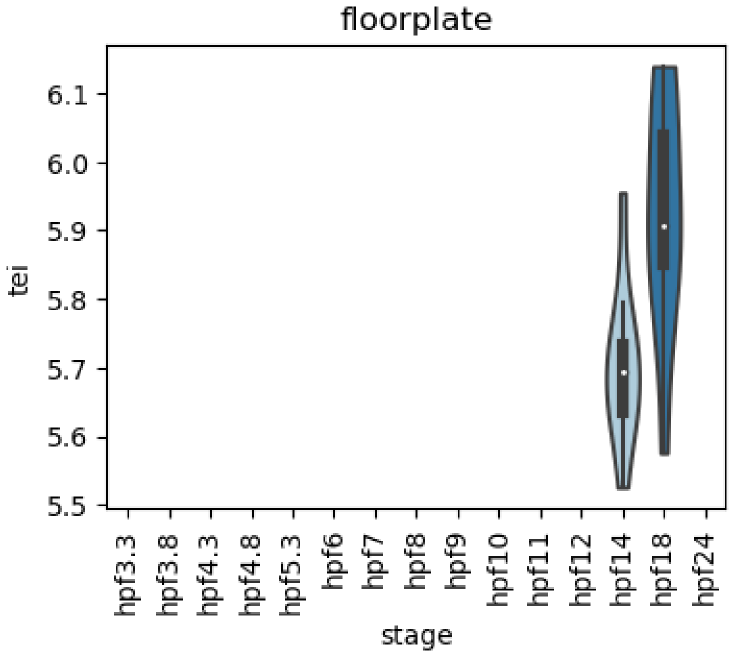
floorplate
6.1
6.0
5.9
5.8
5.7
5.6
5.5
tei
hpf3.3
hpf3.8
hpf4.3
hpf4.8
hpf5.3
hpf6
hpf7
hpf8
hpf9
hpf10
hpf11
hpf12
hpf14
hpf18
hpf24
stage

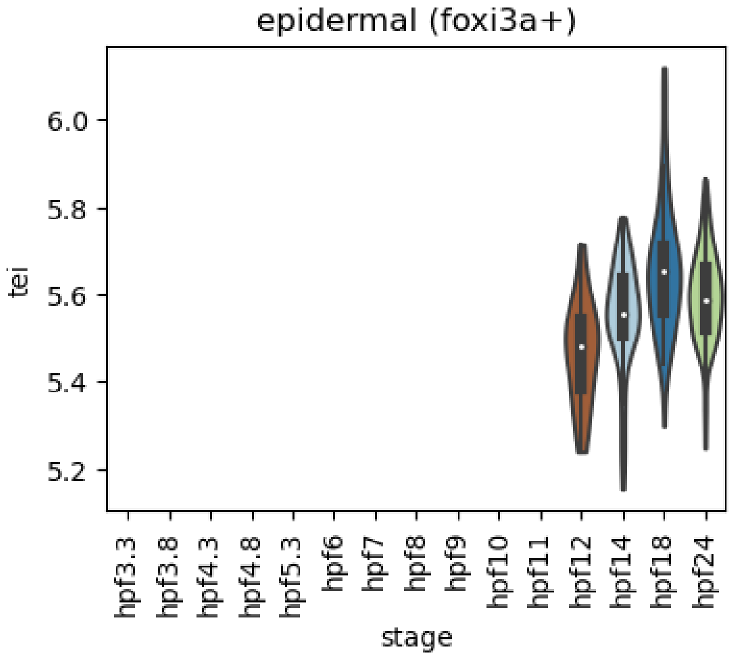
epidermal (foxi3a+)
6.0
5.8
5.6
5.4
5.2
tei
hpf3.3
hpf3.8
hpf4.3
hpf4.8
hpf5.3
hpf6
hpf7
hpf8
hpf9
hpf10
hpf11
hpf12
hpf14
hpf18
hpf24
stage

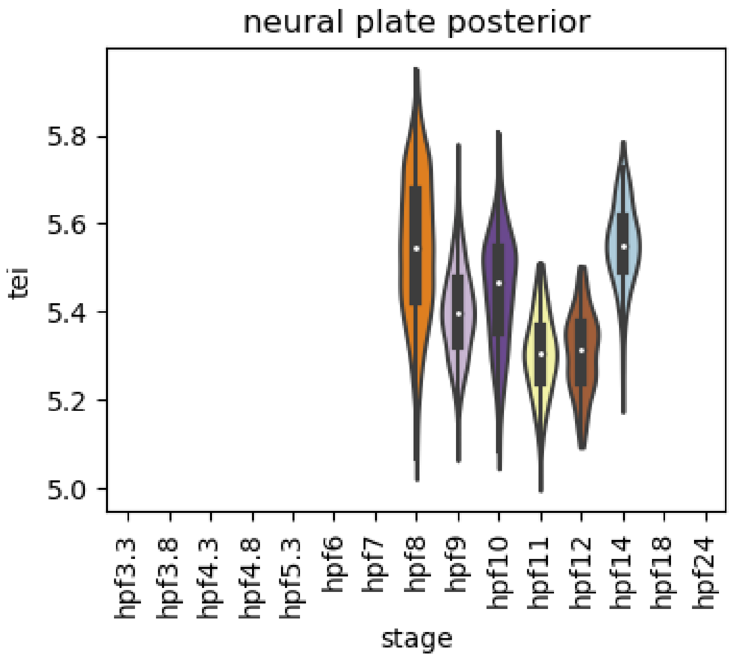
neural plate posterior
5.8
5.6
5.4
5.2
5.0
tei
hpf3.3
hpf3.8
hpf4.3
hpf4.8
hpf5.3
hpf6
hpf7
hpf8
hpf9
hpf10
hpf11
hpf12
hpf14
hpf18
hpf24
stage

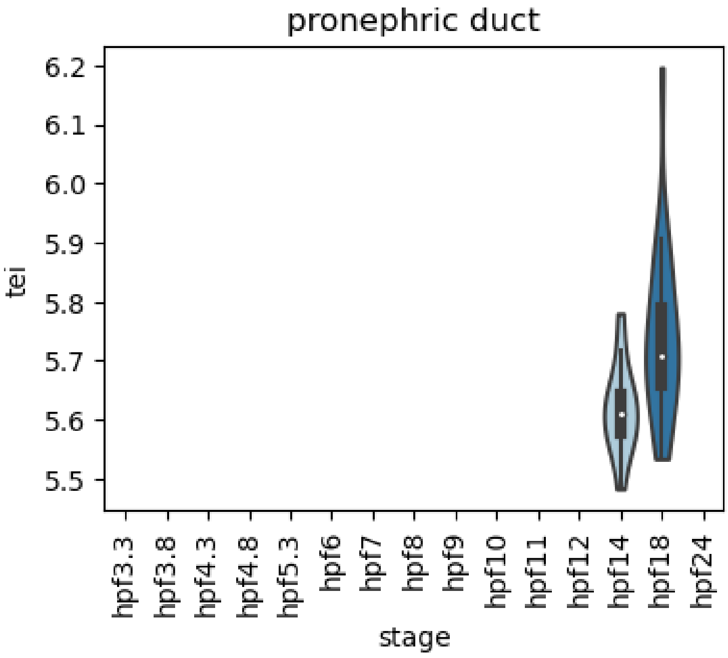
pronephric duct
tei
6.2
6.1
6.0
5.9
5.8
5.7
5.6
5.5
hpf3.3
hpf3.8
hpf4.3
hpf4.8
hpf5.3
hpf6
hpf7
hpf8
hpf9
hpf10
hpf11
hpf12
hpf14
hpf18
hpf24
stage

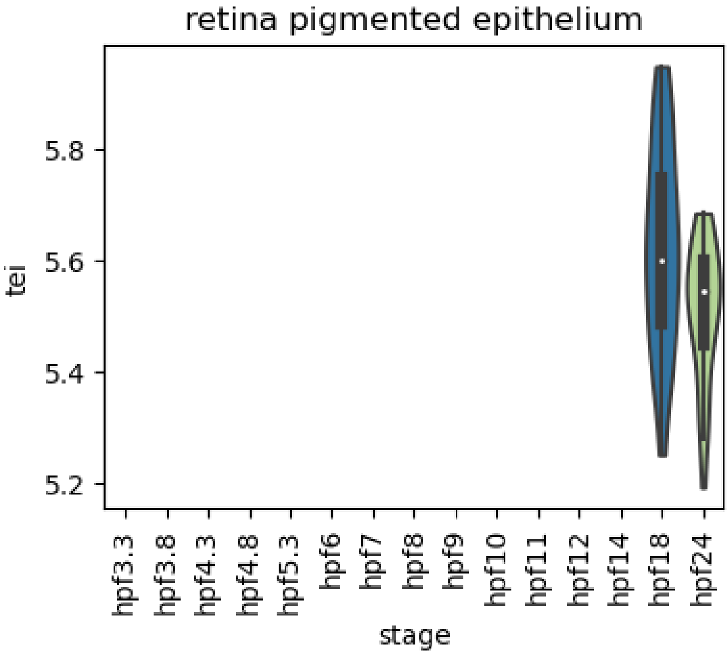
retina pigmented epithelium
tei
5.8
5.6
5.4
5.2
hpf3.3
hpf3.8
hpf4.3
hpf4.8
hpf5.3
hpf6
hpf7
hpf8
hpf9
hpf10
hpf11
hpf12
hpf14
hpf18
hpf24
stage

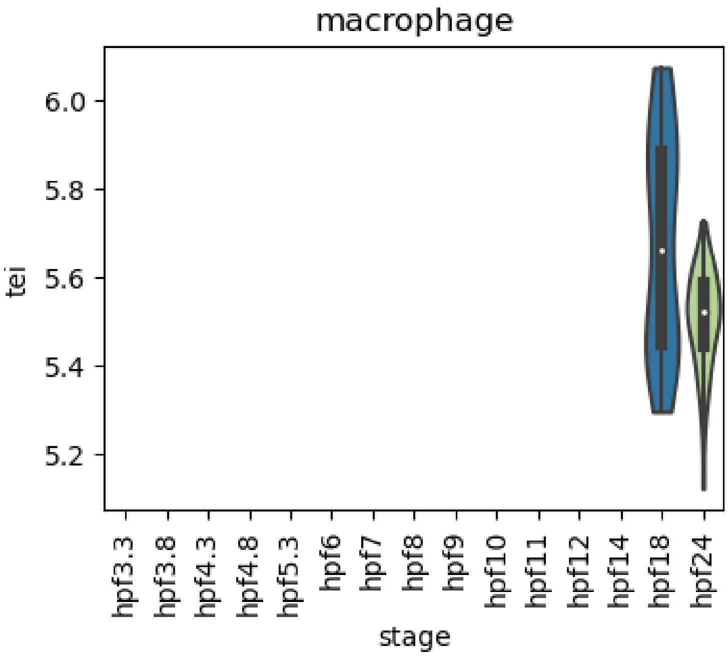
macrophage
6.0
5.8
5.6
5.4
5.2
tei
hpf3.3
hpf3.8
hpf4.3
hpf4.8
hpf5.3
hpf6
hpf7
hpf8
hpf9
hpf10
hpf11
hpf12
hpf14
hpf18
hpf24
stage

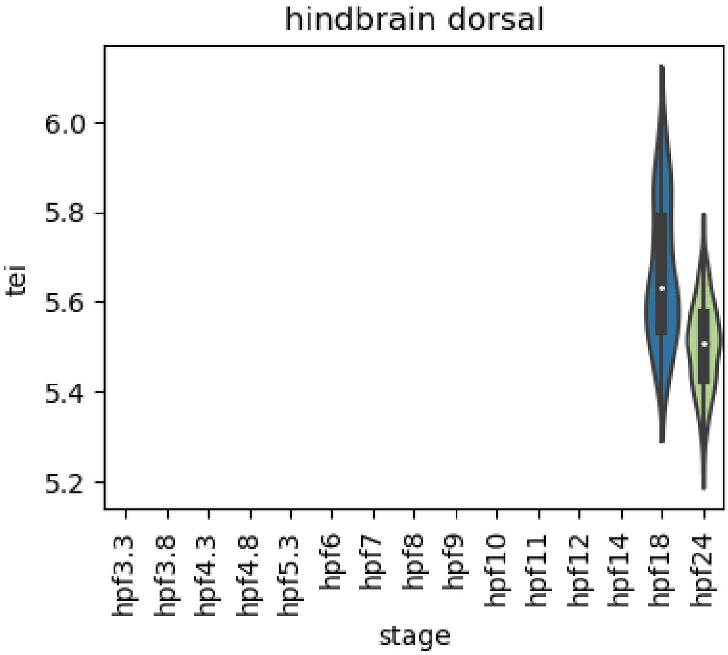
hindbrain dorsal
6.0
5.8
5.6
5.4
5.2
tei
hpf3.3
hpf3.8
hpf4.3
hpf4.8
hpf5.3
hpf6
hpf7
hpf8
hpf9
hpf10
hpf11
hpf12
hpf14
hpf18
hpf24
stage

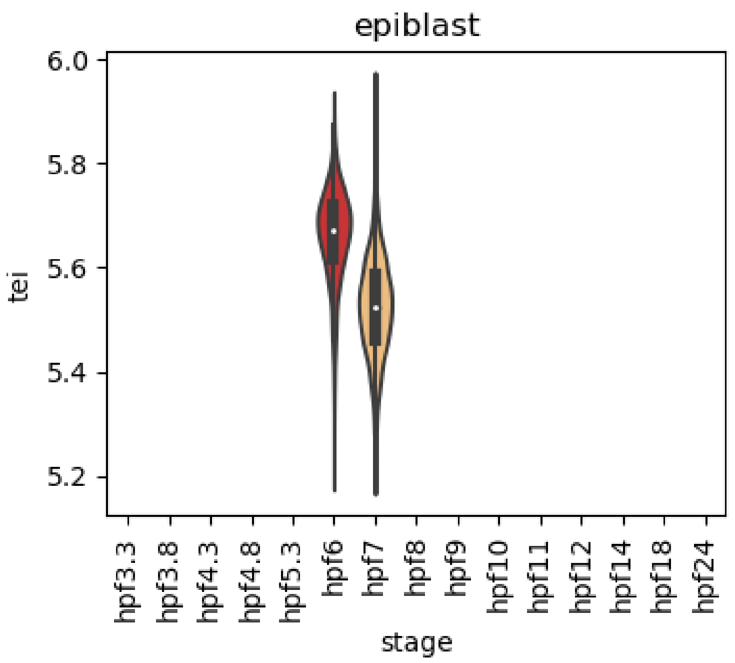
epiblast
6.0
5.8
5.6
5.4
5.2
tei
hpf3.3
hpf3.8
hpf4.3
hpf4.8
hpf5.3
hpf6
hpf7
hpf8
hpf9
hpf10
hpf11
hpf12
hpf14
hpf18
hpf24
stage

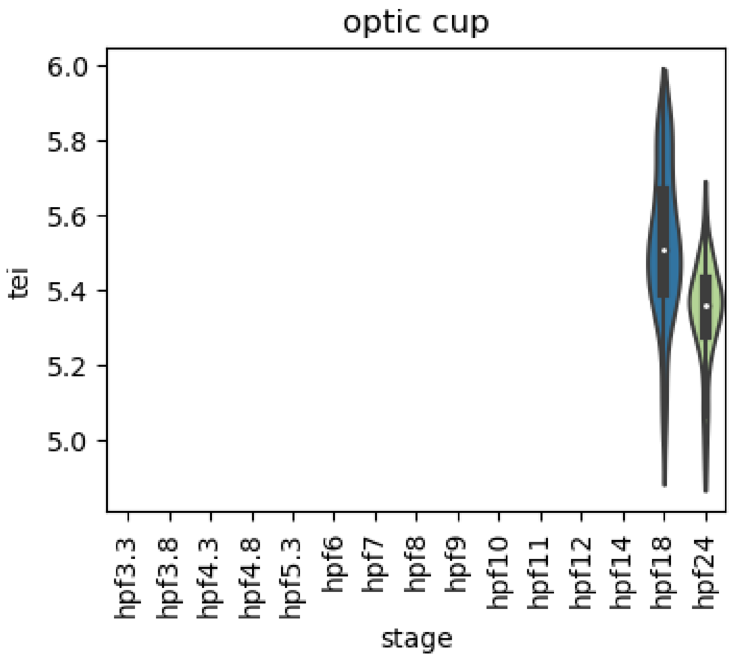
optic cup
6.0
5.8
5.6
5.4
5.2
5.0
tei
hpf3.3
hpf3.8
hpf4.3
hpf4.8
hpf5.3
hpf6
hpf7
hpf8
hpf9
hpf10
hpf11
hpf12
hpf14
hpf18
hpf24
stage

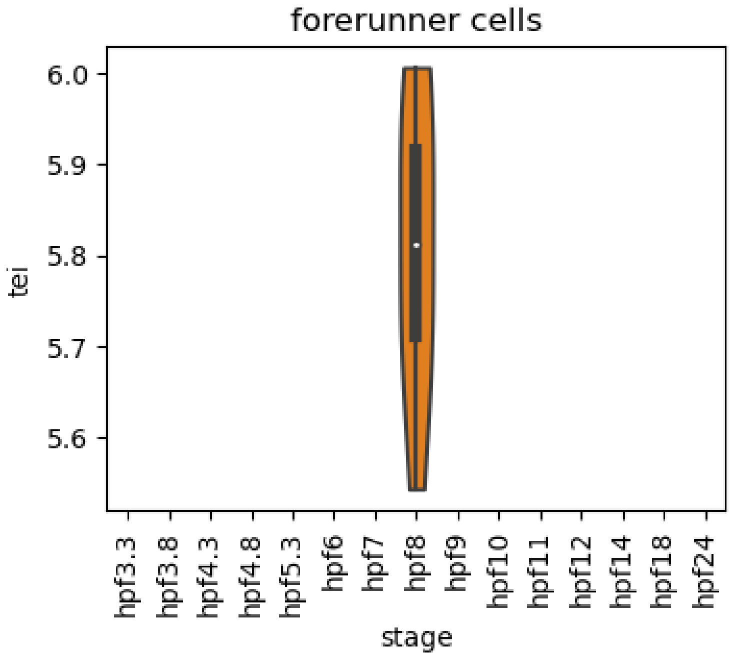
forerunner cells
6.0
5.9
5.8
5.7
5.6
tei
hpf3.3
hpf3.8
hpf4.3
hpf4.8
hpf5.3
hpf6
hpf7
hpf8
hpf9
hpf10
hpf11
hpf12
hpf14
hpf18
hpf24
stage

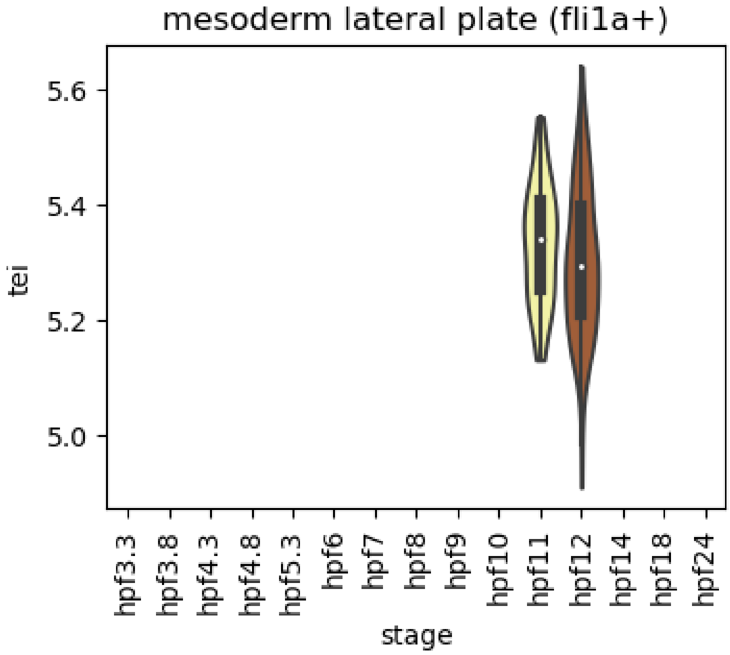
mesoderm lateral plate (fli1a+)
5.6
5.4
5.2
5.0
tei
hpf3.3
hpf3.8
hpf4.3
hpf4.8
hpf5.3
hpf6
hpf7
hpf8
hpf9
hpf10
hpf11
hpf12
hpf14
hpf18
hpf24
stage

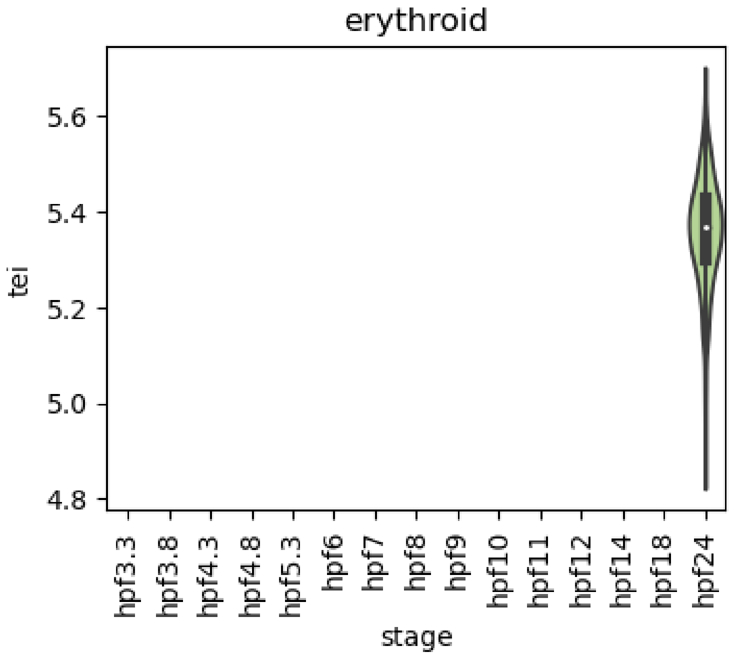

erythroid
5.6
5.4
5.2
5.0
4.8
tei
hpf3.3
hpf3.8
hpf4.3
hpf4.8
hpf5.3
hpf6
hpf7
hpf8
hpf9
hpf10
hpf11
hpf12
hpf14
hpf18
hpf24
stage

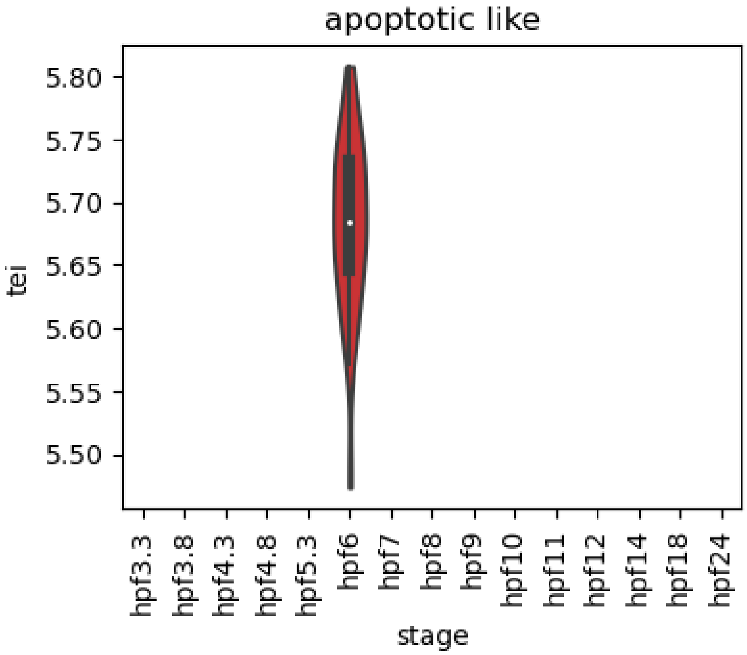

apoptotic like
5.80
5.75
5.70
5.65
5.60
5.55
5.50
tei
hpf3.3
hpf3.8
hpf4.3
hpf4.8
hpf5.3
hpf6
hpf7
hpf8
hpf9
hpf10
hpf11
hpf12
hpf14
hpf18
hpf24
stage

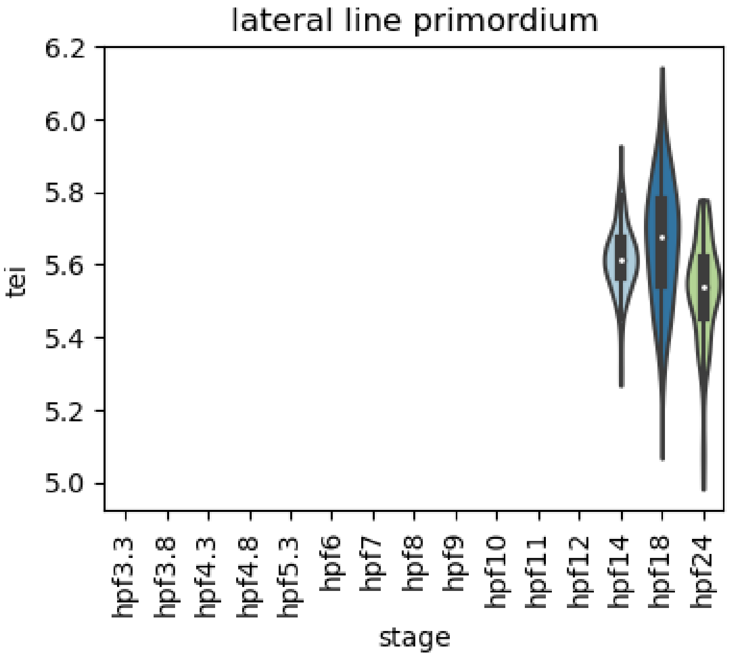
lateral line primordium
6.2
6.0
5.8
5.6
5.4
5.2
5.0
tei
hpf3.3
hpf3.8
hpf4.3
hpf4.8
hpf5.3
hpf6
hpf7
hpf8
hpf9
hpf10
hpf11
hpf12
hpf14
hpf18
hpf24
stage

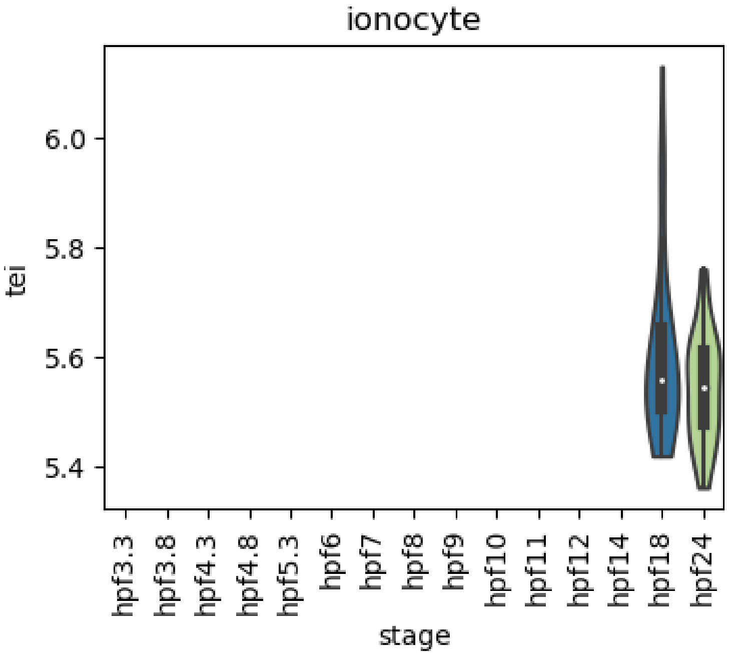
ionocyte
6.0
5.8
5.6
5.4
tei
hpf3.3
hpf3.8
hpf4.3
hpf4.8
hpf5.3
hpf6
hpf7
hpf8
hpf9
hpf10
hpf11
hpf12
hpf14
hpf18
hpf24
stage

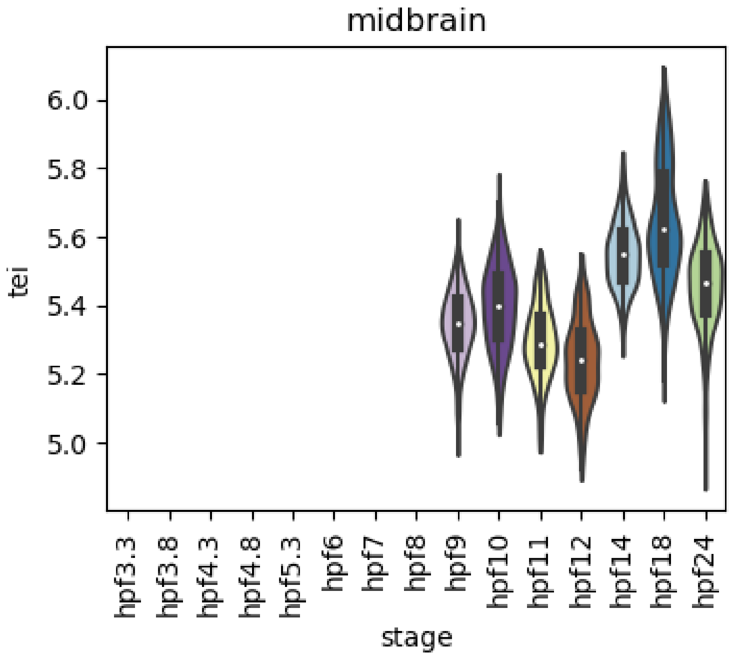
midbrain
tei
6.0
5.8
5.6
5.4
5.2
5.0
hpf3.3
hpf3.8
hpf4.3
hpf4.8
hpf5.3
hpf6
hpf7
hpf8
hpf9
hpf10
hpf11
hpf12
hpf14
hpf18
hpf24
stage

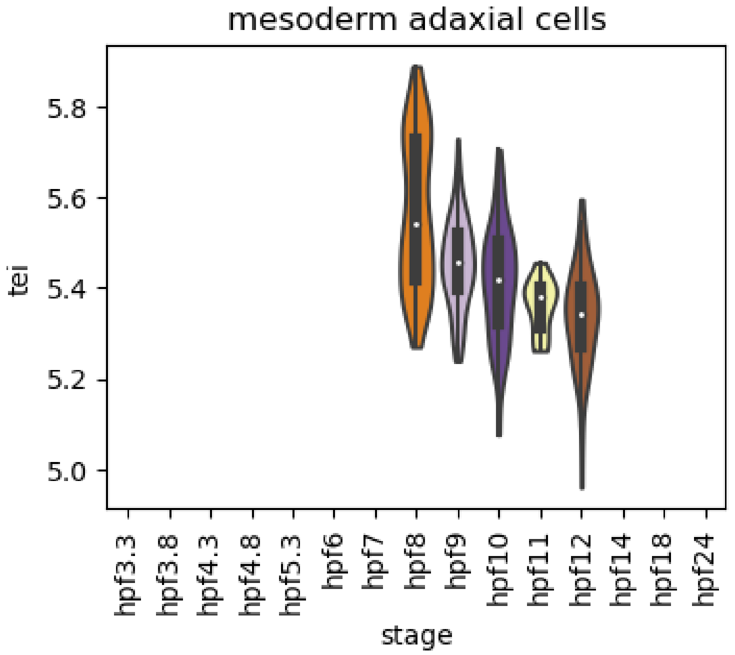
mesoderm adaxial cells
tei
5.8
5.6
5.4
5.2
5.0
hpf3.3
hpf3.8
hpf4.3
hpf4.8
hpf5.3
hpf6
hpf7
hpf8
hpf9
hpf10
hpf11
hpf12
hpf14
hpf18
hpf24
stage

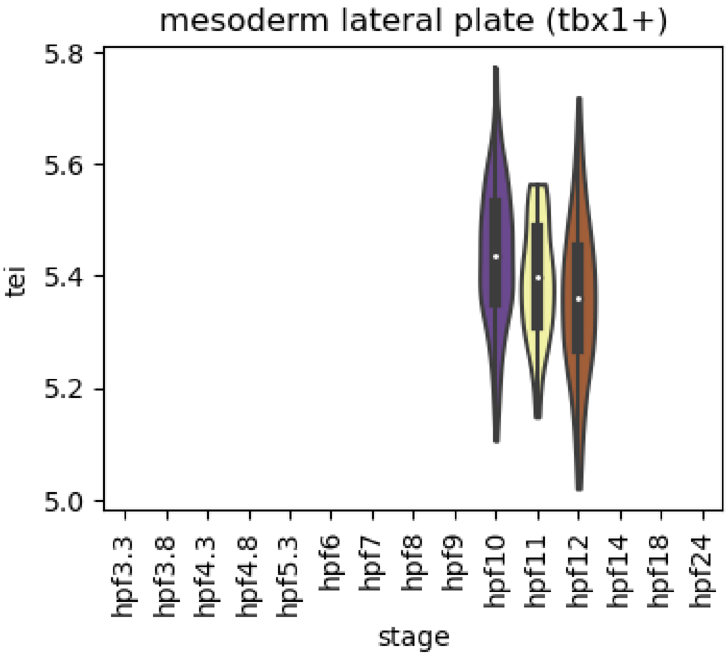
mesoderm lateral plate (tbx1+)
5.8
5.6
5.4
5.2
5.0
tei
hpf3.3
hpf3.8
hpf4.3
hpf4.8
hpf5.3
hpf6
hpf7
hpf8
hpf9
hpf10
hpf11
hpf12
hpf14
hpf18
hpf24
stage

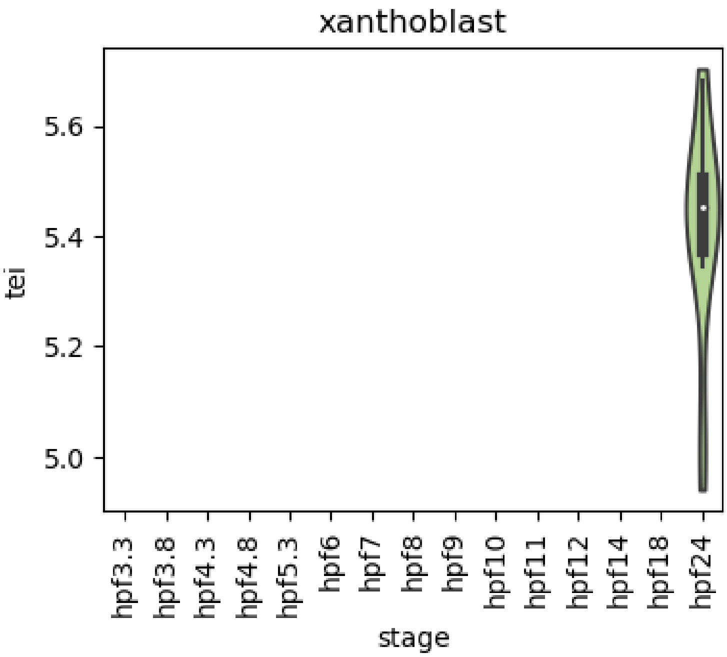
xanthoblast
5.6
5.4
5.2
5.0
tei
hpf3.3
hpf3.8
hpf4.3
hpf4.8
hpf5.3
hpf6
hpf7
hpf8
hpf9
hpf10
hpf11
hpf12
hpf14
hpf18
hpf24
stage

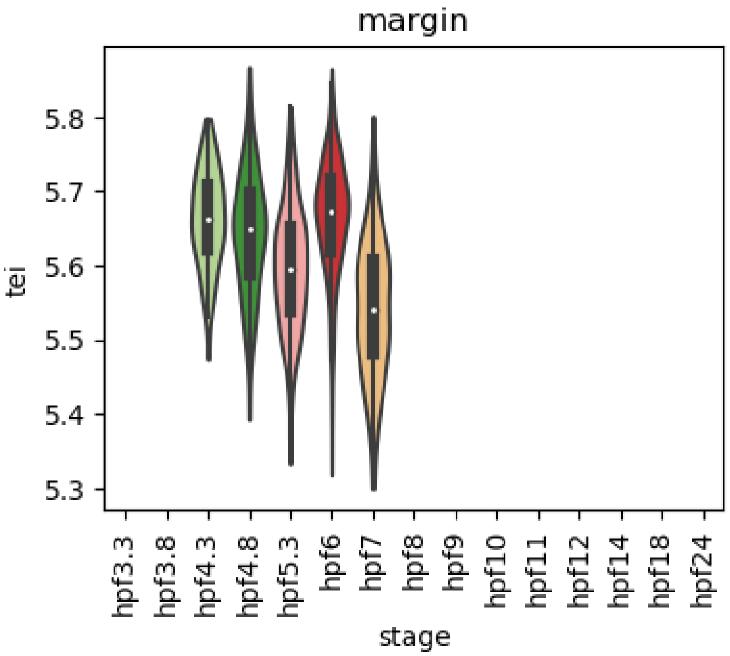
margin
5.8
5.7
5.6
5.5
5.4
5.3
tei
hpf3.3
hpf3.8
hpf4.3
hpf4.8
hpf5.3
hpf6
hpf7
hpf8
hpf9
hpf10
hpf11
hpf12
hpf14
hpf18
hpf24
stage

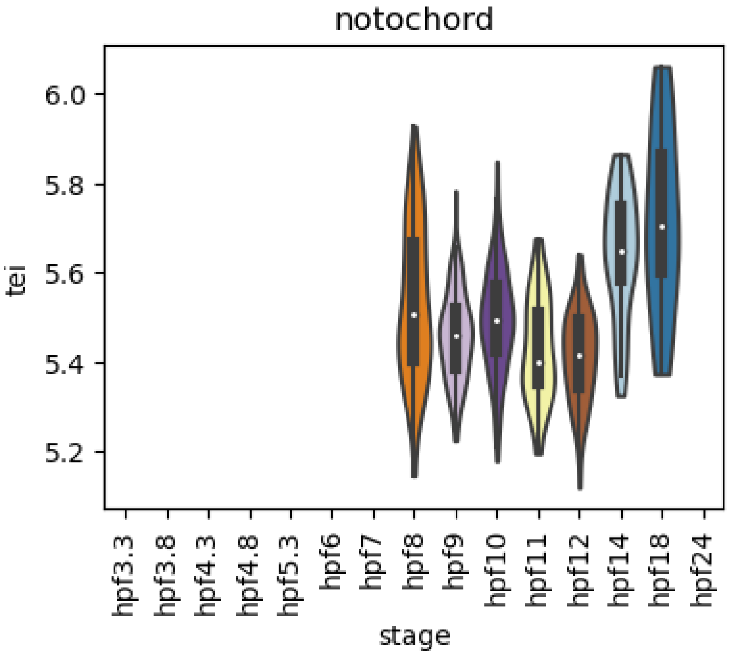
notochord
6.0
5.8
5.6
5.4
5.2
tei
hpf3.3
hpf3.8
hpf4.3
hpf4.8
hpf5.3
hpf6
hpf7
hpf8
hpf9
hpf10
hpf11
hpf12
hpf14
hpf18
hpf24
stage

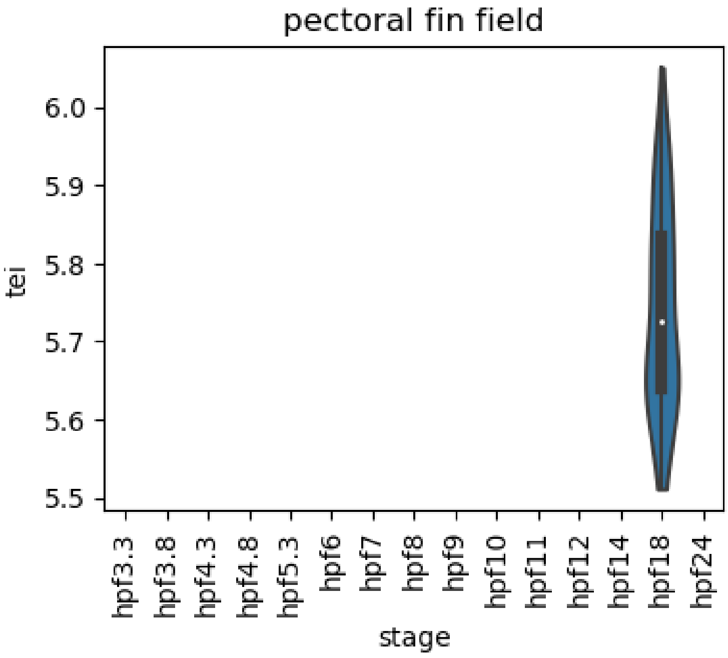
pectoral fin field
tei
6.0
5.9
5.8
5.7
5.6
5.5
hpf3.3
hpf3.8
hpf4.3
hpf4.8
hpf5.3
hpf6
hpf7
hpf8
hpf9
hpf10
hpf11
hpf12
hpf14
hpf18
hpf24
stage

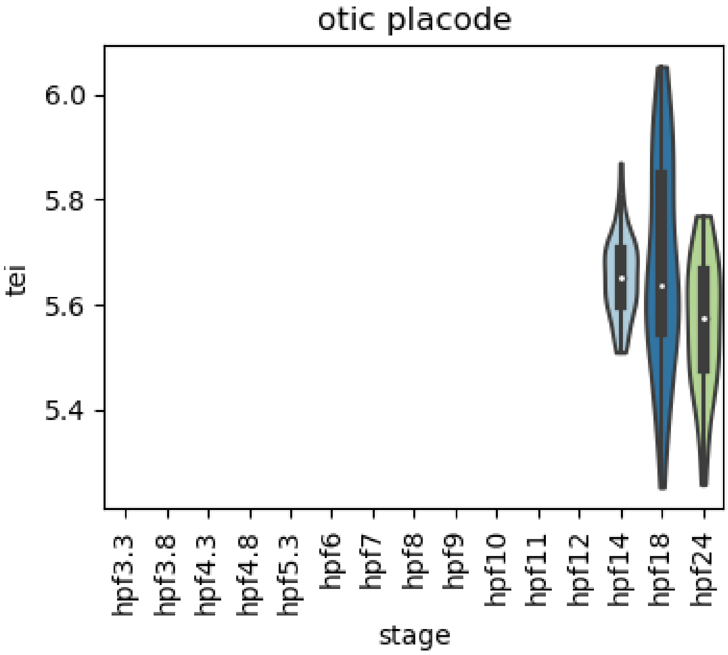
otic placode
tei
6.0
5.8
5.6
5.4
hpf3.3
hpf3.8
hpf4.3
hpf4.8
hpf5.3
hpf6
hpf7
hpf8
hpf9
hpf10
hpf11
hpf12
hpf14
hpf18
hpf24
stage

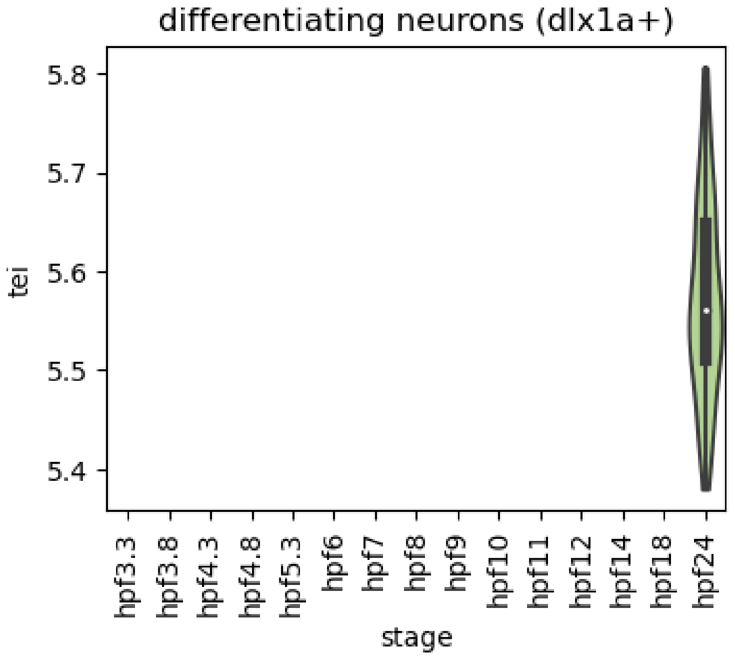
differentiating neurons (dlx1a+)
tei
5.8
5.7
5.6
5.5
5.4
stage
hpf3.3
hpf3.8
hpf4.3
hpf4.8
hpf5.3
hpf6
hpf7
hpf8
hpf9
hpf10
hpf11
hpf12
hpf14
hpf18
hpf24

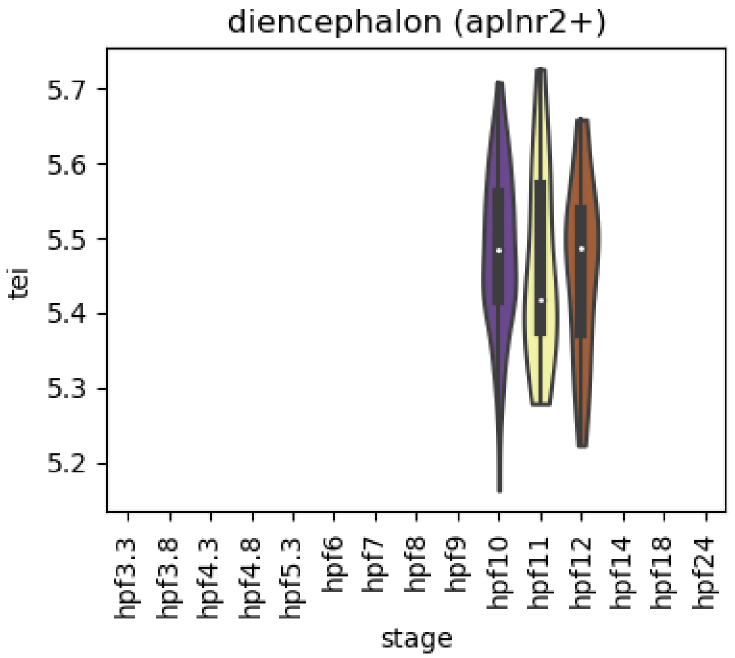
diencephalon (aplnr2+)
tei
5.7
5.6
5.5
5.4
5.3
5.2
stage
hpf3.3
hpf3.8
hpf4.3
hpf4.8
hpf5.3
hpf6
hpf7
hpf8
hpf9
hpf10
hpf11
hpf12
hpf14
hpf18
hpf24

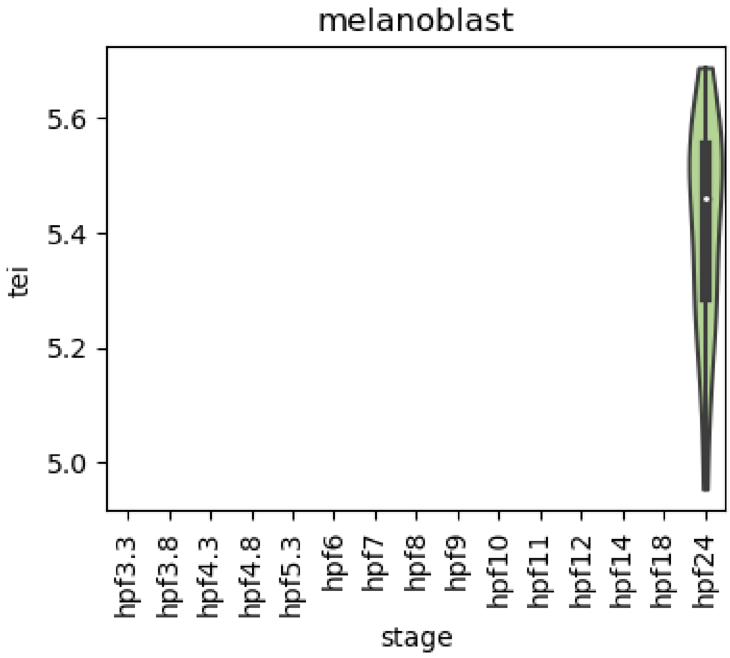
melanoblast
5.6
5.4
5.2
5.0
tei
hpf3.3
hpf3.8
hpf4.3
hpf4.8
hpf5.3
hpf6
hpf7
hpf8
hpf9
hpf10
hpf11
hpf12
hpf14
hpf18
hpf24
stage

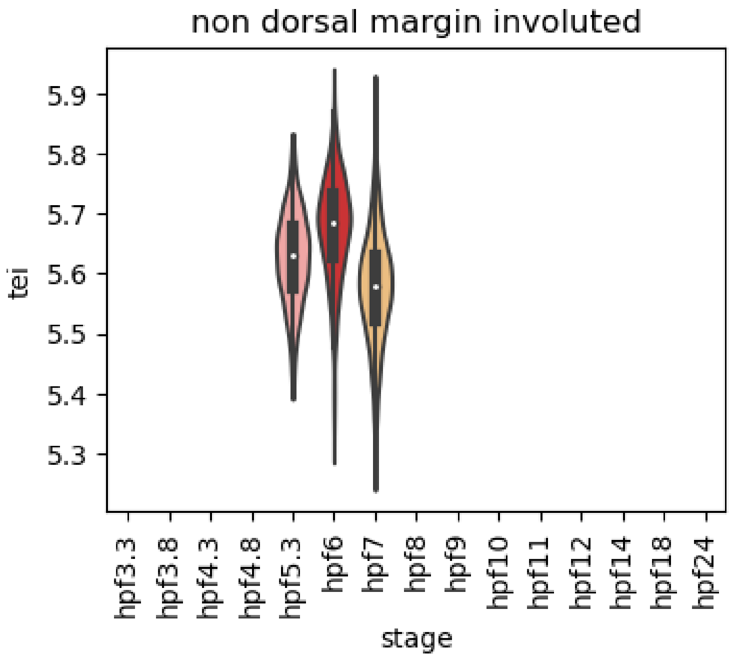
non dorsal margin involuted
5.9
5.8
5.7
5.6
5.5
5.4
5.3
tei
hpf3.3
hpf3.8
hpf4.3
hpf4.8
hpf5.3
hpf6
hpf7
hpf8
hpf9
hpf10
hpf11
hpf12
hpf14
hpf18
hpf24
stage

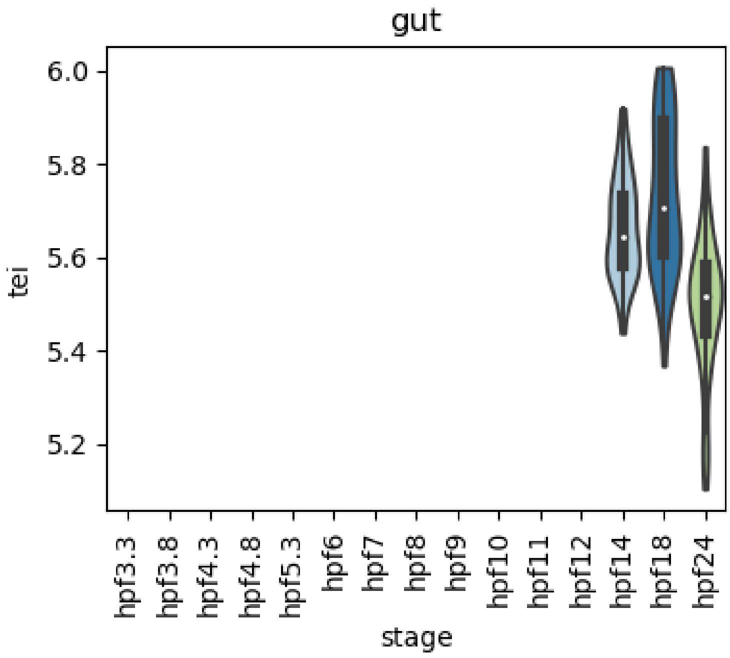
gut
6.0
5.8
5.6
5.4
5.2
tei
hpf3.3
hpf3.8
hpf4.3
hpf4.8
hpf5.3
hpf6
hpf7
hpf8
hpf9
hpf10
hpf11
hpf12
hpf14
hpf18
hpf24
stage

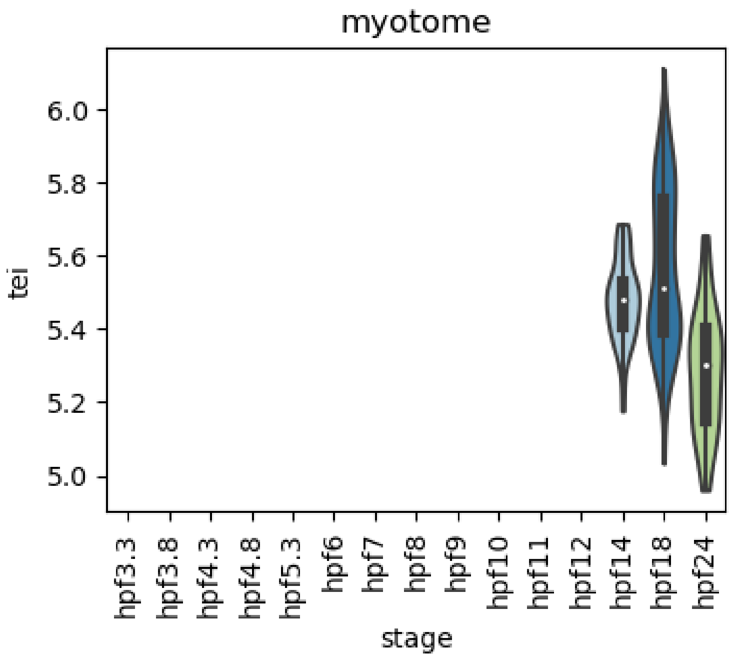
myotome
6.0
5.8
5.6
5.4
5.2
5.0
tei
hpf3.3
hpf3.8
hpf4.3
hpf4.8
hpf5.3
hpf6
hpf7
hpf8
hpf9
hpf10
hpf11
hpf12
hpf14
hpf18
hpf24
stage

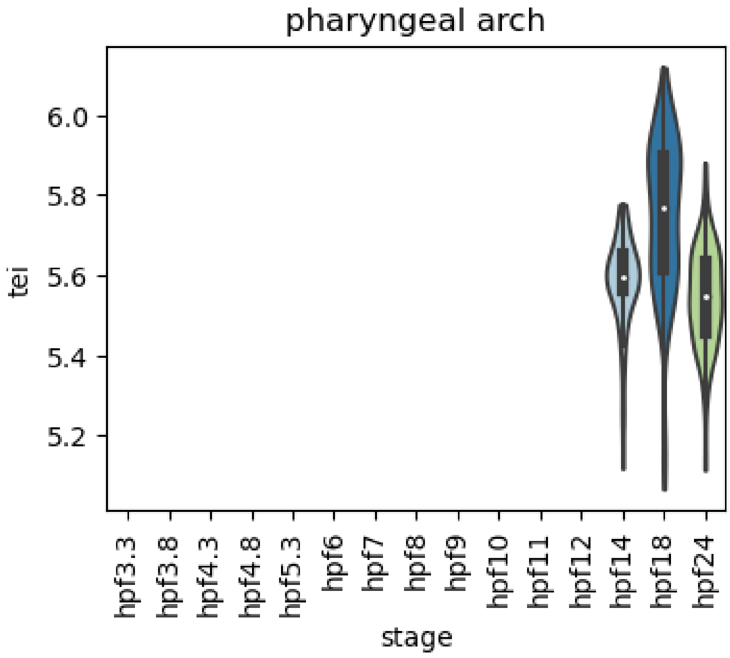
pharyngeal arch
tei
6.0
5.8
5.6
5.4
5.2
hpf3.3
hpf3.8
hpf4.3
hpf4.8
hpf5.3
hpf6
hpf7
hpf8
hpf9
hpf10
hpf11
hpf12
hpf14
hpf18
hpf24
stage

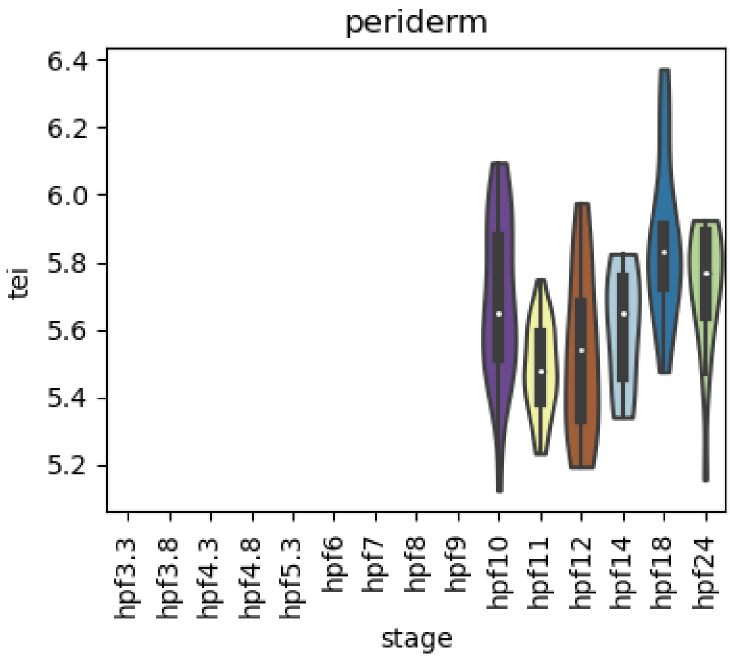
periderm
tei
6.4
6.2
6.0
5.8
5.6
5.4
5.2
hpf3.3
hpf3.8
hpf4.3
hpf4.8
hpf5.3
hpf6
hpf7
hpf8
hpf9
hpf10
hpf11
hpf12
hpf14
hpf18
hpf24
stage

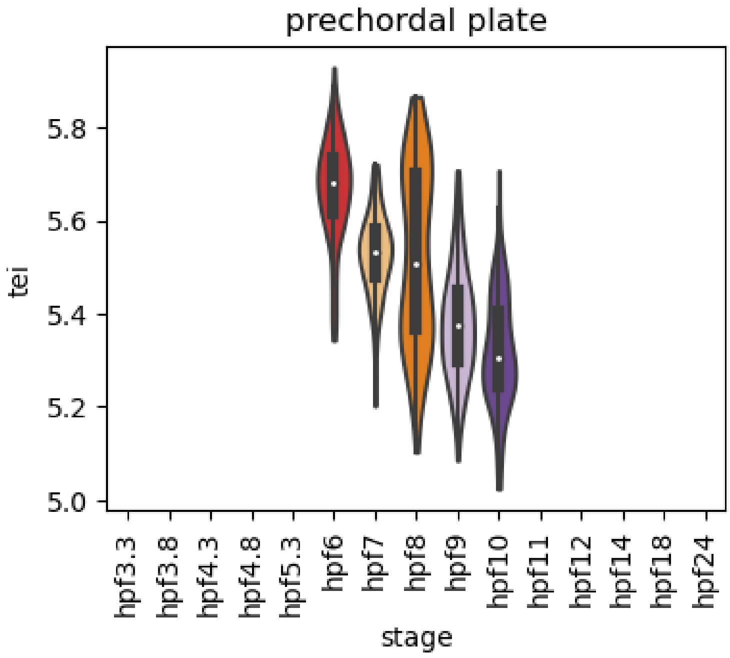
prechordal plate
5.8
5.6
5.4
5.2
5.0
tei
hpf3.3
hpf3.8
hpf4.3
hpf4.8
hpf5.3
hpf6
hpf7
hpf8
hpf9
hpf10
hpf11
hpf12
hpf14
hpf18
hpf24
stage

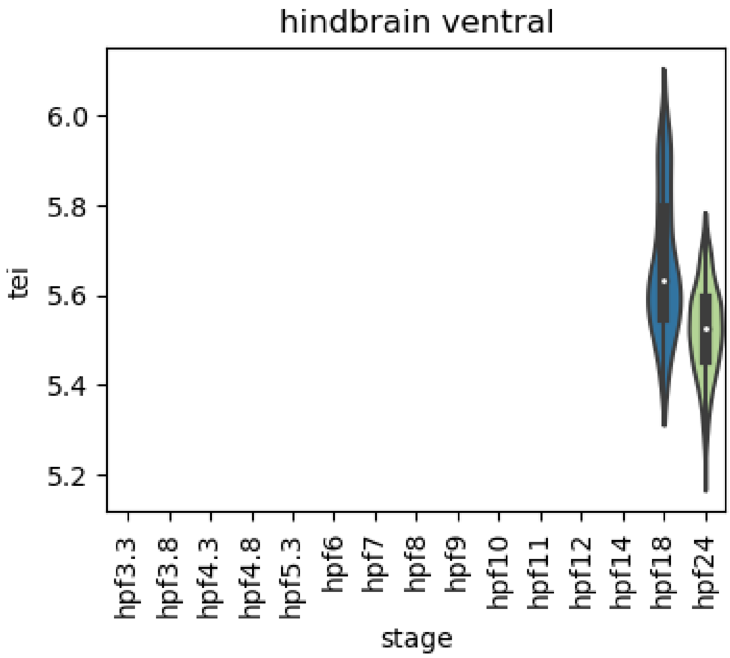
hindbrain ventral
6.0
5.8
5.6
5.4
5.2
tei
hpf3.3
hpf3.8
hpf4.3
hpf4.8
hpf5.3
hpf6
hpf7
hpf8
hpf9
hpf10
hpf11
hpf12
hpf14
hpf18
hpf24
stage

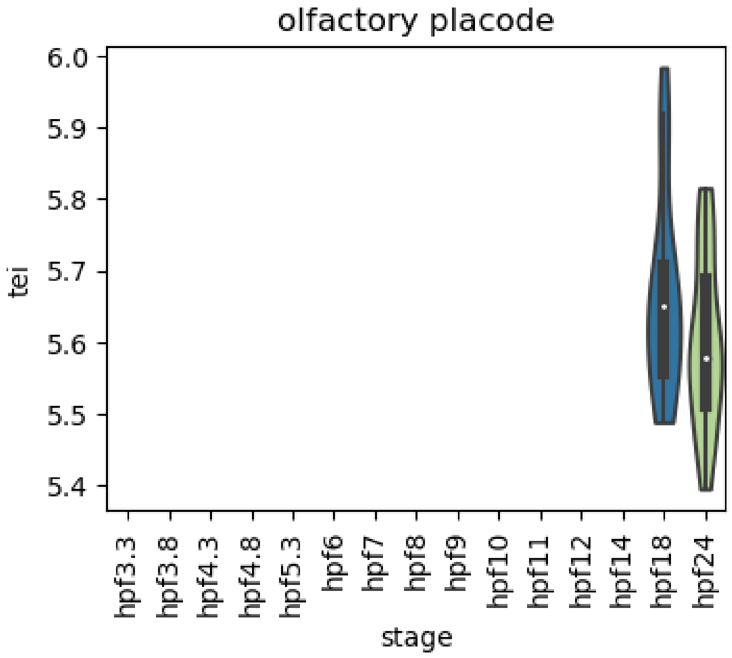
olfactory placode
6.0
5.9
5.8
5.7
5.6
5.5
5.4
tei
hpf3.3
hpf3.8
hpf4.3
hpf4.8
hpf5.3
hpf6
hpf7
hpf8
hpf9
hpf10
hpf11
hpf12
hpf14
hpf18
hpf24
stage

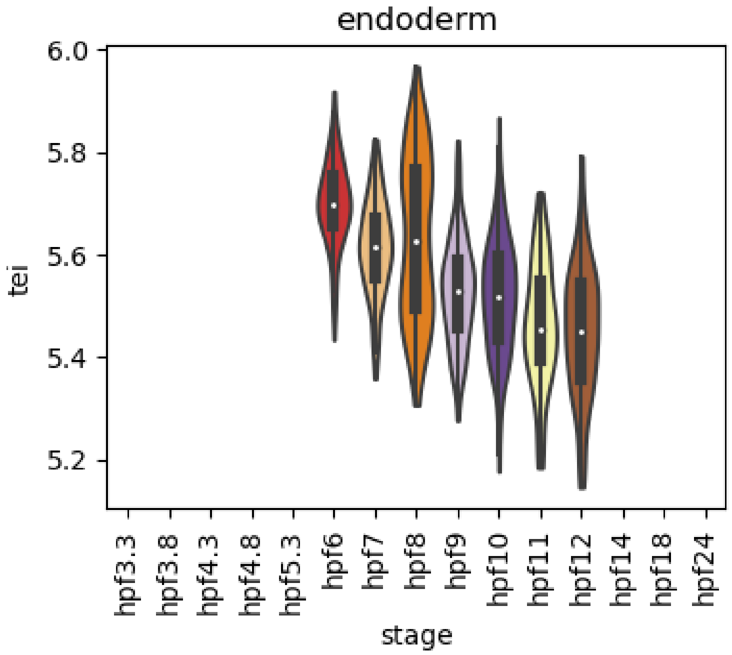
endoderm
6.0
5.8
5.6
5.4
5.2
tei
hpf3.3
hpf3.8
hpf4.3
hpf4.8
hpf5.3
hpf6
hpf7
hpf8
hpf9
hpf10
hpf11
hpf12
hpf14
hpf18
hpf24
stage

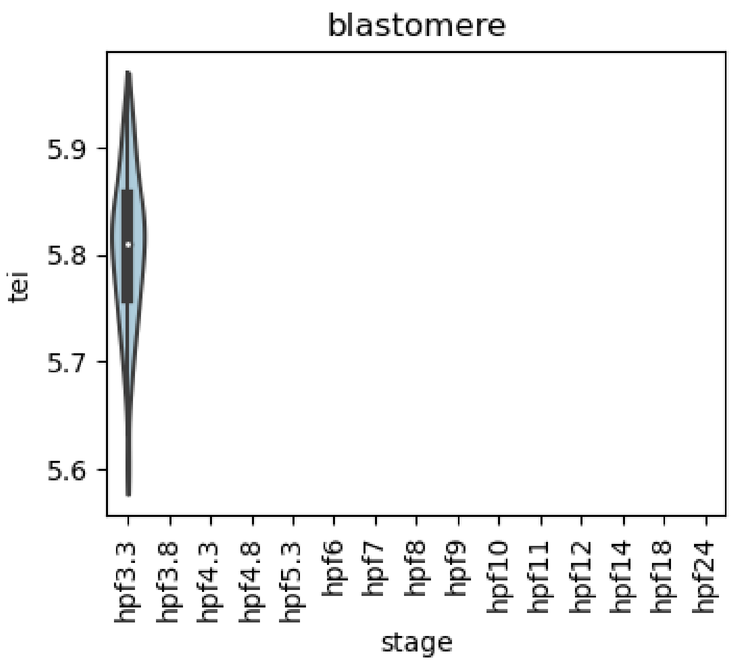
blastomere
tei
5.9
5.8
5.7
5.6
hpf3.3
hpf3.8
hpf4.3
hpf4.8
hpf5.3
hpf6
hpf7
hpf8
hpf9
hpf10
hpf11
hpf12
hpf14
hpf18
hpf24
stage

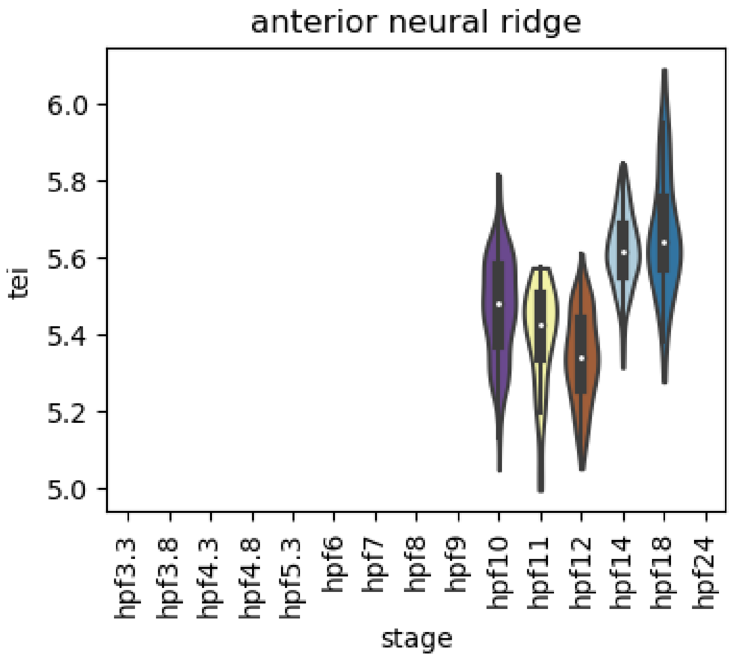
anterior neural ridge
tei
6.0
5.8
5.6
5.4
5.2
5.0
hpf3.3
hpf3.8
hpf4.3
hpf4.8
hpf5.3
hpf6
hpf7
hpf8
hpf9
hpf10
hpf11
hpf12
hpf14
hpf18
hpf24
stage

1.10.4 Plot relative expression per gene age class per sample stage

[28]:
```
zebrafish_data_rematrix_grouped = orthomap2tei.get_rematrix(
    adata=zebrafish_data,
    gene_id=query_orthomap['geneID'],
    gene_age=query_orthomap['PSnum'],
    keep='min',
    layer=None,
    use='counts',
    var_type='mean',
    group_by_obs='stage',
    obs_fillna='__NaN',
    obs_type='mean',
    standard_scale=0,
    normalize_total=True,
    log1p=True,
    target_sum=1e6)
zebrafish_data_rematrix_grouped
```

[28]:
```
stage    hpf3.3    hpf3.8    hpf4.3    hpf4.8    hpf5.3      hpf6      hpf7  \
ps
3      1.000000  0.763787  0.466752  0.284268  0.483931  0.481564  0.384060
6      1.000000  0.809081  0.437535  0.269733  0.388836  0.503856  0.297106
8      1.000000  0.824060  0.414671  0.254490  0.355464  0.470074  0.260572
10     1.000000  0.831077  0.411780  0.261339  0.357994  0.476349  0.260767
11     1.000000  0.865600  0.378906  0.272923  0.352424  0.471416  0.255806
13     1.000000  0.879969  0.452083  0.314456  0.410616  0.532115  0.287119
14     1.000000  0.899687  0.449817  0.328953  0.400997  0.633085  0.233571
16     1.000000  0.867723  0.548742  0.564756  0.774593  0.670847  0.604867
18     1.000000  0.869782  0.452952  0.341595  0.478496  0.249529  0.369100
19     1.000000  0.992605  0.858099  0.824152  0.884298  0.355240  0.668145
20     1.000000  0.735228  0.958915  0.727300  0.850712  0.292708  0.749759
22     1.000000  0.785783  0.241326  0.218616  0.325823  0.594873  0.328793
25     1.000000  0.998153  0.572311  0.474392  0.540644  0.265468  0.346071
29     0.925673  1.000000  0.660387  0.587410  0.709576  0.933434  0.548793

stage      hpf8      hpf9     hpf10     hpf11     hpf12     hpf14     hpf18  \
ps
3      0.324288  0.200180  0.263576  0.000000  0.008070  0.464345  0.564625
6      0.313188  0.155969  0.241321  0.000000  0.003263  0.499420  0.636556
8      0.288058  0.123445  0.193471  0.000000  0.003817  0.458995  0.653966
10     0.288836  0.127462  0.208406  0.000000  0.005533  0.490026  0.667754
11     0.295930  0.109932  0.183937  0.000000  0.006799  0.412106  0.616927
13     0.334316  0.129218  0.223298  0.000000  0.001074  0.544824  0.667480
14     0.321922  0.123774  0.193121  0.000000  0.000249  0.525102  0.875673
16     0.476988  0.311682  0.261083  0.048721  0.000000  0.430039  0.634124
18     0.208163  0.168413  0.066178  0.026141  0.000000  0.094423  0.185906
19     0.444361  0.441715  0.234261  0.172212  0.132878  0.091709  0.200682
20     0.473234  0.571193  0.319447  0.385633  0.362103  0.009518  0.087335
22     0.346191  0.088635  0.202888  0.000000  0.020581  0.356134  0.392432
25     0.225424  0.259061  0.147149  0.144565  0.127149  0.048807  0.176999
29     0.472150  0.218427  0.263950  0.007757  0.000000  0.364540  0.347877
```

```
stage      hpf24
ps
3       0.349337
6       0.371327
8       0.335948
10      0.351927
11      0.344885
13      0.312890
14      0.394390
16      0.286768
18      0.080072
19      0.000000
20      0.000000
22      0.033779
25      0.000000
29      0.170495
```

[29]:
```
ax = sns.lineplot(zebrafish_data_rematrix_grouped.transpose(), palette='Accent',␣
 ↪dashes=False)
ax.legend(fontsize=5, title='age class')
ax.set_title('D. rerio - Relative expression per embryo stage')
ax.set_xlabel('embryo stage')
ax.set_ylabel('Relative expression level')
sns.move_legend(ax, 'upper left', bbox_to_anchor=(1, 1))
#plt.tick_params(labelsize=3)
plt.xticks(rotation=90)
plt.show()
```

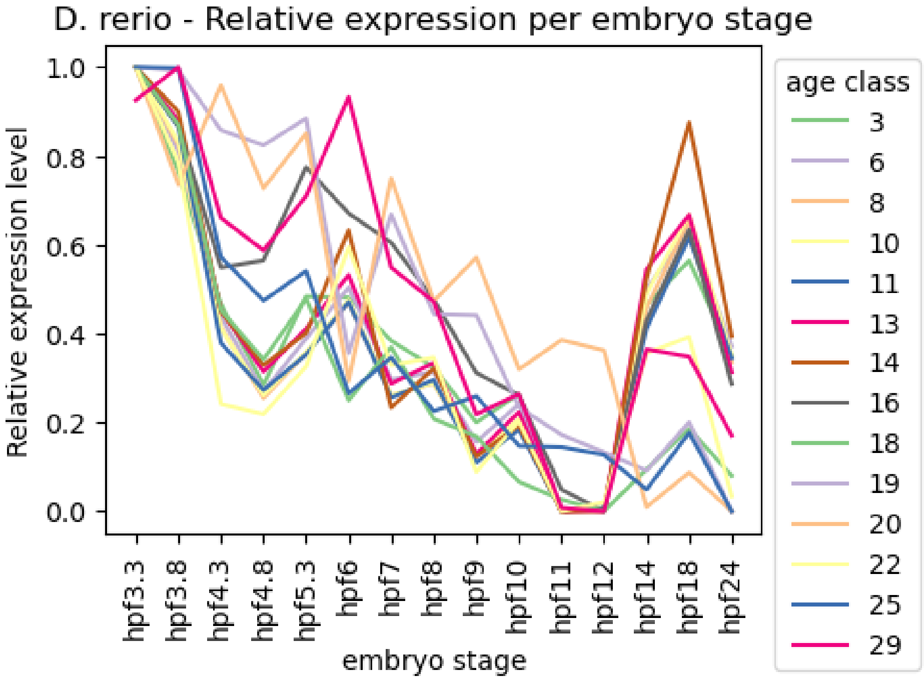

1.10.5 Get partial TEI values to visualize gene age class contributions

Partial `TEI` values can give an idea about which gene age class contributed at most to the `global TEI` pattern.

In detail, each gene gets a `TEI` contribution profile as follows:

$TEI_{is} = f_{is} * ps_i$

, where TEI_{is} is the partial `TEI` value of gene i, $f_{is} = e_{is} / \sum e_{is}$ and ps_i is the phylostratum of gene i.

TEI_{is} values are combined per ps.

The partial `TEI` values combined per strata give an overall impression of the contribution of each strata to the `global TEI` pattern.

One can either start from `counts` (`adata.X`) which is set as default or any other `layer` defined by the `layer` option (`layer=None`).

In addition, the `counts` can be `normalized` and `log-transformed` prior calculating partial `TEI` values (`normalize_total=False`, `log1p=False`, `target_sum=1e6`).

Further, these values can be combined per given observation, e.g. cell typer per sample timepoint (`group_by='cell_state'`).

The `get_pstrata` function of the `orthomap2tei` submodule will return two matrix, the first contains the sum of each partial `TEI` per gene age class and the second the corresponding frequencies.

Both can be further processed by returning the `cumsum` over the gene age classes. To get them set the option `cumsum=True`. The `cumsum` will result in either for the first matrix the `TEI` value per cell or mean `TEI` value per group, if one choose a observation with the `group_by` option. Or in case of the second frequency matrix will result in 1.

With the `standard_scale` option either gene age classes (`standard_scale=0` rows) or cells or groups (`standard_scale=1` columns) can be scaled, subtract the minimum and divide each by its maximum. By default no scaling is applied (`standard_scale=None`).

The resulting data will be visualized in the downstream section.

[30]:
```
zebrafish_pstrata = orthomap2tei.get_pstrata(adata=zebrafish_data,
    gene_id=query_orthomap['geneID'],
    gene_age=query_orthomap['PSnum'],
    keep='min',
    layer=None,
    cumsum=False,
    group_by_obs='stage',
    obs_fillna='__NaN',
    obs_type='mean',
    standard_scale=None,
    normalize_total=True,
    log1p=True,
    target_sum=1e6)
zebrafish_pstrata[0]
```

[30]:
```
stage    hpf3.3    hpf3.8    hpf4.3    hpf4.8    hpf5.3      hpf6      hpf7  \
ps
3      1.113356  1.114005  1.229654  1.273117  1.278153  1.197770  1.313577
6      2.637362  2.616733  2.497146  2.419261  2.424246  2.556530  2.393082
8      0.517103  0.514600  0.454584  0.430204  0.432281  0.463723  0.415157
10     0.678416  0.685693  0.622953  0.612870  0.600758  0.637421  0.585663
```

```
11      0.247444  0.254401  0.206657  0.208631  0.203748  0.221855  0.194637
13      0.135881  0.141672  0.131241  0.132061  0.128311  0.136144  0.122029
14      0.033937  0.035818  0.030766  0.030896  0.029582  0.035797  0.025323
16      0.046340  0.048377  0.051122  0.060191  0.061868  0.053915  0.060276
18      0.100601  0.104195  0.091349  0.093186  0.096897  0.068719  0.093560
19      0.094886  0.105762  0.129563  0.148972  0.135150  0.078638  0.123682
20      0.020965  0.020492  0.029569  0.030210  0.028496  0.018420  0.029210
22      0.032415  0.031151  0.021227  0.023941  0.024829  0.033635  0.027177
25      0.078058  0.088461  0.086041  0.092972  0.085865  0.061021  0.077996
29      0.068072  0.078519  0.079801  0.084859  0.085270  0.102441  0.076930

stage       hpf8      hpf9     hpf10     hpf11     hpf12     hpf14     hpf18  \
ps
3       1.281811  1.363028  1.330284  1.425889  1.429682  1.193865  1.152226
6       2.444176  2.345638  2.406803  2.245628  2.243321  2.590851  2.618168
8       0.429359  0.390495  0.402755  0.373524  0.373645  0.466532  0.502338
10      0.604837  0.573526  0.589225  0.571378  0.572683  0.658186  0.684621
11      0.204859  0.178078  0.185804  0.174756  0.176164  0.211250  0.230991
13      0.128530  0.116387  0.121953  0.116905  0.115877  0.140308  0.138097
14      0.028372  0.024326  0.025258  0.022640  0.022564  0.032801  0.039593
16      0.054464  0.055294  0.048341  0.050639  0.046917  0.044654  0.046494
18      0.076011  0.081326  0.064668  0.076974  0.071033  0.052164  0.054506
19      0.101955  0.116639  0.087341  0.101105  0.094096  0.050310  0.052783
20      0.025264  0.030754  0.024892  0.033129  0.032192  0.013354  0.013284
22      0.027644  0.020457  0.023461  0.020015  0.021431  0.025511  0.022939
25      0.069783  0.084367  0.071543  0.092356  0.089403  0.044680  0.047207
29      0.068802  0.049840  0.049623  0.031444  0.030379  0.052481  0.044994

stage      hpf24
ps
3       1.238431
6       2.538900
8       0.448213
10      0.636619
11      0.216543
13      0.122644
14      0.031172
16      0.043846
18      0.057179
19      0.046790
20      0.015106
22      0.014938
25      0.046444
29      0.038655
```

[31]:
```
#plt.rcParams['figure.figsize'] = [9, 4.5]
ax=zebrafish_pstrata[0].transpose().plot.area(cmap='tab20')
ax.legend(fontsize=3, title='age class')
ax.set_title('D. rerio - Contribution of gene age classes to global TEI')
ax.set_xlabel('embryo stage')
ax.set_ylabel('TEI')
sns.move_legend(ax, 'upper left', bbox_to_anchor=(1, 1))
plt.xticks(rotation=90)
```

```
plt.show()
#plt.rcParams['figure.figsize'] = [6, 4.5]
```

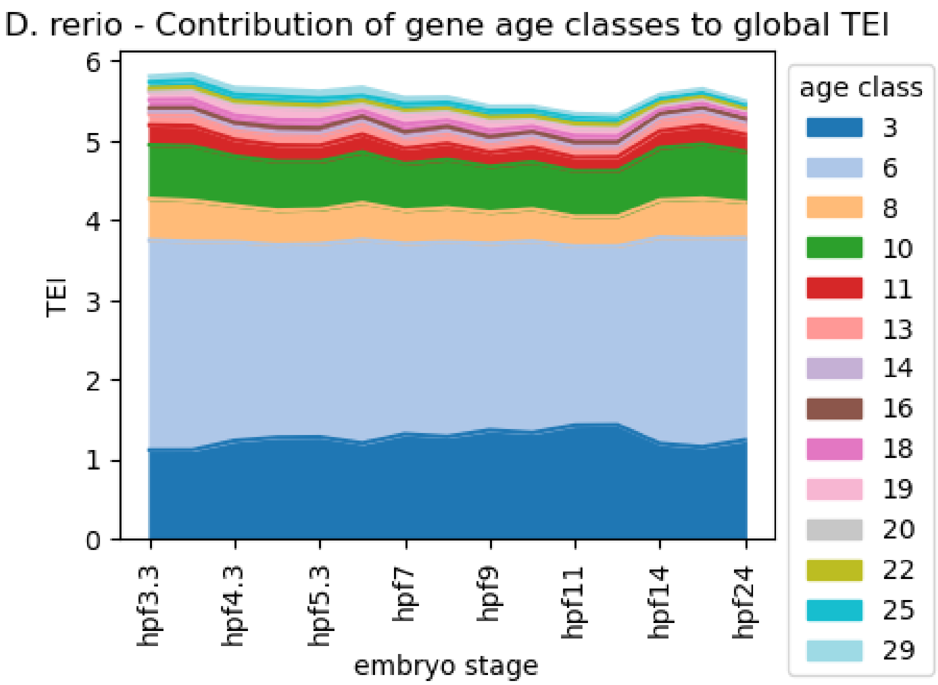

1.10.6 Gene age class contribution of one cell type

EVL:

Note: Please change notebook cell from `raw` to `code` to see the plots.

```
zebrafish_pstrata_cell_type = orthomap2tei.get_pstrata(
    adata=zebrafish_data[zebrafish_data.obs['cell_type']=='EVL'],
    gene_id=query_orthomap['geneID'],
    gene_age=query_orthomap['PSnum'],
    keep='min',
    layer=None,
    cumsum=False,
    group_by_obs='stage',
    obs_fillna='__NaN',
    obs_type='mean',
    standard_scale=None,
    normalize_total=True,
    log1p=True,
    target_sum=1e6)
zebrafish_pstrata_cell_type[0]
```

Note: Please change notebook cell from `raw` to `code` to see the plots.

```
#plt.rcParams['figure.figsize'] = [9, 4.5]
ax=zebrafish_pstrata_cell_type[0].transpose().plot.area(cmap='tab20')
ax.legend(fontsize=3, title='age class')
```

```
ax.set_title('D. rerio - cell type: EVL - Contribution of gene age classes to global TEI')
ax.set_xlabel('embryo stage')
ax.set_ylabel('TEI')
sns.move_legend(ax, 'upper left', bbox_to_anchor=(1, 1))
plt.xticks(rotation=90)
plt.show()
#plt.rcParams['figure.figsize'] = [6, 4.5]
    haticing gland:
```

Note: Please change notebook cell from `raw` to `code` to see the plots.

```
zebrafish_pstrata_cell_type = orthomap2tei.get_pstrata(
    adata=zebrafish_data[zebrafish_data.obs['cell_type']=='hatching gland'],
    gene_id=query_orthomap['geneID'],
    gene_age=query_orthomap['PSnum'],
    keep='min',
    layer=None,
    cumsum=False,
    group_by_obs='stage',
    obs_fillna='__NaN',
    obs_type='mean',
    standard_scale=None,
    normalize_total=True,
    log1p=True,
    target_sum=1e6)
zebrafish_pstrata_cell_type[0]
```

Note: Please change notebook cell from `raw` to `code` to see the plots.

```
#plt.rcParams['figure.figsize'] = [9, 4.5]
ax=zebrafish_pstrata_cell_type[0].transpose().plot.area(cmap='tab20')
ax.legend(fontsize=3, title='age class')
ax.set_title('D. rerio - cell type: hatching gland - Contribution of gene age classes to global TEI')
ax.set_xlabel('embryo stage')
ax.set_ylabel('TEI')
sns.move_legend(ax, 'upper left', bbox_to_anchor=(1, 1))
plt.xticks(rotation=90)
plt.show()
#plt.rcParams['figure.figsize'] = [6, 4.5]
    endoderm:
```

Note: Please change notebook cell from `raw` to `code` to see the plots.

```
zebrafish_pstrata_cell_type = orthomap2tei.get_pstrata(
    adata=zebrafish_data[zebrafish_data.obs['cell_type']=='endoderm'],
    gene_id=query_orthomap['geneID'],
    gene_age=query_orthomap['PSnum'],
    keep='min',
    layer=None,
    cumsum=False,
    group_by_obs='stage',
    obs_fillna='__NaN',
    obs_type='mean',
    standard_scale=None,
    normalize_total=True,
    log1p=True,
    target_sum=1e6)
```

```
zebrafish_pstrata_cell_type[0]
```

Note: Please change notebook cell from `raw` to `code` to see the plots.

```
#plt.rcParams['figure.figsize'] = [9, 4.5]
ax=zebrafish_pstrata_cell_type[0].transpose().plot.area(cmap='tab20')
ax.legend(fontsize=3, title='age class')
ax.set_title('D. rerio - cell type: endoderm - Contribution of gene age classes to global TEI')
ax.set_xlabel('embryo stage')
ax.set_ylabel('TEI')
sns.move_legend(ax, 'upper left', bbox_to_anchor=(1, 1))
plt.xticks(rotation=90)
plt.show()
#plt.rcParams['figure.figsize'] = [6, 4.5]
```

1.10.7 Color UMAP/TSNE by TEI

Follwoing the basic tutorial of the Scanpy python toolkit (Wolf et al., 2018), one can highlight `TEI` values on a dimensional reduction of the scRNA dataset, like `PCA`, `UMAP` or `TSNE`.

Filtering

[32]:
```
sc.pp.filter_genes(zebrafish_data, min_cells=3)
sc.pp.filter_cells(zebrafish_data, min_genes=200)
```

Normalization, Log transformation and Scaling

[33]:
```
sc.pp.normalize_total(zebrafish_data, target_sum=1e6)
sc.pp.log1p(zebrafish_data)
sc.pp.scale(zebrafish_data, max_value=10)
```

PCA and Neighbor calculations

[34]:
```
sc.tl.pca(zebrafish_data, svd_solver='arpack')
sc.pl.pca(zebrafish_data, color=['stage', 'tei'])
```

```
/opt/anaconda3/envs/scanpy/lib/python3.8/site-
packages/scanpy/plotting/_tools/scatterplots.py:392: UserWarning: No data for
colormapping provided via 'c'. Parameters 'cmap' will be ignored
  cax = scatter(
```

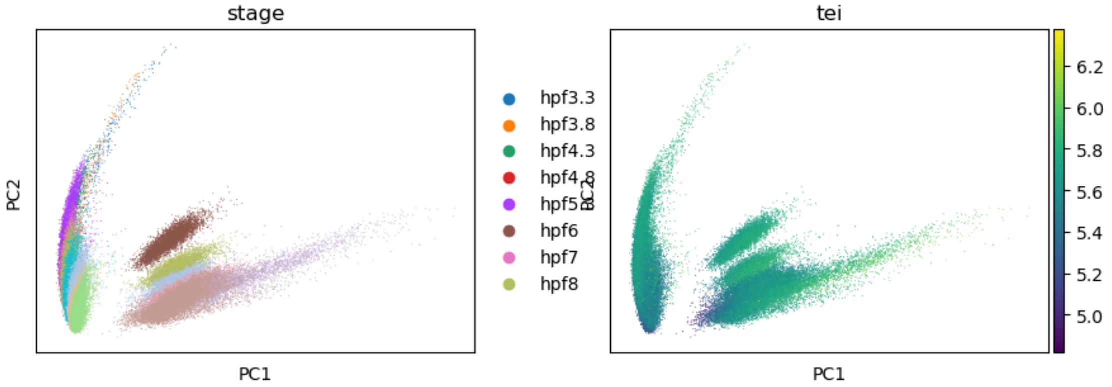

[35]:
```
sc.pp.neighbors(zebrafish_data)
```

Embedding the neighborhood graph

[36]:
```
sc.tl.paga(zebrafish_data, groups='stage')
sc.pl.paga(zebrafish_data, title='D. rerio - embryo stage - PAGA graph')
```

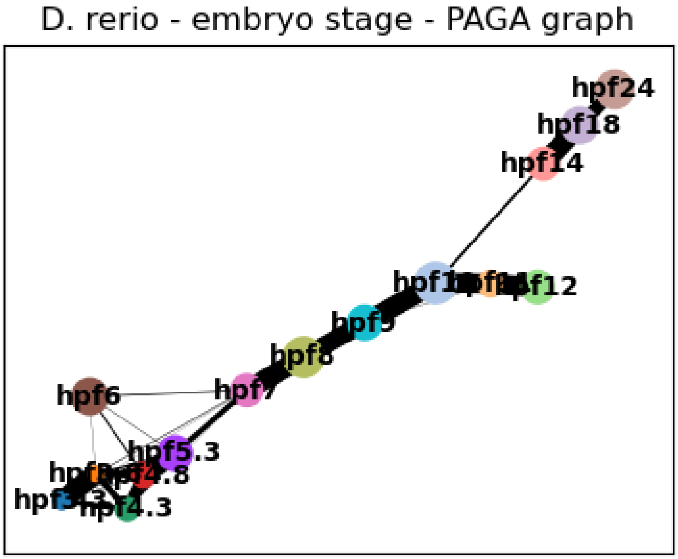

UMAP

[37]:
```
sc.tl.umap(zebrafish_data,
           init_pos='paga')
sc.pl.umap(zebrafish_data,
           title='D. rerio - embryo stage - UMAP', color=['stage'])
```

```
/opt/anaconda3/envs/scanpy/lib/python3.8/site-
packages/scanpy/plotting/_tools/scatterplots.py:392: UserWarning: No data for
colormapping provided via 'c'. Parameters 'cmap' will be ignored
  cax = scatter(
```

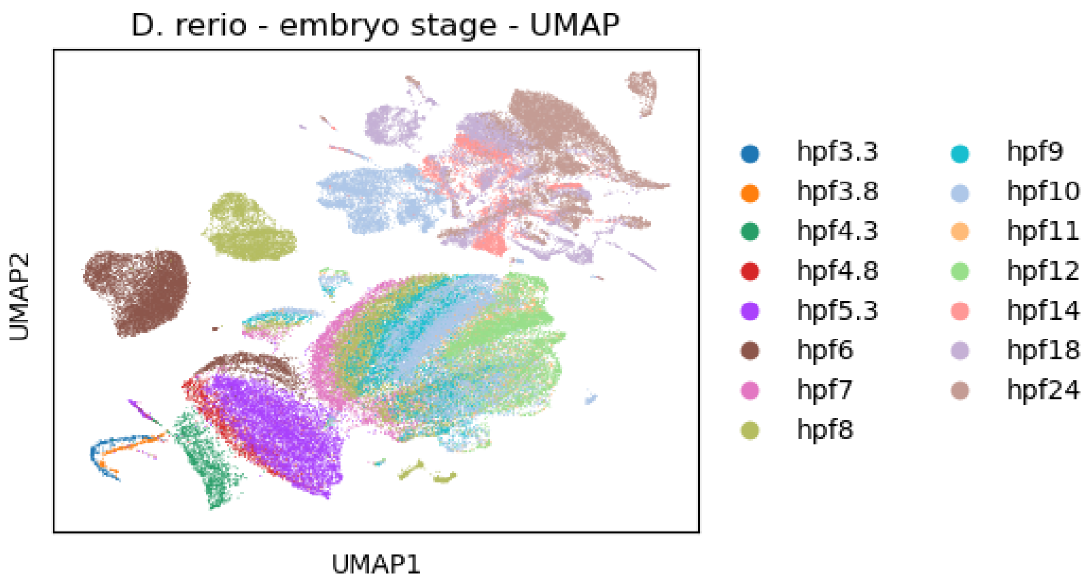

[38]:
```
#plt.rcParams['figure.figsize'] = [7.5, 4.5]
sc.pl.umap(zebrafish_data,
           title='D. rerio - TEI - UMAP',
           color=['tei'],
           color_map='viridis',
           vmin='p5',
           vmax='p95')
#plt.rcParams['figure.figsize'] = [6, 4.5]
```

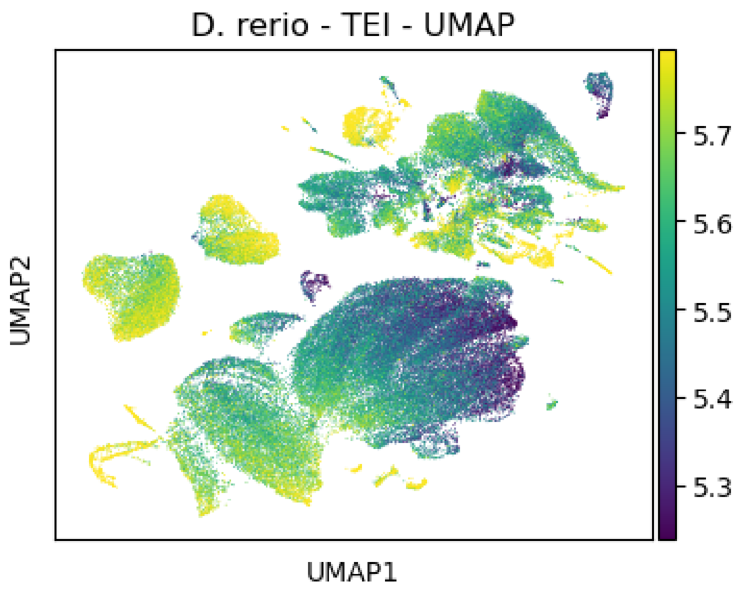

corresponds to main Figure 1F

[39]:
```
plt.rcParams['figure.figsize'] = [7.5, 4.5]
#3d
sc.tl.umap(zebrafish_data,
           n_components=3)
sc.pl.umap(zebrafish_data,
           title='D. rerio - embryo stage - UMAP', color=['stage'],
           projection='3d')
plt.rcParams['figure.figsize'] = [4.4, 3.3]
```

```
/opt/anaconda3/envs/scanpy/lib/python3.8/site-
packages/scanpy/plotting/_tools/scatterplots.py:325: UserWarning: No data for
colormapping provided via 'c'. Parameters 'cmap' will be ignored
  cax = ax.scatter(
```

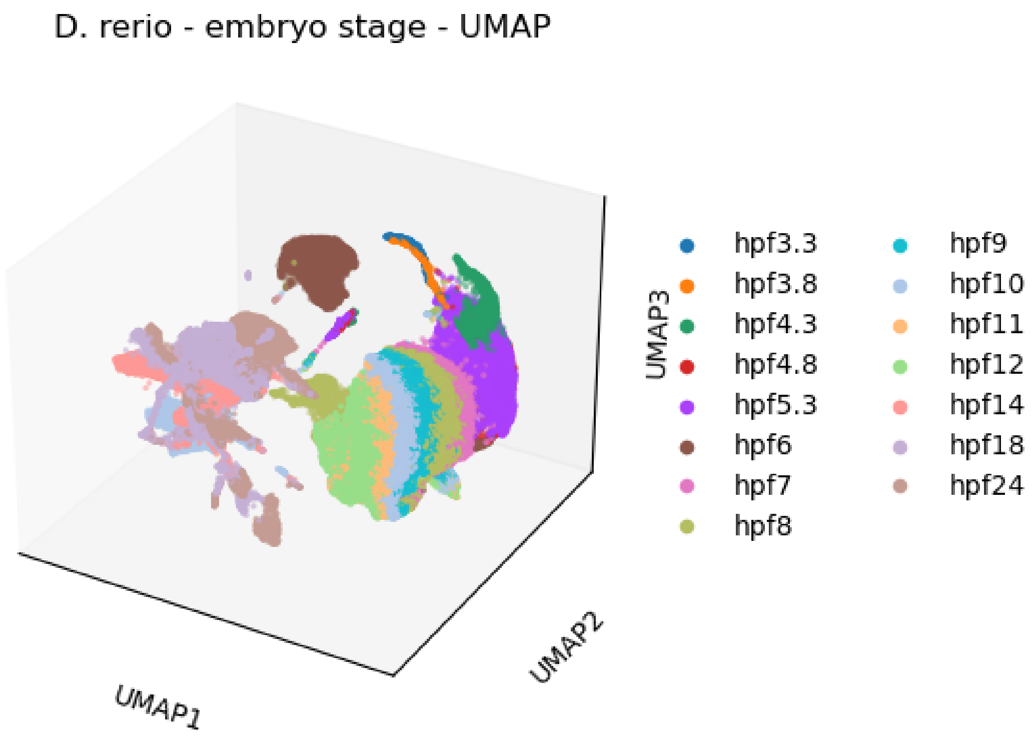

corresponds to main Figure 1G

[40]:
```
plt.rcParams['figure.figsize'] = [7.5, 4.5]
sc.pl.umap(zebrafish_data,
           title='D. rerio - TEI - UMAP',
           color=['tei'],
           color_map='viridis',
           vmin='p5',
           vmax='p95',
           projection='3d')
plt.rcParams['figure.figsize'] = [4.4, 3.3]
```

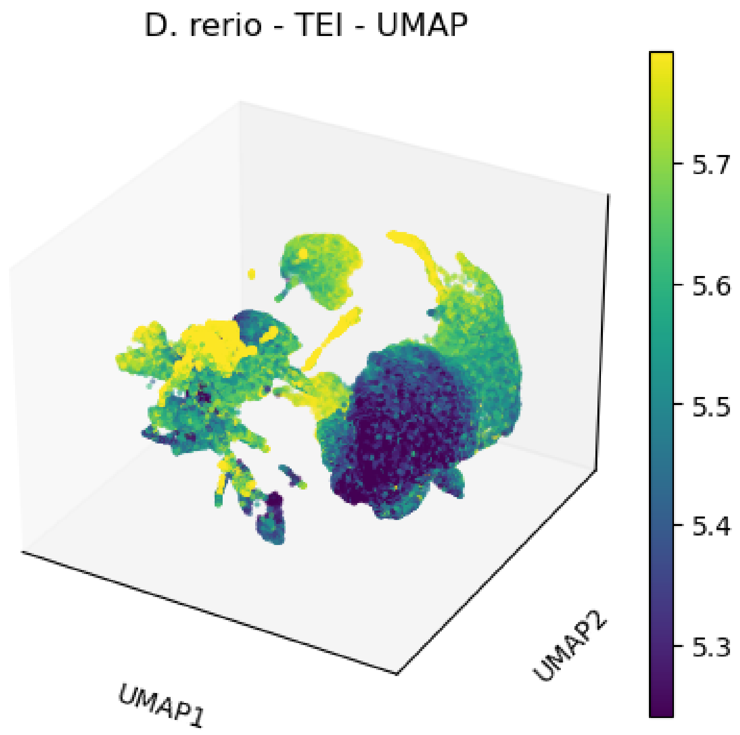

[41]:
```
import plotly
import kaleido
import plotly.express as px
plotly.offline.init_notebook_mode(connected=False)
```

[42]:
```
zebrafish_data
```

[42]:
```
AnnData object with n_obs × n_vars = 71203 × 19761
    obs: 'orig.ident', 'nCount_RNA', 'nFeature_RNA', 'sample', 'stage', 'group',
'cell_state', 'cell_type', 'tei', 'n_genes'
    var: 'features', 'genes', 'Phylostrata', 'n_cells', 'mean', 'std'
    uns: 'log1p', 'pca', 'stage_colors', 'neighbors', 'paga', 'stage_sizes',
'umap'
    obsm: 'X_pca', 'X_umap'
    varm: 'PCs'
    layers: 'counts'
    obsp: 'distances', 'connectivities'
```

[43]:
```
df = pd.DataFrame( {'UMAP1':zebrafish_data.obsm['X_umap'][:,0],
                    'UMAP2':zebrafish_data.obsm['X_umap'][:,1],
                    'UMAP3':zebrafish_data.obsm['X_umap'][:,2],
                    'cell_type':zebrafish_data.obs['cell_type'].values.astype(str),
                    'cell_state':zebrafish_data.obs['cell_state'].values.astype(str),
                    'tei':zebrafish_data.obs['tei'].values,
                    'stage':zebrafish_data.obs['stage'].values,
                    'cell_id':zebrafish_data.obs.index.to_list()  } )
df.set_index('cell_id', inplace = True)
```

```
df.head()
```

[43]:

```
                                   UMAP1     UMAP2      UMAP3   cell_type  \
cell_id
hpf3.3_ZFHIGH_WT_DS5_AAAAGTTGCCTC  8.001603  7.798748  13.366315  blastomere
hpf3.3_ZFHIGH_WT_DS5_AAACAAGTGTAT  8.396315  7.608164  11.704103  blastomere
hpf3.3_ZFHIGH_WT_DS5_AAACACCTCGTC  8.022252  7.873591  12.900765  blastomere
hpf3.3_ZFHIGH_WT_DS5_AAATGAGGTTTN  7.795810  7.474202  14.216955  blastomere
hpf3.3_ZFHIGH_WT_DS5_AACCCTCTCGAT  7.463636  5.477603  16.006929  blastomere

                                           cell_state       tei   stage
cell_id
hpf3.3_ZFHIGH_WT_DS5_AAAAGTTGCCTC  hpf3.3:blastomere  5.847391  hpf3.3
hpf3.3_ZFHIGH_WT_DS5_AAACAAGTGTAT  hpf3.3:blastomere  5.794241  hpf3.3
hpf3.3_ZFHIGH_WT_DS5_AAACACCTCGTC  hpf3.3:blastomere  5.783182  hpf3.3
hpf3.3_ZFHIGH_WT_DS5_AAATGAGGTTTN  hpf3.3:blastomere  5.780480  hpf3.3
hpf3.3_ZFHIGH_WT_DS5_AACCCTCTCGAT  hpf3.3:blastomere  5.915213  hpf3.3
```

[44]:

```
fig = px.scatter_3d(data_frame = df,
                    x='UMAP1',
                    y='UMAP2',
                    z='UMAP3',
                    color='stage')
fig.update_traces(marker_size = 2)
fig.write_html('zebrafish_scatter_3d_stage.html')
```

[45]:

```
fig = px.scatter_3d(data_frame = df,
                    x='UMAP1',
                    y='UMAP2',
                    z='UMAP3',
                    color='tei',
                    range_color=(5,5.5))
fig.update_traces(marker_size = 2)
fig.write_html('zebrafish_scatter_3d_tei.html')
```

[46]:

```
fig = px.scatter_3d(data_frame = df,
                    x='UMAP1',
                    y='UMAP2',
                    z='UMAP3',
                    color='cell_type')
fig.update_traces(marker_size = 2)
fig.write_html('zebrafish_scatter_3d_cell_type.html')
```

[47]:

```
fig = px.scatter_3d(data_frame = df,
                    x='UMAP1',
                    y='UMAP2',
                    z='UMAP3',
                    color='cell_state')
fig.update_traces(marker_size = 2)
fig.write_html('zebrafish_scatter_3d_cell_state.html')
```

Please have a look at the documentation for other case studies.

1 Case study: re-analysis of nematode (*Caenorhabditis elegans*) embryogenesis single-cell data

This notebook will demonstrate scRNA-seq processing with `oggmap` using nematode scRNA data from (Packer and Zhu al., 2019).

scRNA data were obtained from https://www.ncbi.nlm.nih.gov/geo/query/acc.cgi?acc=GSE126954, converted into Scanpy `AnnData` objects (Wolf et al., 2018) and are availabe here:

https://doi.org/10.5281/zenodo.7245547

or can be accessed with the `dataset` submodule of `oggmap`

`datasets.packer19(datapath='data')` (download folder set to `'data'`).

1.1 Notebook file

Notebook file can be obtained here:

https://raw.githubusercontent.com/kullrich/oggmap/main/docs/notebooks/nematode_example.ipynb

1.2 Steps

To process the scRNA data, we will do the following:

0. Use pre-calculated gene age classification
1. Get query species taxonomic lineage information
2. Get query species orthomap
3. Map OrthoFinder gene names and scRNA gene/transcript names
4. Get TEI values and add them to scRNA dataset
5. Get partial TEI values to visualize gene age class contributions
6. Process scRNA data and visualize TEI

1.3 Import libraries

[1]:
```
import numpy as np
import pandas as pd
import scanpy as sc
import seaborn as sns
import matplotlib.pyplot as plt
from statannot import add_stat_annotation
# increase dpi
%matplotlib inline
#plt.rcParams['figure.dpi'] = 300
#plt.rcParams['savefig.dpi'] = 300
```

```
#plt.rcParams['figure.figsize'] = [6, 4.5]
plt.rcParams['figure.figsize'] = [4.4, 3.3]
```

1.4 Import oggmap python package submodules

[2]:
```
# import submodules
from oggmap import qlin, gtf2t2g, of2orthomap, orthomap2tei, datasets
```

1.5 Step 0 - Use pre-calculated gene age classification

Orthomap was pre-calculated (Sun et al., 2021) and is available here:

https://doi.org/10.5281/zenodo.7242263

or can be accessed with the `dataset` submodule of `oggmap`

`datasets.sun21_orthomap(datapath='data')` (download folder set to `'data'`).

If you want to use your own OrthoFinder results:

`oggmap` can extract gene age classification from existing OrthoFinder results and link them with scRNA data.

A detailed how-to is available here:

https://oggmap.readthedocs.io/en/latest/tutorials/orthofinder.html

[3]:
```
# download pre-calculated orthomap into data folder
datasets.sun21_orthomap(datapath='data')
```

```
100% [...] 344640 / 344640
```

[3]:
```
'data/Sun2021_Orthomap.tsv'
```

1.6 Step 0 - Use different pre-calculated evolutionary indices

Diversity parameter were pre-calculated (Ma et al., 2021) and is available here:

https://doi.org/10.5281/zenodo.7242263

or can be accessed with the `dataset` submodule of `oggmap`

`datasets.ma21_orthomap(datapath='data')` (download folder set to `'data'`).

[4]:
```
# download pre-calculated evolutionay indices into data folder
datasets.ma21_fst(datapath='data')
```

```
100% [...] 1049100 / 1049100
```

[4]:
```
'data/Ma2021_Fst.tsv'
```

1.7 Step 1 - get query species taxonomic lineage information

Given a species name or taxonomic ID, the query species lineage information is extracted with the help of the `ete3` python toolkit and the `NCBI taxonomy` (Huerta-Cepas et al., 2016). This information is needed alongside with the taxonomic classifications for all species used in the OrthoFinder comparison.

The oggmap submodule qlin helps to get this information for you with the qlin.get_qlin() function as follows:

```
[5]: # get query species taxonomic lineage information
query_lineage = qlin.get_qlin(q='Caenorhabditis elegans')
```

```
query name: Caenorhabditis elegans
query taxID: 6239
query kingdom: Eukaryota
query lineage names:
['root(1)', 'cellular organisms(131567)', 'Eukaryota(2759)',
'Opisthokonta(33154)', 'Metazoa(33208)', 'Eumetazoa(6072)', 'Bilateria(33213)',
'Protostomia(33317)', 'Ecdysozoa(1206794)', 'Nematoda(6231)',
'Chromadorea(119089)', 'Rhabditida(6236)', 'Rhabditina(2301116)',
'Rhabditomorpha(2301119)', 'Rhabditoidea(55879)', 'Rhabditidae(6243)',
'Peloderinae(55885)', 'Caenorhabditis(6237)', 'Caenorhabditis elegans(6239)']
query lineage:
[1, 131567, 2759, 33154, 33208, 6072, 33213, 33317, 1206794, 6231, 119089, 6236,
2301116, 2301119, 55879, 6243, 55885, 6237, 6239]
```

1.8 Step 2 - gene age class assignment (query species orthomap)

Orthomap was pre-calculated (Sun et al., 2021; see Figure 3B) it is also available here:

https://doi.org/10.5281/zenodo.7242263

or can be accessed with the dataset submodule of oggmap

datasets.sun21_orthomap(datapath='data') (download folder set to 'data').

The pre-calculated orthomap can be imported from the orthomap2tei submodule with the orthomap2tei.read_orthomap() function as follwos:

```
[6]: # get query species orthomap

# download pre-calculated orthomap here: https://doi.org/10.5281/zenodo.7242263
# or download with datasets.sun21_orthomap(datapath='data')
query_orthomap = orthomap2tei.read_orthomap(orthomapfile='data/Sun2021_Orthomap.tsv')
query_orthomap
```

[6]:

	GeneID	Phylostratum
0	WBGene00000001	1
1	WBGene00000002	1
2	WBGene00000003	1
3	WBGene00000004	1
4	WBGene00000005	2
...	...	...
20035	WBGene00305132	9
20036	WBGene00305157	1
20037	WBGene00305158	13
20038	WBGene00305159	11
20039	WBGene00305173	0

[20040 rows x 2 columns]

1.8.1 Get query species evolutionary indices

[7]:
```
# get query species Fst values

# download pre-calculated Fst values here: https://doi.org/10.5281/zenodo.7242263
# or download with datasets.ma21_fst(datapath='data')
query_fst = pd.read_csv('data/Ma2021_Fst.tsv', delimiter='\t')
query_fst
```

[7]:
```
         WormBase_ID  Chr      Gene  TajimaD  NormalizedPi   FayWu     FST
0      WBGene00000001    I     aap-1  -0.6957        0.0002 -1.2575  0.8062
1      WBGene00000002   IV     aat-1  -0.4724        0.0001 -1.4628  0.8846
2      WBGene00000003    V     aat-2  -1.5266        0.0001  0.0816  0.1691
3      WBGene00000004    X     aat-3  -1.6401        0.0003 -4.7685  0.8129
4      WBGene00000005   IV     aat-4  -1.2137        0.0006 -0.7617  0.3725
...               ...  ...       ...      ...           ...     ...     ...
20217  WBGene00271701    X   F10D7.10  -0.7428        0.0000 -1.9308  0.1111
20218  WBGene00271703  III  ZK1010.12  -1.3386        0.0008 -3.2528  0.7683
20219  WBGene00271706   II   D2089.8  -0.8312        0.0012  0.4489  0.5551
20220  WBGene00271707    V   ZK105.14  -1.0748        0.0004 -3.2069  0.6433
20221  WBGene00271715  III   B0244.17  -0.6617        0.0010 -1.1870  0.6556

[20222 rows x 7 columns]
```

1.8.2 Group evolutionary indices into bins

[8]:
```
# see here for additional quantile methods: https://numpy.org/doc/stable/reference/
↪generated/numpy.nanquantile.html

orthomap2tei.get_bins(tobin_df=query_fst,
                      bincol='TajimaD',
                      q=[.1, .2, .3, .4, .5, .6, .7, .8, .9],
                      method='median_unbiased')
orthomap2tei.get_bins(tobin_df=query_fst,
                      bincol='NormalizedPi',
                      q=[.2, .4, .6, .8],
                      method='median_unbiased')
orthomap2tei.get_bins(tobin_df=query_fst,
                      bincol='FayWu',
                      q=[.1, .2, .3, .4, .5, .6, .7, .8, .9],
                      method='median_unbiased')
orthomap2tei.get_bins(tobin_df=query_fst,
                      bincol='FST',
                      q=[.2, .4, .6, .8],
                      method='median_unbiased')
```

[8]:
```
         WormBase_ID  Chr      Gene  TajimaD  NormalizedPi   FayWu     FST  \
0      WBGene00000001    I     aap-1  -0.6957        0.0002 -1.2575  0.8062
1      WBGene00000002   IV     aat-1  -0.4724        0.0001 -1.4628  0.8846
2      WBGene00000003    V     aat-2  -1.5266        0.0001  0.0816  0.1691
3      WBGene00000004    X     aat-3  -1.6401        0.0003 -4.7685  0.8129
4      WBGene00000005   IV     aat-4  -1.2137        0.0006 -0.7617  0.3725
...               ...  ...       ...      ...           ...     ...     ...
```

```
20217  WBGene00271701    X   F10D7.10  -0.7428        0.0000 -1.9308  0.1111
20218  WBGene00271703  III  ZK1010.12  -1.3386        0.0008 -3.2528  0.7683
20219  WBGene00271706   II    D2089.8  -0.8312        0.0012  0.4489  0.5551
20220  WBGene00271707    V   ZK105.14  -1.0748        0.0004 -3.2069  0.6433
20221  WBGene00271715  III   B0244.17  -0.6617        0.0010 -1.1870  0.6556

       TajimaD_binned                         TajimaD_bins  \
0                 8.0                -0.84 >= x < -0.67368
1                 9.0  -0.67368 >= x < -0.20841999999999972
2                 3.0    -1.6187 >= x < -1.4602466666666667
3                 2.0               -1.8383 >= x < -1.6187
4                 5.0               -1.3196 >= x < -1.1656
...               ...                                  ...
20217             8.0                -0.84 >= x < -0.67368
20218             4.0    -1.4602466666666667 >= x < -1.3196
20219             8.0                -0.84 >= x < -0.67368
20220             6.0               -1.1656 >= x < -1.0416
20221             9.0  -0.67368 >= x < -0.20841999999999972

       NormalizedPi_binned     NormalizedPi_bins  FayWu_binned  \
0                      3.0  0.0002 >= x < 0.0004           5.0
1                      2.0  0.0001 >= x < 0.0002           5.0
2                      2.0  0.0001 >= x < 0.0002           8.0
3                      3.0  0.0002 >= x < 0.0004           2.0
4                      4.0  0.0004 >= x < 0.0011           6.0
...                    ...                   ...           ...
20217                  1.0            x < 0.0001           4.0
20218                  4.0  0.0004 >= x < 0.0011           3.0
20219                  5.0            0.0011 < x          10.0
20220                  4.0  0.0004 >= x < 0.0011           3.0
20221                  4.0  0.0004 >= x < 0.0011           5.0

                                            FayWu_bins  FST_binned  \
0                            -1.68402 >= x < -0.99135         5.0
1                            -1.68402 >= x < -0.99135         5.0
2      0.03219333333333343 >= x < 0.10182000000000008         3.0
3       -9.455993333333332 >= x < -3.9512999999999994         5.0
4                                 -0.99135 >= x < 0.0         4.0
...                                                ...         ...
20217                -2.277410000000001 >= x < -1.68402         2.0
20218   -3.9512999999999994 >= x < -2.277410000000001         5.0
20219                          0.2674900000000009 < x         4.0
20220   -3.9512999999999994 >= x < -2.277410000000001         5.0
20221                        -1.68402 >= x < -0.99135         5.0

                  FST_bins
0               0.6286 < x
1               0.6286 < x
2      0.1347 >= x < 0.3595
3               0.6286 < x
4      0.3595 >= x < 0.6286
...                    ...
20217     0.0 >= x < 0.1347
```

```
20218              0.6286 < x
20219  0.3595 >= x < 0.6286
20220              0.6286 < x
20221              0.6286 < x

[20222 rows x 15 columns]
```

1.8.3 Gene age assignments per query species lineage node

Given an orthomap, one can get an overview of the gene age assignments per query species lineage node.

The oggmap submodule of2orhomap and the of2orthomap.get_counts_per_ps() function will show the distribution of the gene age classes and can be further visualized as follows:

[9]:
```
# show count per taxonomic group (PStaxID)
of2orthomap.get_counts_per_ps(omap_df=query_orthomap,
    psnum_col='Phylostratum',
    pstaxid_col=None,
    psname_col=None)

# bar plot count per taxonomic group (PSname)
ax = of2orthomap.get_counts_per_ps(omap_df=query_orthomap,
    psnum_col='Phylostratum',
    pstaxid_col=None,
    psname_col=None).plot.bar(y='counts', x='Phylostratum')
ax.set_title('C. elegans - Number of genes per gene age class')
plt.show()
```

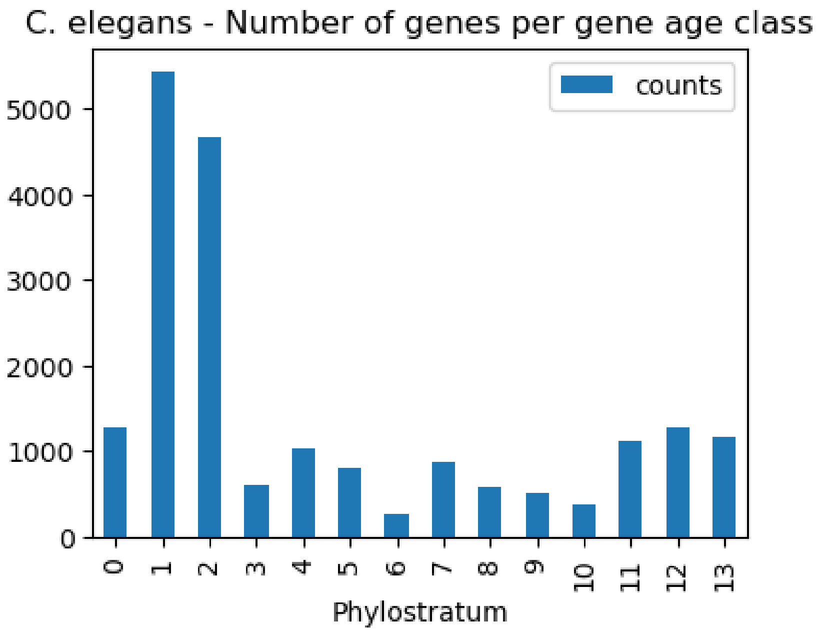

Gene assignments per query species evolutionary index

[10]:
```python
# show count per TajimaD group (TajimaD_binned)
of2orthomap.get_counts_per_ps(omap_df=query_fst,
    psnum_col='TajimaD_binned',
    pstaxid_col=None,
    psname_col=None)

# bar plot count per taxonomic group (PSname)
ax = of2orthomap.get_counts_per_ps(omap_df=query_fst,
    psnum_col='TajimaD_binned',
    pstaxid_col=None,
    psname_col=None).plot.bar(y='counts', x='TajimaD_binned')
ax.set_title('C. elegans - Number of genes per TajimaD class')
plt.show()
```

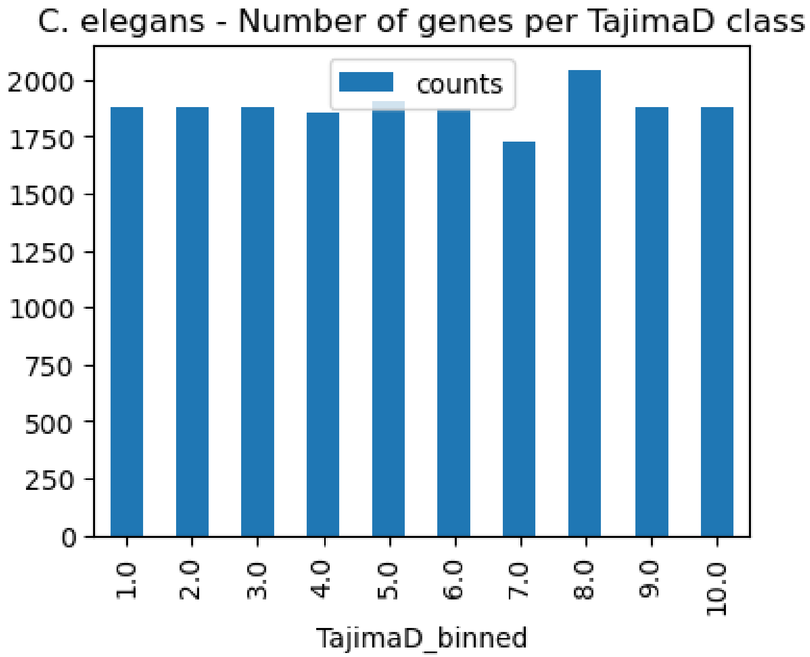

1.9 Step 3 - map OrthoFinder gene names and scRNA gene/transcript names

To be able to link gene ages assignments from an orthomap and gene or transcript of scRNA dataset, one needs to check the overlap of the annotated gene names. With the `gtf2t2g` submodule of `oggmap` and the `gtf2t2g.parse_gtf()` function, one can extract gene and transcript names from a given gene feature file (`GTF`).

Here, pre-calculated orthomap gene names already overlap, so no `GTF` import is necessary (Sun et al., 2021).

1.9.1 Import now, the scRNA dataset of the query species

Here, data is used, like in the original publication (Packer and Zhu al., 2019).

scRNA data were downloaded from https://www.ncbi.nlm.nih.gov/geo/query/acc.cgi?acc=GSE126954 converted into Seurat object and converted into loom and AnnData (h5ad) files to be able to analyse with e.g. python scanpy or `oggmap` package and is availbale here:

https://doi.org/10.5281/zenodo.7245547

or can be accessed with the `dataset` submodule of `oggmap`:

`datasets.packer19(datapath='data')` (download folder set to `'data'`).

Note: A smaller scRNA data set for the same data exist and can be obtained via:

`datasets.packer19_small(datapath='data')` (download folder set to `'data'`).

[11]:
```
# load scRNA data

# download zebrafish scRNA data here: https://doi.org/10.5281/zenodo.7245547
# or download with datasets.packer19('data')

#celegans_data = datasets.packer19(datapath='data')
celegans_data = sc.read('data/GSE126954.h5ad')
```

1.9.2 Get an overview of observations

[12]:
```
celegans_data
```

[12]:
```
AnnData object with n_obs × n_vars = 89701 × 20222
    obs: 'orig.ident', 'nCount_RNA', 'nFeature_RNA', 'cell', 'n.umi',
'time.point', 'batch', 'Size_Factor', 'cell.type', 'cell.subtype',
'plot.cell.type', 'raw.embryo.time', 'embryo.time', 'embryo.time.bin',
'raw.embryo.time.bin', 'lineage', 'passed_initial_QC_or_later_whitelisted'
    var: 'features', 'genes'
```

[13]:
```
celegans_data.obs
```

[13]:
```
                          orig.ident  nCount_RNA  nFeature_RNA  \
AAACCTGAGACAATAC-300.1.1           0      1630.0           781
AAACCTGAGGGCTCTC-300.1.1           0      2323.0          1116
AAACCTGAGTGCGTGA-300.1.1           0      3725.0          1322
AAACCTGAGTTGAGTA-300.1.1           0      4236.0          1747
AAACCTGCAAGACGTG-300.1.1           0      1003.0           621
...                              ...         ...           ...
TCTGAGACATGTCGAT-b02               0       581.0           361
TCTGAGACATGTCTCC-b02               0       516.0           327
TGGCCAGCACGAAGCA-b02               0       843.0           543
TGGCGCACAGGCAGTA-b02               0       634.0           397
TGGGCGTTCAGGCCCA-b02               0      1126.0           702

                                              cell  n.umi   time.point  \
AAACCTGAGACAATAC-300.1.1  AAACCTGAGACAATAC-300.1.1   1630  300_minutes
AAACCTGAGGGCTCTC-300.1.1  AAACCTGAGGGCTCTC-300.1.1   2319  300_minutes
AAACCTGAGTGCGTGA-300.1.1  AAACCTGAGTGCGTGA-300.1.1   3719  300_minutes
AAACCTGAGTTGAGTA-300.1.1  AAACCTGAGTTGAGTA-300.1.1   4251  300_minutes
AAACCTGCAAGACGTG-300.1.1  AAACCTGCAAGACGTG-300.1.1   1003  300_minutes
...                                            ...    ...          ...
TCTGAGACATGTCGAT-b02          TCTGAGACATGTCGAT-b02    585        mixed
TCTGAGACATGTCTCC-b02          TCTGAGACATGTCTCC-b02    510        mixed
TGGCCAGCACGAAGCA-b02          TGGCCAGCACGAAGCA-b02    843        mixed
TGGCGCACAGGCAGTA-b02          TGGCGCACAGGCAGTA-b02    636        mixed
```

```
TGGGCGTTCAGGCCCA-b02          TGGGCGTTCAGGCCCA-b02   1132        mixed

                                        batch  Size_Factor  \
AAACCTGAGACAATAC-300.1.1  Waterston_300_minutes     1.023195
AAACCTGAGGGCTCTC-300.1.1  Waterston_300_minutes     1.458210
AAACCTGAGTGCGTGA-300.1.1  Waterston_300_minutes     2.338283
AAACCTGAGTTGAGTA-300.1.1  Waterston_300_minutes     2.659051
AAACCTGCAAGACGTG-300.1.1  Waterston_300_minutes     0.629610
...                                         ...          ...
TCTGAGACATGTCGAT-b02                 Murray_b02     0.364709
TCTGAGACATGTCTCC-b02                 Murray_b02     0.323907
TGGCCAGCACGAAGCA-b02                 Murray_b02     0.529174
TGGCGCACAGGCAGTA-b02                 Murray_b02     0.397979
TGGGCGTTCAGGCCCA-b02                 Murray_b02     0.706820

                                       cell.type    cell.subtype  \
AAACCTGAGACAATAC-300.1.1        Body_wall_muscle  BWM_head_row_1
AAACCTGAGGGCTCTC-300.1.1                      NA              NA
AAACCTGAGTGCGTGA-300.1.1                      NA              NA
AAACCTGAGTTGAGTA-300.1.1        Body_wall_muscle    BWM_anterior
AAACCTGCAAGACGTG-300.1.1  Ciliated_amphid_neuron             AFD
...                                          ...             ...
TCTGAGACATGTCGAT-b02                 Rectal_gland    Rectal_gland
TCTGAGACATGTCTCC-b02                          NA              NA
TGGCCAGCACGAAGCA-b02                          NA              NA
TGGCGCACAGGCAGTA-b02                          NA              NA
TGGGCGTTCAGGCCCA-b02                          NA              NA

                          plot.cell.type  raw.embryo.time  embryo.time  \
AAACCTGAGACAATAC-300.1.1  BWM_head_row_1              360        380.0
AAACCTGAGGGCTCTC-300.1.1              NA              260        220.0
AAACCTGAGTGCGTGA-300.1.1              NA              270        230.0
AAACCTGAGTTGAGTA-300.1.1    BWM_anterior              260        280.0
AAACCTGCAAGACGTG-300.1.1             AFD              350        350.0
...                                  ...              ...          ...
TCTGAGACATGTCGAT-b02        Rectal_gland              390        700.0
TCTGAGACATGTCTCC-b02                  NA              510        470.0
TGGCCAGCACGAAGCA-b02                  NA              400        470.0
TGGCGCACAGGCAGTA-b02                  NA              330        350.0
TGGGCGTTCAGGCCCA-b02                  NA              260        265.0

                         embryo.time.bin raw.embryo.time.bin  \
AAACCTGAGACAATAC-300.1.1         330-390             330-390
AAACCTGAGGGCTCTC-300.1.1         210-270             210-270
AAACCTGAGTGCGTGA-300.1.1         210-270             270-330
AAACCTGAGTTGAGTA-300.1.1         270-330             210-270
AAACCTGCAAGACGTG-300.1.1         330-390             330-390
...                                  ...                 ...
TCTGAGACATGTCGAT-b02               > 650             390-450
TCTGAGACATGTCTCC-b02             450-510             510-580
TGGCCAGCACGAAGCA-b02             450-510             390-450
TGGCGCACAGGCAGTA-b02             330-390             330-390
TGGGCGTTCAGGCCCA-b02             210-270             210-270
```

```
                                                    lineage  \
AAACCTGAGACAATAC-300.1.1                    MSxpappp
AAACCTGAGGGCTCTC-300.1.1                    MSxapaap
AAACCTGAGTGCGTGA-300.1.1                          NA
AAACCTGAGTTGAGTA-300.1.1                        Dxap
AAACCTGCAAGACGTG-300.1.1  ABalpppapav/ABpraaaapav
...                                              ...
TCTGAGACATGTCGAT-b02                              NA
TCTGAGACATGTCTCC-b02                              NA
TGGCCAGCACGAAGCA-b02                              NA
TGGCGCACAGGCAGTA-b02                              NA
TGGGCGTTCAGGCCCA-b02                              NA

                          passed_initial_QC_or_later_whitelisted
AAACCTGAGACAATAC-300.1.1                                       1
AAACCTGAGGGCTCTC-300.1.1                                       1
AAACCTGAGTGCGTGA-300.1.1                                       1
AAACCTGAGTTGAGTA-300.1.1                                       1
AAACCTGCAAGACGTG-300.1.1                                       1
...                                                          ...
TCTGAGACATGTCGAT-b02                                           1
TCTGAGACATGTCTCC-b02                                           1
TGGCCAGCACGAAGCA-b02                                           1
TGGCGCACAGGCAGTA-b02                                           1
TGGGCGTTCAGGCCCA-b02                                           1

[89701 rows x 17 columns]
```

[14]:
```
celegans_data.obs.dtypes
```

[14]:
```
orig.ident                                  int32
nCount_RNA                                float64
nFeature_RNA                                int32
cell                                       object
n.umi                                       int32
time.point                               category
batch                                    category
Size_Factor                               float64
cell.type                                category
cell.subtype                             category
plot.cell.type                           category
raw.embryo.time                             int32
embryo.time                               float64
embryo.time.bin                          category
raw.embryo.time.bin                      category
lineage                                  category
passed_initial_QC_or_later_whitelisted      int32
dtype: object
```

Prior any analysis the observations `'embryo.time.bin'` and `'batch'` will be converted into the `'category'` type. In addition a new observation `'cell.type.per.embryo.time.bin.cat'` will be created that combines sample timepoint and assigned cell type.

[15]:
```python
# add embryo.time.bin as category
celegans_data.obs['embryo.time.bin.cat'] =\
    celegans_data.obs['embryo.time.bin'].astype('category')
celegans_data.obs['embryo.time.bin.cat'] =\
    celegans_data.obs['embryo.time.bin.cat'].cat.reorder_categories(['< 100',\
    '100-130','130-170','170-210','210-270','270-330','330-390','390-450','450-510',\
    '510-580','580-650','> 650'])
celegans_data.obs['batch.cat'] = celegans_data.obs['batch'].astype('category')
```

[16]:
```python
celegans_data.obs['cell.type.per.embryo.time.bin.cat'] =\
    (celegans_data.obs['cell.type'].astype('string') +\
    '-' +\
    celegans_data.obs['embryo.time.bin.cat'].astype('string')).astype('category')
```

1.9.3 Helper functions to match gene names

The `orthomap2tei` submodule contains the `orthomap2tei.geneset_overlap()` helper function to check for gene name overlap between the constructed orthomap from `OrthoFinder` results and a given scRNA dataset.

[17]:
```python
# check overlap of orthomap <seqID> and scRNA data <var_names>
orthomap2tei.geneset_overlap(geneset1=celegans_data.var_names,
                             geneset2=query_orthomap['GeneID'])
```

[17]:

	g1_g2_overlap	g1_ratio	g2_ratio
0	19795	0.978884	0.987774

1.10 step 4 - Get TEI values and add them to scRNA dataset

Since now the gene names correspond to each other in the orthomap and the scRNA `adata` object, one can calculate the transcriptome evolutionary index (`TEI`) and add them to the scRNA dataset (`adata` object).

The `TEI` measure represents the weighted arithmetic mean (expression levels as weights for the phylostratum value) over all evolutionary age categories denoted as *phylostra*.

$TEI_s = \sum(e_{is} * ps_i) / \sum e_{is}$

, where TEI_s denotes the `TEI` value in developmental stage s, e_{is} denotes the gene expression level of gene i in stage s, and ps_i denotes the corresponding phylostratum of gene $i, i = 1, ..., N$ and $N = total\ number\ of\ genes$.

Note: If e.g. two different isoforms would fall into two different gene age classes, their gene ages might differ based on the oldest ortholog found in their corresponding orthologous groups. However, both isoforms share the same gene name and their gene ages would clash. In this case one can decide either to use the `keep='min'` or `keep='max'` gene age to be kept by the `get_tei` function, which defaults to keep in this cases the `keep='min'` or in other words the 'older' gene age.

To be able to re-use the original `count` data, they are added as a new `layer` to the `adata` object. This is useful because later on the `count` data can be used to extract either the relative expression per gene age class.

This can be done either on un-normalized `counts`, on `normalized` and `log-transformed` data.

[18]:
```python
celegans_data.layers['counts'] = celegans_data.X
```

1.10.1 add TEI to adata object

Using the submodule `orthomap2tei` from `oggmap` and the `orthomap2tei.get_tei()` function, transcriptome evolutionary index (`TEI`) values are calculated and directyl added to the existing `adata` object (`add_obs=True`).

There are other options to e.g. not start from the `adata.X counts` but from another `layer` from the `adata` object, the default is to use the `adata.X` (`layer=None`). The values can be pre-processed by the `normalize_total` option and the `log1p` option.

If `add_obs=True` the resulting `TEI` values are added to the existing `adata` object as a new observation with the name set with the `obs_name` option.

If `add_var=True` the gene age values are added to the existing `adata` object as a new variable with the name set with the `var_name` option.

Note: Genes not assigned to any gene class will get a missing assignment.

If one wants to calculate bootstrap `TEI` values per cell, the `boot` option can be set to `boot=True` and gene age classes will be randomly chosen prior calculating `TEI` values `bt=10` times.

[19]:
```
# add TEI values to existing adata object
orthomap2tei.get_tei(adata=celegans_data,
    gene_id=query_orthomap['GeneID'],
    gene_age=query_orthomap['Phylostratum'],
    keep='min',
    layer=None,
    add_var=True,
    var_name='Phylostrata',
    add_obs=True,
    obs_name='tei',
    boot=False,
    bt=10,
    normalize_total=True,
    log1p=True,
    target_sum=1e6)
```

[19]:
```
                                tei
AAACCTGAGACAATAC-300.1.1   1.876533
AAACCTGAGGGCTCTC-300.1.1   2.239562
AAACCTGAGTGCGTGA-300.1.1   2.126684
AAACCTGAGTTGAGTA-300.1.1   2.362362
AAACCTGCAAGACGTG-300.1.1   2.581323
...                             ...
TCTGAGACATGTCGAT-b02       1.964531
TCTGAGACATGTCTCC-b02       2.155930
TGGCCAGCACGAAGCA-b02       2.262958
TGGCGCACAGGCAGTA-b02       2.565133
TGGGCGTTCAGGCCCA-b02       2.269434

[89701 rows x 1 columns]
```

1.10.2 add TajimaD, Fst and NormalizedPi to adata object

[20]:
```python
# add TajimaD binned values to existing adata object
orthomap2tei.get_tei(adata=celegans_data,
    gene_id=query_fst['WormBase_ID'],
    gene_age=query_fst['TajimaD_binned'],
    keep='min',
    layer=None,
    add_var=True,
    var_name='TajimaD_bin',
    add_obs=True,
    obs_name='TajimaD',
    boot=False,
    bt=10,
    normalize_total=True,
    log1p=True,
    target_sum=1e6)
```

[20]:
```
                              TajimaD
AAACCTGAGACAATAC-300.1.1  5.769133
AAACCTGAGGGCTCTC-300.1.1  5.898489
AAACCTGAGTGCGTGA-300.1.1  5.736505
AAACCTGAGTTGAGTA-300.1.1  5.795881
AAACCTGCAAGACGTG-300.1.1  5.746057
...                            ...
TCTGAGACATGTCGAT-b02      5.465485
TCTGAGACATGTCTCC-b02      5.543912
TGGCCAGCACGAAGCA-b02      5.524013
TGGCGCACAGGCAGTA-b02      5.652469
TGGGCGTTCAGGCCCA-b02      5.634361

[89701 rows x 1 columns]
```

[21]:
```python
# add Fst binned values to existing adata object
orthomap2tei.get_tei(adata=celegans_data,
    gene_id=query_fst['WormBase_ID'],
    gene_age=query_fst['FST_binned'],
    keep='min',
    layer=None,
    add_var=True,
    var_name='FST_bin',
    add_obs=True,
    obs_name='Fst',
    boot=False,
    bt=10,
    normalize_total=True,
    log1p=True,
    target_sum=1e6)
```

[21]:
```
                                Fst
AAACCTGAGACAATAC-300.1.1  2.997840
AAACCTGAGGGCTCTC-300.1.1  3.103704
AAACCTGAGTGCGTGA-300.1.1  3.100244
```

```
AAACCTGAGTTGAGTA-300.1.1  3.105081
AAACCTGCAAGACGTG-300.1.1  3.011721
...                            ...
TCTGAGACATGTCGAT-b02      3.011302
TCTGAGACATGTCTCC-b02      3.134352
TGGCCAGCACGAAGCA-b02      3.054067
TGGCGCACAGGCAGTA-b02      3.117862
TGGGCGTTCAGGCCCA-b02      3.149005

[89701 rows x 1 columns]
```

[22]:

```
# add NormalizedPi binned values to existing adata object
orthomap2tei.get_tei(adata=celegans_data,
    gene_id=query_fst['WormBase_ID'],
    gene_age=query_fst['NormalizedPi_binned'],
    keep='min',
    layer=None,
    add_var=True,
    var_name='NormalizedPi_bin',
    add_obs=True,
    obs_name='NormalizedPi',
    boot=False,
    bt=10,
    normalize_total=True,
    log1p=True,
    target_sum=1e6)
```

[22]:

```
                          NormalizedPi
AAACCTGAGACAATAC-300.1.1      2.321107
AAACCTGAGGGCTCTC-300.1.1      2.527517
AAACCTGAGTGCGTGA-300.1.1      2.445354
AAACCTGAGTTGAGTA-300.1.1      2.507382
AAACCTGCAAGACGTG-300.1.1      2.655676
...                                ...
TCTGAGACATGTCGAT-b02          2.267350
TCTGAGACATGTCTCC-b02          2.484896
TGGCCAGCACGAAGCA-b02          2.378922
TGGCGCACAGGCAGTA-b02          2.483049
TGGGCGTTCAGGCCCA-b02          2.470968

[89701 rows x 1 columns]
```

1.11 Step 5 - downstream analysis

Once the gene age data has been added to the scRNA dataset, one can e.g. plot the corresponding transcriptome evolutionary index (`TEI`) values by any given observation pre-defined in the scRNA dataset.

Here, we plot them against the assigned sample timepoint and against assigned cell types of the nematode using the `scanpy` `sc.pl.violin()` function as follows:

1.11.1 Boxplot gene age class per sample timepoint

[23]:
```
ax = sc.pl.violin(adata=celegans_data,
                  keys=['tei'],
                  groupby='embryo.time.bin.cat',
                  rotation=90,
                  palette='Paired',
                  stripplot=False,
                  inner='box',
                  order=['< 100', '100-130', '130-170', '170-210',
                         '210-270', '270-330', '330-390', '450-510',
                         '510-580', '580-650', '> 650'],
                  show=False)
ax.set_title('C. elegans - TEI distribution per embryo time')
plt.show()
```

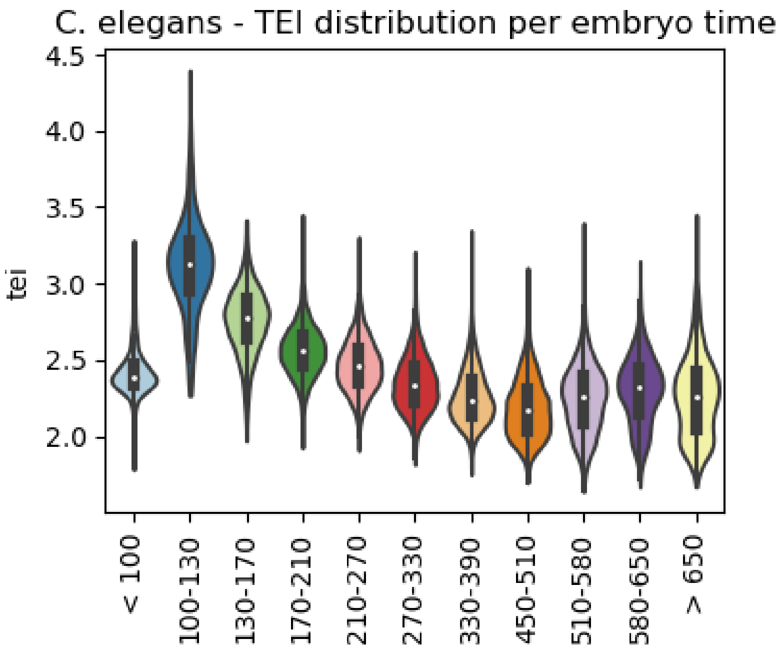

1.11.2 Boxplot TajimaD class per sample timepoint

[24]:
```
ax = sc.pl.violin(adata=celegans_data,
                  keys=['TajimaD'],
                  groupby='embryo.time.bin.cat',
                  rotation=90,
                  palette='Paired',
                  stripplot=False,
                  inner='box',
                  order=['< 100', '100-130', '130-170', '170-210',
                         '210-270', '270-330', '330-390', '450-510',
                         '510-580', '580-650', '> 650'],
                  show=False)
```

```
ax.set_title('C. elegans - TajimaD distribution per embryo time')
plt.show()
```

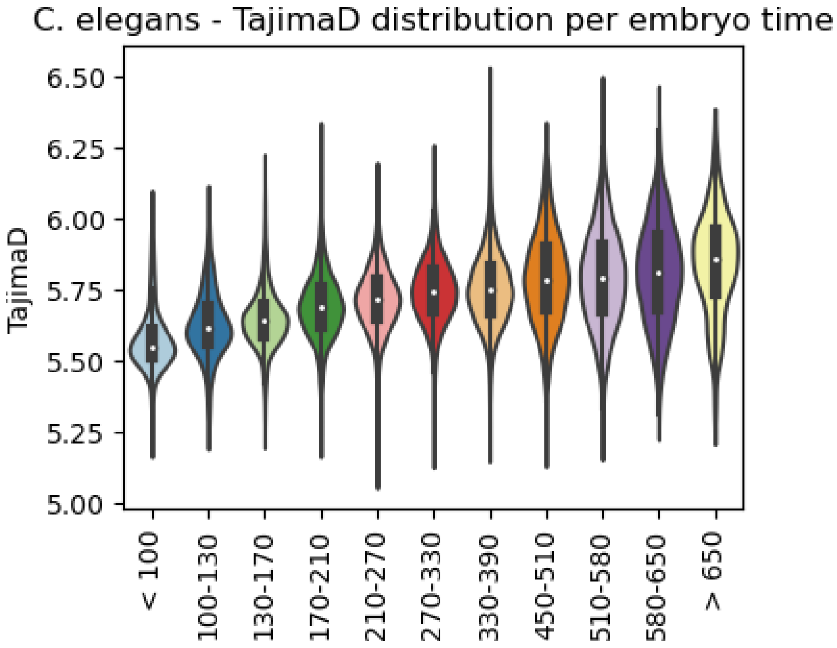

1.11.3 Boxplot Fst class per sample timepoint

[25]:
```
ax = sc.pl.violin(adata=celegans_data,
                  keys=['Fst'],
                  groupby='embryo.time.bin.cat',
                  rotation=90,
                  palette='Paired',
                  stripplot=False,
                  inner='box',
                  order=['< 100', '100-130', '130-170', '170-210',
                         '210-270', '270-330', '330-390', '450-510',
                         '510-580', '580-650', '> 650'],
                  show=False)
ax.set_title('C. elegans - Fst distribution per embryo time')
plt.show()
```

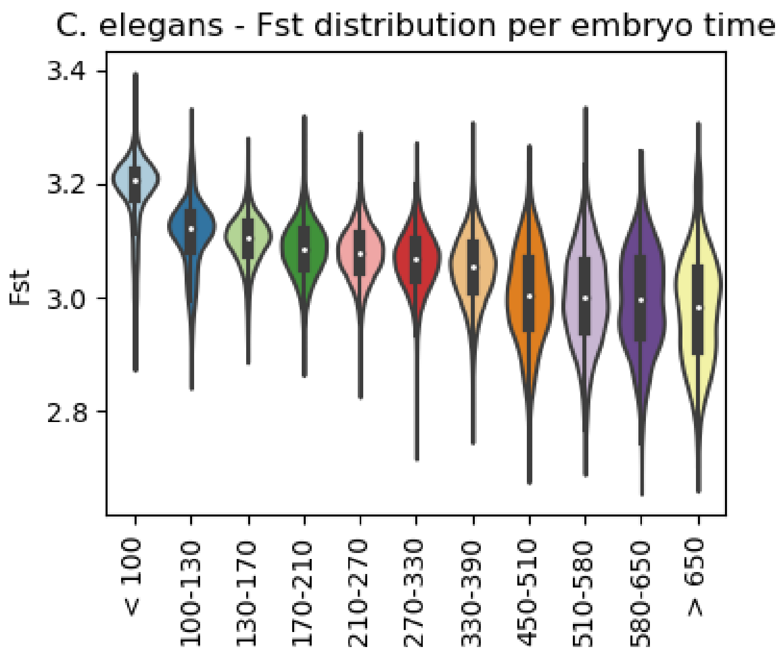

1.11.4 Boxplot NormalizedPi class per sample timepoint

[26]:
```
ax = sc.pl.violin(adata=celegans_data,
                  keys=['NormalizedPi'],
                  groupby='embryo.time.bin.cat',
                  rotation=90,
                  palette='Paired',
                  stripplot=False,
                  inner='box',
                  order=['< 100', '100-130', '130-170', '170-210',
                         '210-270', '270-330', '330-390', '450-510',
                         '510-580', '580-650', '> 650'],
                  show=False)
ax.set_title('C. elegans - NormalizedPi distribution per embryo time')
plt.show()
```

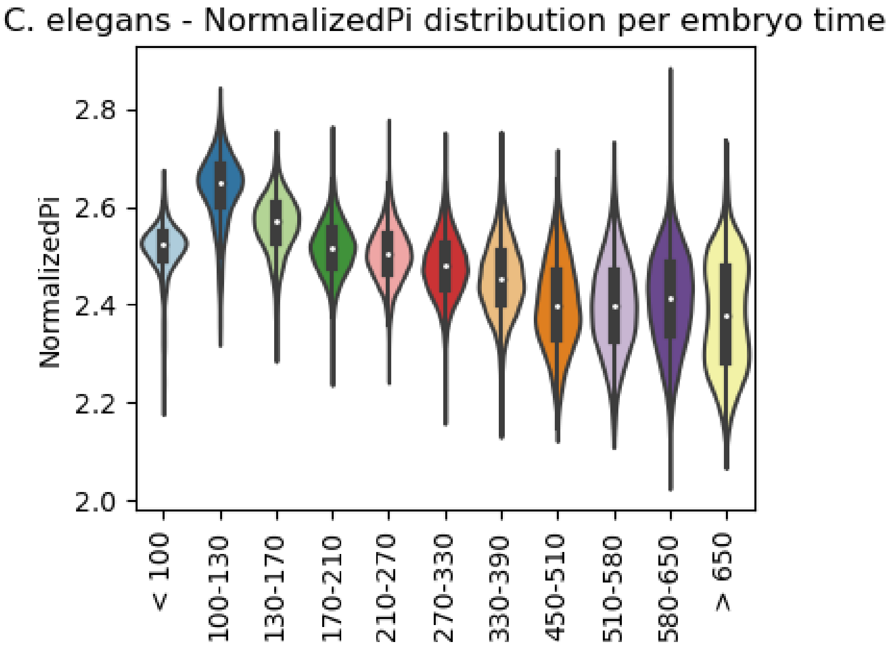

1.11.5 Boxplot gene age class per sample timepoint and add significance

Note: Please change notebook cell from `raw` to `code` to see the plots.

```
ax = sns.boxplot(
    x='embryo.time.bin.cat',
    y='tei',
    data=celegans_data.obs)
ax.set_title('C. elegans - TEI distribution per embryo time')
test_results = add_stat_annotation(
    ax,
    x='embryo.time.bin.cat',
    y='tei',
    data=celegans_data.obs,
    box_pairs=orthomap2tei._get_pairwise_comb_self(
        list1=celegans_data.obs['embryo.time.bin.cat'].value_counts().index),
        test='Mann-Whitney',
        text_format='star',
        loc='outside',
    verbose=0)
plt.xticks(rotation=90)
plt.show()
```

1.11.6 Boxplot gene age class per sample timepoint and per cell type

E.g. to just show the same plot for a selected cell-type, one could do the following.

1. List all annotated cell types:

[27]:
```
list(set(celegans_data.obs['cell.type']))
```

[27]:
```
['Excretory_duct_and_pore',
 'Parent_of_exc_gland_AVK',
 'Pharyngeal_intestinal_valve',
 'M_cell',
 'Pharyngeal_muscle',
 'hmc',
 'T',
 'Coelomocyte',
 'Z1_Z4',
 'Seam_cell',
 'G2_and_W_blasts',
 'Body_wall_muscle',
 'Hypodermis',
 'Pharyngeal_neuron',
 'Pharyngeal_gland',
 'hmc_homolog',
 'Intestinal_and_rectal_muscle',
 'Ciliated_non_amphid_neuron',
 'Excretory_cell_parent',
 'ABarpaaa_lineage',
 'Arcade_cell',
 'Excretory_gland',
 'XXX',
 'Pharyngeal_marginal_cell',
 'Excretory_cell',
 'hmc_and_homolog',
 'NA',
 'GLR',
 'Germline',
 'Parent_of_exc_duct_pore_DB_1_3',
 'hyp1V_and_ant_arc_V',
 'Glia',
 'Intestine',
 'Ciliated_amphid_neuron',
 'Rectal_cell',
 'Rectal_gland',
 'Parent_of_hyp1V_and_ant_arc_V']
```

2. Loop over all cell types:

Note: Please change notebook cell from `raw` to `code` to see the plots.

[28]:
```
for ct in list(set(celegans_data.obs['cell.type'])):
    ax = sc.pl.violin(adata=celegans_data[celegans_data.obs['cell.type']==ct],
                      keys=['tei'],
                      groupby='embryo.time.bin.cat',
                      rotation=90,
                      palette='Paired',
                      stripplot=False,
                      inner='box',
                      order=['< 100', '100-130', '130-170', '170-210',
                             '210-270', '270-330', '330-390', '450-510',
                             '510-580', '580-650', '> 650'],
                      show=False)
```

```
    ax.set_title(ct)
    ax.set_xlabel('embryo.time.bin.cat')
    plt.show()
```

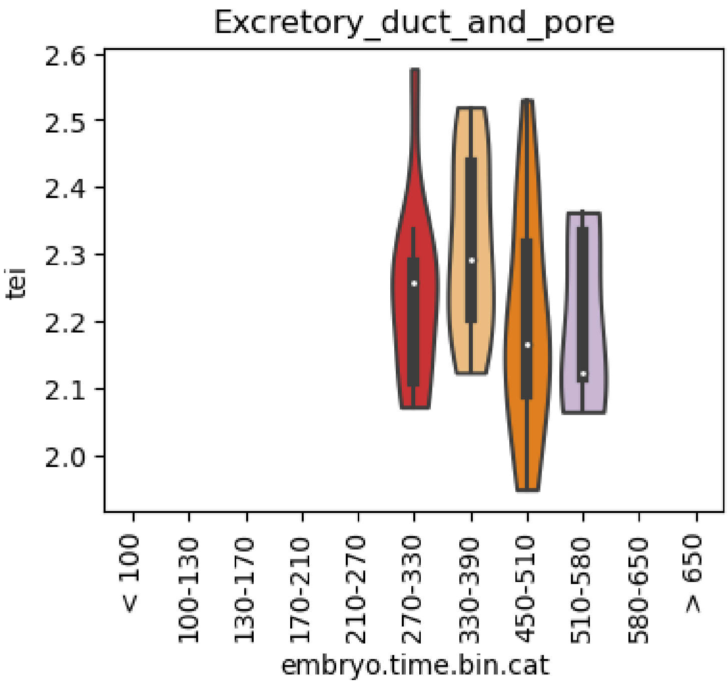

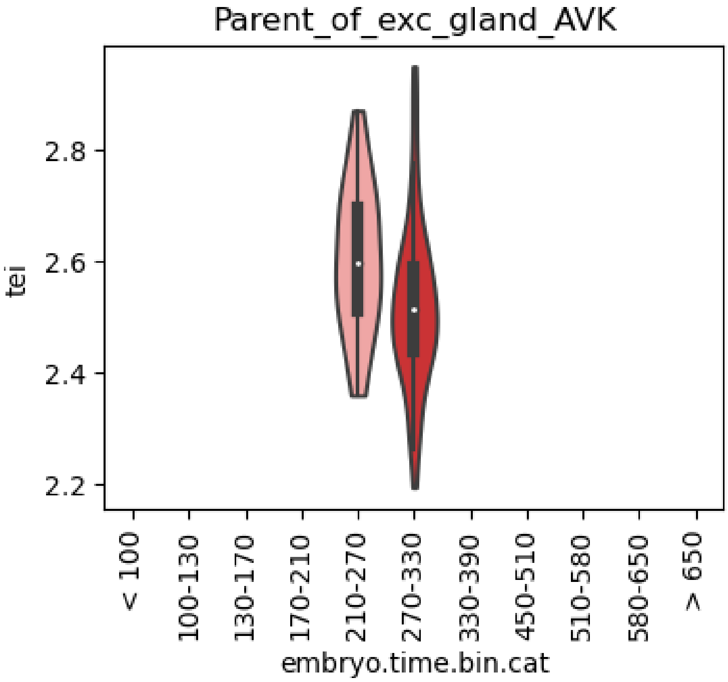

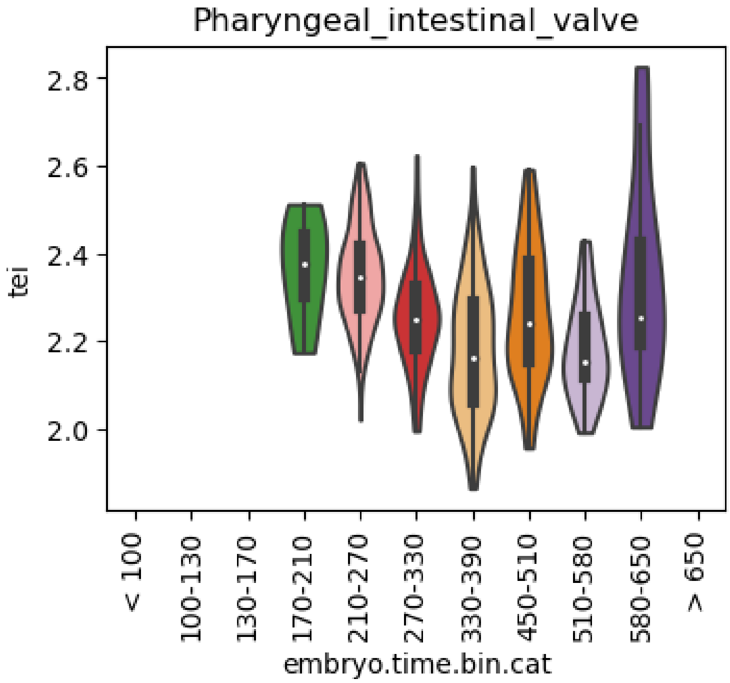

Pharyngeal_intestinal_valve
2.8
2.6
2.4
2.2
2.0
tei
< 100
100-130
130-170
170-210
210-270
270-330
330-390
450-510
510-580
580-650
> 650
embryo.time.bin.cat

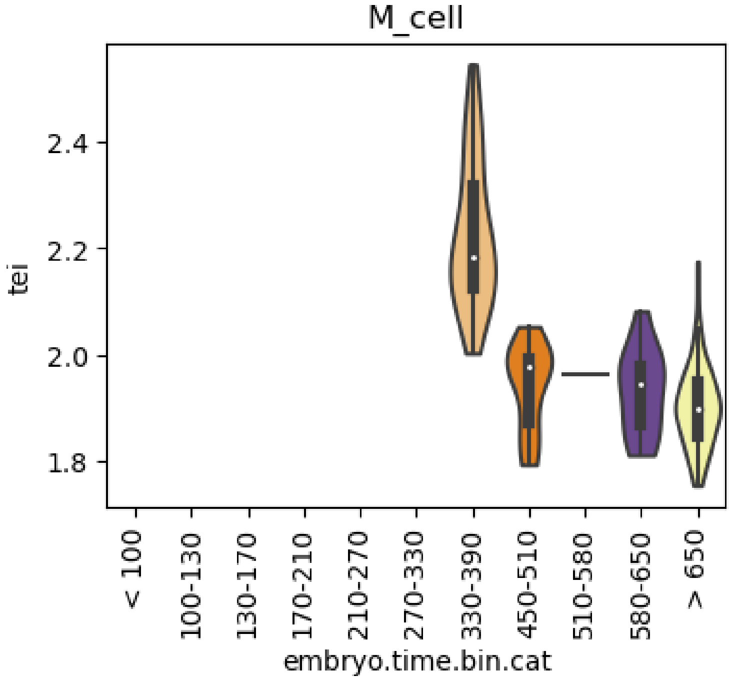

M_cell
2.4
2.2
2.0
1.8
tei
< 100
100-130
130-170
170-210
210-270
270-330
330-390
450-510
510-580
580-650
> 650
embryo.time.bin.cat

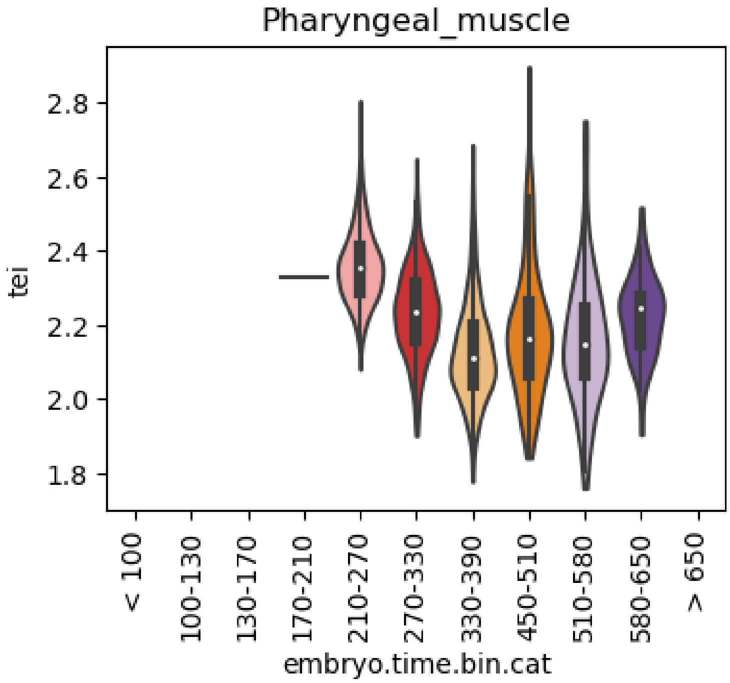
Pharyngeal_muscle
2.8
2.6
2.4
2.2
2.0
1.8
tei
< 100
100-130
130-170
170-210
210-270
270-330
330-390
450-510
510-580
580-650
> 650
embryo.time.bin.cat

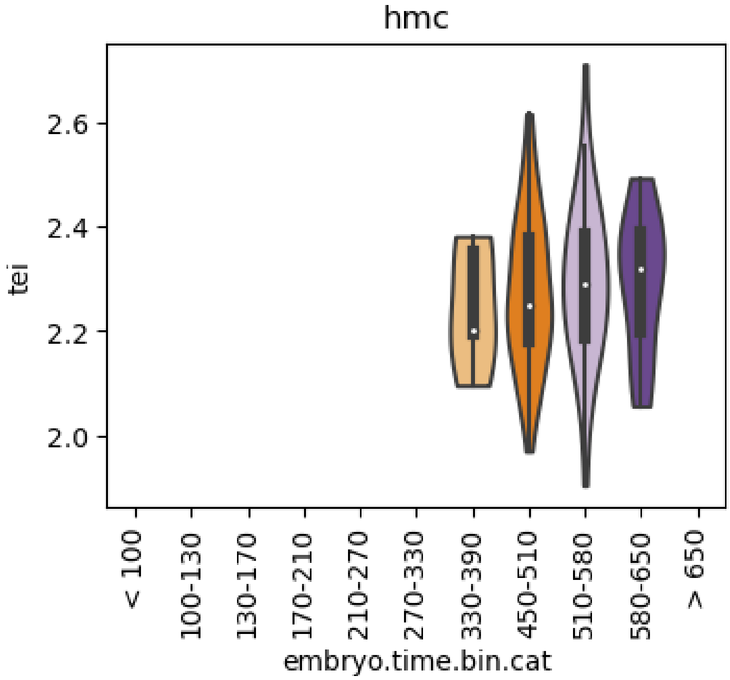
hmc
2.6
2.4
2.2
2.0
tei
< 100
100-130
130-170
170-210
210-270
270-330
330-390
450-510
510-580
580-650
> 650
embryo.time.bin.cat

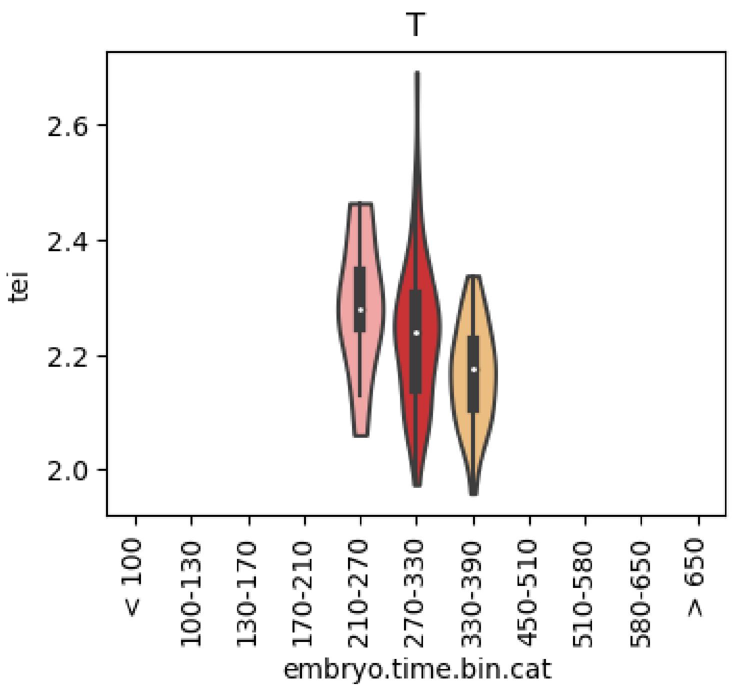
T
2.6
2.4
2.2
2.0
tei
< 100
100-130
130-170
170-210
210-270
270-330
330-390
450-510
510-580
580-650
> 650
embryo.time.bin.cat

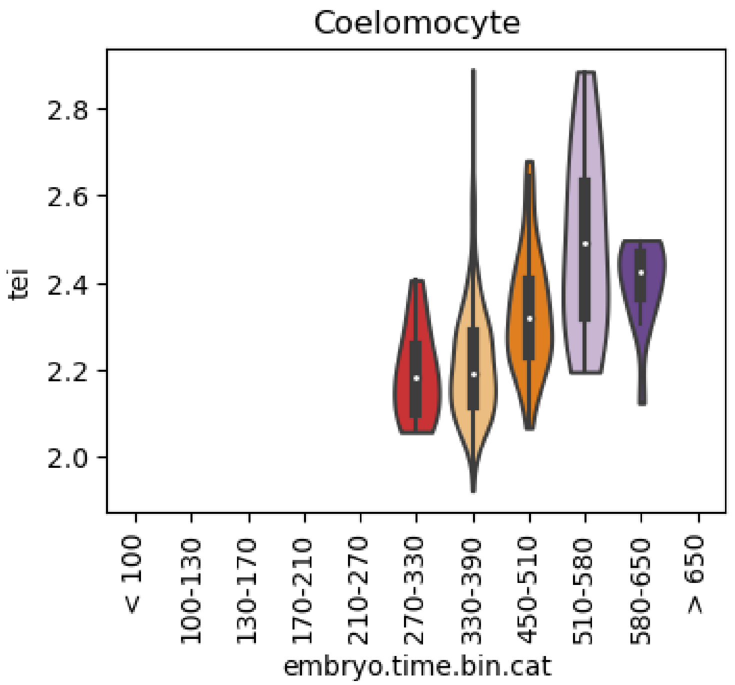
Coelomocyte
2.8
2.6
2.4
2.2
2.0
tei
< 100
100-130
130-170
170-210
210-270
270-330
330-390
450-510
510-580
580-650
> 650
embryo.time.bin.cat

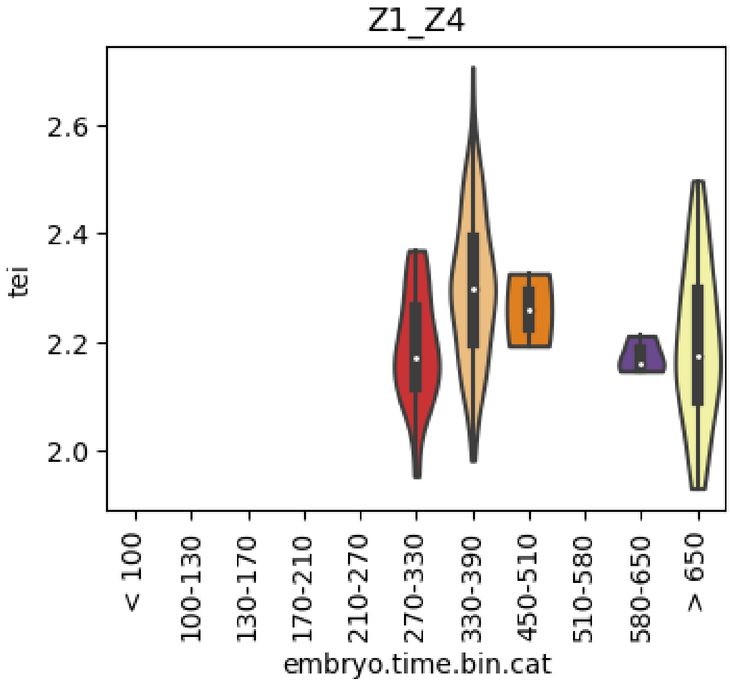
Z1_Z4
tei
2.0
2.2
2.4
2.6
< 100
100-130
130-170
170-210
210-270
270-330
330-390
450-510
510-580
580-650
> 650
embryo.time.bin.cat

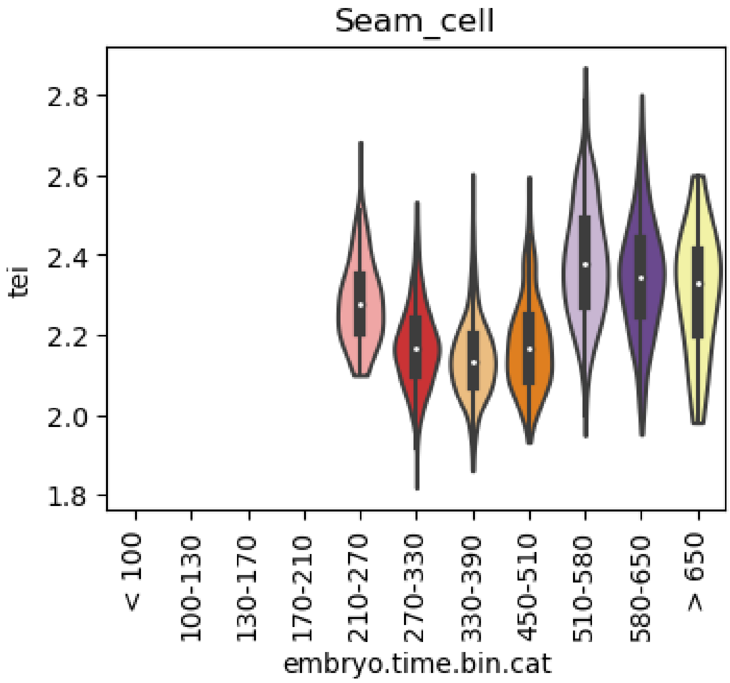
Seam_cell
tei
1.8
2.0
2.2
2.4
2.6
2.8
< 100
100-130
130-170
170-210
210-270
270-330
330-390
450-510
510-580
580-650
> 650
embryo.time.bin.cat

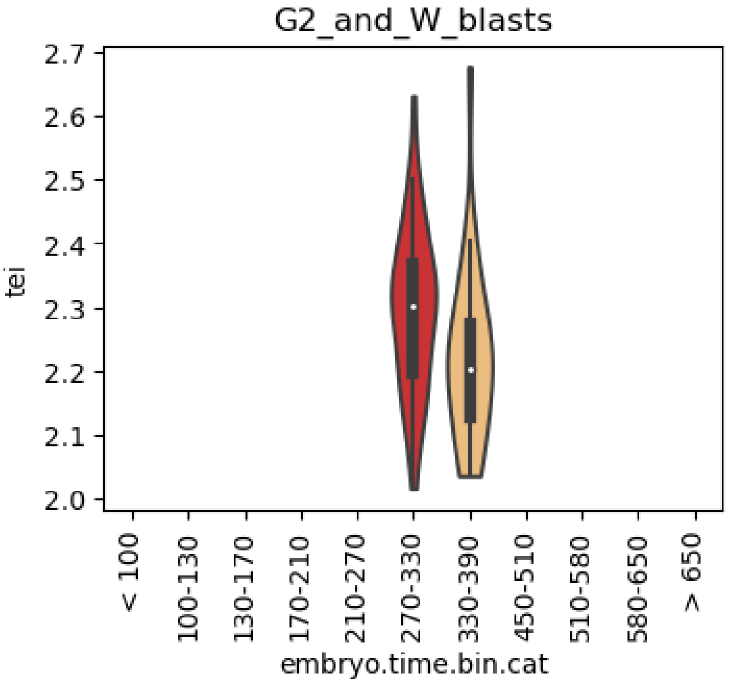

G2_and_W_blasts
tei
2.7
2.6
2.5
2.4
2.3
2.2
2.1
2.0
< 100
100-130
130-170
170-210
210-270
270-330
330-390
450-510
510-580
580-650
> 650
embryo.time.bin.cat

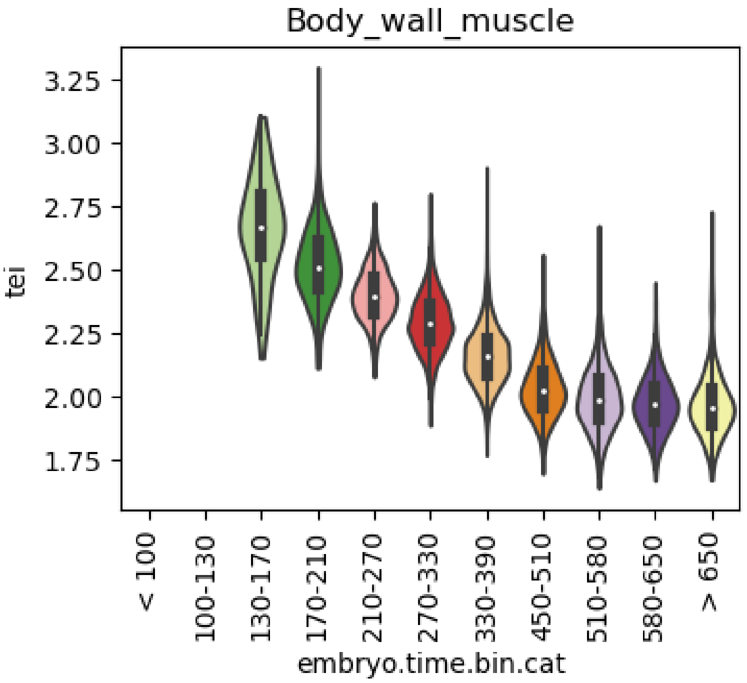

Body_wall_muscle
tei
3.25
3.00
2.75
2.50
2.25
2.00
1.75
< 100
100-130
130-170
170-210
210-270
270-330
330-390
450-510
510-580
580-650
> 650
embryo.time.bin.cat

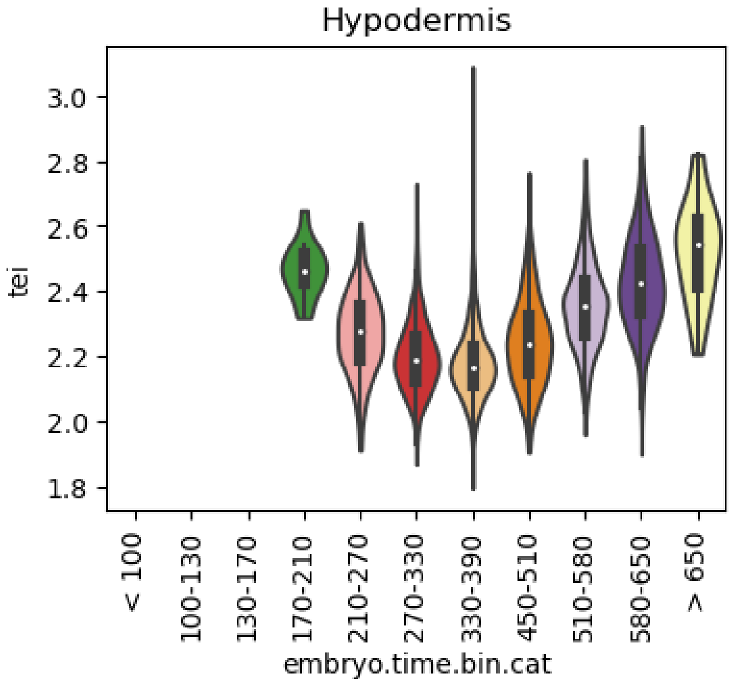

Hypodermis
3.0
2.8
2.6
2.4
2.2
2.0
1.8
tei
< 100
100-130
130-170
170-210
210-270
270-330
330-390
450-510
510-580
580-650
> 650
embryo.time.bin.cat

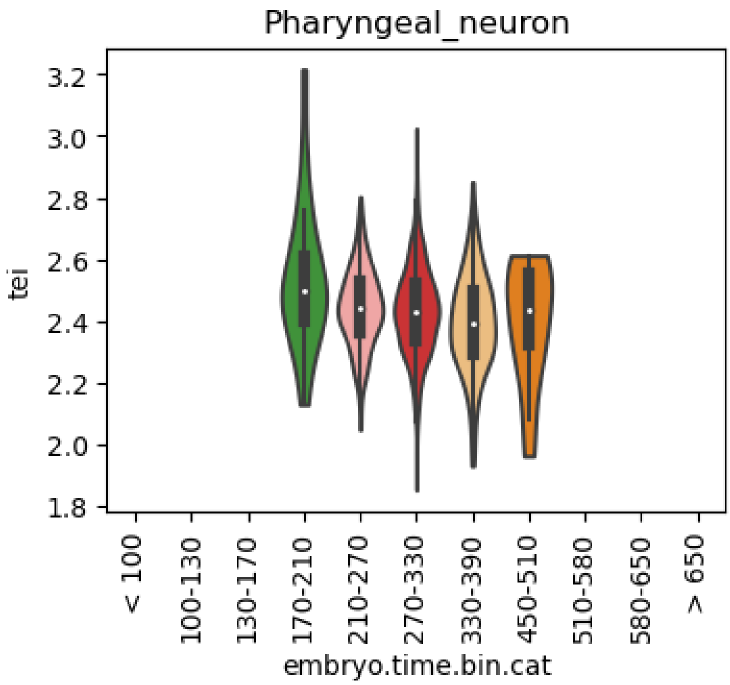

Pharyngeal_neuron
3.2
3.0
2.8
2.6
2.4
2.2
2.0
1.8
tei
< 100
100-130
130-170
170-210
210-270
270-330
330-390
450-510
510-580
580-650
> 650
embryo.time.bin.cat

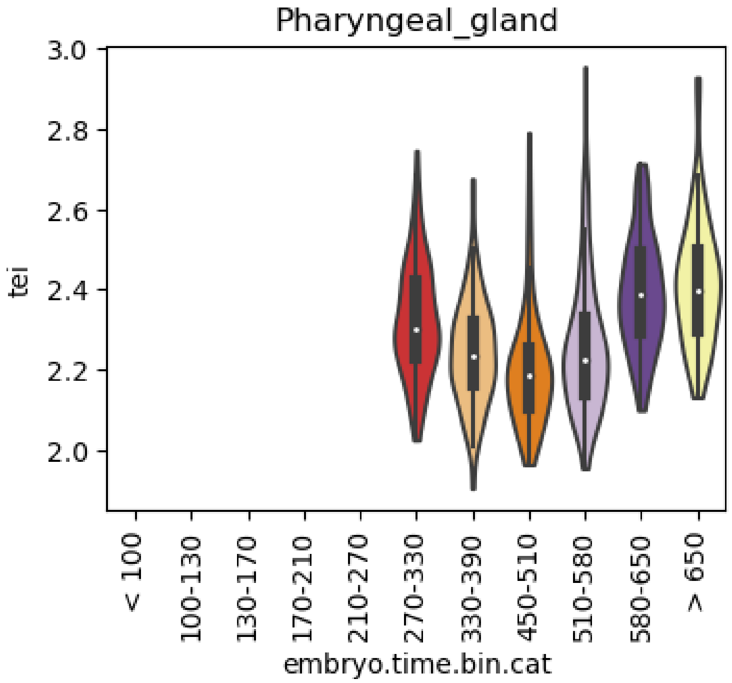
Pharyngeal_gland
tei
3.0
2.8
2.6
2.4
2.2
2.0
< 100
100-130
130-170
170-210
210-270
270-330
330-390
450-510
510-580
580-650
> 650
embryo.time.bin.cat

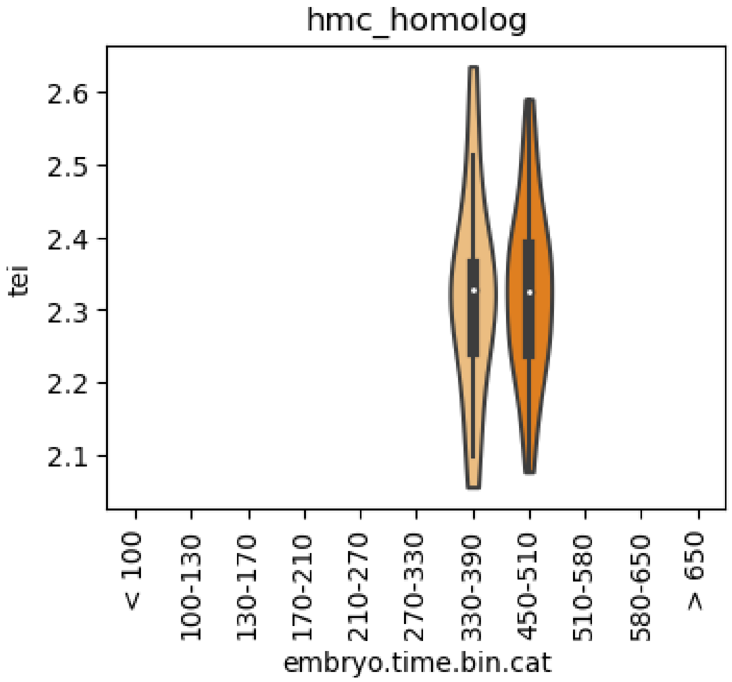
hmc_homolog
tei
2.6
2.5
2.4
2.3
2.2
2.1
< 100
100-130
130-170
170-210
210-270
270-330
330-390
450-510
510-580
580-650
> 650
embryo.time.bin.cat

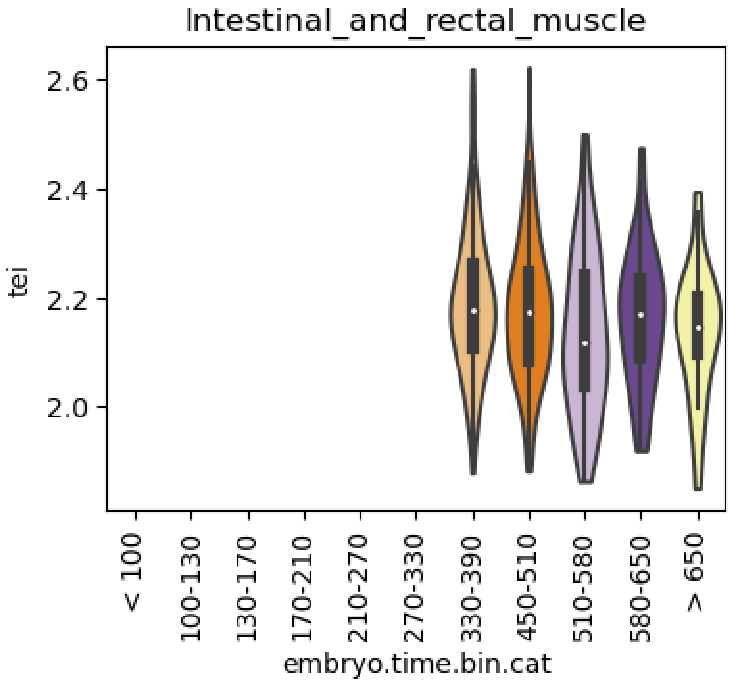
Intestinal_and_rectal_muscle
2.6
2.4
2.2
2.0
tei
< 100
100-130
130-170
170-210
210-270
270-330
330-390
450-510
510-580
580-650
> 650
embryo.time.bin.cat

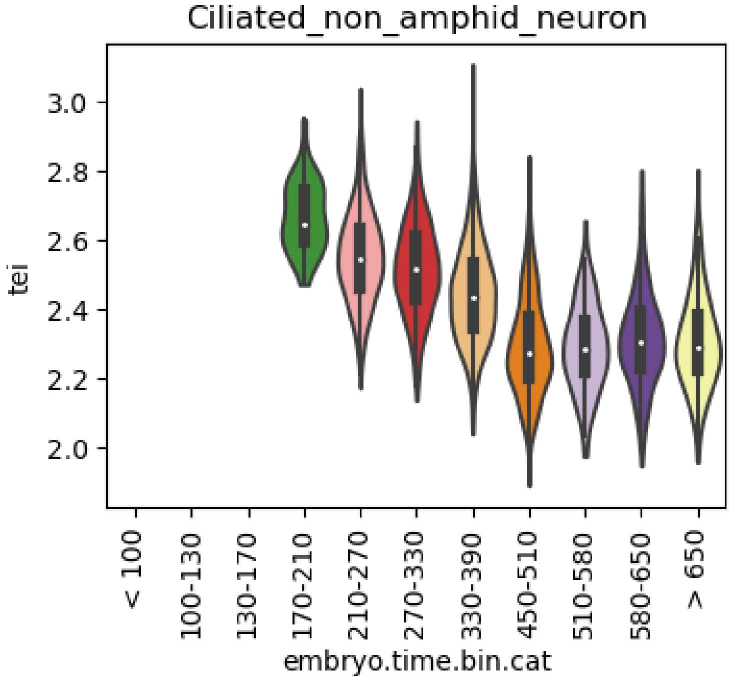
Ciliated_non_amphid_neuron
3.0
2.8
2.6
2.4
2.2
2.0
tei
< 100
100-130
130-170
170-210
210-270
270-330
330-390
450-510
510-580
580-650
> 650
embryo.time.bin.cat

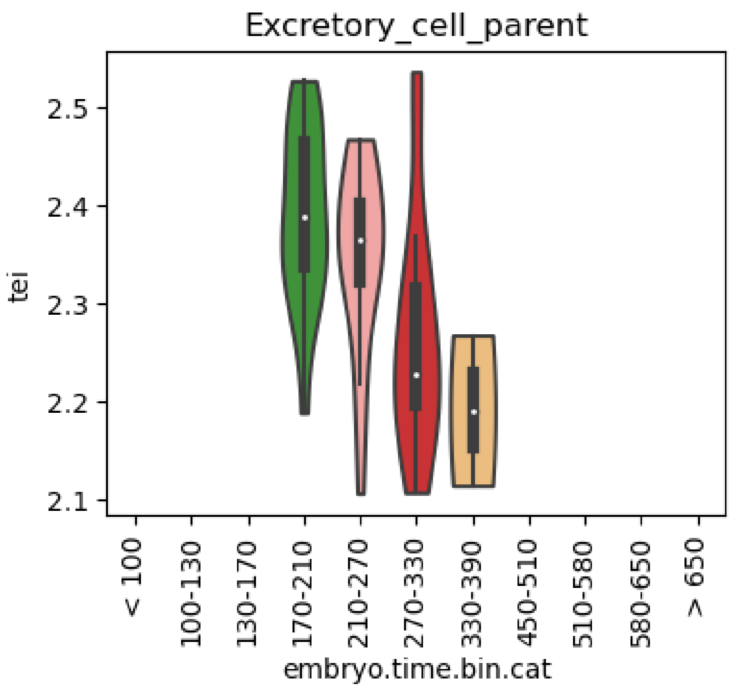
Excretory_cell_parent
tei
2.5
2.4
2.3
2.2
2.1
< 100
100-130
130-170
170-210
210-270
270-330
330-390
450-510
510-580
580-650
> 650
embryo.time.bin.cat

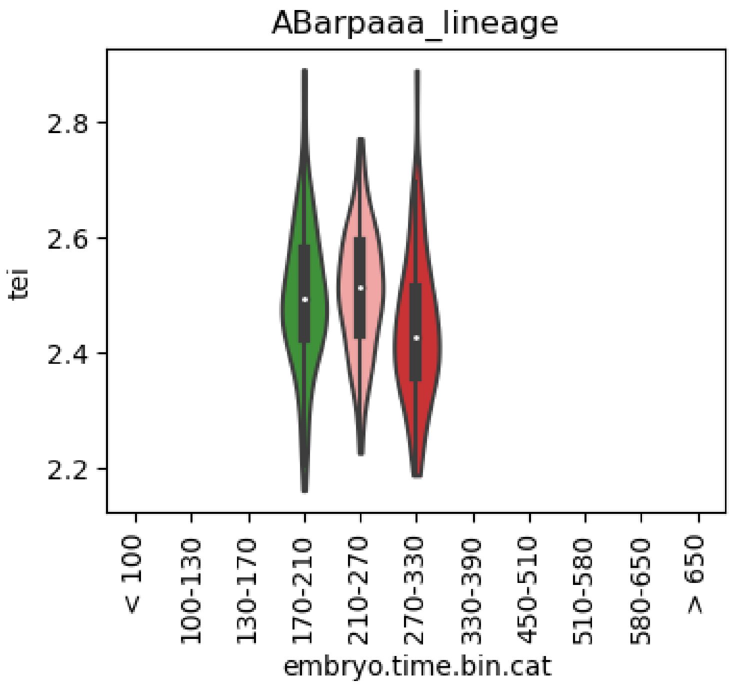
ABarpaaa_lineage
tei
2.8
2.6
2.4
2.2
< 100
100-130
130-170
170-210
210-270
270-330
330-390
450-510
510-580
580-650
> 650
embryo.time.bin.cat

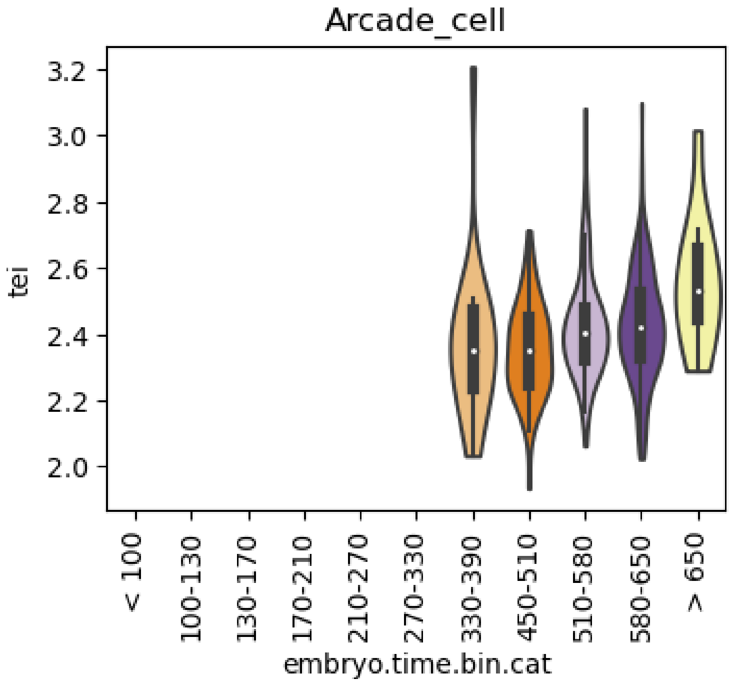
Arcade_cell
3.2
3.0
2.8
2.6
2.4
2.2
2.0
tei
< 100
100-130
130-170
170-210
210-270
270-330
330-390
450-510
510-580
580-650
> 650
embryo.time.bin.cat

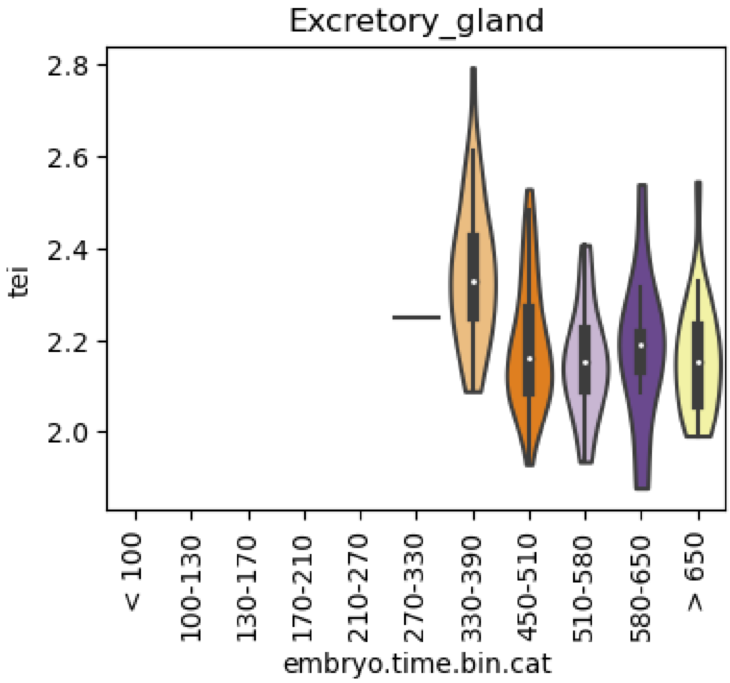
Excretory_gland
2.8
2.6
2.4
2.2
2.0
tei
< 100
100-130
130-170
170-210
210-270
270-330
330-390
450-510
510-580
580-650
> 650
embryo.time.bin.cat

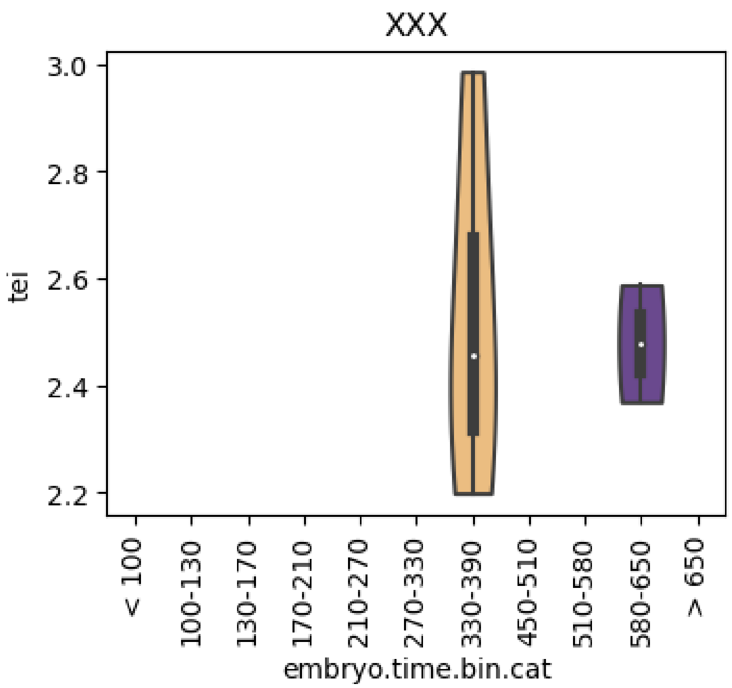

XXX
tei
3.0
2.8
2.6
2.4
2.2
< 100
100-130
130-170
170-210
210-270
270-330
330-390
450-510
510-580
580-650
> 650
embryo.time.bin.cat

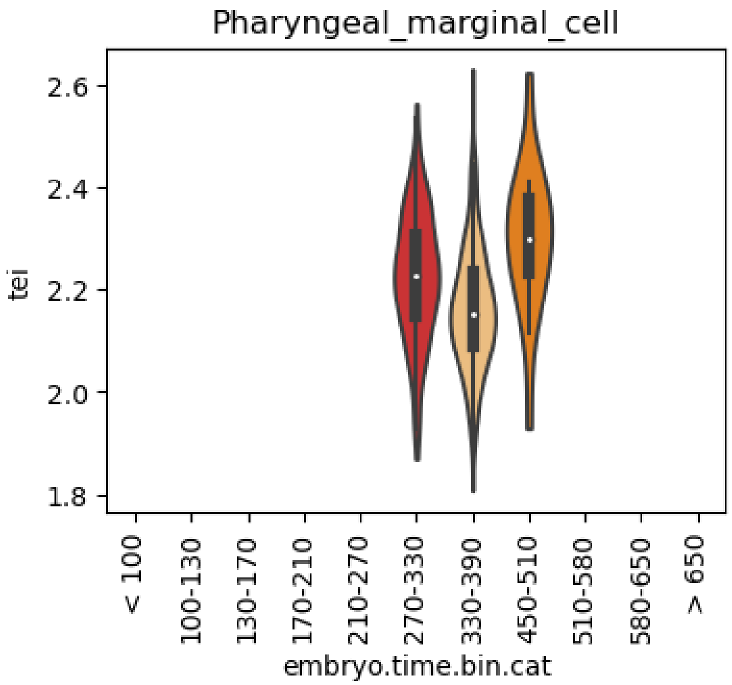

Pharyngeal_marginal_cell
tei
2.6
2.4
2.2
2.0
1.8
< 100
100-130
130-170
170-210
210-270
270-330
330-390
450-510
510-580
580-650
> 650
embryo.time.bin.cat

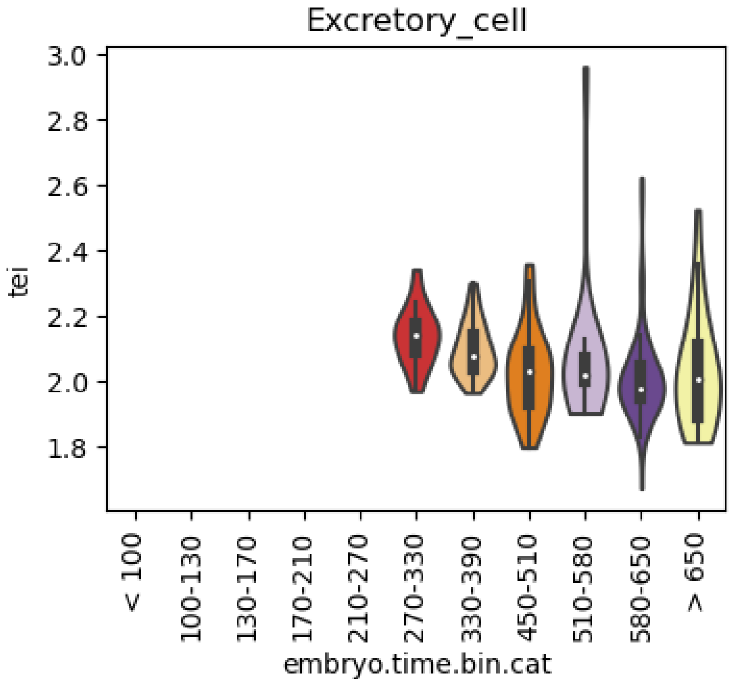
Excretory_cell
3.0
2.8
2.6
2.4
2.2
2.0
1.8
tei
< 100
100-130
130-170
170-210
210-270
270-330
330-390
450-510
510-580
580-650
> 650
embryo.time.bin.cat

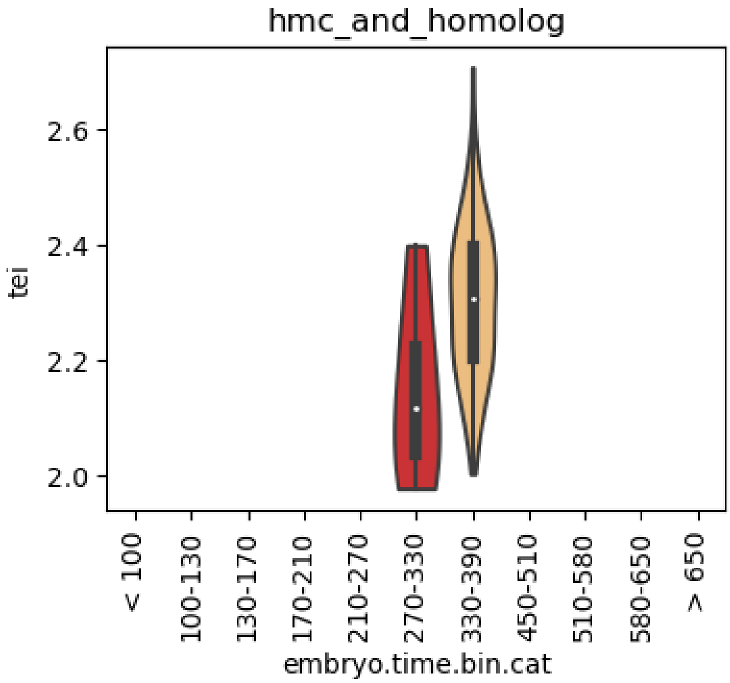
hmc_and_homolog
2.6
2.4
2.2
2.0
tei
< 100
100-130
130-170
170-210
210-270
270-330
330-390
450-510
510-580
580-650
> 650
embryo.time.bin.cat

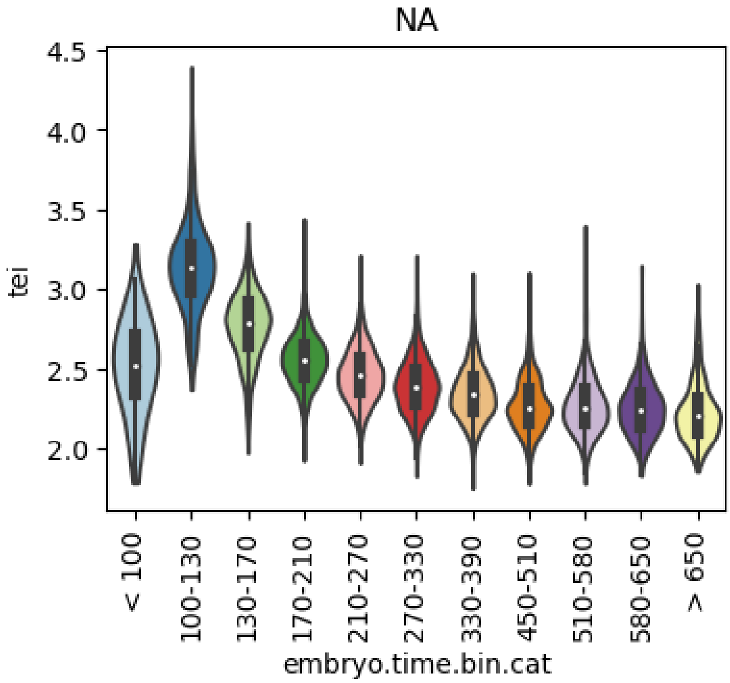
NA
4.5
4.0
3.5
3.0
2.5
2.0
tei
< 100
100-130
130-170
170-210
210-270
270-330
330-390
450-510
510-580
580-650
> 650
embryo.time.bin.cat

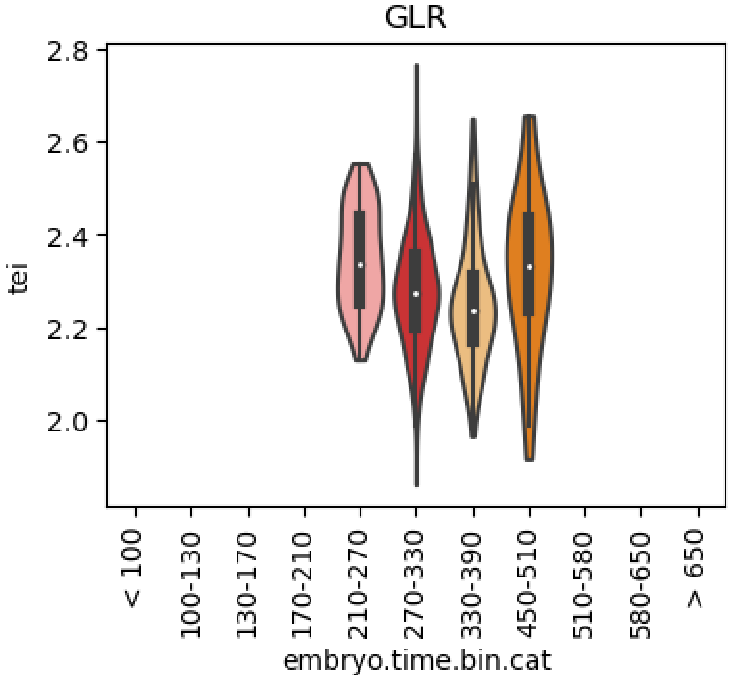
GLR
2.8
2.6
2.4
2.2
2.0
tei
< 100
100-130
130-170
170-210
210-270
270-330
330-390
450-510
510-580
580-650
> 650
embryo.time.bin.cat

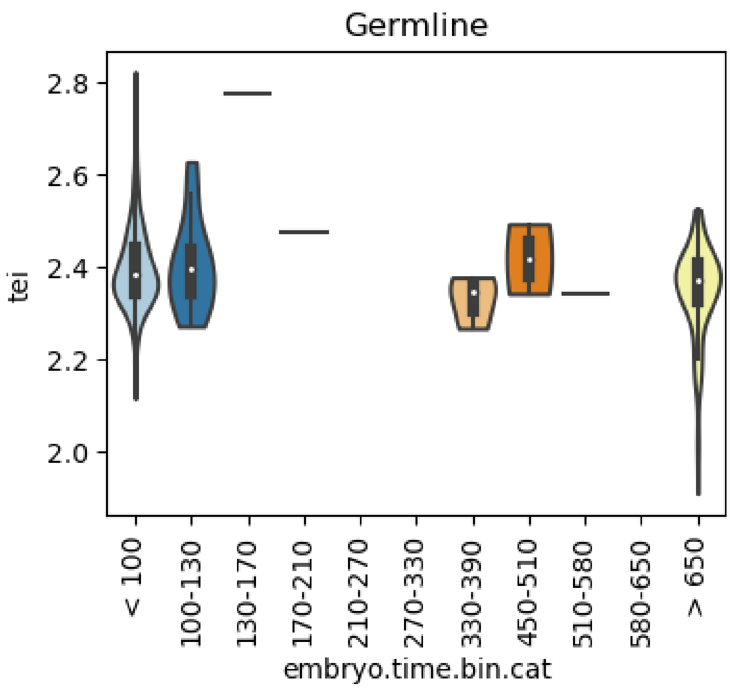
Germline
2.8
2.6
2.4
2.2
2.0
tei
< 100
100-130
130-170
170-210
210-270
270-330
330-390
450-510
510-580
580-650
> 650
embryo.time.bin.cat

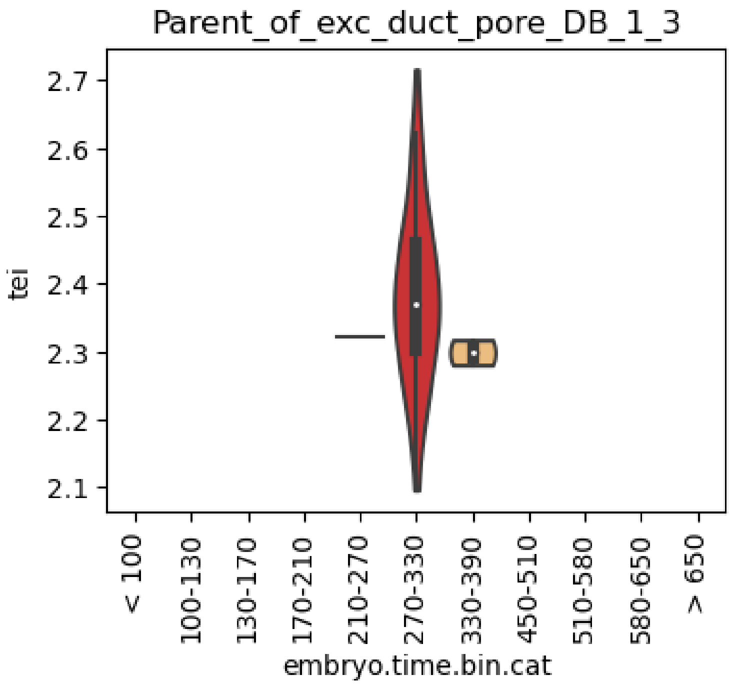
Parent_of_exc_duct_pore_DB_1_3
2.7
2.6
2.5
2.4
2.3
2.2
2.1
tei
< 100
100-130
130-170
170-210
210-270
270-330
330-390
450-510
510-580
580-650
> 650
embryo.time.bin.cat

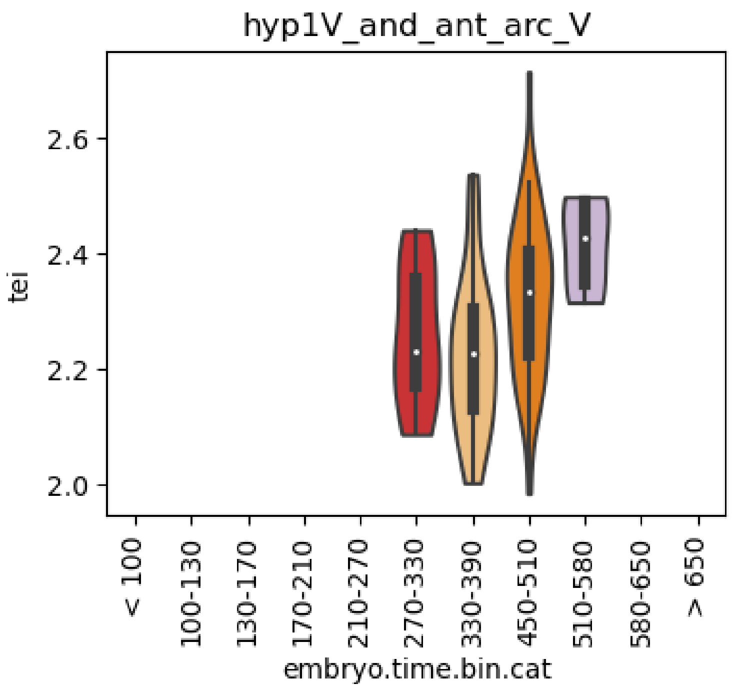
hyp1V_and_ant_arc_V
tei
2.6
2.4
2.2
2.0
< 100
100-130
130-170
170-210
210-270
270-330
330-390
450-510
510-580
580-650
> 650
embryo.time.bin.cat

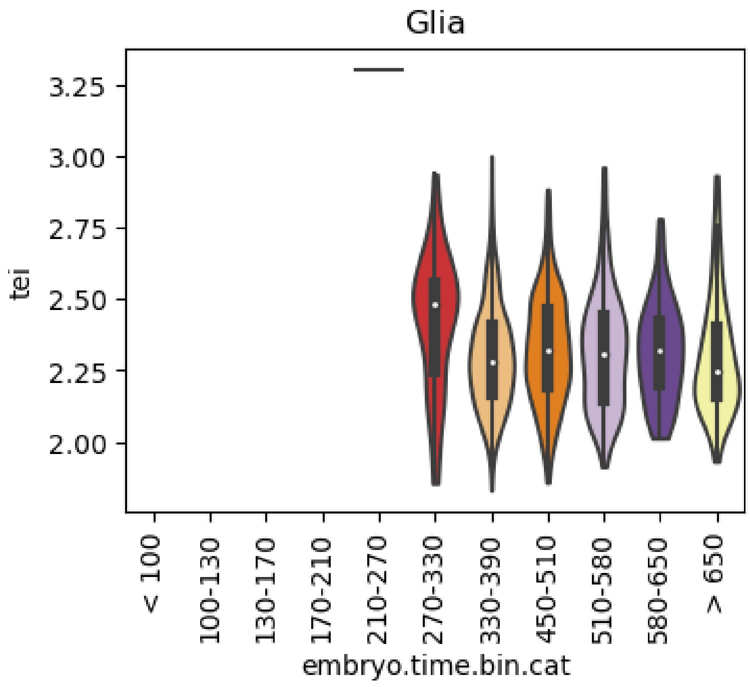
Glia
tei
3.25
3.00
2.75
2.50
2.25
2.00
< 100
100-130
130-170
170-210
210-270
270-330
330-390
450-510
510-580
580-650
> 650
embryo.time.bin.cat

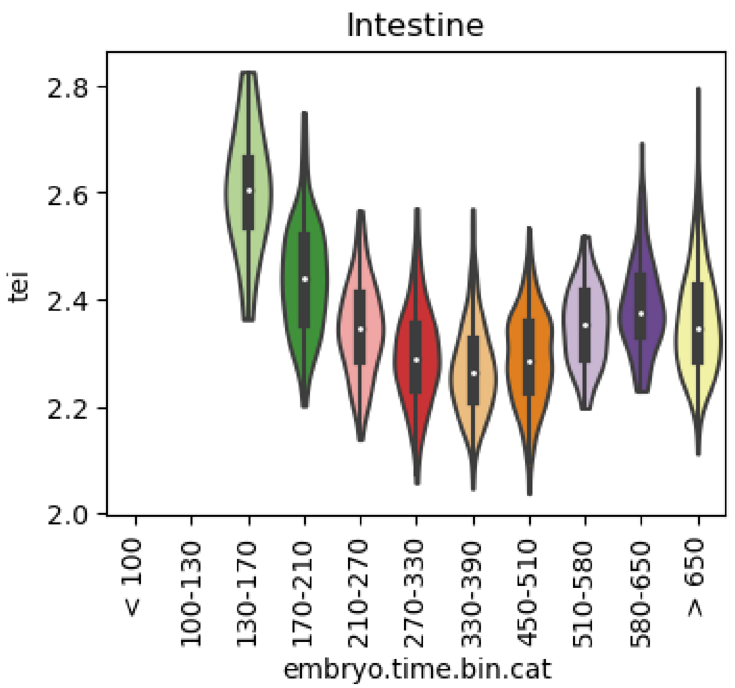

Intestine
tei
2.8
2.6
2.4
2.2
2.0
< 100
100-130
130-170
170-210
210-270
270-330
330-390
450-510
510-580
580-650
> 650
embryo.time.bin.cat

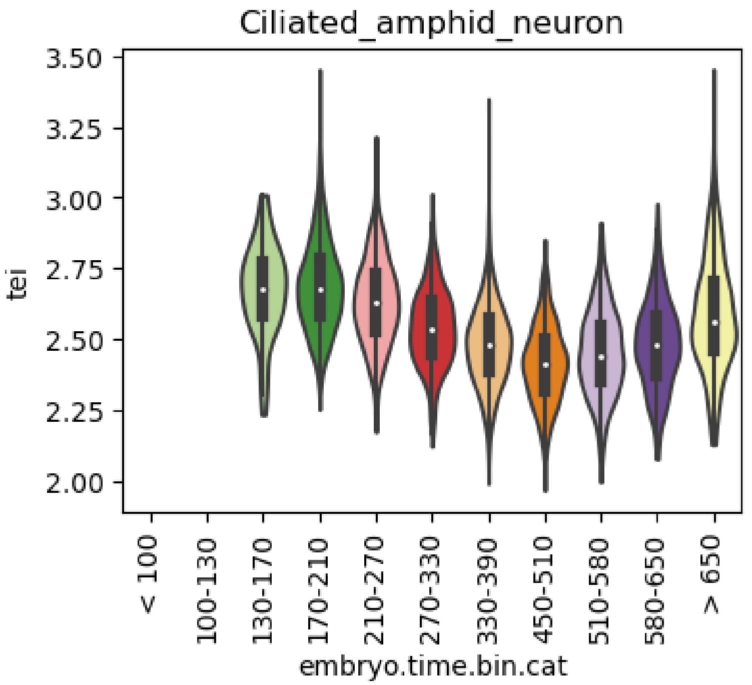

Ciliated_amphid_neuron
tei
3.50
3.25
3.00
2.75
2.50
2.25
2.00
< 100
100-130
130-170
170-210
210-270
270-330
330-390
450-510
510-580
580-650
> 650
embryo.time.bin.cat

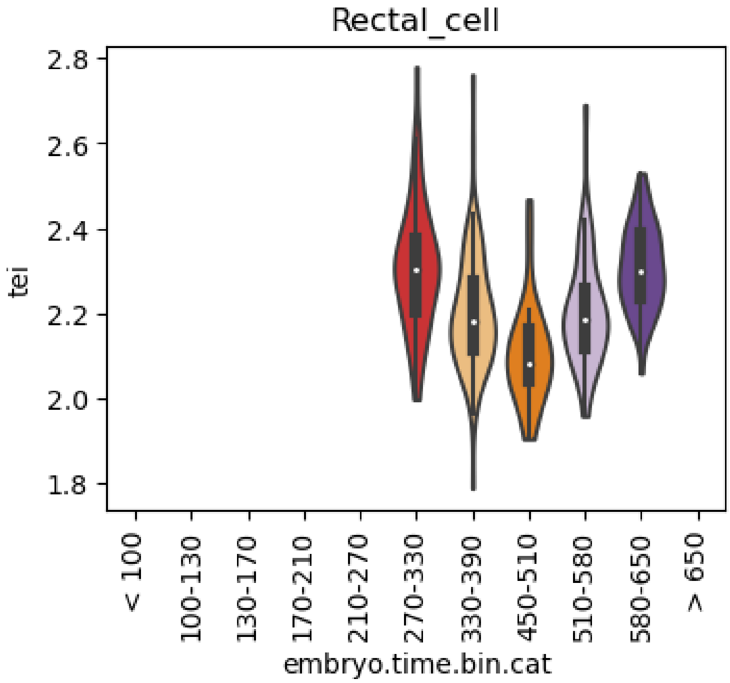
Rectal_cell
2.8
2.6
2.4
2.2
2.0
1.8
tei
< 100
100-130
130-170
170-210
210-270
270-330
330-390
450-510
510-580
580-650
> 650
embryo.time.bin.cat

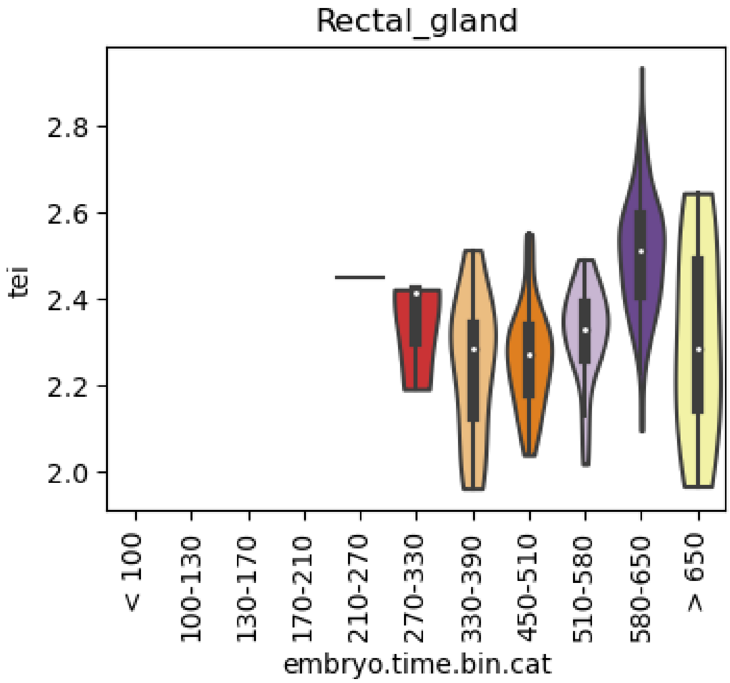
Rectal_gland
2.8
2.6
2.4
2.2
2.0
tei
< 100
100-130
130-170
170-210
210-270
270-330
330-390
450-510
510-580
580-650
> 650
embryo.time.bin.cat

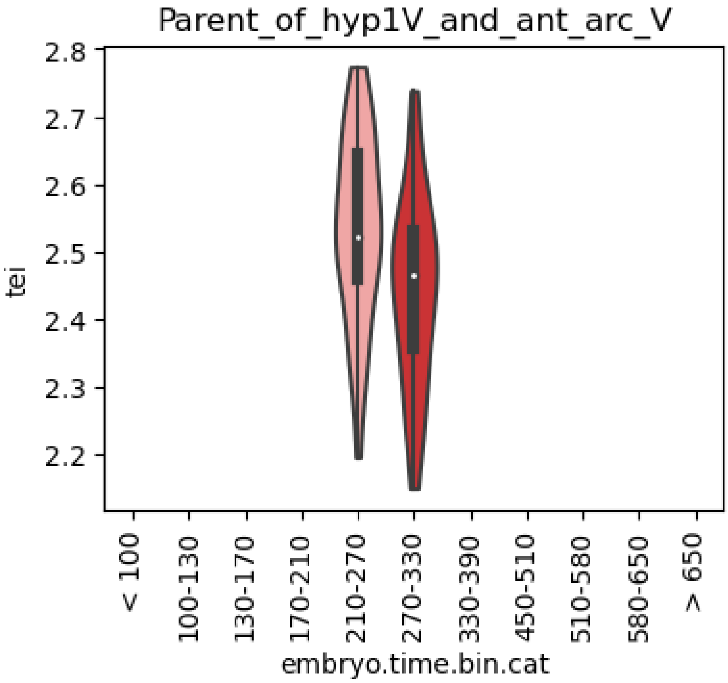

1.11.7 Scatterplot TEI vs TajimaD and color each cell by sample timepoint per cell type

Note: Please change notebook cell from `raw` to `code` to see the plots.

```
for ct in list(set(celegans_data.obs['cell.type'])):
    ax = sns.scatterplot(data=celegans_data[celegans_data.obs['cell.type']==ct].obs,
            x='TajimaD',
            y='tei',
            hue='embryo.time.bin.cat')
    ax.set_title(ct)
    sns.move_legend(ax, 'upper left', bbox_to_anchor=(1, 1))
    plt.show()
```

1.11.8 Scatterplot TEI vs Fst and color each cell by sample timepoint per cell type

Note: Please change notebook cell from `raw` to `code` to see the plots.

```
for ct in list(set(celegans_data.obs['cell.type'])):
    ax = sns.scatterplot(data=celegans_data[celegans_data.obs['cell.type']==ct].obs,
            x='Fst',
            y='tei',
            hue='embryo.time.bin.cat')
    ax.set_title(ct)
    sns.move_legend(ax, 'upper left', bbox_to_anchor=(1, 1))
    plt.show()
```

1.11.9 Scatterplot TEI vs NormalizedPi and color each cell by sample timepoint per cell type

Note: Please change notebook cell from `raw` to `code` to see the plots.

[29]:

```
for ct in list(set(celegans_data.obs['cell.type'])):
    ax = sns.scatterplot(data=celegans_data[celegans_data.obs['cell.type']==ct].obs,
            x='NormalizedPi',
            y='tei',
            hue='embryo.time.bin.cat')
    ax.set_title(ct)
    sns.move_legend(ax, 'upper left', bbox_to_anchor=(1, 1))
    plt.show()
```

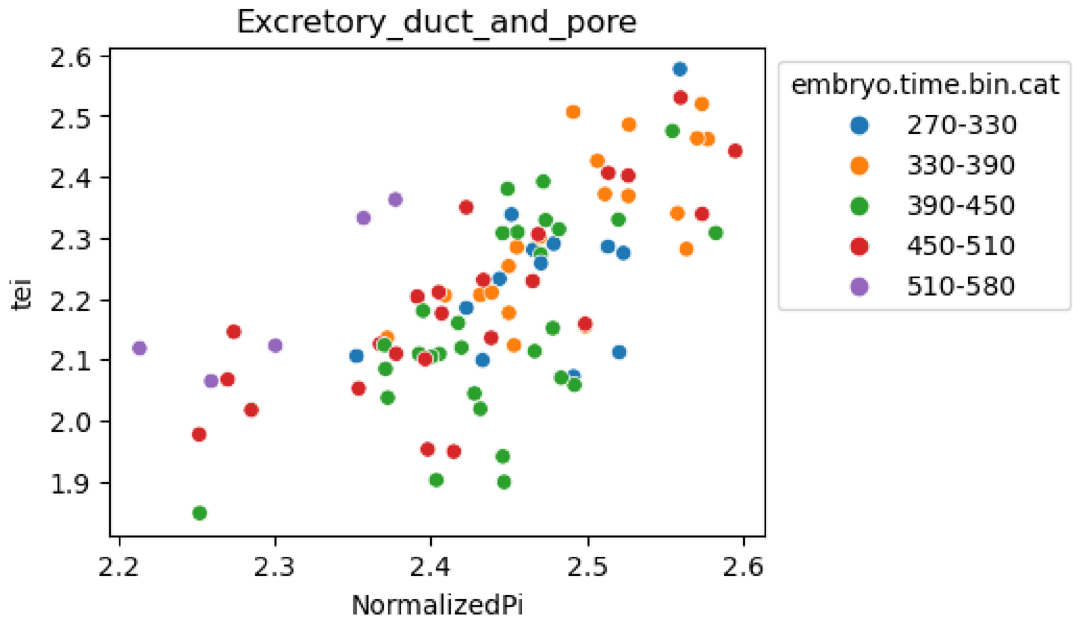

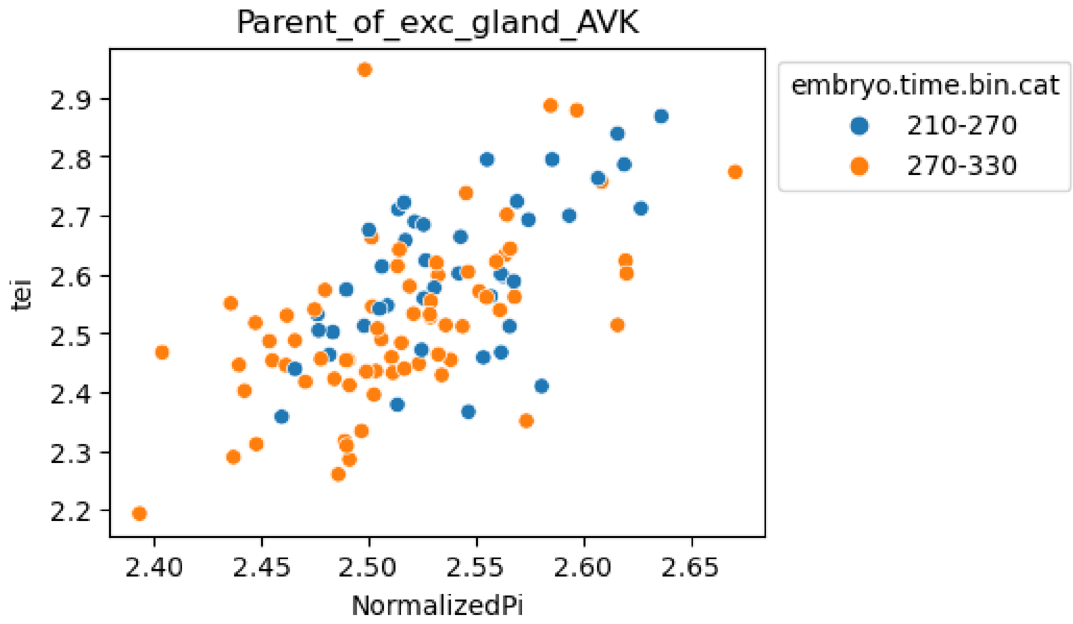

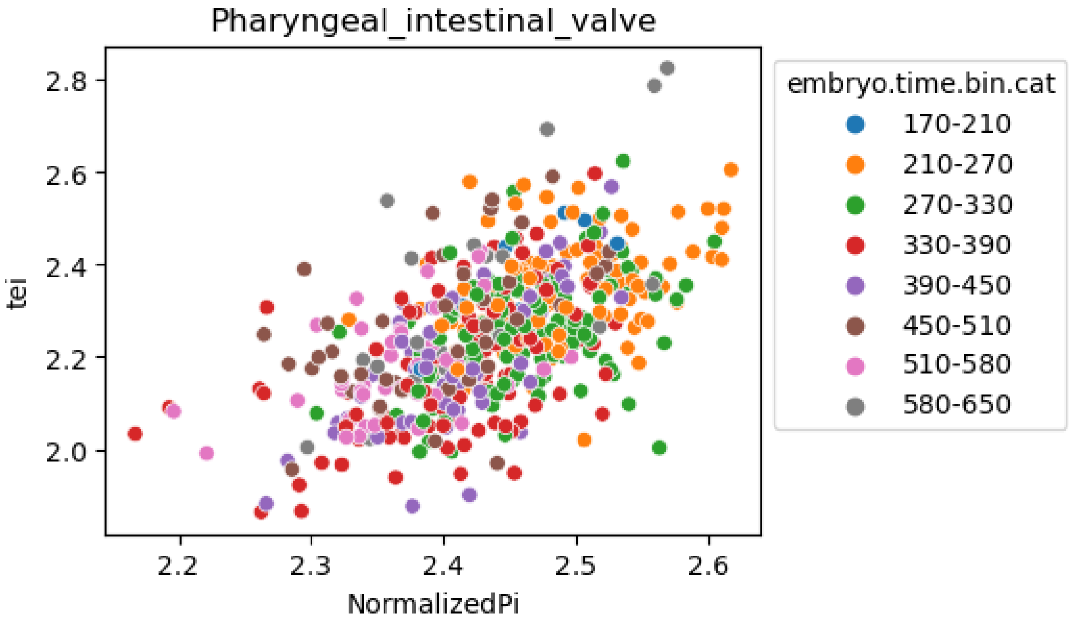
Pharyngeal_intestinal_valve
tei
NormalizedPi
2.8
2.6
2.4
2.2
2.0
2.2
2.3
2.4
2.5
2.6
embryo.time.bin.cat
170-210
210-270
270-330
330-390
390-450
450-510
510-580
580-650

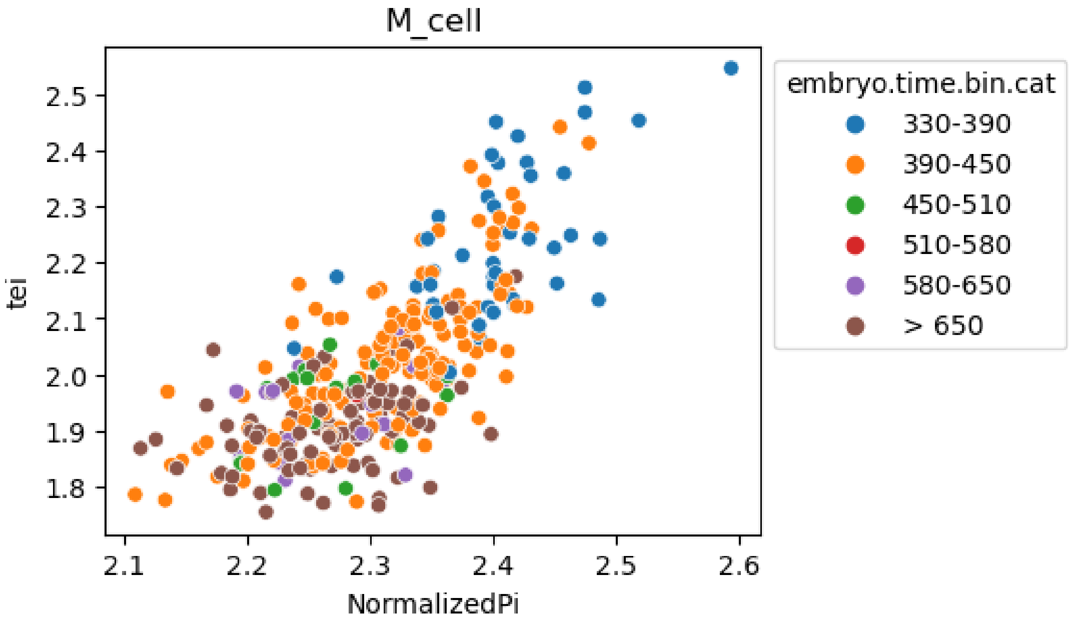
M_cell
tei
NormalizedPi
2.5
2.4
2.3
2.2
2.1
2.0
1.9
1.8
2.1
2.2
2.3
2.4
2.5
2.6
embryo.time.bin.cat
330-390
390-450
450-510
510-580
580-650
> 650

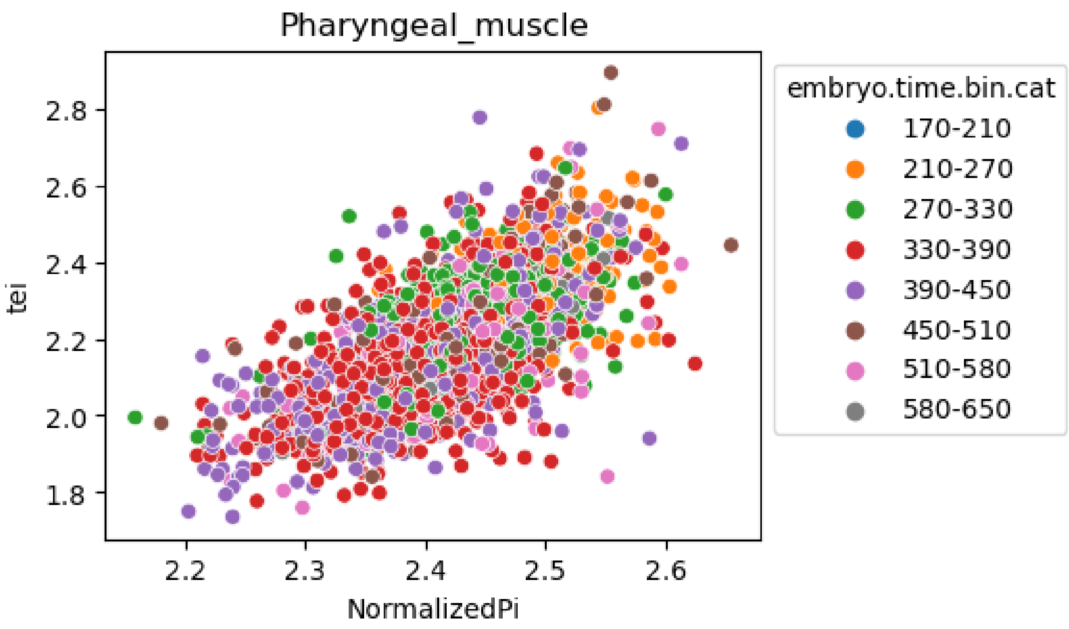
Pharyngeal_muscle
tei
2.8
2.6
2.4
2.2
2.0
1.8
2.2
2.3
2.4
2.5
2.6
NormalizedPi
embryo.time.bin.cat
170-210
210-270
270-330
330-390
390-450
450-510
510-580
580-650

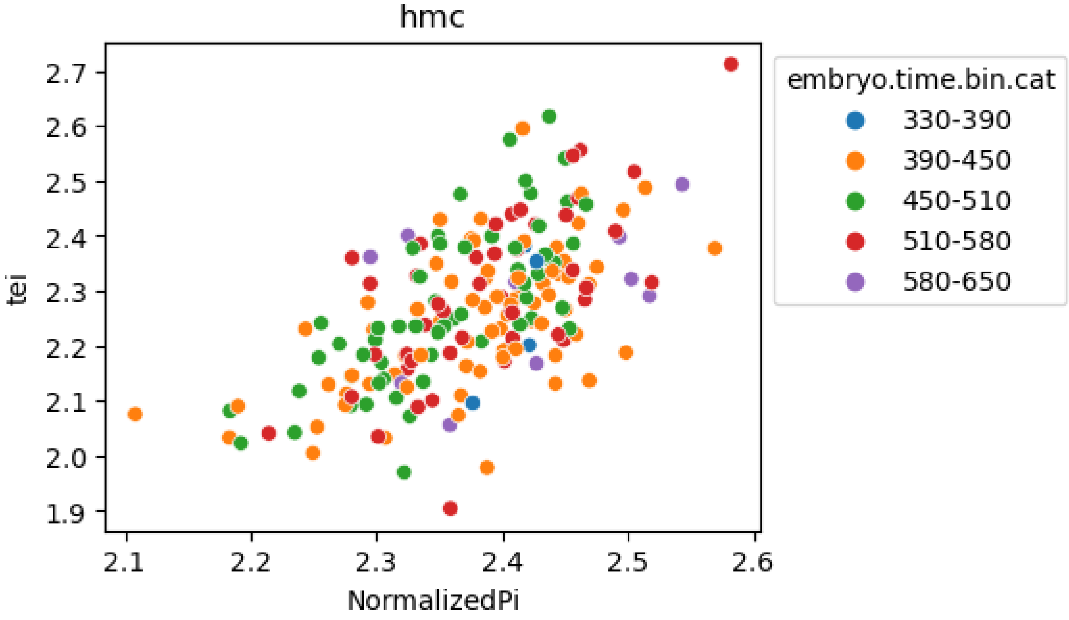
hmc
tei
2.7
2.6
2.5
2.4
2.3
2.2
2.1
2.0
1.9
2.1
2.2
2.3
2.4
2.5
2.6
NormalizedPi
embryo.time.bin.cat
330-390
390-450
450-510
510-580
580-650

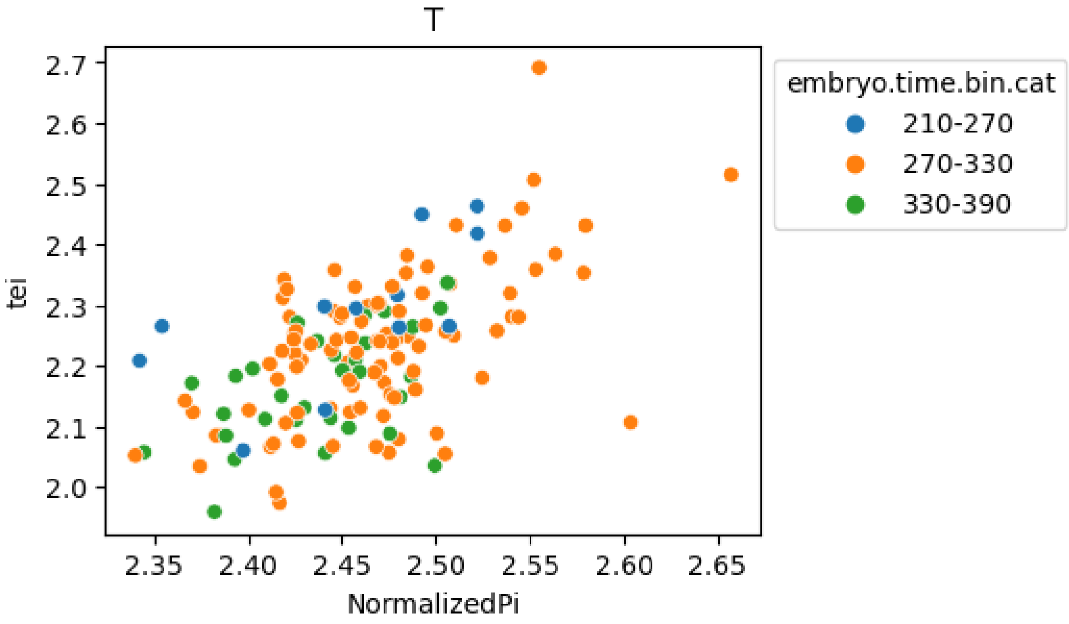
T
tei
NormalizedPi
embryo.time.bin.cat
210-270
270-330
330-390
2.0
2.1
2.2
2.3
2.4
2.5
2.6
2.7
2.35
2.40
2.45
2.50
2.55
2.60
2.65

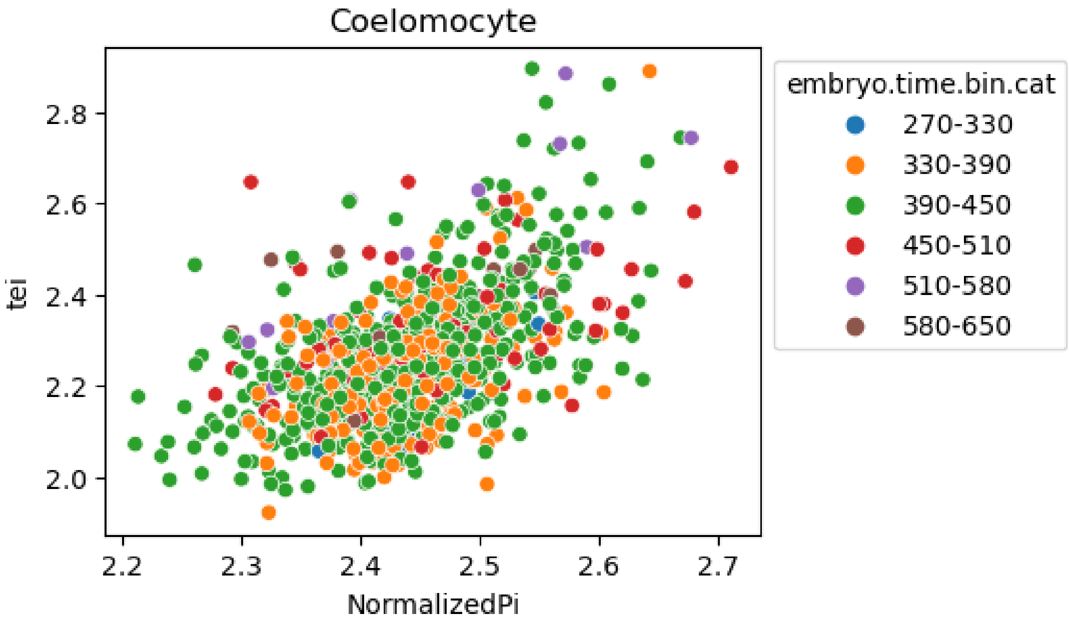
Coelomocyte
tei
NormalizedPi
embryo.time.bin.cat
270-330
330-390
390-450
450-510
510-580
580-650
2.0
2.2
2.4
2.6
2.8
2.2
2.3
2.4
2.5
2.6
2.7

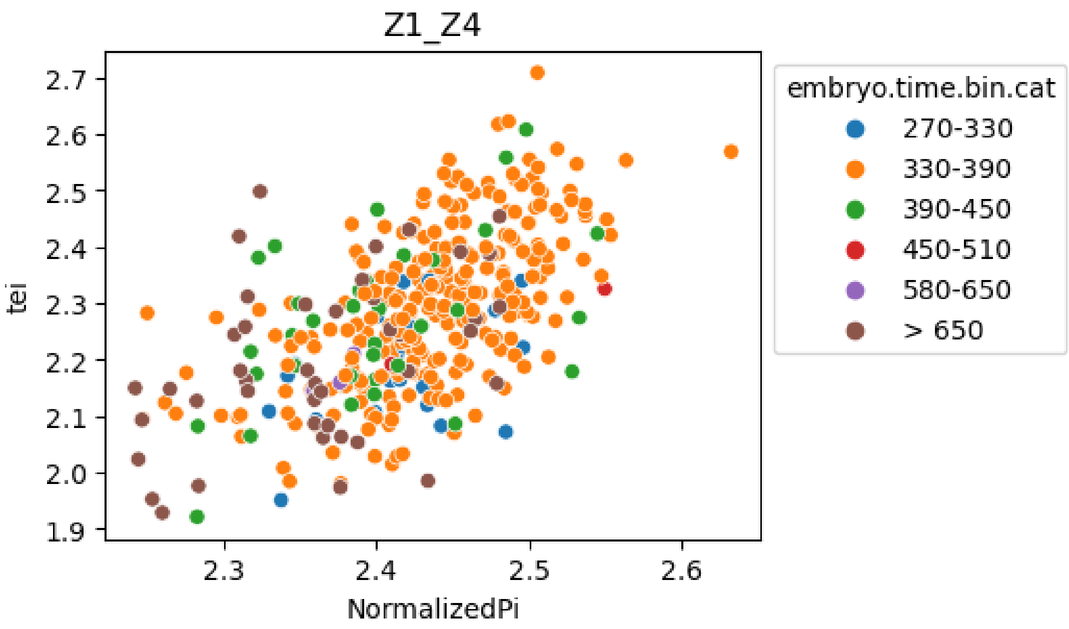
Z1_Z4
tei
NormalizedPi
embryo.time.bin.cat
270-330
330-390
390-450
450-510
580-650
> 650

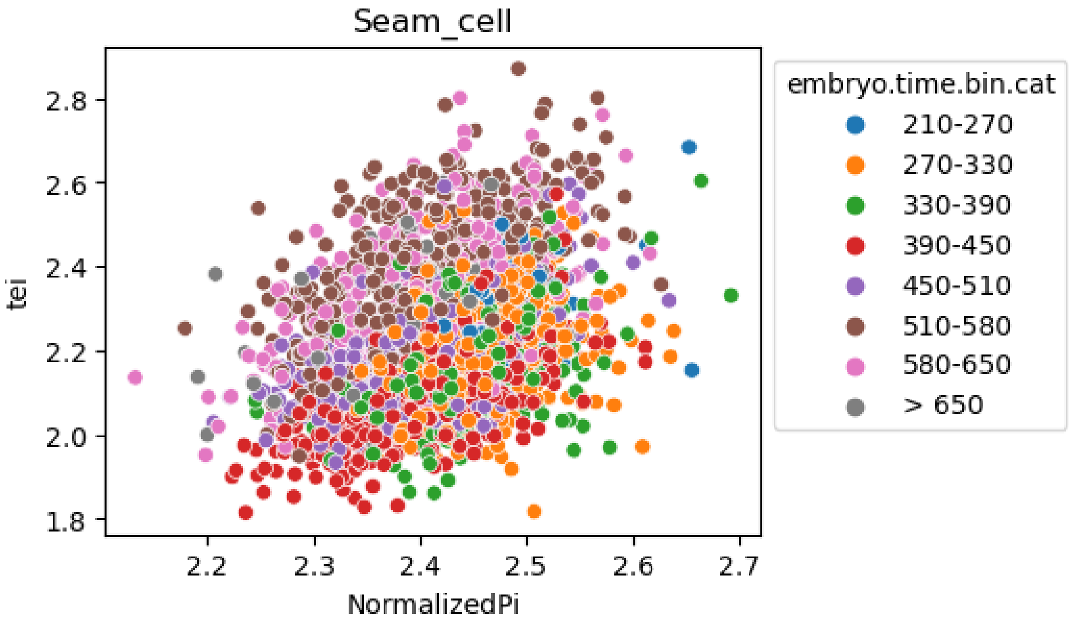
Seam_cell
tei
NormalizedPi
embryo.time.bin.cat
210-270
270-330
330-390
390-450
450-510
510-580
580-650
> 650

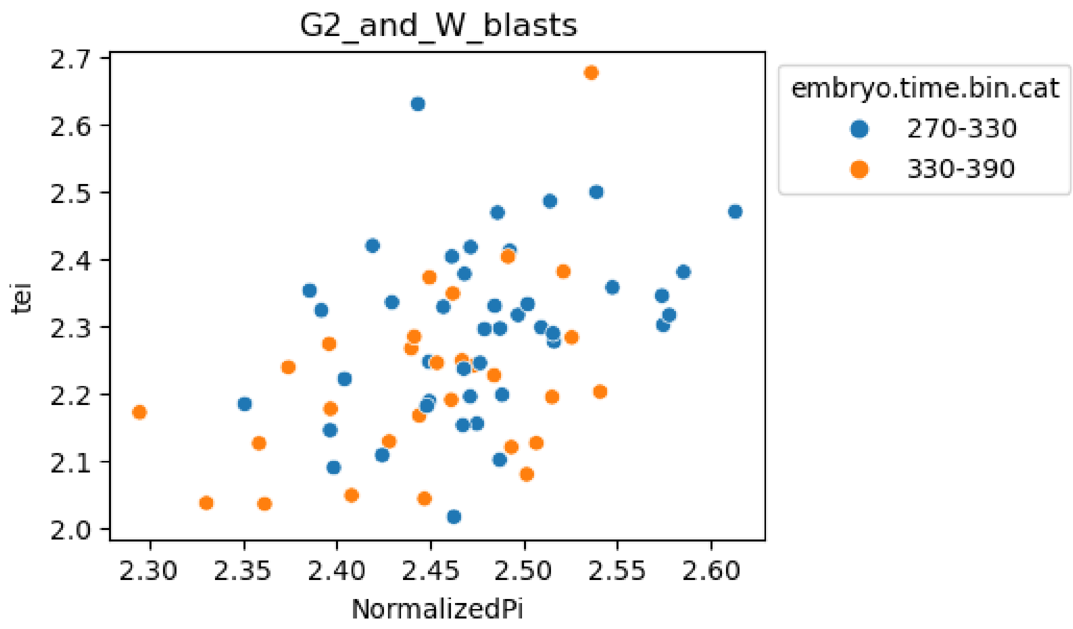
G2_and_W_blasts
embryo.time.bin.cat
270-330
330-390
tei
NormalizedPi
2.0
2.1
2.2
2.3
2.4
2.5
2.6
2.7
2.30
2.35
2.40
2.45
2.50
2.55
2.60

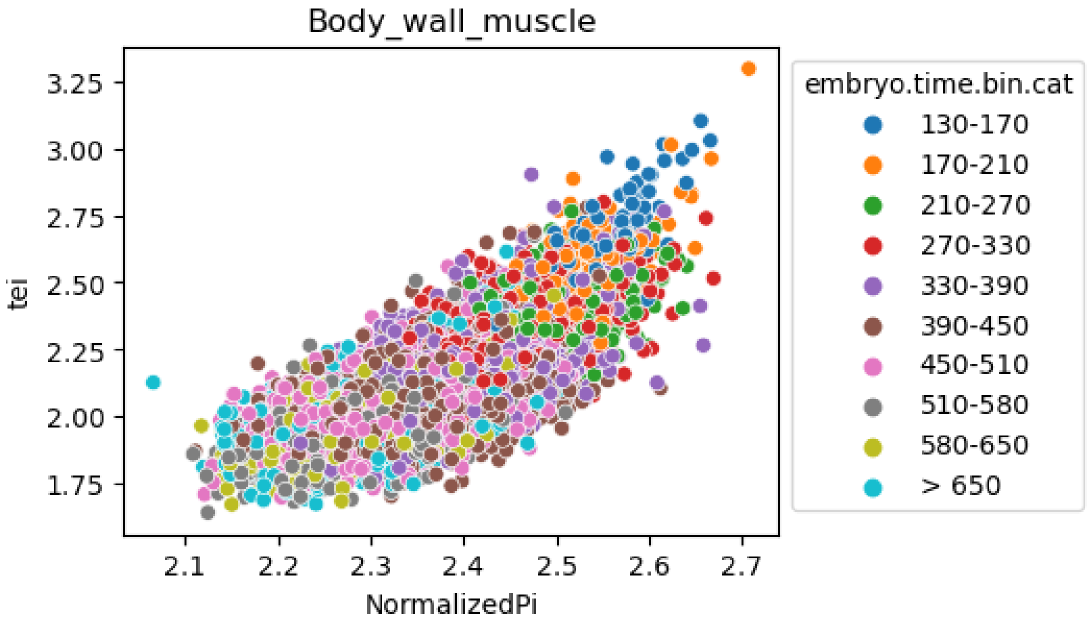
Body_wall_muscle
embryo.time.bin.cat
130-170
170-210
210-270
270-330
330-390
390-450
450-510
510-580
580-650
> 650
tei
NormalizedPi
1.75
2.00
2.25
2.50
2.75
3.00
3.25
2.1
2.2
2.3
2.4
2.5
2.6
2.7

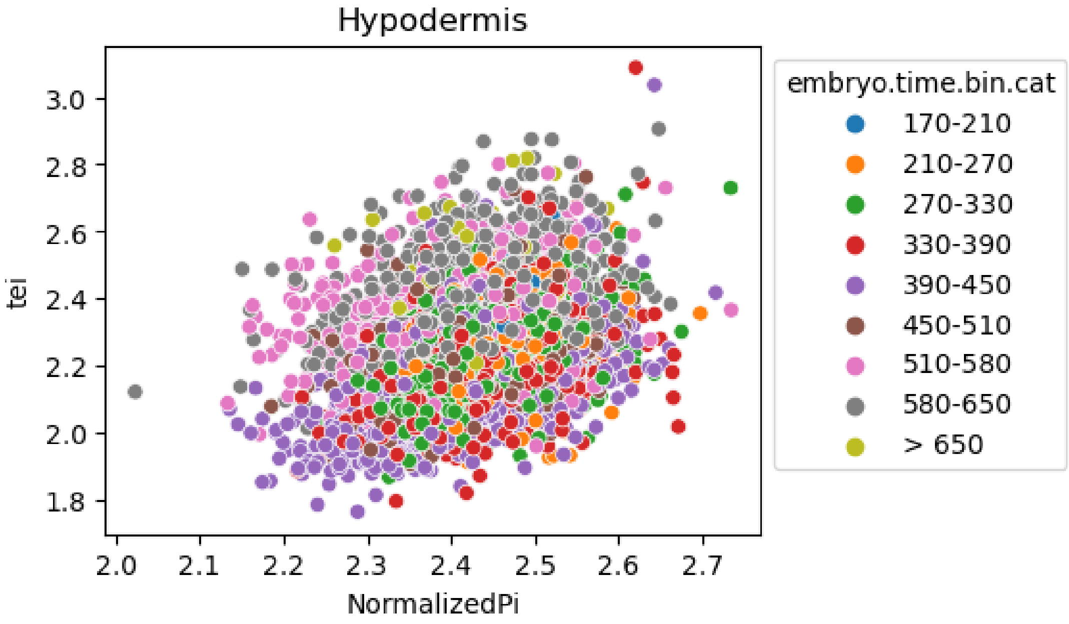
Hypodermis
tei
NormalizedPi
3.0
2.8
2.6
2.4
2.2
2.0
1.8
2.0
2.1
2.2
2.3
2.4
2.5
2.6
2.7
embryo.time.bin.cat
170-210
210-270
270-330
330-390
390-450
450-510
510-580
580-650
> 650

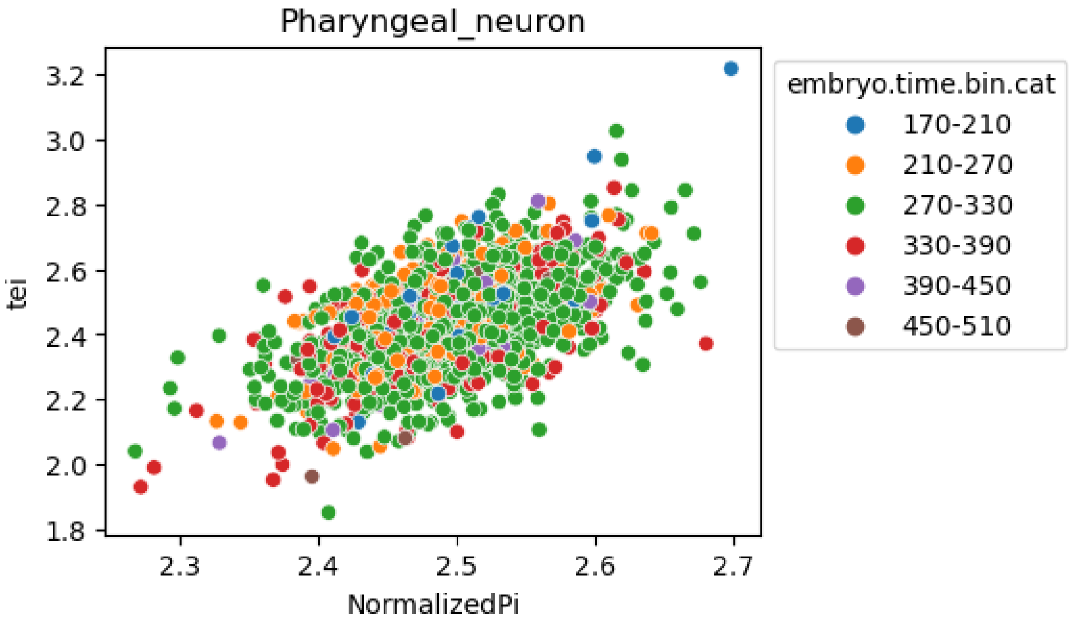
Pharyngeal_neuron
tei
NormalizedPi
3.2
3.0
2.8
2.6
2.4
2.2
2.0
1.8
2.3
2.4
2.5
2.6
2.7
embryo.time.bin.cat
170-210
210-270
270-330
330-390
390-450
450-510

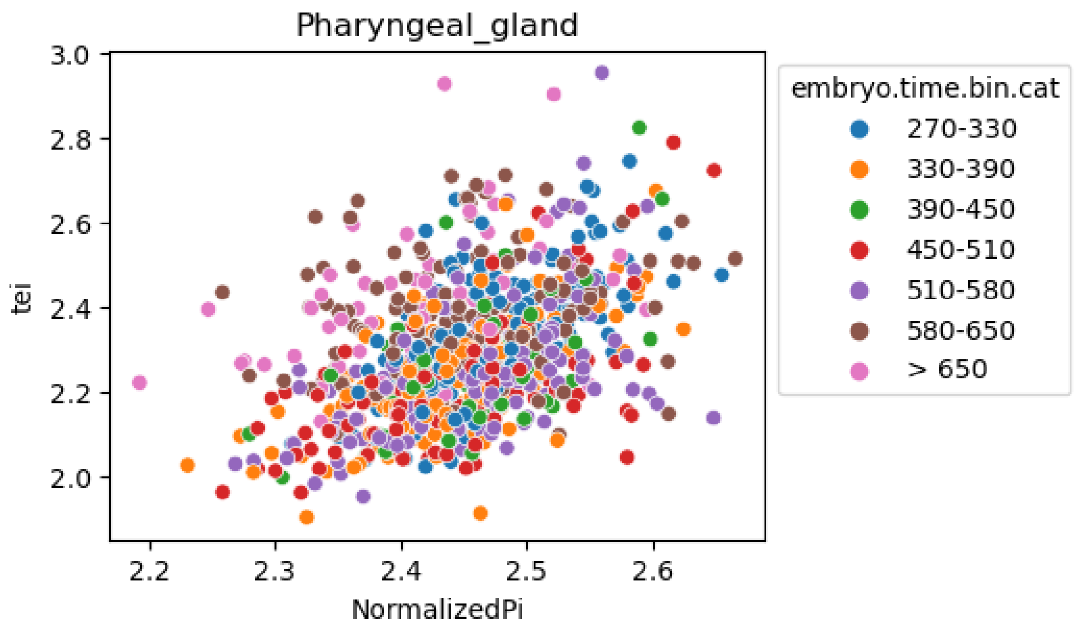
Pharyngeal_gland
tei
NormalizedPi
3.0
2.8
2.6
2.4
2.2
2.0
2.2
2.3
2.4
2.5
2.6
embryo.time.bin.cat
270-330
330-390
390-450
450-510
510-580
580-650
> 650

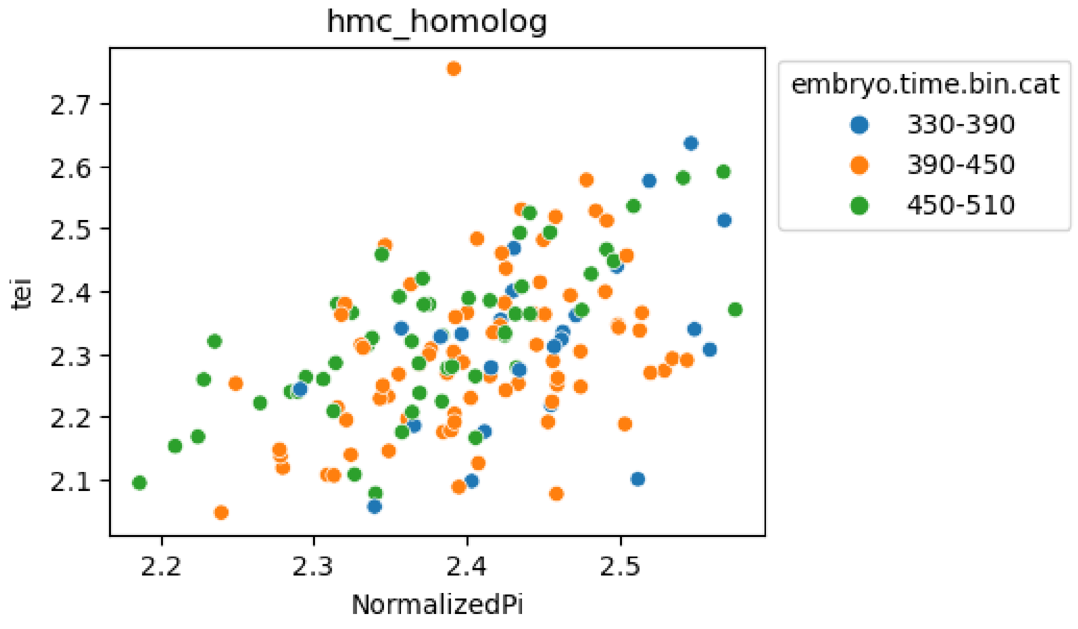
hmc_homolog
tei
NormalizedPi
2.7
2.6
2.5
2.4
2.3
2.2
2.1
2.2
2.3
2.4
2.5
embryo.time.bin.cat
330-390
390-450
450-510

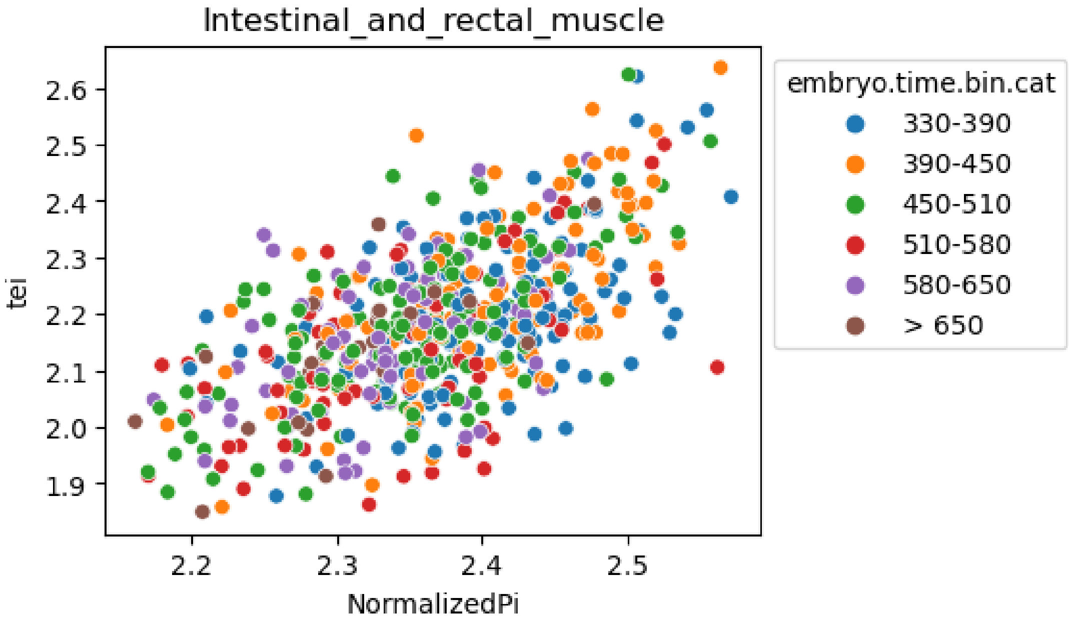
Intestinal_and_rectal_muscle
tei
NormalizedPi
embryo.time.bin.cat
330-390
390-450
450-510
510-580
580-650
> 650

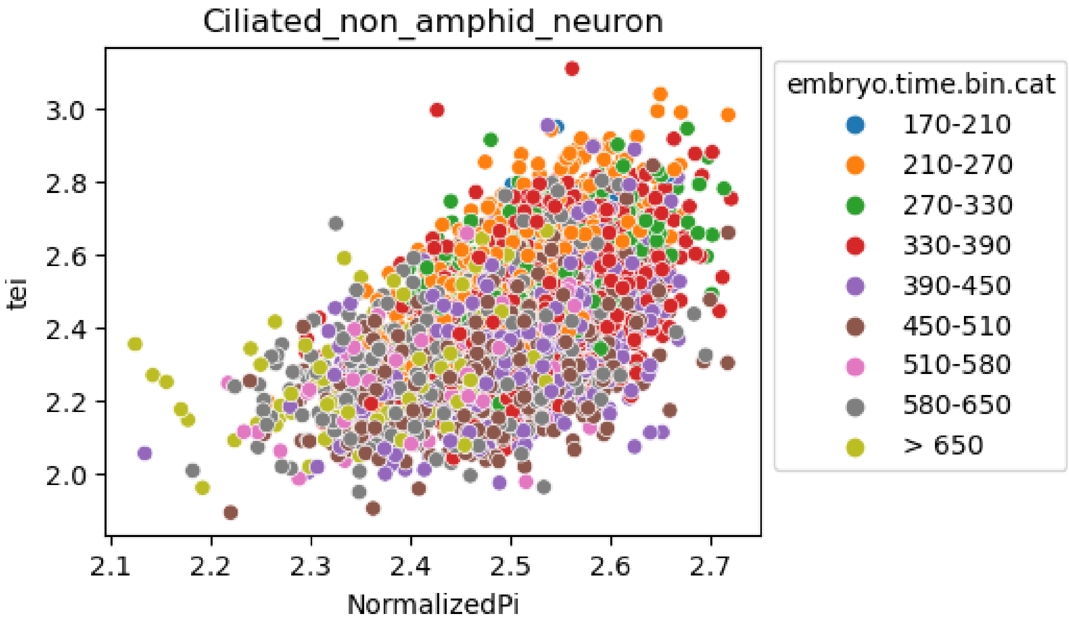
Ciliated_non_amphid_neuron
tei
NormalizedPi
embryo.time.bin.cat
170-210
210-270
270-330
330-390
390-450
450-510
510-580
580-650
> 650

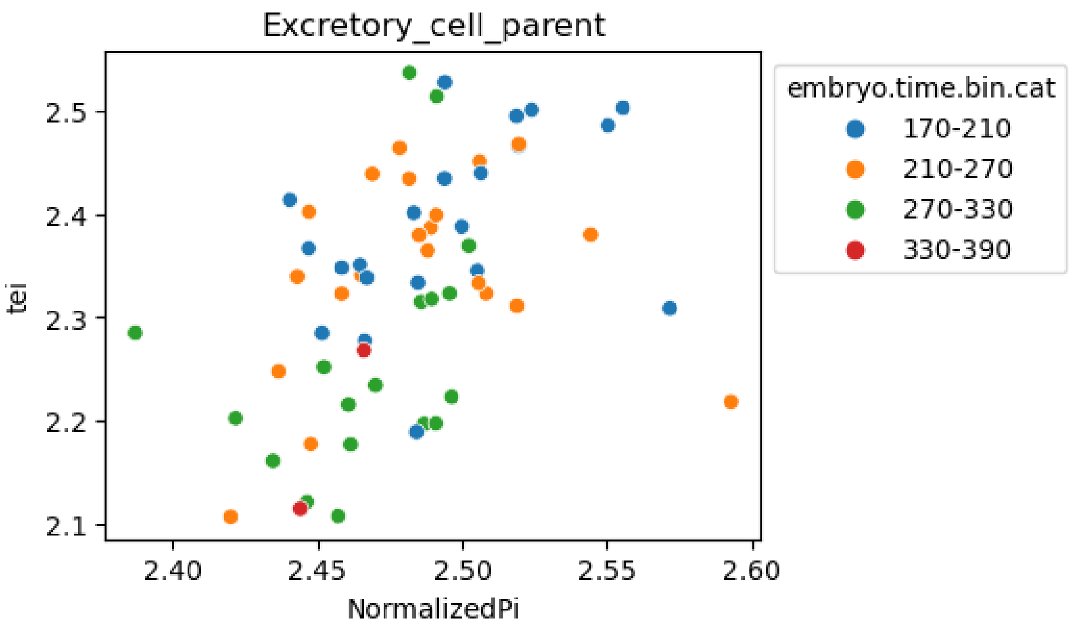
Excretory_cell_parent
tei
2.5
2.4
2.3
2.2
2.1
2.40
2.45
2.50
2.55
2.60
NormalizedPi
embryo.time.bin.cat
170-210
210-270
270-330
330-390

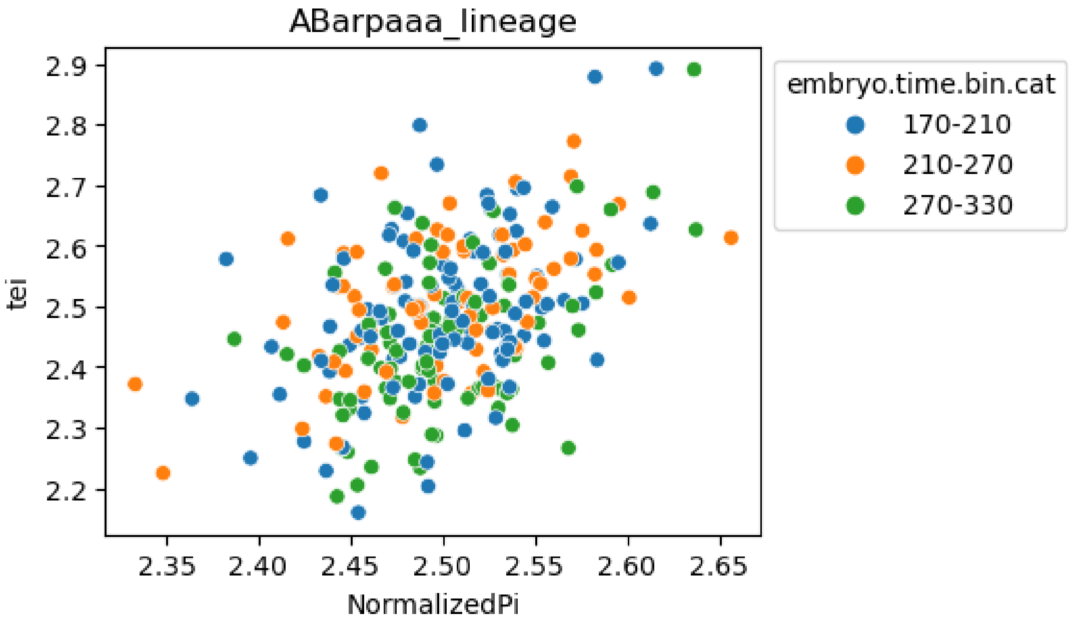
ABarpaaa_lineage
tei
2.9
2.8
2.7
2.6
2.5
2.4
2.3
2.2
2.35
2.40
2.45
2.50
2.55
2.60
2.65
NormalizedPi
embryo.time.bin.cat
170-210
210-270
270-330

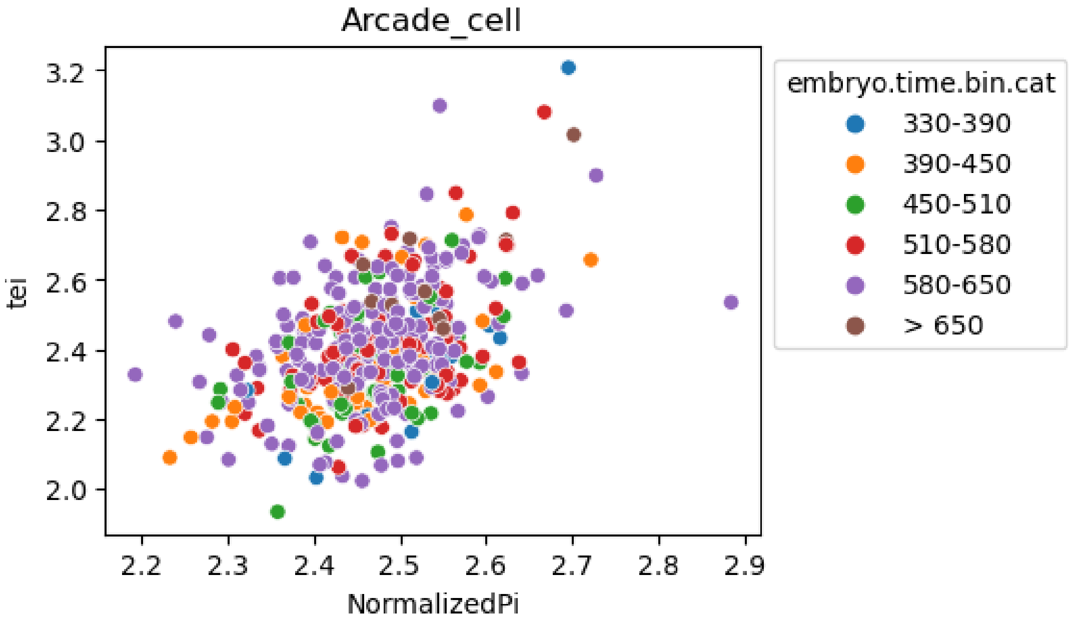
Arcade_cell
tei
3.2
3.0
2.8
2.6
2.4
2.2
2.0
2.2
2.3
2.4
2.5
2.6
2.7
2.8
2.9
NormalizedPi
embryo.time.bin.cat
330-390
390-450
450-510
510-580
580-650
> 650

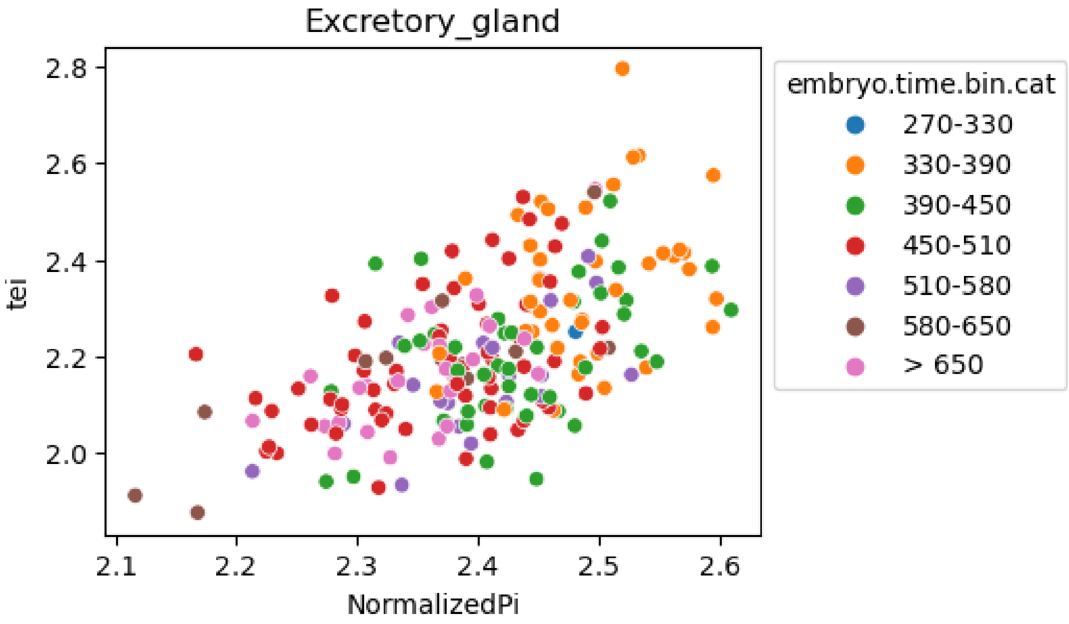
Excretory_gland
tei
2.8
2.6
2.4
2.2
2.0
2.1
2.2
2.3
2.4
2.5
2.6
NormalizedPi
embryo.time.bin.cat
270-330
330-390
390-450
450-510
510-580
580-650
> 650

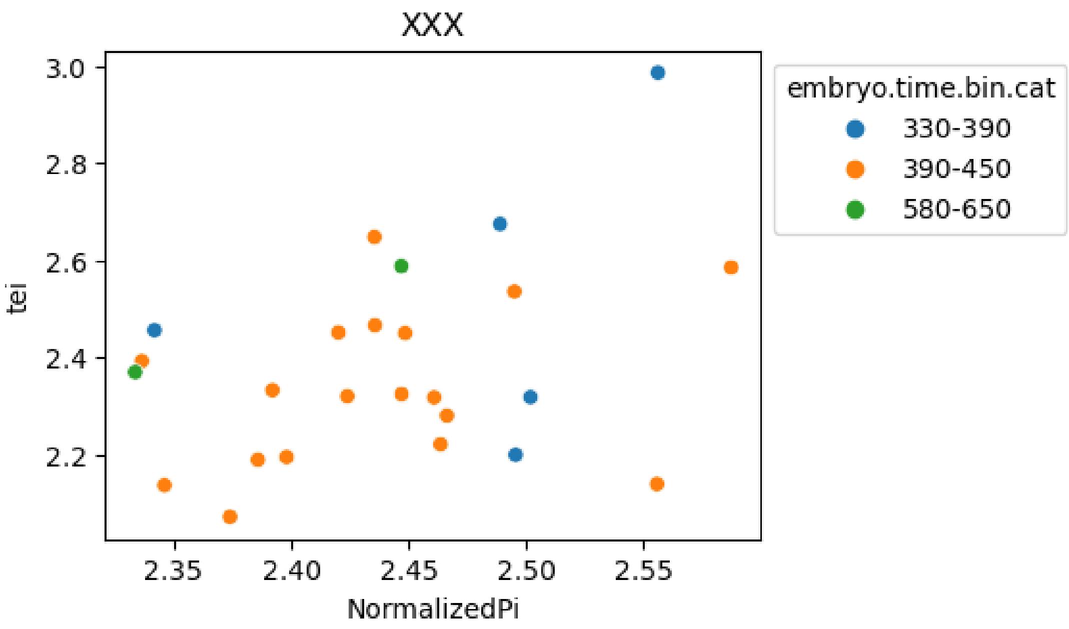
XXX
3.0
2.8
2.6
2.4
2.2
tei
2.35
2.40
2.45
2.50
2.55
NormalizedPi
embryo.time.bin.cat
330-390
390-450
580-650

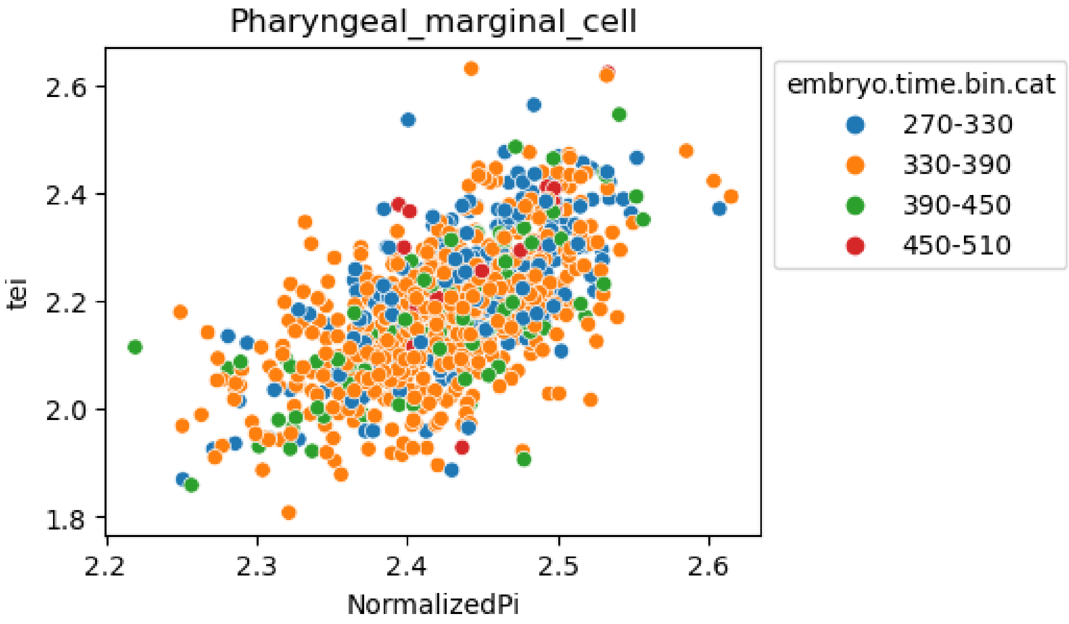
Pharyngeal_marginal_cell
2.6
2.4
2.2
2.0
1.8
tei
2.2
2.3
2.4
2.5
2.6
NormalizedPi
embryo.time.bin.cat
270-330
330-390
390-450
450-510

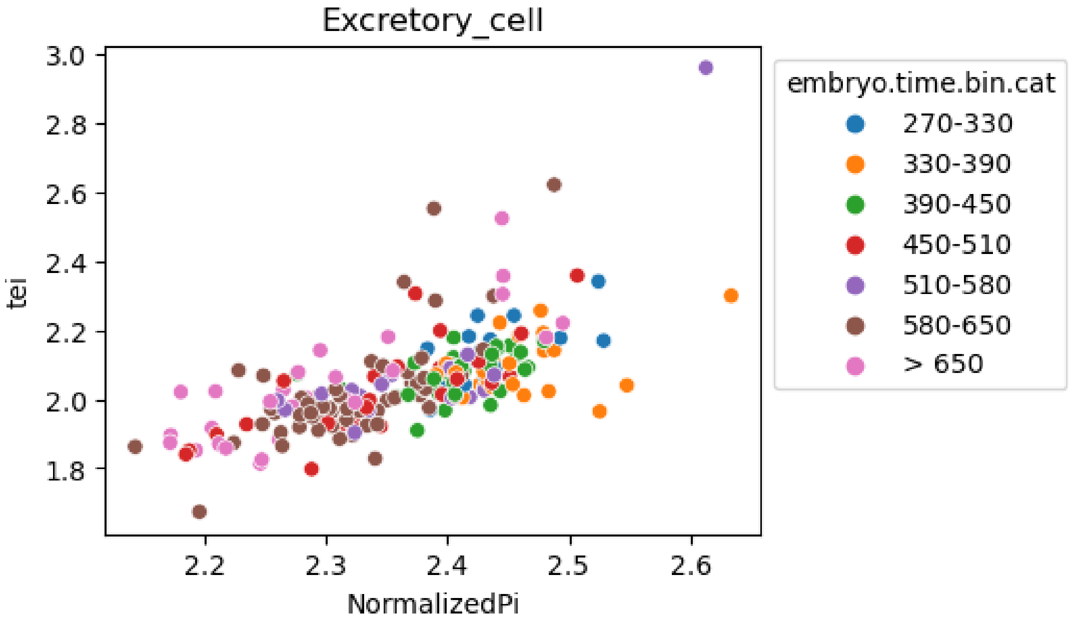
Excretory_cell
3.0
2.8
2.6
2.4
2.2
2.0
1.8
tei
2.2
2.3
2.4
2.5
2.6
NormalizedPi
embryo.time.bin.cat
270-330
330-390
390-450
450-510
510-580
580-650
> 650

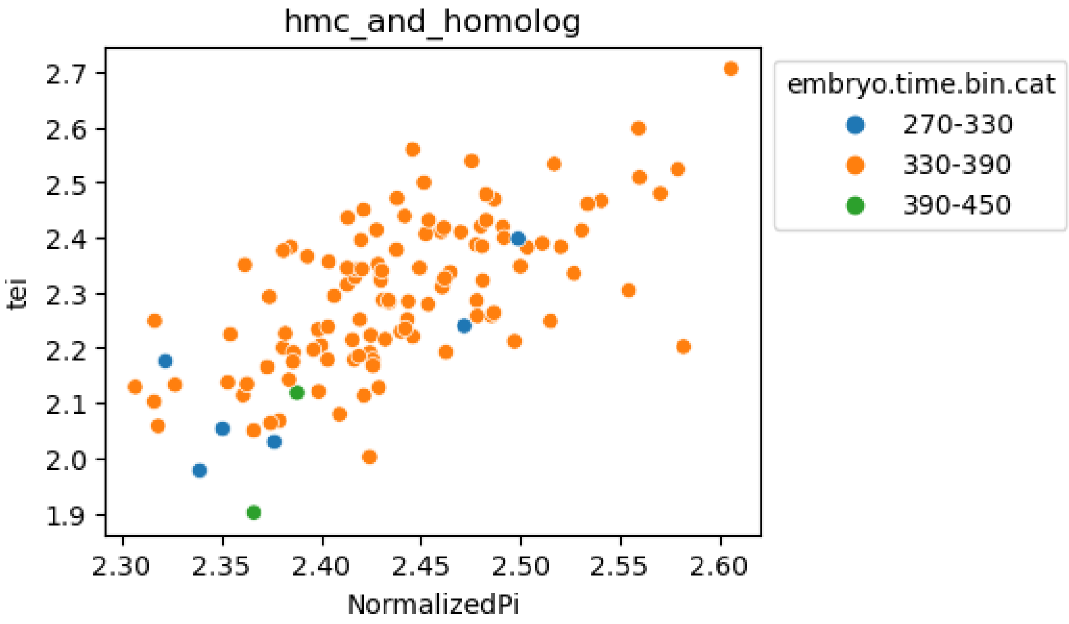
hmc_and_homolog
2.7
2.6
2.5
2.4
2.3
2.2
2.1
2.0
1.9
tei
2.30
2.35
2.40
2.45
2.50
2.55
2.60
NormalizedPi
embryo.time.bin.cat
270-330
330-390
390-450

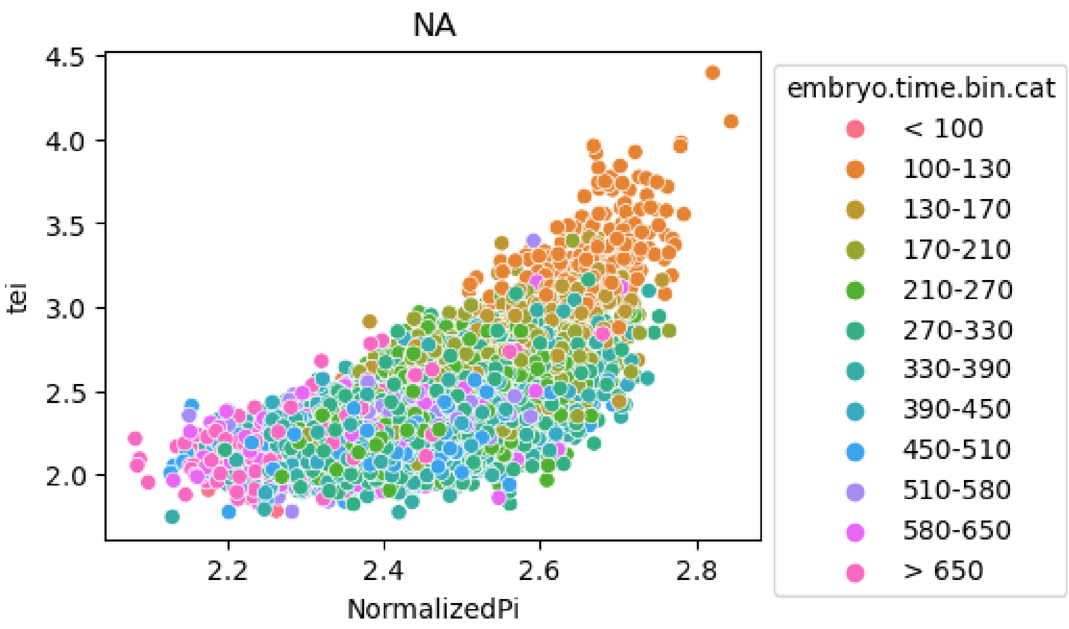
NA
tei
NormalizedPi
4.5
4.0
3.5
3.0
2.5
2.0
2.2
2.4
2.6
2.8
embryo.time.bin.cat
< 100
100-130
130-170
170-210
210-270
270-330
330-390
390-450
450-510
510-580
580-650
> 650

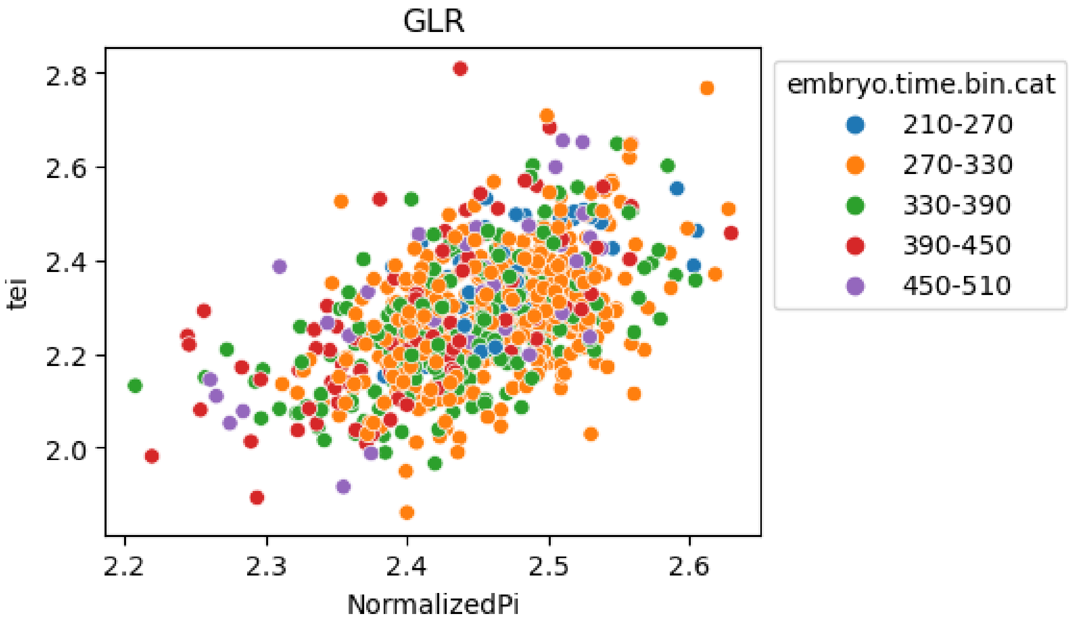
GLR
tei
NormalizedPi
2.8
2.6
2.4
2.2
2.0
2.2
2.3
2.4
2.5
2.6
embryo.time.bin.cat
210-270
270-330
330-390
390-450
450-510

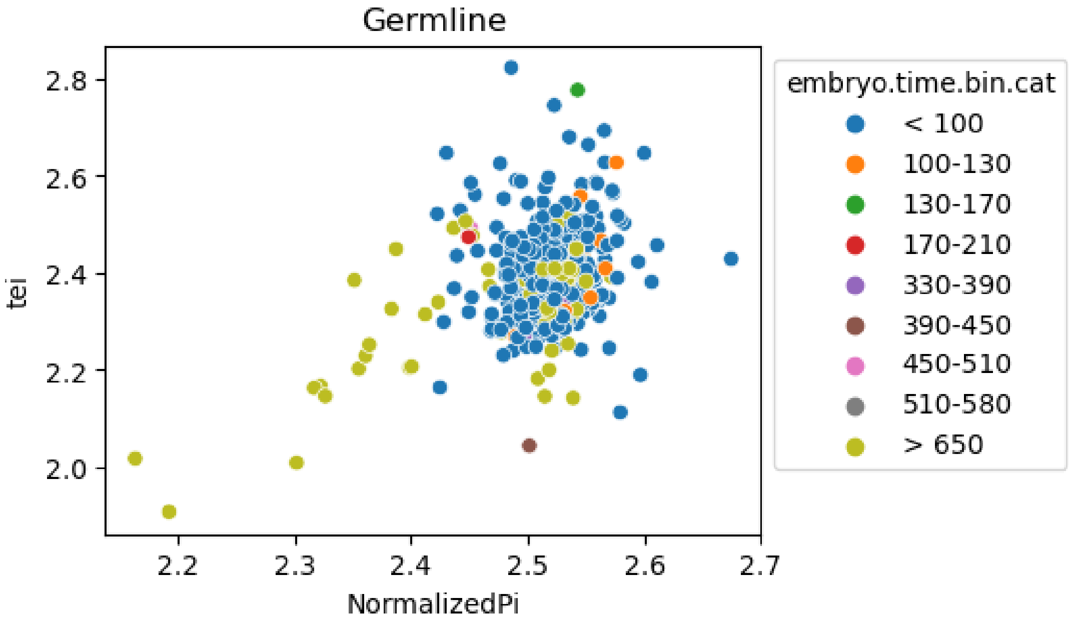
Germline
tei
2.8
2.6
2.4
2.2
2.0
2.2
2.3
2.4
2.5
2.6
2.7
NormalizedPi
embryo.time.bin.cat
< 100
100-130
130-170
170-210
330-390
390-450
450-510
510-580
> 650

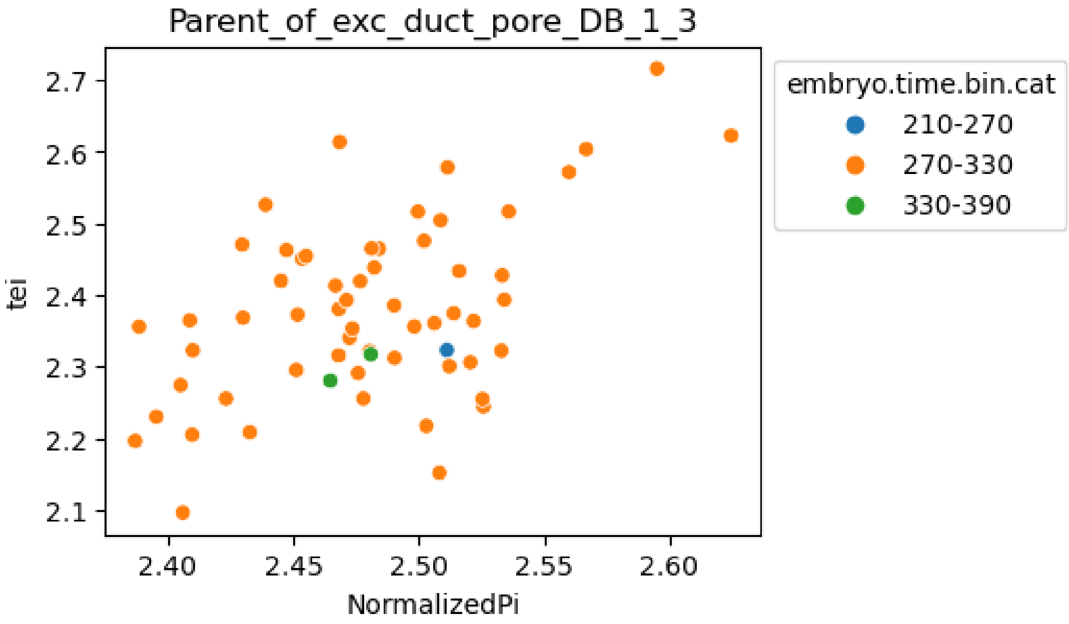
Parent_of_exc_duct_pore_DB_1_3
tei
2.7
2.6
2.5
2.4
2.3
2.2
2.1
2.40
2.45
2.50
2.55
2.60
NormalizedPi
embryo.time.bin.cat
210-270
270-330
330-390

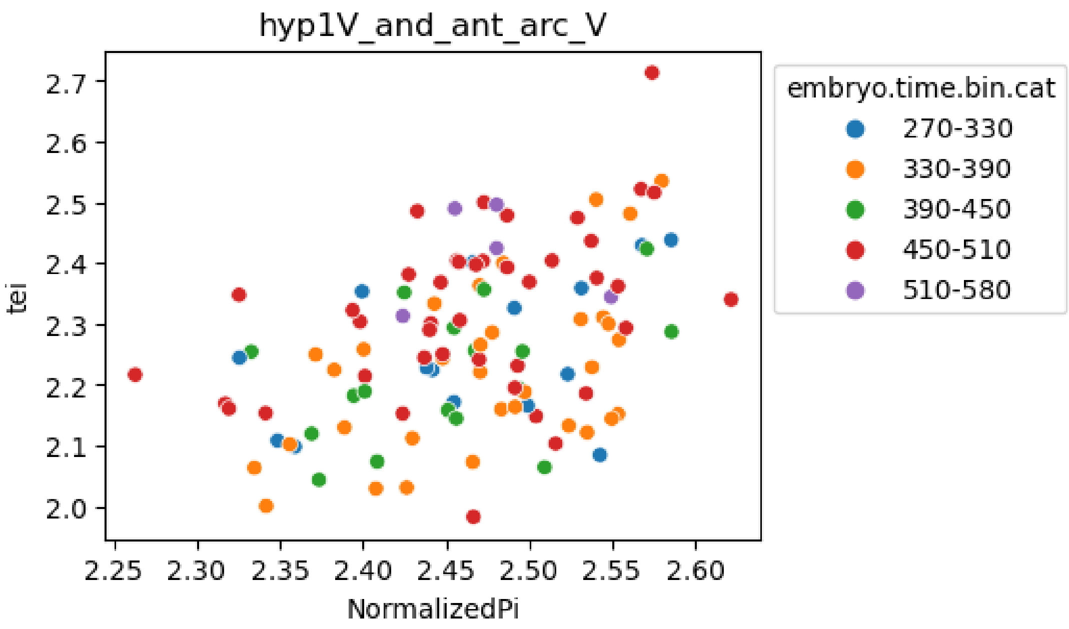
hyp1V_and_ant_arc_V
tei
NormalizedPi
2.7
2.6
2.5
2.4
2.3
2.2
2.1
2.0
2.25
2.30
2.35
2.40
2.45
2.50
2.55
2.60
embryo.time.bin.cat
270-330
330-390
390-450
450-510
510-580

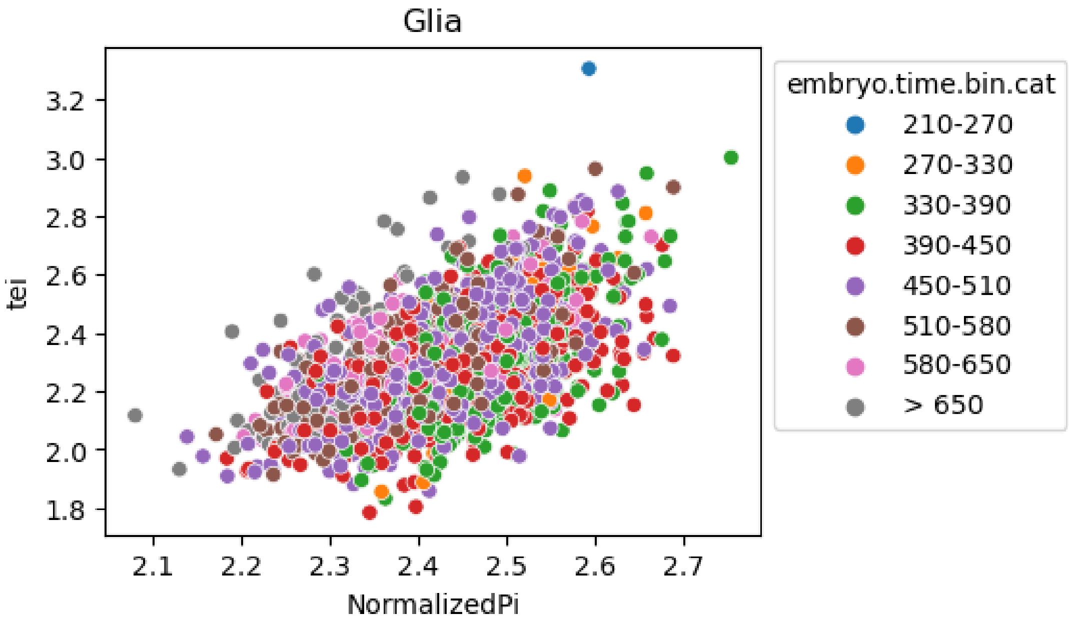
Glia
tei
NormalizedPi
3.2
3.0
2.8
2.6
2.4
2.2
2.0
1.8
2.1
2.2
2.3
2.4
2.5
2.6
2.7
embryo.time.bin.cat
210-270
270-330
330-390
390-450
450-510
510-580
580-650
> 650

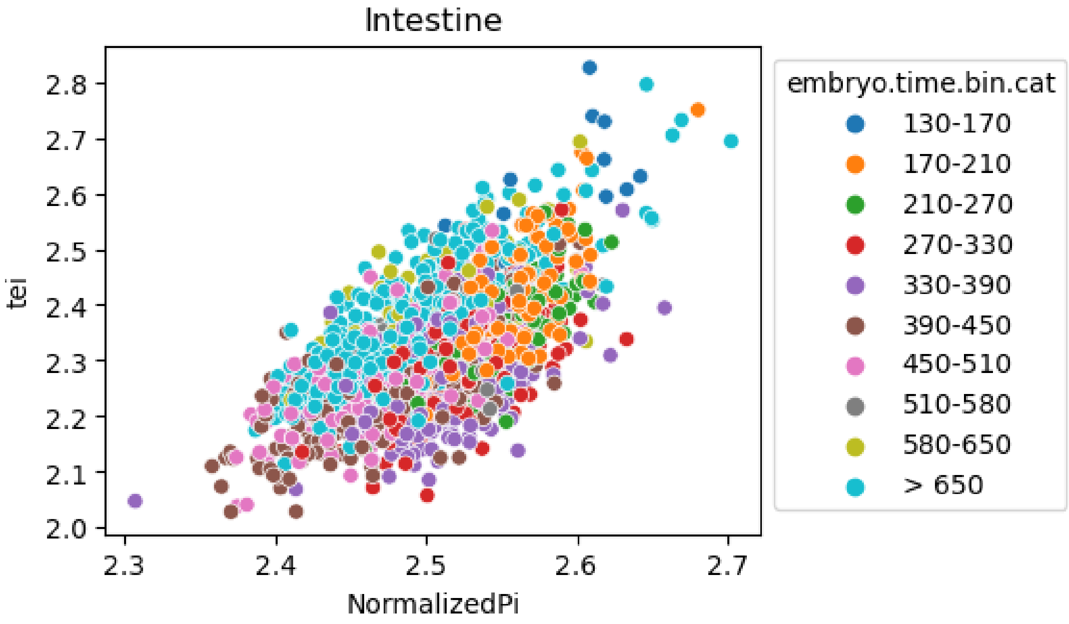
Intestine
tei
2.8
2.7
2.6
2.5
2.4
2.3
2.2
2.1
2.0
2.3
2.4
2.5
2.6
2.7
NormalizedPi
embryo.time.bin.cat
130-170
170-210
210-270
270-330
330-390
390-450
450-510
510-580
580-650
> 650

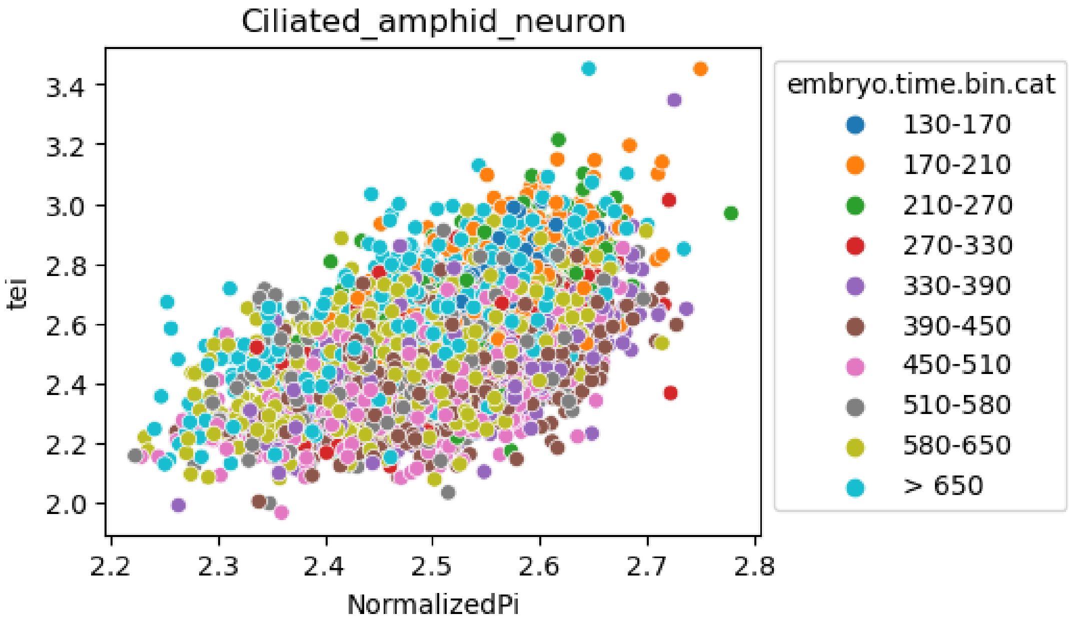
Ciliated_amphid_neuron
tei
3.4
3.2
3.0
2.8
2.6
2.4
2.2
2.0
2.2
2.3
2.4
2.5
2.6
2.7
2.8
NormalizedPi
embryo.time.bin.cat
130-170
170-210
210-270
270-330
330-390
390-450
450-510
510-580
580-650
> 650

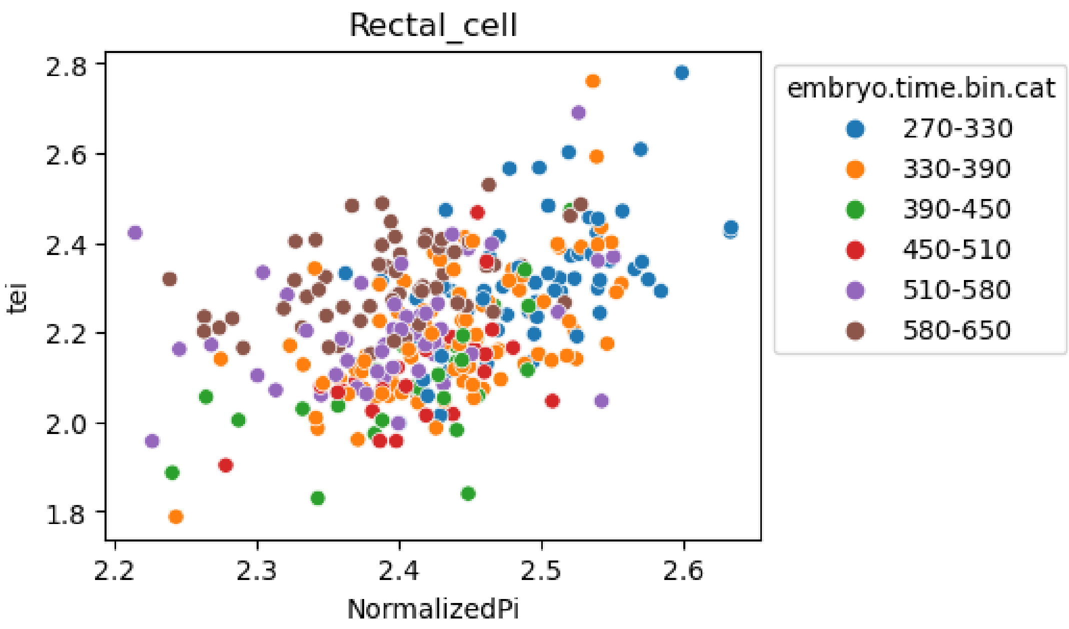
Rectal_cell
tei
NormalizedPi
embryo.time.bin.cat
270-330
330-390
390-450
450-510
510-580
580-650

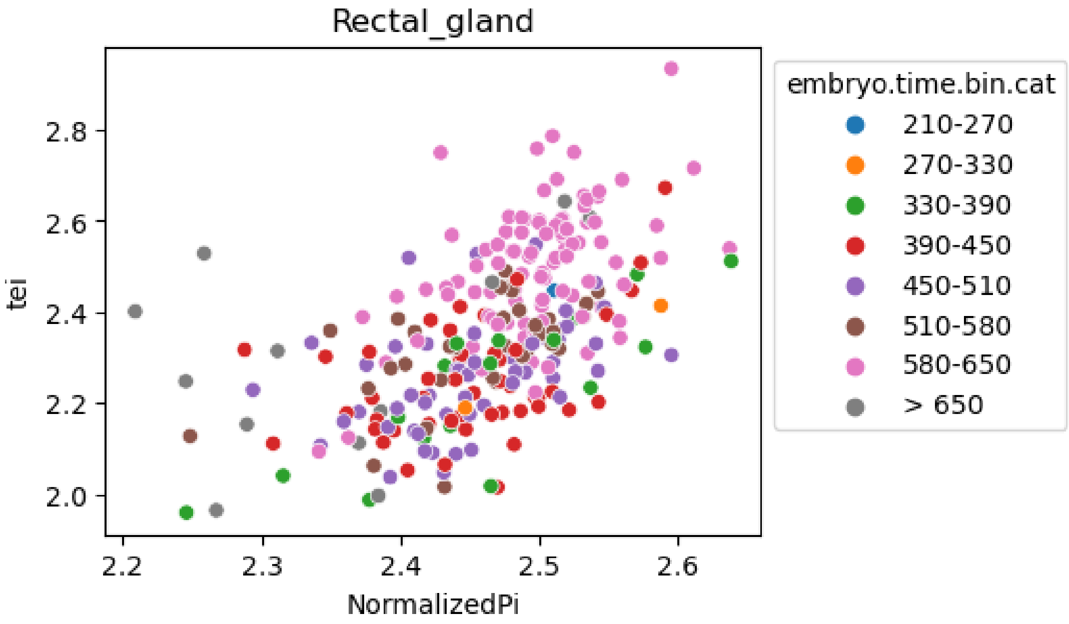
Rectal_gland
tei
NormalizedPi
embryo.time.bin.cat
210-270
270-330
330-390
390-450
450-510
510-580
580-650
> 650

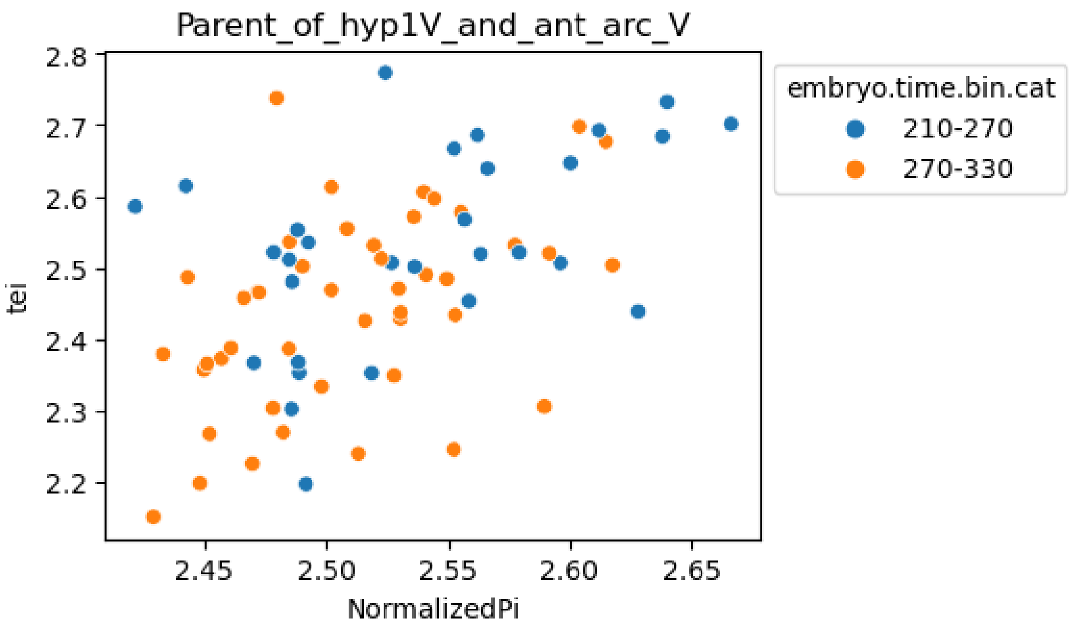

1.11.10 Plot relative expression per gene age class per sample timepoint

[30]:
```
celegans_data_rematrix_grouped = orthomap2tei.get_rematrix(
    adata=celegans_data,
    gene_id=query_orthomap['GeneID'],
    gene_age=query_orthomap['Phylostratum'],
    keep='min',
    layer=None,
    use='counts',
    var_type='mean',
    group_by_obs='embryo.time.bin.cat',
    obs_fillna='__NaN',
    obs_type='mean',
    standard_scale=0,
    normalize_total=True,
    log1p=True,
    target_sum=1e6)
celegans_data_rematrix_grouped
```

[30]:

embryo.time.bin.cat	< 100	100-130	130-170	170-210	210-270	\
ps						
0	0.717118	1.000000	0.584168	0.310426	0.302204	
1	1.000000	0.547242	0.477675	0.295885	0.250502	
2	1.000000	0.450475	0.387854	0.213375	0.168202	
3	1.000000	0.456666	0.312041	0.126569	0.089371	
4	1.000000	0.351679	0.245304	0.080961	0.056258	
5	1.000000	0.454323	0.381592	0.113054	0.024290	
6	1.000000	0.393570	0.331241	0.082090	0.019724	
7	1.000000	0.404537	0.296849	0.164328	0.114308	

```
8                    1.000000  0.374614  0.332113  0.229286  0.175440
9                    1.000000  0.421409  0.314549  0.205528  0.172487
10                   1.000000  0.473084  0.224513  0.014137  0.000000
11                   0.613516  1.000000  0.562404  0.273833  0.202082
12                   0.470709  1.000000  0.665627  0.351440  0.257487
13                   0.411756  1.000000  0.649935  0.344974  0.252738

embryo.time.bin.cat   270-330   330-390   390-450   450-510   510-580  \
ps
0                    0.260612  0.206768  0.105727  0.048908  0.000000
1                    0.231566  0.176154  0.112925  0.089850  0.033621
2                    0.154225  0.113436  0.062726  0.048594  0.026063
3                    0.076377  0.078763  0.018744  0.046053  0.002353
4                    0.050955  0.028812  0.000000  0.017548  0.057651
5                    0.000000  0.019198  0.011902  0.006195  0.088254
6                    0.015556  0.019509  0.000000  0.010253  0.100935
7                    0.089470  0.035970  0.005399  0.000000  0.015025
8                    0.116940  0.052691  0.001487  0.004222  0.003206
9                    0.141735  0.098434  0.040396  0.017756  0.011292
10                   0.042069  0.067210  0.077679  0.173604  0.316754
11                   0.145245  0.092380  0.043486  0.033280  0.000000
12                   0.176988  0.101492  0.025500  0.011935  0.000000
13                   0.172185  0.096022  0.034795  0.025026  0.004728

embryo.time.bin.cat   580-650     > 650
ps
0                    0.013732  0.073366
1                    0.000000  0.165599
2                    0.000000  0.171621
3                    0.000000  0.394810
4                    0.015902  0.256118
5                    0.077768  0.503433
6                    0.250751  0.605489
7                    0.024354  0.153953
8                    0.000000  0.105183
9                    0.000000  0.105804
10                   0.270774  0.566895
11                   0.003208  0.043322
12                   0.000159  0.055289
13                   0.000000  0.057878
```

[31]:
```
ax = sns.lineplot(celegans_data_rematrix_grouped.transpose(),\
    palette='tab20', dashes=False)
ax.legend(fontsize=5, title='age class')
ax.set_title('C. elegans - Relative expression per embryo time')
ax.set_xlabel('embryo.time.bin')
ax.set_ylabel('Relative expression level')
sns.move_legend(ax, 'upper left', bbox_to_anchor=(1, 1))
plt.xticks(rotation=90)
plt.show()
```

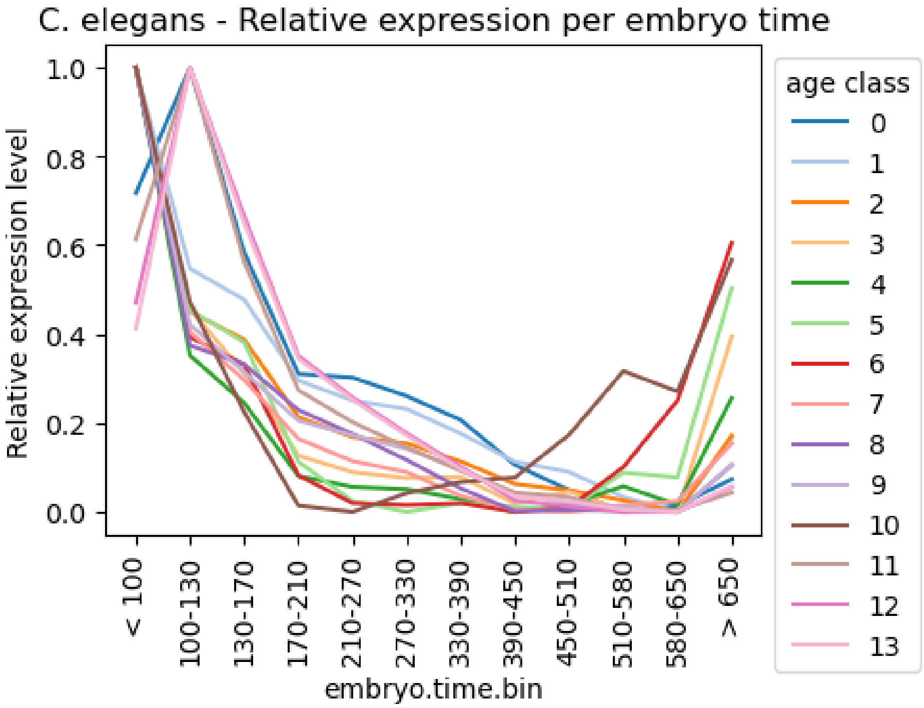

1.11.11 Get partial TEI values to visualize gene age class contributions

Partial `TEI` values can give an idea about which gene age class contributed at most to the `global TEI` pattern.

In detail, each gene gets a `TEI` contribution profile as follows:

$TEI_{is} = f_{is} * ps_i$

, where TEI_{is} is the partial `TEI` value of gene i, $f_{is} = e_{is}/\sum e_{is}$ and ps_i is the phylostratum of gene i.

TEI_{is} values are combined per ps.

The partial `TEI` values combined per strata give an overall impression of the contribution of each strata to the `global TEI` pattern.

One can either start from `counts` (`adata.X`) which is set as default or any other `layer` defined by the `layer` option (`layer=None`).

In addition, the `counts` can be `normalized` and `log-transformed` prior calculating partial `TEI` values (`normalize_total=False`, `log1p=False`, `target_sum=1e6`).

Further, these values can be combined per given observation, e.g. sample timepoint (`group_by='raw.embryo.time'`).

The `get_pstrata` function of the `orthomap2tei` submodule will return two matrix, the first contains the sum of each partial `TEI` per gene age class and the second the corresponding frequencies.

Both can be further processed by returning the `cumsum` over the gene age classes. To get them set the option `cumsum=True`. The `cumsum` will result in either for the first matrix the `TEI` value per cell or mean `TEI` value per group, if one choose a observation with the `group_by` option. Or in case of the second frequency matrix will result in 1.

With the `standard_scale` option either gene age classes (`standard_scale=0` rows) or cells or groups (`standard_scale=1` columns) can be scaled, subtract the minimum and divide each by its maximum. By default no scaling is applied (`standard_scale=None`).

The resulting data will be visualized in the downstream section.

[32]:
```
celegans_pstrata = orthomap2tei.get_pstrata(adata=celegans_data,
    gene_id=query_orthomap['GeneID'],
    gene_age=query_orthomap['Phylostratum'],
    keep='min',
    layer=None,
    cumsum=True,
    group_by_obs='embryo.time.bin.cat',
    obs_fillna='__NaN',
    obs_type='mean',
    standard_scale=None,
    normalize_total=True,
    log1p=True,
    target_sum=1e6)
celegans_pstrata[0]
```

[32]:
```
embryo.time.bin.cat     < 100   100-130   130-170   170-210   210-270  \
ps
0                    0.000000  0.000000  0.000000  0.000000  0.000000
1                    0.564379  0.530493  0.554769  0.564274  0.568691
2                    1.066948  0.969366  1.019430  1.040665  1.048817
3                    1.110209  1.010589  1.060572  1.084848  1.094679
4                    1.222601  1.106044  1.157717  1.187257  1.202083
5                    1.269068  1.150924  1.206516  1.236391  1.250166
6                    1.288148  1.168326  1.225392  1.255229  1.269197
7                    1.436406  1.275586  1.325999  1.352471  1.361118
8                    1.593160  1.379820  1.436263  1.467291  1.469098
9                    1.723074  1.475801  1.529523  1.564003  1.566499
10                   1.750491  1.499340  1.549016  1.580853  1.584175
11                   1.998728  2.001177  1.895514  1.864111  1.846383
12                   2.249283  2.611671  2.381169  2.269536  2.214056
13                   2.419276  3.114069  2.756838  2.561030  2.465832

embryo.time.bin.cat   270-330   330-390   390-450   450-510   510-580  \
ps
0                    0.000000  0.000000  0.000000  0.000000  0.000000
1                    0.574967  0.574761  0.572603  0.567071  0.540339
2                    1.063191  1.072649  1.085847  1.086674  1.085105
3                    1.110265  1.124388  1.139774  1.144051  1.145294
4                    1.221465  1.242487  1.266474  1.278308  1.303513
5                    1.269788  1.296496  1.326829  1.340107  1.379603
6                    1.289450  1.318017  1.350846  1.364751  1.410713
7                    1.378901  1.398513  1.431710  1.446642  1.507610
8                    1.473799  1.480398  1.503826  1.523043  1.589848
9                    1.568462  1.572929  1.588345  1.603053  1.674875
10                   1.588804  1.596092  1.614562  1.635971  1.721640
11                   1.824879  1.817629  1.822785  1.842534  1.912595
12                   2.144069  2.100194  2.057414  2.069561  2.143782
13                   2.348615  2.261164  2.178997  2.185036  2.246225
```

```
embryo.time.bin.cat   580-650     > 650
ps
0                    0.000000  0.000000
1                    0.525781  0.547670
2                    1.076311  1.078825
3                    1.139959  1.142614
4                    1.299550  1.289389
5                    1.379764  1.367366
6                    1.419942  1.404574
7                    1.527176  1.513817
8                    1.613239  1.603044
9                    1.700259  1.690460
10                   1.746472  1.738911
11                   1.954244  1.922988
12                   2.200446  2.149454
13                   2.300599  2.255455
```

corresponds to main figure Figure 1E

[33]:
```
plt.rcParams['figure.figsize'] = [6.5, 4.5]
ax = sns.lineplot(celegans_pstrata[0].transpose(), palette='tab20', dashes=False)
ax.legend(fontsize=3, title='age class')
ax.set_title('C. elegans - Contribution of gene age classes to global TEI')
ax.set_xlabel('embryo.time.bin.cat')
ax.set_ylabel('TEI')
sns.move_legend(ax, 'upper left', bbox_to_anchor=(1, 1))
plt.xticks(rotation=90)
plt.show()
plt.rcParams['figure.figsize'] = [4.4, 3.3]
```

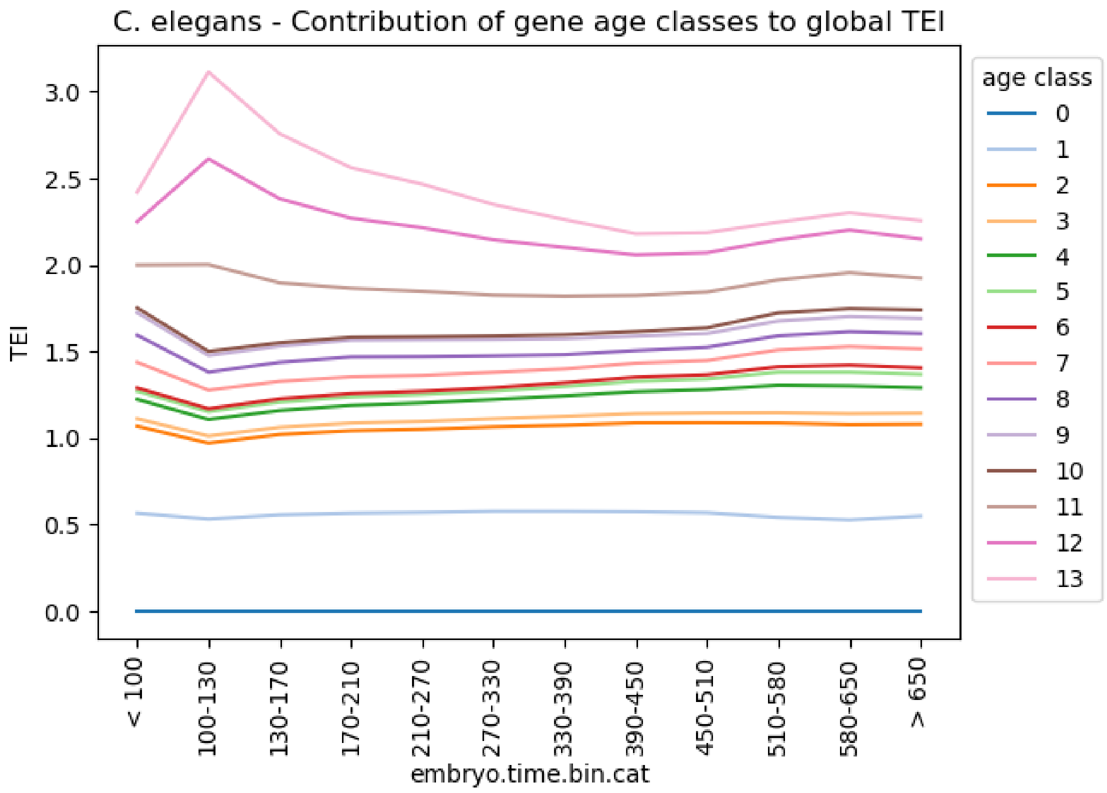

partial TajimaD **Note:** Please change notebook cell from `raw` to `code` to see the plots.

```
celegans_pstrata_tajimaD = orthomap2tei.get_pstrata(adata=celegans_data,
    gene_id=query_fst['WormBase_ID'],
    gene_age=query_fst['TajimaD_binned'],
    keep='min',
    layer=None,
    cumsum=False,
    group_by_obs='raw.embryo.time',
    obs_fillna='__NaN',
    obs_type='mean',
    standard_scale=None,
    normalize_total=True,
    log1p=True,
    target_sum=1e6)
celegans_pstrata_tajimaD[0]
```

Note: Please change notebook cell from `raw` to `code` to see the plots.

```
ax=celegans_pstrata_tajimaD[0].transpose().plot.area(cmap='tab20')
ax.legend(fontsize=3, title='age class')
ax.set_title('C. elegans - Contribution of TajimaD classes to global TajimaD')
ax.set_xlabel('raw.embryo.time')
ax.set_ylabel('TajimaD')
sns.move_legend(ax, 'upper left', bbox_to_anchor=(1, 1))
plt.xticks(rotation=90)
```

```
plt.show()
```

partial Fst **Note:** Please change notebook cell from `raw` to `code` to see the plots.

```
celegans_pstrata_Fst = orthomap2tei.get_pstrata(adata=celegans_data,
    gene_id=query_fst['WormBase_ID'],
    gene_age=query_fst['FST_binned'],
    keep='min',
    layer=None,
    cumsum=False,
    group_by_obs='raw.embryo.time',
    obs_fillna='__NaN',
    obs_type='mean',
    standard_scale=None,
    normalize_total=True,
    log1p=True,
    target_sum=1e6)
celegans_pstrata_Fst[0]
```

Note: Please change notebook cell from `raw` to `code` to see the plots.

```
ax=celegans_pstrata_Fst[0].transpose().plot.area(cmap='tab20')
ax.legend(fontsize=3, title='age class')
ax.set_title('C. elegans - Contribution of Fst classes to global Fst')
ax.set_xlabel('raw.embryo.time')
ax.set_ylabel('Fst')
sns.move_legend(ax, 'upper left', bbox_to_anchor=(1, 1))
plt.xticks(rotation=90)
plt.show()
```

partial NormalizedPi **Note:** Please change notebook cell from `raw` to `code` to see the plots.

```
celegans_pstrata_normPi = orthomap2tei.get_pstrata(adata=celegans_data,
    gene_id=query_fst['WormBase_ID'],
    gene_age=query_fst['NormalizedPi_binned'],
    keep='min',
    layer=None,
    cumsum=False,
    group_by_obs='raw.embryo.time',
    obs_fillna='__NaN',
    obs_type='mean',
    standard_scale=None,
    normalize_total=True,
    log1p=True,
    target_sum=1e6)
celegans_pstrata_normPi[0]
\endn{verbatim}
    \textbf{Note:} Please change notebook cell from \texttt{raw} to
\texttt{code} to see the plots.
\begin{verbatim}
ax=celegans_pstrata_normPi[0].transpose().plot.area(cmap='tab20')
ax.legend(fontsize=3, title='age class')
ax.set_title('C. elegans - Contribution of NormalizedPi classes to global NormalizedPi')
ax.set_xlabel('raw.embryo.time')
ax.set_ylabel('NormalizedPi')
sns.move_legend(ax, 'upper left', bbox_to_anchor=(1, 1))
```

```
plt.xticks(rotation=90)
plt.show()
```

1.11.12 Color UMAP/TSNE by TEI

Follwoing the basic tutorial of the Scanpy python toolkit (Wolf et al., 2018), one can highlight `TEI` values on a dimensional reduction of the scRNA dataset, like `PCA`, `UMAP` or `TSNE`.

Filtering

[34]:
```
sc.pp.filter_genes(celegans_data, min_cells=3)
sc.pp.filter_cells(celegans_data, min_genes=200)
```

Normalization, Log transformation and Scaling

[35]:
```
sc.pp.normalize_total(celegans_data, target_sum=1e6)
sc.pp.log1p(celegans_data)
sc.pp.scale(celegans_data, max_value=10)
```

PCA and Neighbor calculations

[36]:
```
sc.tl.pca(celegans_data, svd_solver='arpack')
sc.pl.pca(celegans_data, color=['embryo.time.bin.cat', 'tei'])
```

```
/opt/anaconda3/envs/scanpy/lib/python3.8/site-
packages/scanpy/plotting/_tools/scatterplots.py:392: UserWarning: No data for
colormapping provided via 'c'. Parameters 'cmap' will be ignored
  cax = scatter(
```

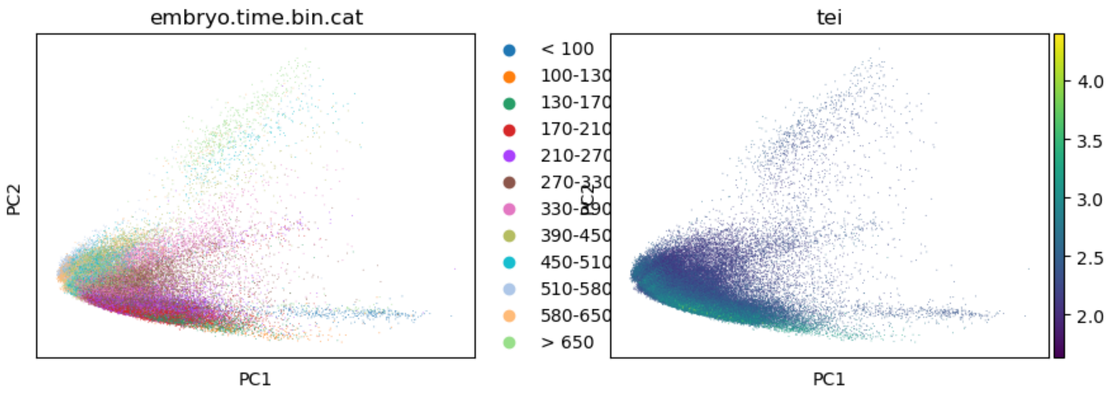

[37]:
```
sc.pp.neighbors(celegans_data)
```

Embedding the neighborhood graph

[38]:
```
sc.tl.paga(celegans_data, groups='embryo.time.bin.cat')
sc.pl.paga(celegans_data, title='C. elegans - embryo stage - PAGA graph')
```

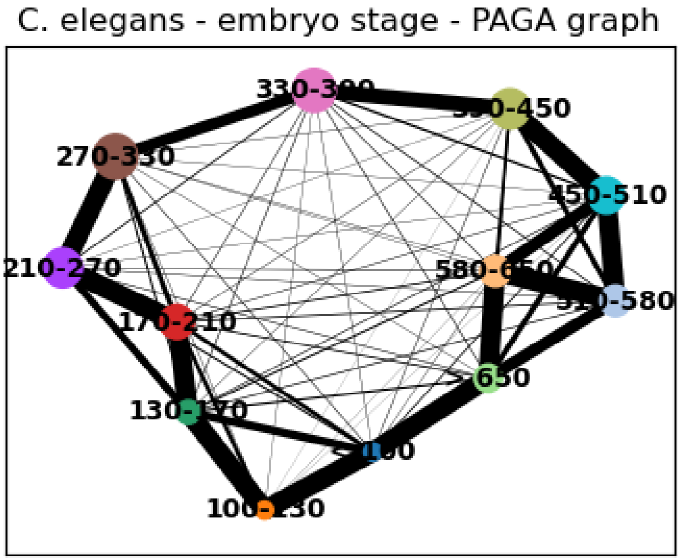

[39]:
```
sc.pl.paga(celegans_data,\
    title='C. elegans - embryo stage - PAGA graph', color=['tei'])
```

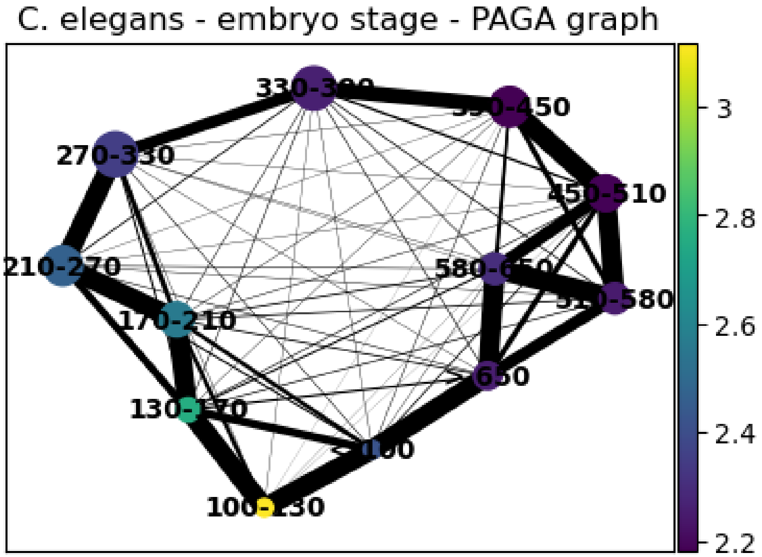

UMAP

[40]:
```
sc.tl.umap(celegans_data,
           init_pos='paga')
sc.pl.umap(celegans_data,
           title='C. elegans - embryo stage - UMAP', color=['embryo.time.bin.cat'])
```

/opt/anaconda3/envs/scanpy/lib/python3.8/site-

```
packages/scanpy/plotting/_tools/scatterplots.py:392: UserWarning: No data for
colormapping provided via 'c'. Parameters 'cmap' will be ignored
  cax = scatter(
```

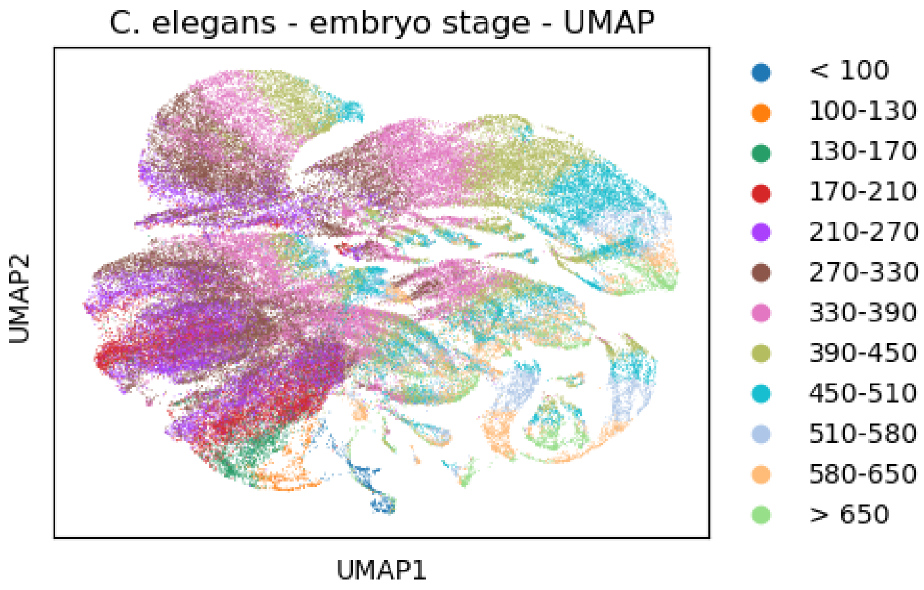

Color UMAP by cell type

[41]:
```
sc.pl.umap(celegans_data,
           title='C. elegans - cell type - UMAP', color=['cell.type'])
```

```
/opt/anaconda3/envs/scanpy/lib/python3.8/site-
packages/scanpy/plotting/_tools/scatterplots.py:392: UserWarning: No data for
colormapping provided via 'c'. Parameters 'cmap' will be ignored
  cax = scatter(
```

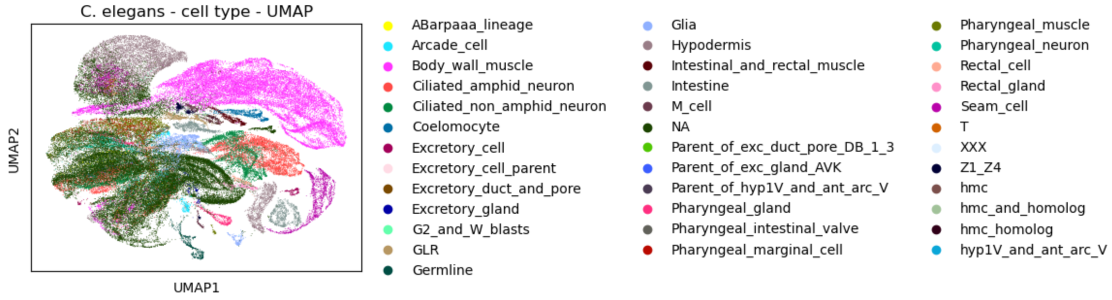

Color UMAP by TEI

[42]:
```
#plt.rcParams['figure.figsize'] = [7.5, 4.5]
sc.pl.umap(celegans_data,
           title='C. elegans - TEI - UMAP',
```

```
           color=['tei'],
           color_map='viridis',
           vmin='p5',
           vmax='p95')
#plt.rcParams['figure.figsize'] = [6, 4.5]
```

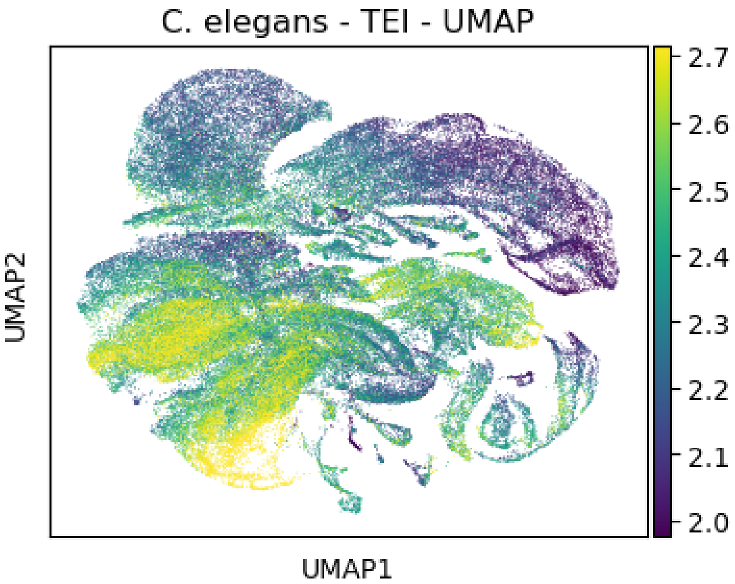

Color UMAP by TajimaD bins

[43]:
```
#plt.rcParams['figure.figsize'] = [7.5, 4.5]
sc.pl.umap(celegans_data,
           title='C. elegans - TajimaD bins - UMAP',
           color=['TajimaD'],
           color_map='terrain',
           vmin='p5',
           vmax='p95')
#plt.rcParams['figure.figsize'] = [6, 4.5]
```

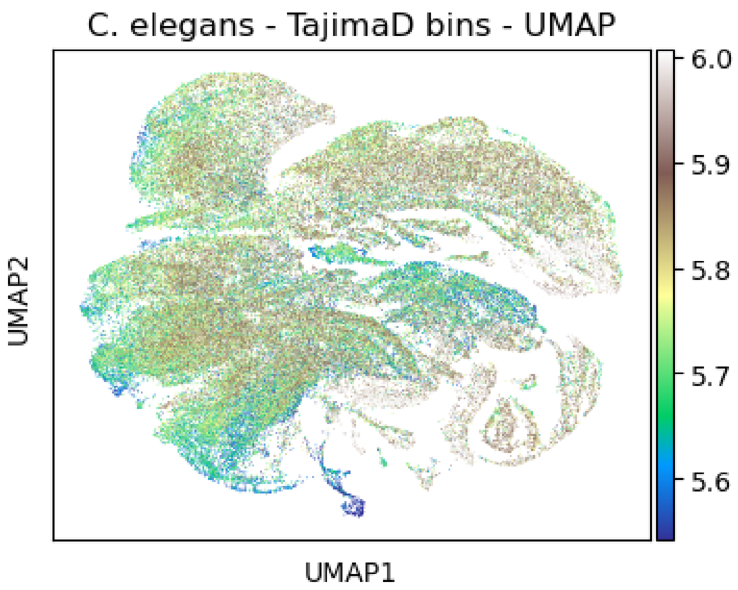

Color UMAP by Fst bins

[44]:
```
#plt.rcParams['figure.figsize'] = [7.5, 4.5]
sc.pl.umap(celegans_data,
           title='C. elegans - Fst bins - UMAP',
           color=['Fst'],
           color_map='tab20c',
           vmin='p5',
           vmax='p95')
#plt.rcParams['figure.figsize'] = [6, 4.5]
```

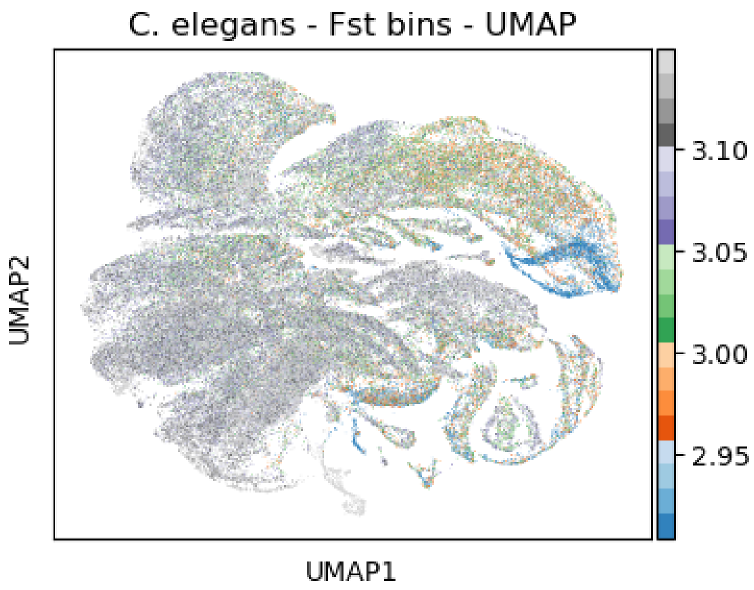

Color UMAP by NormalizedPi bins

[45]:
```
#plt.rcParams['figure.figsize'] = [7.5, 4.5]
sc.pl.umap(celegans_data,
           title='C. elegans - NormalizedPi bins - UMAP',
           color=['NormalizedPi'],
           color_map='RdBu',
           vmin='p5',
           vmax='p95')
#plt.rcParams['figure.figsize'] = [6, 4.5]
```

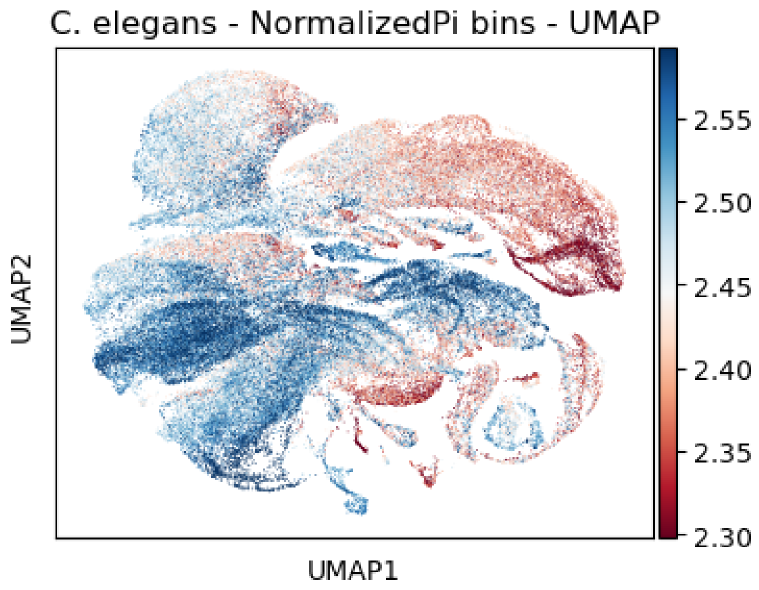

3D-UMAP

[46]:
```
plt.rcParams['figure.figsize'] = [7.5, 4.5]
#3d
sc.tl.umap(celegans_data,
           n_components=3)
sc.pl.umap(celegans_data,
           title='C. elegans - embryo stage - UMAP',
           color=['embryo.time.bin.cat'],
           projection='3d')
plt.rcParams['figure.figsize'] = [4.4, 3.3]
```

```
/opt/anaconda3/envs/scanpy/lib/python3.8/site-
packages/scanpy/plotting/_tools/scatterplots.py:325: UserWarning: No data for
colormapping provided via 'c'. Parameters 'cmap' will be ignored
  cax = ax.scatter(
```

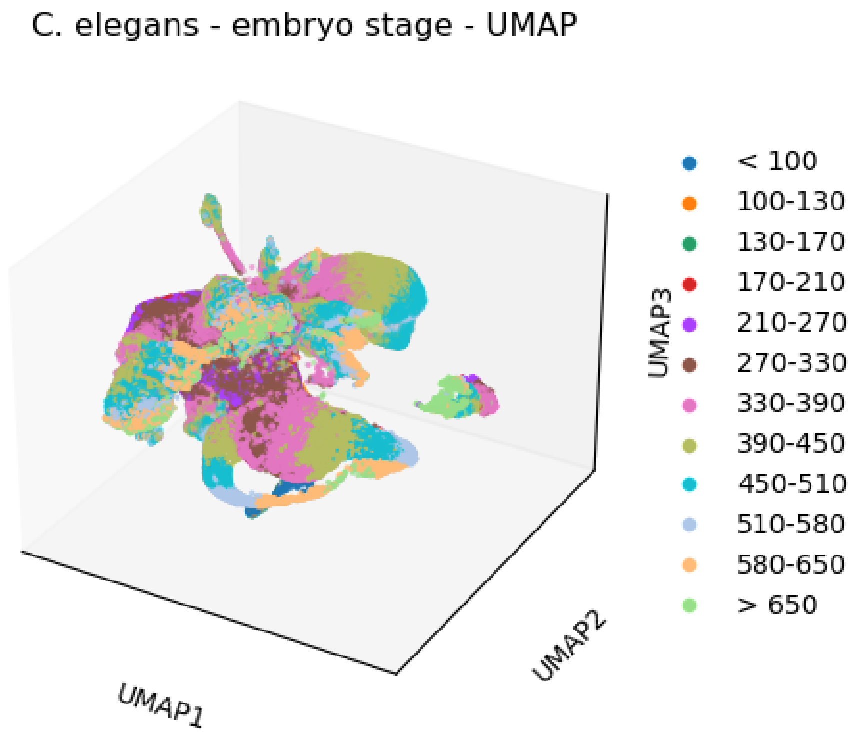

[47]:
```
plt.rcParams['figure.figsize'] = [7.5, 4.5]
#3d
sc.pl.umap(celegans_data,
           title='C. elegans - cell type - UMAP',
           color=['cell.type'],
           projection='3d')
plt.rcParams['figure.figsize'] = [4.4, 3.3]
```

```
/opt/anaconda3/envs/scanpy/lib/python3.8/site-
packages/scanpy/plotting/_tools/scatterplots.py:325: UserWarning: No data for
colormapping provided via 'c'. Parameters 'cmap' will be ignored
  cax = ax.scatter(
```

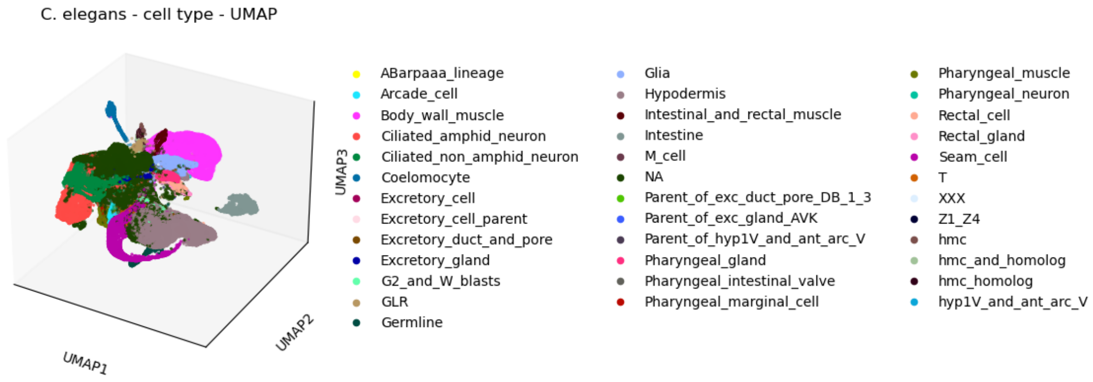

[48]:
```
plt.rcParams['figure.figsize'] = [7.5, 4.5]
#3d
sc.pl.umap(celegans_data,
           title='C. elegans - TEI - UMAP',
           color=['tei'],
           color_map='viridis',
           vmin='p5',
           vmax='p95',
           projection='3d')
plt.rcParams['figure.figsize'] = [4.4, 3.3]
```

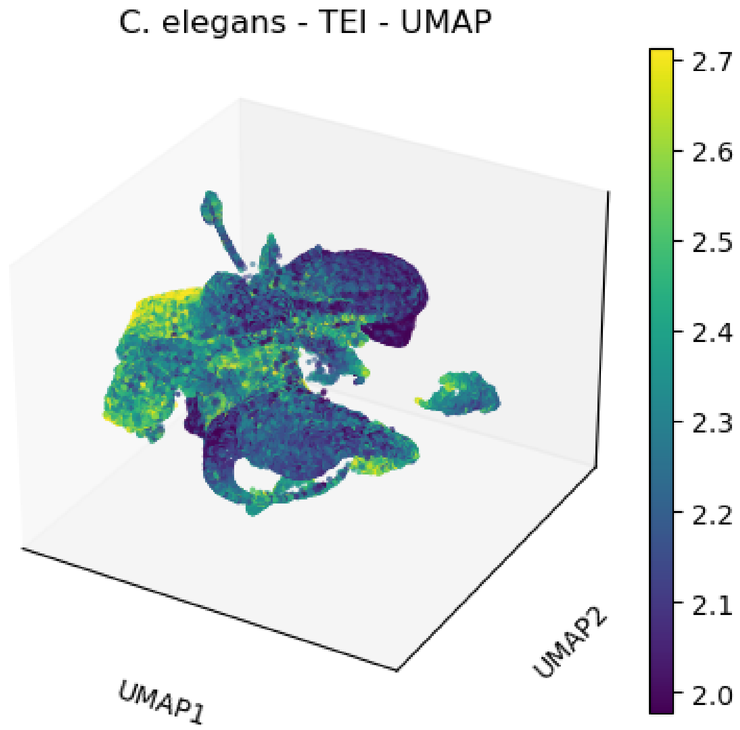

[49]:
```
plt.rcParams['figure.figsize'] = [7.5, 4.5]
#3d
sc.pl.umap(celegans_data,
           title='C. elegans - TajimaD bins - UMAP',
           color=['TajimaD'],
           color_map='terrain',
           vmin='p5',
           vmax='p95',
           projection='3d')
plt.rcParams['figure.figsize'] = [4.4, 3.3]
```

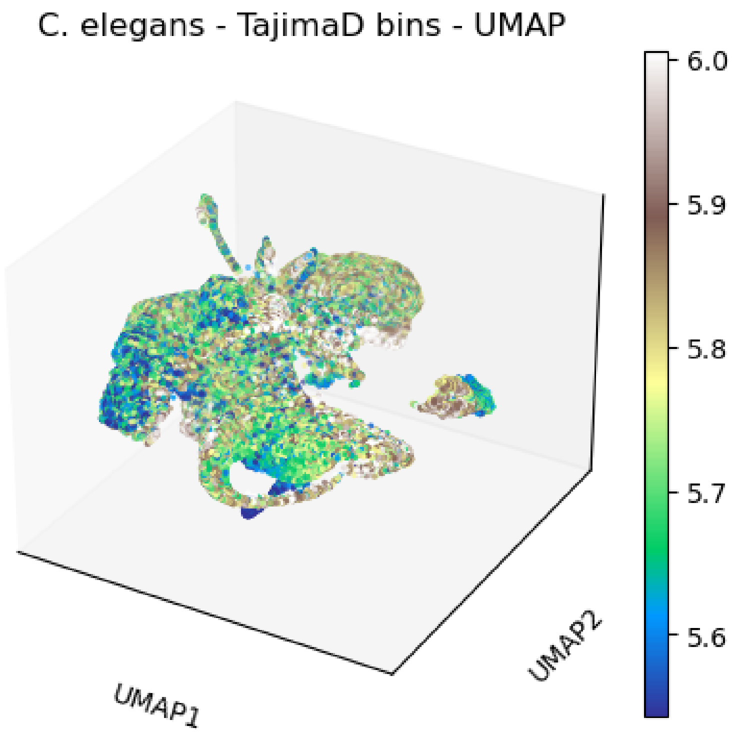

[50]:
```
plt.rcParams['figure.figsize'] = [7.5, 4.5]
sc.pl.umap(celegans_data,
           title='C. elegans - Fst bins - UMAP',
           color=['Fst'],
           color_map='tab20c',
           vmin='p5',
           vmax='p95',
           projection='3d')
plt.rcParams['figure.figsize'] = [4.4, 3.3]
```

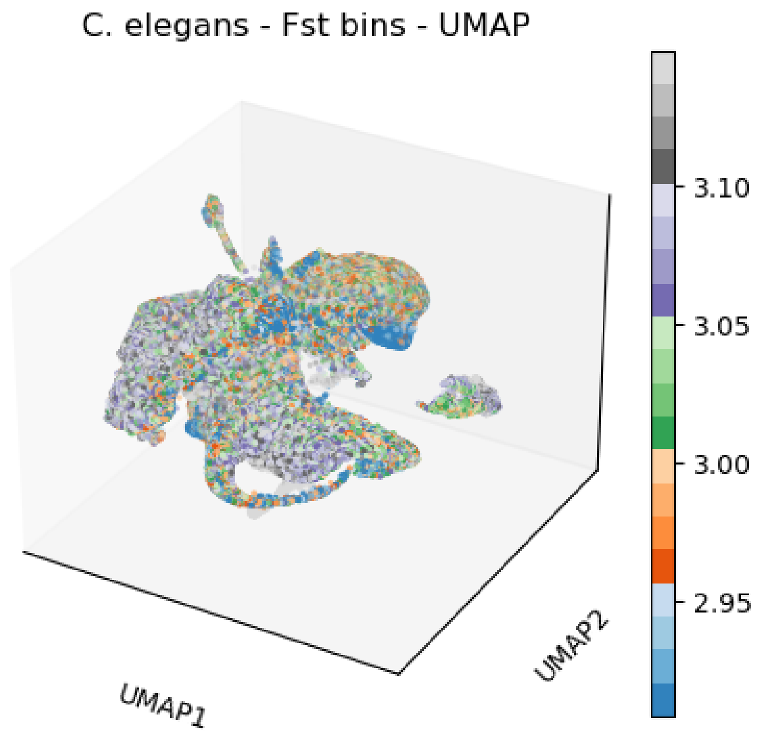

[51]:
```
plt.rcParams['figure.figsize'] = [7.5, 4.5]
#3d
sc.pl.umap(celegans_data,
           title='C. elegans - NormalizedPi bins - UMAP',
           color=['NormalizedPi'],
           color_map='RdBu',
           vmin='p5',
           vmax='p95',
           projection='3d')
plt.rcParams['figure.figsize'] = [4.4, 3.3]
```

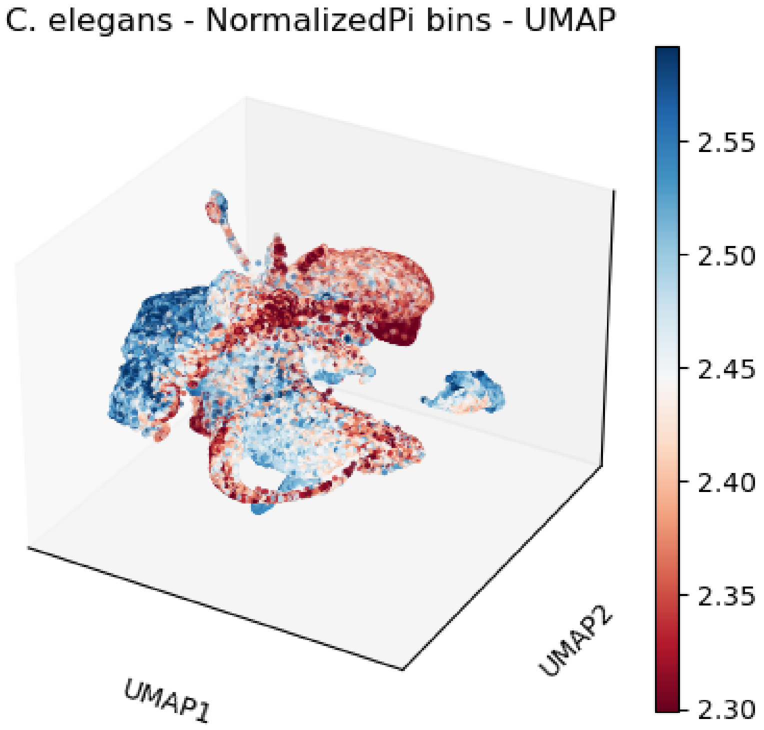

Please have a look at the documentation for other case studies.
